# Supplementary material for: Computational design of high-performance ligand for enantioselective Markovnikov hydroboration of aliphatic terminal alkenes
Source: Nat Commun. 2018 Jun 12;9:2290. doi: 10.1038/s41467-018-04693-9 (PMC5997753; doi:10.1038/s41467-018-04693-9)
Supplement: Supplementary file 1 — Supplementary Information [file 41467_2018_4693_MOESM1_ESM.pdf]

Supplementary Methods

Instrumentation and Chemicals

Materials were obtained from commercial suppliers and purified by standard procedures unless otherwise noted. Solvents were also purchased from commercial suppliers, degassed via three freeze-pump-thaw cycles, and further dried over molecular sieves (MS 4A). NMR spectra were recorded on JEOL JNM-ECX400P and JNM-ECS400 spectrometers (^{1}H: 400 MHz, ^{13}C: 100 MHz, ^{31}P: 160 MHz). Tetramethylsilane (^{1}H), $CDCl_3$ (^{13}C) and H_3PO_4 in D_2O (^{31}P) were employed as the external standards, respectively. CuCl (ReagentPlus® grade, 224332-25G, ≥99%) and K(O-*t*-Bu)/THF (1.0 M, 328650-50ML) were purchased from Sigma-Aldrich Co. and used as received. GLC analyses were conducted with a Shimadzu GC-2014 or GC-2025 equipped with a ULBON HR-1 glass capillary column (Shinwa Chemical Industries) and a FID detector. HPLC analyses with chiral stationary phase were carried out using a Hitachi LaChrome Elite HPLC system with a L-2400 UV detector. High-resolution mass spectra were recorded at the Global Facility Center, Hokkaido University. Single crystal X-ray structural analyses were carried out on a Rigaku R-AXIS RAPID diffractometer using graphite monochromated Mo-Kα radiation. The structure was solved by direct methods and expanded using Fourier techniques. Non-hydrogen atoms were refined anisotoropically. Hydrogen atoms were refined using the riding model. All calculations were performed using the CrystalStructure crystallographic software package except for refinement, which was performed using SHELXL-97.

General Borylation Procedures

Procedure for the Enantioselective Hydroboration of 1a with CuCl (Supplementary Table 1). Copper chloride (1.2 mg, 0.0125 mmol), bis(pinacolato)diboron (**2**) (76.2 mg, 0.30 mmol) and chiral ligand (0.0125 mmol) were placed in an oven-dried reaction vial. After the vial was sealed with a screw cap containing a Teflon™-coated rubber septum, the vial was connected to a vacuum/nitrogen manifold through a needle. It was evacuated and then backfilled with nitrogen. This cycle was repeated three times. Dry THF (0.20 mL) and K(O-*t*-Bu)/THF (1.00 M, 0.30 mL, 0.30 mmol) were added in the vial through the rubber septum using a syringe. After stirring for 30 min, **1a** (33.0 mg, 0.25 mmol) and methanol (0.0404 mL, 1.0 mmol) were added to the mixture. After the reaction was complete, the reaction mixture was passed through a short silica gel (Φ: 10 mm, height of the silica-gel column: 30 mm) eluting with Et_2O. The yield and regioselectivity of **3a** were determined by GC analysis with an internal standard using the crude material. The crude material was treated with $NaBO_3$•$4H_2O$ as an oxidant for the boryl group in THF/H_2O (1:1). The mixture was stirred at room temperature. After the reaction complete, the reaction mixture was extracted with Et_2O and dried over $MgSO_4$. The solvents were removed under reduced pressure. The crude material was purified by flash column chromatography (SiO_2, EtOAc/hexane, 0:100–20:80) to give the corresponding alcohol as a colorless oil.

Procedure for the Enantioselective Hydroboration of 1 with [Cu(MeCN)$_4$]BF$_4$ (Table 1, 2 and Supplementary Table 1). [Cu(MeCN)$_4$]BF$_4$ (4.7 mg, 0.015 mmol), bis(pinacolato)diboron (**2**) (152.4 mg, 0.60 mmol) and chiral ligand (0.015 mmol) were placed in an oven-dried reaction vial. After the vial was sealed with a screw cap containing a Teflon™-coated rubber septum, the vial was connected to a vacuum/nitrogen manifold through a needle. It was evacuated and then backfilled with nitrogen. This cycle was repeated three times. Dry THF (0.40 mL) and K(O-*t*-Bu)/THF (1.00 M, 0.60 mL, 0.60 mmol) were added in the vial through the rubber septum using a syringe. After stirring for 30 min at –40 ºC, **1** (0.50 mmol) and methanol (0.0404 mL, 1.0 mmol) were added to the mixture at –40 °C. After the reaction was complete, the reaction mixture was passed through a short silica gel (Φ: 10 mm, height of the silica-gel column: 30 mm) eluting with Et_2O. The regioselectivity of **3** was determined by GC analysis. The crude material was purified by flash column chromatography (SiO_2, Et_2O/hexane, typically 0:100–3:97) to give the corresponding alkylboronate (*S*)-**3**. The enantioselectivity was determined by HPLC after the stereospecific derivatization of the boryl group.

Supplementary Table 1. Chiral ligand screening of enantioselective Markovnikov hydroboration of aliphatic terminal alkene **1a**.

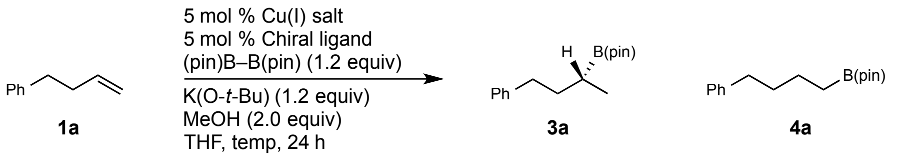

entry	Cu(I) salt	chiral ligand	temp (ºC)	time (h)	yield (%) of **3a**[a]	**3**/**4**[a]	ee (%) of **3a**[b]
1	CuCl	(*R,S*)-Joshiphos	–10	48	3	-	-
2	CuCl	(R_p,R_p)-Mandyphos	–10	72	7	83:17	-
3	CuCl	(*R*,*R*)-Et-Ferrotane	–10	48	31	42:58	52
4	CuCl	(*R*)-DIPAMP	–10	48	<1	-	-
5	CuCl	(*R*)-Phanephos	–10	72	5	-	-
6	CuCl	(*R*,*R*)-Me-Duphos	–10	24	41	46:54	59
7	CuCl	(*R*,*R*)-*i*-Pr-Duphos	0	24	7	64:36	-
8	CuCl	(*R*,*R*)-*i*-Pr-Duphos	rt	24	48	56:44	73
9	CuCl	(*R*)-MOP	rt	72	16	71:29	0
10	CuCl	(*R*,*R*)-DACH-Ph	rt	72	10	88:12	29
11	CuCl	(*S*)-DTMB-Segphos	–10	48	32	86:14	–8
12	CuCl	(*R*,*R*,*S*,*S*)-Tangphos	–10	48	11	27:73	18
13	CuCl	(*R*,*R*,*S*,*S*)-Duanphos	–10	48	8	15:85	29
14	CuCl	(*S*,*S*)-QuinoxP*	–10	24	24	24:76	–73
15	CuCl	(*S*)-Quinox-*t*Bu_3	–10	24	55	65:35	–87
16[c]	$[Cu(MeCN)_4]BF_4$	(*S*)-Quinox-*t*Bu_3	–40	24	70	72:28	–87
17[c]	$[Cu(MeCN)_4]BF_4$	(*S*,*S*)-QuinoxP*	–40	24	12	30:70	–54

[a]Determined by GC ananlysis with an internal standard using the crude material. [b]Determined by HPLC analysis after oxidation of **3a**. [c]3 mol % catalyst loading.

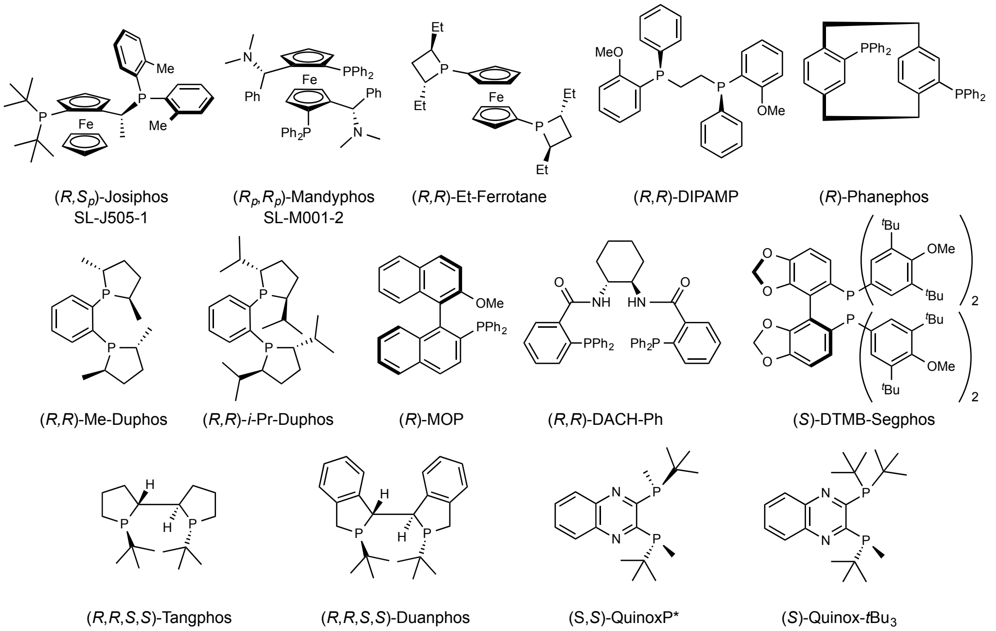

Supplementary Table 2. Evaluation of three-hindered quadrant bisphosphine ligands.

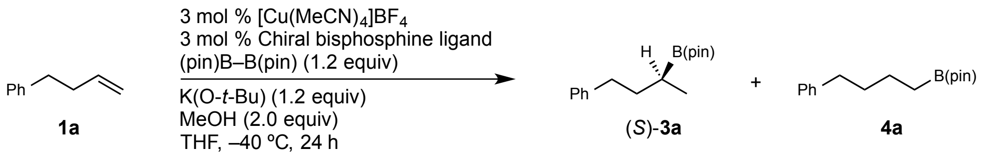

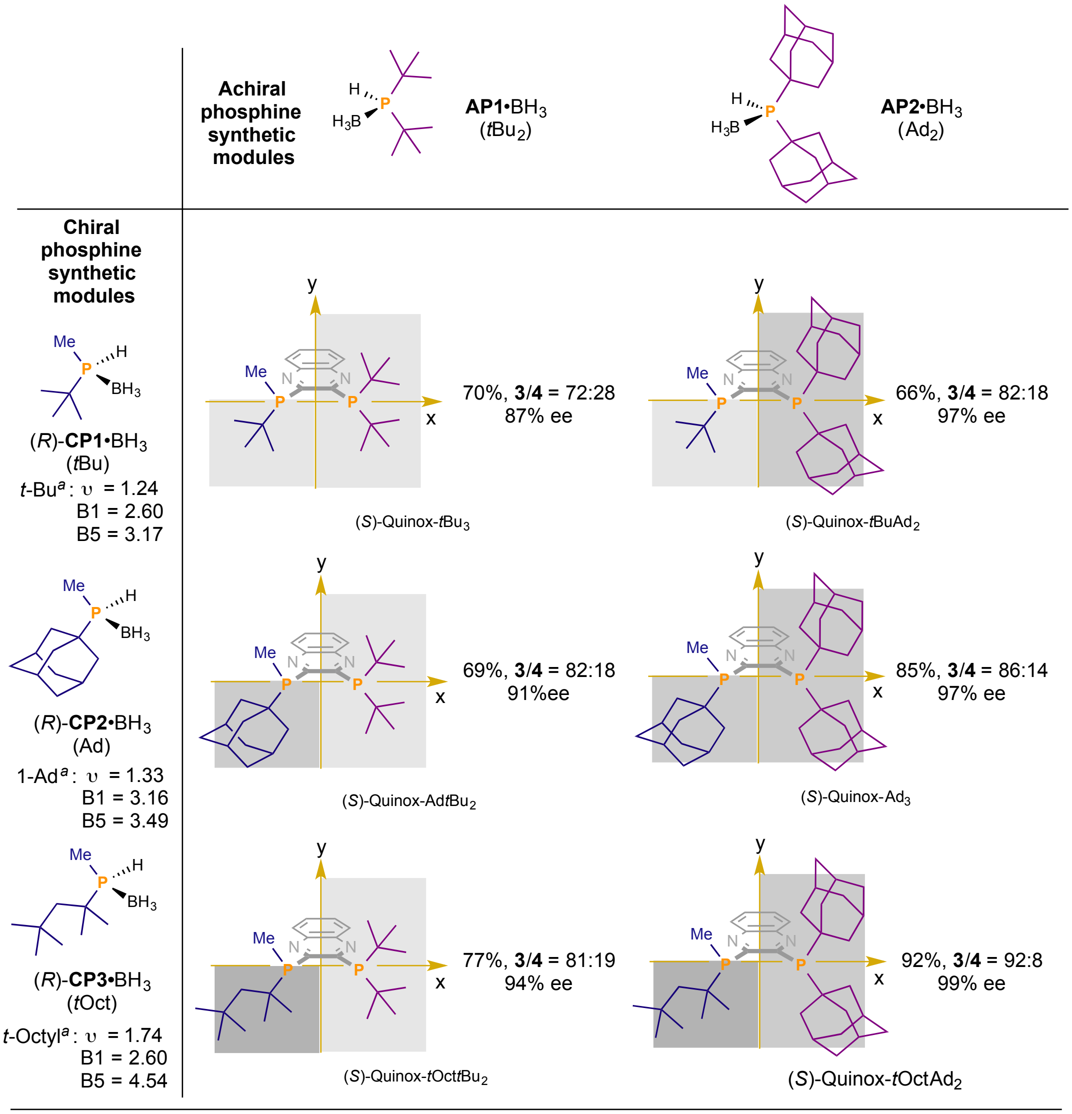

[a]All steric parameters are literature values[1–3]. υ: Charton's value; B1: Sterimol B1 parameter; B5: Sterimol parameter.

Preparation of Three-Hindered Quadrant Chiral Bisphosphine Ligands

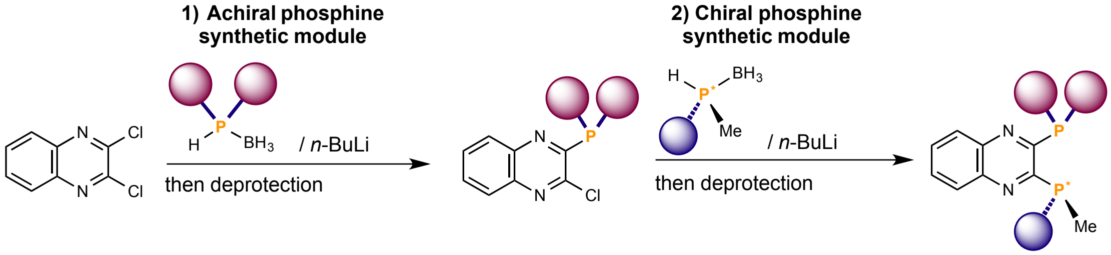

Supplementary Figure 1. General synthetic scheme of modular synthesis of chiral bisphosphine ligand.

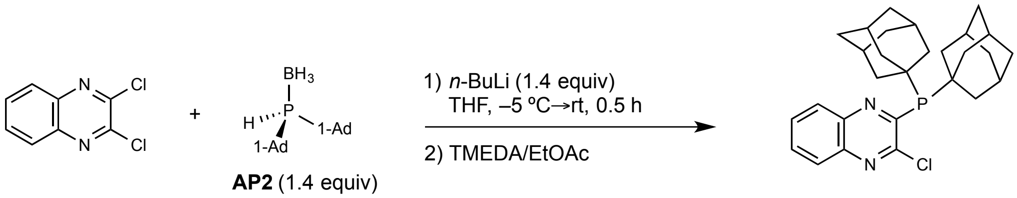

Preparation of 2-[(diadamantan-1-yl)phosphanyl]-3-chloroquinoxaline. The nucleophilic phosphination was performed according to the literature procedure[4]. A hexane solution of *n*-BuLi (1.64 M, 1.68 mmol, 1.02 mL) was added to a solution of (1-Ad)$_2$PH•BH$_3$ (1.68 mmol, 531 mg) in dry THF (3.6 mL) at –5 ºC under nitrogen atmosphere. After the additon of THF (4.0 mL), the resulting solution was added to a solution of 2,3-dichloroquinoxaline (1.2 mmol, 239 mg) in THF (4.8 mL) at 0 ºC. Then, the mixture was warmed to room temperature and stirred for 30 min. The reaction mixture was quenched by H_2O and the mixture was extracted with *n*-hexane three times. The combined organic layer was then dried over Na_2SO_4. After filtration, the solvents were removed under reduced pressure. TMEDA (1.2 mL) and EtOAc (2.4 mL) was added to the residual oil at room temperature. After stirring for 3 h, the reaction mixture was diluted with EtOAc and washed with 1.5 M aqueous HCl solution, water and brine subsequently. The organic layer was dried over Na_2SO_4. After filtration, the solutions solvents were removed under reduced pressure. The crude material was purified by flash column chromatography (SiO_2, Et_2O/hexane, 0:100–2:98) to give the corresponding phosphine as a yellow solid (0.93 mmol, 434 mg, 78%).

^{1}H NMR (392 MHz, $CDCl_3$, δ): 1.68 (br-s, 12H), 1.87–2.03 (br-m, 12H), 2.12–2.25 (br-m, 6H), 7.77–7.83 (m, 2H), 7.99–8.04 (m, 2H), 8.18–8.23 (m, 1H).^{13}C NMR (99 MHz, $CDCl_3$, δ): 28.8 (d, *J* = 9.4 Hz, *C*H), 36.9 (s, *C*H$_2$), 39.2 (d, *J* = 22.6 Hz, *C*), 41.3 (d, *J* = 12.2 Hz, *C*H$_2$), 128.3 (*C*H), 129.5 (*C*H), 129.8 (*C*H), 131.2 (*C*H), 140.3 (*C*), 141.1 (*C*), 153.8 (d, *J* = 37.6 Hz, *C*), 159.5 (d, *J* = 33.8 Hz, *C*). ^{31}P NMR (160 MHz, $CDCl_3$, δ): 26.1. HRMS-EI (*m*/*z*): [M]$^+$ calcd for $C_{28}H_{34}N_2PCl$, 464.2148; found, 464.2146. mp 209 ºC.

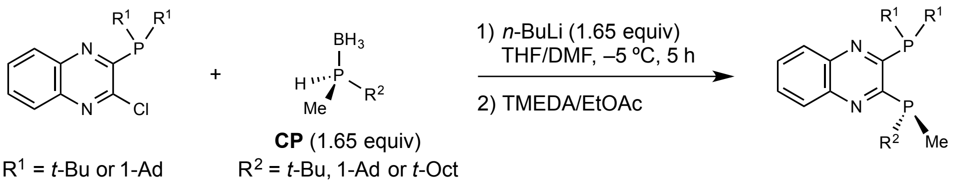

General procedure for the phosphination of 2-phosphanyl-3-chloroquinoxaline. The bisphosphine was synthesized according to the literature procedure[4]. A hexane solution of *n*-BuLi (1.55 M, 1.65 equiv) was added to a solution of the chiral phosphine–borane complex **CP** (>99% ee, 1.65 equiv) in dry THF (1.2 M) at 0 ºC under nitrogen atmosphere. The resulting solution was added to a suspension of the corresponding 2-phosphanyl-3-chloroquinoxaline in DMF (0.19 M) at –5 ºC. After stirring for 5 h, the reaction was quenched by H_2O and the mixture was extracted with EtOAc twice. The combined organic layer was washed with brine and dried over Na_2SO_4. After filtration, the solvents were removed by evaporation under reduced pressure. TMEDA/EtOAc (1:2) (0.07 M) was added to the residual oil at room temperature. After stirred for 2 h, the mixture was diluted with EtOAc and extracted with EtOAc. The organic layer was washed with water, aqueous HCl (6 M), water, and brine and was then dried over Na_2SO_4. After filtration, the solvents were removed under reduced pressure. The crude material was purified by flash column chromatography (SiO_2, Et_2O/hexane, 0:100–2:98) and reprecipitation or recrystallization to give the corresponding chiral bisphosphine as an orange or red solid.

2-[(1*S*)-(Adamantan-1-yl)(methyl)phosphanyl]-3-(di-*tert*-butylphosphanyl) quinoxaline [(*S*)-Quinox-Ad*t*Bu$_2$].

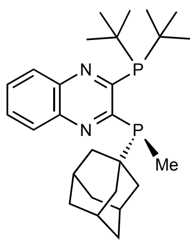

(*S*)-Quinox-Ad*t*Bu$_2$

(*S*)-Quinox-Ad*t*Bu$_2$ was prepared from the corresponding chiral phosphine–borane complex (**AP2,** >99% ee, 0.50 mmol, 97.1 mg)[5] and 2-phosphinyl-3-chloro quinoxaline (0.30 mmol, 92.6 mg)[4] according to the procedure described above. Recrystallization from hexane/MeOH (1:4) afforded red crystals (0.26 mmol, 119 mg, 87%).

^{1}H NMR (392 MHz, $CDCl_3$, δ): 1.17 (d, $J_{P–H}$ = 11.2 Hz, 9H), 1.37 (d, $J_{P–H}$ = 11.7 Hz, 9H), 1.38 (d, $J_{P–H}$ = 7.2 Hz, 3H), 1.59–1.72 (br-m, 6H), 1.73–1.83 (br-m, 3H), 1.87–1.97 (br-m, 6H), 7.69–7.76 (m, 2H), 8.06–8.12 (m, 2H).^{13}C NMR (99 MHz, $CDCl_3$, δ): 4.3 (dd, *J* = 8.5, 17.9 Hz, *C*H_3), 28.6 (d, *J* = 8.5 Hz, *C*H), 30.4 (dd, *J* = 2.4, 12.7 Hz, *C*H_3), 30.7 (d,

J = 14.1 Hz, *C*H3), 34.3 (dd, *J* = 5.4, 23.7 Hz, *C*), 35.0 (dd, *J* = 1.9, 15.5 Hz, *C*), 35.5 (dd, *J* = 1.9, 24.0 Hz, *C*), 37.0 (*C*H2), 38.9 (d, *J* = 10.8 Hz, *C*H2), 129.48 (*C*H), 129.50 (*C*H), 129.56 (*C*H), 129.64 (*C*H), 140.9 (*C*), 141.0 (*C*), 166.3 (t, *J* = 32.2 Hz, *C*), 166.7 (t, *J* = 31.0 Hz, *C*). ^{31}P NMR (160 MHz, $CDCl_3$, δ): –16.8 (d, *J* = 107.5 Hz), 21.4 (d, *J* = 107.5 Hz). HRMS-ESI (*m*/*z*): $[M+H]^+$ calcd for $C_{27}H_{41}N_2P_2$, 455.2740; found, 455.2737. $[\alpha]_D^{23}$ +151 (c 1.0, EtOAc). mp 116 ºC.

(*S*)-2-(Di-*tert*-butylphosphanyl)-3-[methyl(2,4,4-trimethylpentan-2-yl)phosphanyl] quinoxaline [(*S*)-Quinox-*t*Oct*t*Bu2].

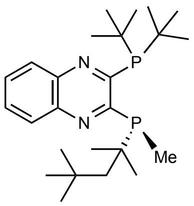

(*S*)-Quinox-*t*Oct*t*Bu2

(*S*)-Quinox-*t*Oct*t*Bu2 was prepared from the corresponding chiral phosphine–borane complex (**CP3**, >99% ee, 0.84 mmol, 146 mg)[6] and 2-phosphinyl-3-chloro quinoxaline (0.60 mmol, 185 mg)[4] according to the procedure described above. Recrystallization from hexane/MeOH (1:4) afforded red crystals (0.49 mmol, 211 mg, 81%).

^{1}H NMR (392 MHz, $CDCl_3$, δ): 1.00 (s, 9H), 1.20 (d, $J_{P–H}$ = 11.8 Hz, 9H), 1.30 (d, $J_{P–H}$ = 5.4 Hz, 3H), 1.33 (d, $J_{P–H}$ = 3.1 Hz, 3H), 1.35 (d, $J_{P–H}$ = 11.7 Hz, 9H), 1.41 (d, $J_{P–H}$ = 5.8 Hz, 3H), 1.52 (dd, $J_{P–H}$ = 7.2, 14.4 Hz, 1H), 1.82 (dd, $J_{P–H}$ = 9.0, 14.3 Hz, 1H), 7.69–7.75 (m, 2H), 8.05–8.10 (m, 2H).^{13}C NMR (99 MHz, $CDCl_3$, δ): 6.5 (dd, *J* = 8.2, 20.4 Hz, *C*H3), 25.0 (d, *J* = 12.2 Hz, *C*H3), 25.6 (d, *J* = 10.8 Hz, *C*H3), 30.5 (d, *J* = 10.8 Hz, *C*H3), 30.6 (d, *J* = 13.6 Hz, *C*H3), 33.6 (d, *J* = 11.3 Hz, *C*), 34.4 (dd, *J* = 4.7, 23.5 Hz, *C*), 35.2 (dd, *J* = 2.3, 23.5 Hz, *C*), 36.3 (dd, *J* = 3.5, 16.7 Hz, *C*), 51.1 (d. *J* = 17.4 Hz, *C*H2), 129.35 (*C*H), 129.44 (*C*H), 129.5 (*C*H), 129.7 (*C*H), 140.8 (*C*), 140.9 (*C*), 166.5 (t, *J* = 30.8 Hz, *C*), 167.6 (dd, *J* = 31.0, 33.4 Hz, *C*). ^{31}P NMR (160 MHz, $CDCl_3$, δ): –9.4 (d, *J* = 103.2 Hz), 21.3 (d, *J* = 103.2 Hz). HRMS-ESI (*m*/*z*): $[M+H]^+$ calcd for $C_{25}H_{43}N_2P_2$, 433.2896; found, 433.2893. $[\alpha]_D^{24}$ +84 (c 0.52, EtOAc). mp 121 ºC.

2-[(Diadamantan-1-yl)phosphanyl]-3-[(*S*)-*tert*-butyl(methyl)phosphanyl]quinoxaline [(*S*)-Quinox-*t*BuAd$_2$].

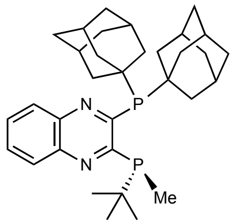

(*S*)-Quinox-*t*BuAd$_2$

(*R*)-Quinox-*t*BuAd$_2$ was prepared from the corresponding chiral phosphine–borane complex (**CP1**, >99% ee, 0.83 mmol, 97.3 mg)[7] and 2-phosphinyl-3-chloro quinoxaline (0.50 mmol, 233 mg)[4] according to the procedure described above. Recrystallization from hexane/MeOH (1:4) afforded red crystals (0.33 mmol, 174 mg, 65%).

^{1}H NMR (392 MHz, $CDCl_3$, δ): 1.16 (d, $J_{P–H}$ = 12.1 Hz, 9H), 1.42 (d, $J_{P–H}$ = 5.4 Hz, 3H), 1.59–1.77 (br-m, 12H), 1.82–1.97 (br-m, 9H), 1.99–2.10 (br-m, 6H), 2.27–2.36 (br-m, 3H), 7.72 (dd, *J* = 3.1, 6.7 Hz, 1H), 7.74 (dd, *J* = 2.9, 6.5 Hz, 1H), 8.05–8.15 (m, 2H). ^{13}C NMR (99 MHz, $CDCl_3$, δ): 6.8 (dd, *J* = 7.5, 18.8 Hz, *C*H_3), 27.7 (d, *J* = 14.1 Hz, *C*H_3), 28.9 (d, *J* = 7.5 Hz, *C*H), 29.0 (d, *J* = 7.5 Hz, *C*H), 31.2 (dd, *J* = 3.3, 14.6 Hz, *C*), 36.9 (*C*H_2), 37.1 (*C*H_2), 39.4 (dd, *J* = 4.7, 24.4 Hz, *C*), 40.1 (dd, *J* = 2.8, 24.4 Hz, *C*), 41.5 (d, *J* = 11.3 Hz, *C*H_2), 41.6 (d, *J* = 11.3 Hz, *C*H_2), 129.4 (*C*H), 129.6 (*C*H), 129.7 (*C*H), 140.7 (*C*), 141.0 (*C*), 164.8 (t, *J* = 30.5 Hz, *C*), 167.9 (t, *J* = 31.0 Hz, *C*). ^{31}P NMR (160 MHz, $CDCl_3$, δ): –14.9 (d, *J* = 107.5 Hz), 21.4 (d, *J* = 103.2 Hz). HRMS-ESI (*m*/*z*): $[M+H]^+$ calcd for $C_{33}H_{47}N_2P_2$, 533.3209; found, 533.3209. $[\alpha]_D^{24}$ +45 (c 0.48, $CHCl_3$). mp 203 ºC.

(*S*)-2-(Adamantan-1-yl(methyl)phosphanyl)-3-[(diadamantan-1-yl)phosphanyl] quinoxaline [(*S*)-Quinox-Ad$_3$].

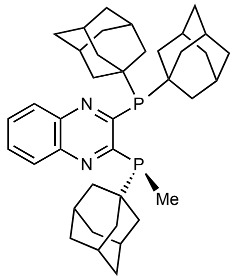

(*S*)-Quinox-Ad$_3$

(*S*)-Quinox-Ad$_3$ was prepared from the corresponding chiral phosphine–borane complex (**CP2**, >99% ee, 0.83 mmol, 162 mg)[5] and 2-phosphinyl-3-chloro quinoxaline (0.50 mmol, 233 mg)[4] according to the procedure described above. The product was

obtained as an orange solid (0.45 mmol, 273 mg, 90%). Recrystallization from CH_2Cl_2/MeOH (1:5) afforded red crystals to prepare crystals for single crystal XRD analysis.

^{1}H NMR (392 MHz, $CDCl_3$, δ): 1.37 (d, $J_{P\text{–}H}$ = 5.4 Hz, 3H), 1.57–2.10 (br-m, 42H), 2.30–2.39 (m, 3H), 7.73 (dd, *J* = 3.1, 6.7 Hz, 1H), 7.74 (dd, *J* = 3.4, 6.5 Hz, 1H), 8.07–8.15 (m, 2H).^{13}C NMR (99 MHz, $CDCl_3$, δ): 4.6 (dd, *J* = 8.5, 17.9 Hz, CH_3), 28.6 (d, *J* = 8.5 Hz, *C*H), 28.9 (d, *J* = 8.5 Hz, *C*H), 29.0 (d, *J* = 8.5 Hz, CH_3), 35.0 (dd, *J* = 2.3, 15.5 Hz, *C*), 36.9 (CH_2), 37.07 (CH_2), 37.14 (CH_2), 39.0 (d, *J* = 10.3 Hz, CH_2), 39.3 (dd, *J* = 5.2, 24.9 Hz, *C*), 40.4 (dd, *J* = 1.9, 24.4 Hz, *C*), 41.5 (d, *J* = 11.3 Hz, *C*), 41.6 (d. *J* = 8.5 Hz, CH_2), 129.4 (*C*H), 129.50 (*C*H), 129.52 (*C*H), 129.7 (*C*H), 140.6 (*C*), 140.9 (*C*). ^{31}P NMR (160 MHz, $CDCl_3$, δ): –16.8 (d, *J* = 103.2 Hz), 21.5 (d, *J* = 103.2 Hz). HRMS-ESI (*m*/*z*): $[M+H]^+$ calcd for $C_{39}H_{53}N_2P_2$, 611.3679; found, 611.3681. $[\alpha]_D^{24}$ +125 (c 0.52, $CHCl_3$). mp 268 ºC.

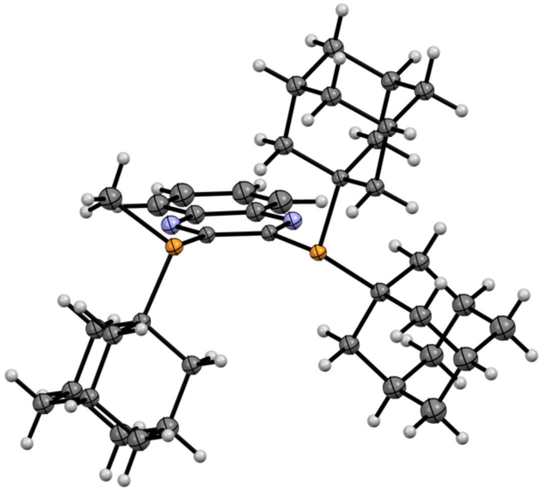

Supplementary Figure 2. ORTEP structure of (*S*)-Quinox-Ad3. Thermal ellipsoids are drawn at the 50% probability level (CCDC 1817859).

2-[(Diadamantan-1-yl)phosphanyl]-3-[(*S*)-methyl(2,4,4-trimethylpentan-2-yl) phosphanyl]quinoxaline [(*S*)-Quinox-*t*OctAd$_2$].

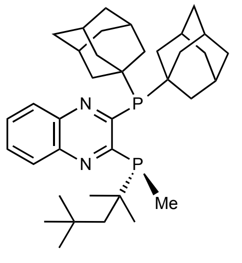

(*S*)-Quinox-*t*OctAd$_2$

(*S*)-Quinox-*t*OctAd$_2$ was prepared from the corresponding chiral phosphine–borane complex (**CP3**, >99% ee, 0.83 mmol, 144 mg)[6] and 2-phosphinyl-3-chloro quinoxaline (0.50 mmol, 233 mg)[4] according to the procedure described above. Reprecipitation from MeOH afforded orange powder (0.36 mmol, 215 mg, 73%).

^{1}H NMR (392 MHz, $CDCl_3$, δ): 0.99 (s, 9H), 1.30 (d, $J_{P–H}$ = 2.7 Hz, 3H), 1.33 (s, 3H), 1.40 (d, $J_{P–H}$ = 5.8 Hz, 3H), 1.48 (dd, $J_{P–H}$ = 7.4, 14.4 Hz, 1H), 1.58–1.75 (m, 12H), 1.82–1.96 (br-m, 10H), 2.00–2.10 (br-m, 6H), 2.28–2.36 (br-m, 3H), 7.72 (dd, *J* = 3.4, 6.5 Hz, 1H), 7.74 (dd, *J* = 3.4, 6.5 Hz, 1H), 8.05–8.15 (m, 2H). ^{13}C NMR (99 MHz, $CDCl_3$, δ): 6.7 (dd, *J* = 7.5, 20.7 Hz, *C*H$_3$), 25.1 (d, *J* = 14.1 Hz, *C*H$_3$), 25.4 (d, *J* = 9.4 Hz, *C*H$_3$), 28.9 (d, *J* = 4.7 Hz, *C*H), 29.0 (d, *J* = 5.6 Hz, *C*H), 32.3 (*C*H$_3$), 33.7 (d, *J* = 11.3 Hz, *C*), 36.5 (dd, *J* = 2.8, 16.9 Hz, *C*), 36.9 (*C*H$_2$), 37.1 (*C*H$_2$), 39.4 (dd, *J* = 4.7, 24.4 Hz, *C*), 40.1 (dd, *J* = 2.4, 24.9 Hz, *C*H$_2$), 41.5 (d, *J* = 11.3 Hz, *C*H$_2$), 41.6 (d, *J* = 9.4 Hz, *C*H$_2$), 51.2 (d, *J* = 17.9 Hz, *C*H$_2$), 129.4 (*C*H), 129.6 (*C*H), 129.7 (*C*H), 140.6 (*C*), 140.9 (*C*), 165.0 (t, *J* = 30.1 Hz, *C*), 168.1 (t, *J* = 32.4 Hz, *C*). ^{31}P NMR (160 MHz, $CDCl_3$, δ): –9.5 (d, *J* = 98.9 Hz), 21.6 (d, *J* = 103.2 Hz). HRMS-ESI (*m*/*z*): $[M+H]^+$ calcd for $C_{37}H_{55}N_2P_2$, 589.3835; found, 589.3837. $[\alpha]_D^{23}$ +126 (c 0.51, EtOAc). mp 214 ºC.

Supplementary Table 3. Summary of X-ray crystallographic data of (*S*)-Quinox-Ad_3.

CCDC Name	1817859
Empirical Formula	$C_{39}H_{52}N_2P_2$
Formula Weight	610.80
Crystal System	monoclinic
Crystal Size / mm	0.368 × 0.367 × 0.091
a / Å	23.233(4)
b / Å	12.269(2)
c / Å	22.880(4)
β / º	91.775(7)
V / Å3	6518.9(19)
Space Group	C_2 (#5)
Z value	8
Dcalc / g cm^{-3}	1.245
Temperature / K	123.15
$2\theta_{max}$ / °	55.0
μ (Mo-Kα) / cm^{-1}	1.644
No. of Reflections Measured	Total: 29639<br>Unique: 12918<br>(R_{int} = 0.0648)
No. of Observations (All reflections)	12918
Residuals: R_1 (I > 2.00σ (I))	0.0729
Residuals: wR_2-(All reflections)	0.2362
Goodness of Fit Indicator (GOF)	0.898
Maximum Peak in Final Diff. Map / Å^3	1.37 e-
Minimum Peak in Final Diff. Map / Å^3	-0.58 e-
Flack Parameter	0.09(10)

Substrate Preparation Procedures

1f and **1h** were prepared by the reaction between allylmagnesium bromide and the corresponding electrophile. **1l** was synthesized from 10-bromo-1-decene through azidation of bromide. **1k**, **1m** and **1n** were synthesized according to our previous publication[8]. Other terminal alkene substrates were purchased from suppliers. The purchased starting materials were used without further purification.

Preparation of allyldimethyl(phenyl)silane (1f)[9].

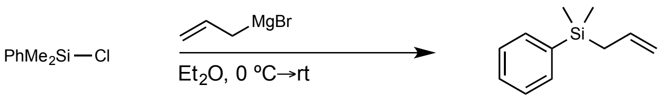

1f

In a vacuum dried round bottomed flask, dimethylphenylsilyl chloride (6.0 mmol, 1.0 mL) was added dropwise to a solution of allylmagnesium bromide in Et_2O (0.7 M, 6.0 mmol, 8.6 mL) at 0 ºC under nitrogen atmosphere. After stirring for 2 h at room temperature, the reaction mixture was quenched by addition of saturated aqueous NH_4Cl solution and extracted with Et_2O three times. The combined organic layer was dried over $MgSO_4$. After filtration, the solvent was removed under reduced pressure. The crude material was purified by column chromatography (SiO_2, hexane) to obtain the corresponding allylsilane (798 mg, 4.5 mmol, 75%) as a colorless oil.

^{1}H NMR (392 MHz, $CDCl_3$, δ): 0.28 (s, 6H), 1.76 (d, J = 8.1 Hz, 2H), 4.83–4.89 (m, 2H), 5.72–5.83 (m, 1H), 7.33–7.39 (m, 3H), 7.49–7.54 (m, 2H). ^{13}C NMR (99 MHz, $CDCl_3$, δ): –3.5 (CH$_3$), 23.7 (CH$_2$), 113.4 (CH$_2$), 127.7 (CH), 129.0 (CH), 133.6 (CH), 134.6 (CH), 138.6 (C). HRMS–EI (m/z): $[M]^+$ calcd for $C_{11}H_{16}Si$, 176.1021; found, 176.1022.

Preparation of 4-bromo-1-(but-3-en-1-yl)-2-fluorobenzene (1h).

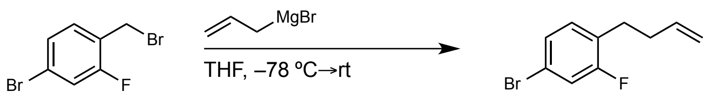

1h

In a vacuum dried round bottomed flask, a solution of allylmagnesium in Et_2O (0.7 M, 5.5 mmol, 7.9 mL) was added over 10 min to a solution of the corresponding benzyl bromide (5.0 mmol, 1.34 mg) in THF (12.5 mL) at –78 ºC under nitrogen atmosphere. After stirring for 1 h at –78 ºC, the mixture was then warmed to room temperature and stirring for 1 h. The reaction mixture was quenched by addition of water and extracted with Et_2O three times. The combined organic layer was dried over $MgSO_4$. After filtration, the

solvent was removed under reduced pressure. The crude material was purified by column chromatography (SiO_2, hexane) to obtain the corresponding terminal alkene (927 mg, 4.0 mmol, 81%) as a colorless oil.

^{1}H NMR (392 MHz, $CDCl_3$, δ): 2.34 (dt, *J* = 7.0, 15.0 Hz, 2H), 2.69 (t, *J* = 7.6 Hz, 2H), 4.97–5.05 (m, 2H), 5.82 (ddt, *J* = 6.6, 10.3, 17.1 Hz, 1H), 7.05 (t, *J* = 8.1 Hz, 1H), 7.17–7.21 (m, 2H). ^{13}C NMR (99 MHz, $CDCl_3$, δ): 28.1 (CH_2), 33.8 (CH_2), 115.5 (CH_2), 118.8 (d, *J* = 26.3 Hz, *C*H), 119.5 (d, *J* = 9.4 Hz, *C*), 127.1 (d, *J* = 2.8 Hz, *C*H), 127.7 (d, *J* = 16.0 Hz, *C*), 131.7 (d, *J* = 5.6 Hz, *C*H), 160.9 (d, *J* = 249.0 Hz, *C*). HRMS–EI (*m*/*z*): $[M]^+$ calcd for $C_{10}H_{10}BrF$, 227.9950; found, 227.9949.

Preparation of *tert*-butyl dec-9-en-1-ylcarbamate (1i)[10].

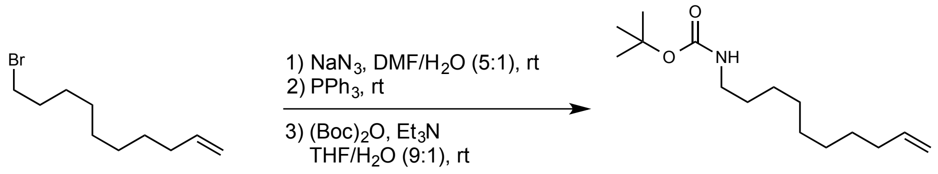

In a vacuum dried round bottomed flask, NaN_3 (11.7 mmol, 759 mg) was added portionwise to a solution of 10-bromo-1-dececne (4.7 mmol, 1.0 mL) in DMF/H_2O (9:1, 18.6 mL) at room temperature under nitrogen atmosphere. After stirring for 19 h, the reaction mixture was diluted with brine and extracted with Et_2O three times. The organic layer was dried over $MgSO_4$. After filtration, the solvents were removed under reduced pressure. In a vacuum dried round bottomed flask, the crude azide was dissolved to THF/H_2O (5:1, 16.4 mL). Then PPh_3 (5.6 mmol, 1.47 g) was added portionwise to the solution at room temperature under nitrogen atmosphere. After stirred for 17 h, triethylamine (18.8 mmol, 2.62 mL) and Boc_2O (10.3 mmol, 2.26 mL) were subsequently added to the mixture. After 11 h, the solvents were removed from the reaction mixture under reduced pressure. The residue was diluted with brine and extracted with Et_2O three times. The combined organic layer was dried over $MgSO_4$. After filtration, the solvent was removed under reduced pressure. The crude material was purified by column chromatography (SiO_2, EtOAc/hexane, 0:100–20:80) to obtained the corresponding Boc-protected amine **1i** (2.4 mmol, 617 mg) as a colorless oil.

^{1}H NMR (392 MHz, $CDCl_3$, δ): 1.23–1.52 (m, 21H), 2.04 (dt, *J* = 6.7, 14.8 Hz, 2H), 3.02–3.15 (m, 2H), 4.49 (br-s, N*H*, 1H), 4.91–5.02 (m, 2H), 5.81 (ddt, *J* = 6.7, 10.3, 17.1 Hz, 1 H). ^{13}C NMR (99 MHz, $CDCl_3$, δ): 26.7 (CH_2), 28.3 (CH_3), 28.8 (CH_2), 29.0 (CH_2), 29.2 (CH_2), 29.3 (CH_2), 30.0 (CH_2), 33.7 (CH_2), 40.5 (CH_2), 78.8 (*C*), 114.1 (CH_2), 139.1 (*C*H), 155.9 (*C*). HRMS–ESI (*m*/*z*): $[M+Na]^+$ calcd for $C_{15}H_{29}NO_2Na$, 278.2091; found, 278.2093.

Borylation Product Characterization

(*S*)-4,4,5,5-Tetramethyl-2-(4-phenylbutan-2-yl)-1,3,2-dioxaborolane [(*S*)-3a][11,12].

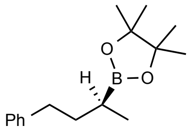

(*S*)-**3a**

According to the general borylation procedure, the reaction was conducted with 3 mol % $[Cu(MeCN)_4]BF_4$/(*S*)-Quinox-*t*OctAd$_2$ and 66.1 mg (0.50 mmol) of **1a**. The product (*S*)-**3a** was obtained in 94% yield (122 mg, 0.47 mmol, colorless oil) with 99% ee, **3**/**4** = 92:8. The regioselectivity was determined by GC analysis using the crude material. The absolute configuration of (*S*)-**3a** was confirmed using the optical rotation value of the previous reports[11,12].

^{1}H NMR (392 MHz, $CDCl_3$, δ): 0.99–1.13 (m, 4H), 1.25 (s, 12H), 1.54–1.67 (m, 1H), 1.74–1.83 (m, 1H), 2.56–2.68 (m, 2H), 7.13–7.22 (m, 3H), 7.23–7.30 (m, 2H). ^{13}C NMR (99 MHz, $CDCl_3$, δ): 15.4 (CH_3), 16.7 (br, B–*C*H), 24.7 (CH_3), 24.8 (CH_3), 35.3 (CH_2), 82.8 (*C*), 125.5 (*C*H), 128.1 (*C*H), 128.4 (*C*H), 143.0 (*C*). HRMS–EI (*m*/*z*): $[M]^+$ calcd for $C_{16}H_{25}BO_2$, 260.1951; found, 260.1947. $[\alpha]_D^{24}$ +5.2 (c 1.1, $CHCl_3$, 99% ee). The ee value was determined by HPLC analysis of the corresponding alcohol after $NaBO_3$•$4H_2O$ oxidation of (*S*)-**3a** in comparison of the racemic sample. Daicel CHIRALPAK® OZ-3, 2-PrOH/hexane = 2/98, 0.25 mL/min, 40 ºC, *S* isomer: t_R = 38.5 min., *R* isomer: t_R = 43.8 min.

(*S*)-4,4,5,5-Tetramethyl-2-(octan-2-yl)-1,3,2-dioxaborolane [(*S*)-3b][13].

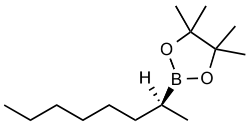

(*S*)-**3b**

According to the general borylation procedure, the reaction was conducted with 3 mol % $[Cu(MeCN)_4]BF_4$/(*S*)-Quinox-*t*OctAd$_2$ and 56.1 mg (0.50 mmol) of **1b**. The product (*S*)-**3b** was obtained in 76% yield (91.5 mg, 0.38 mmol, colorless oil) with 98% ee, **3**/**4** = 88:12. The regioselectivity was determined by GC analysis using the crude material. The

regioisomer **4b** was removed by flash column chromatography (SiO_2, CH_2Cl_2/hexane, 0:100–32:68).

^{1}H NMR (392 MHz, $CDCl_3$, δ): 0.87 (t, *J* = 7.0 Hz, 3H), 0.92–1.07 (m, 4H), 1.20–1.35 (m, 9H), 1.24 (s, 12H), 1.37–1.49 (m, 1H). ^{13}C NMR (99 MHz, $CDCl_3$, δ): 14.1 (CH_3), 15.5 (CH_3), 16.8 (br, B–*C*H), 22.6 (CH_2), 24.69 (CH_3), 24.73 (CH_3), 28.9 (CH_2), 29.5 (CH_2), 31.9 (CH_2), 33.2 (CH_2), 82.7 (*C*). HRMS–EI (*m*/*z*): [M]$^+$ calcd for $C_{14}H_{29}BO_2$, 240.2263; found, 240.2267. $[\alpha]_D^{23}$ +4.8 (c 1.0, $CHCl_3$, 98% ee). The ee value was determined by HPLC analysis of the corresponding ester after $NaBO_3$•$4H_2O$ oxidation and esterification with *p*-bromobenzoyl chloride of (*S*)-**3b** in comparison of the racemic sample. Daicel CHIRALPAK® OZ-3, 2-PrOH/hexane = 0.1/99.9, 0.50 mL/min, 40 ºC, *R* isomer: t_R = 20.3 min., *S* isomer: t_R = 21.7 min.

(*S*)-2-(Decan-2-yl)-4,4,5,5-tetramethyl-1,3,2-dioxaborolane [(*S*)-3c][11].

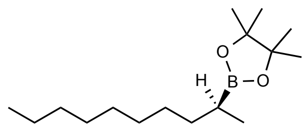

(*S*)-**3c**

According to the general borylation procedure, the reaction was conducted with 3 mol % $[Cu(MeCN)_4]BF_4$/(*S*)-Quinox-*t*OctAd$_2$ and 70.1 mg (0.50 mmol) of **1c**. The product (*S*)-**3c** was obtained in 61% yield (81.6 mg, 0.31 mmol, colorless oil) with 99% ee, **3**/**4** = 89:11. The regioselectivity was determined by GC analysis using the crude material. The regioisomer **4c** was removed by flash column chromatography (SiO_2, CH_2Cl_2/hexane, 0:100–32:68).

Experiment in 5.0 mmol scale: According to the general borylation procedure, the reaction was conducted with 701.4 mg (5.0 mmol) of **1c**. The product (*S*)-**3c** was obtained in 71% yield (946 mg, 3.5 mmol, colorless oil) with 98% ee, **3**/**4** = 88:12. The regioselectivity was determined by GC analysis using the crude material. The regioisomer **4c** was removed by flash column chromatography (SiO_2, CH_2Cl_2/hexane, 0:100–32:68). The absolute configuration of (*S*)-**3c** was confirmed using the value of optical rotation of the previous report[11].

^{1}H NMR (392 MHz, $CDCl_3$, δ): 0.87 (t, *J* = 7.0 Hz, 3H), 0.92–1.07 (m, 4H), 1.19–1.35 (m, 13H), 1.24 (s, 12H), 1.36–1.49 (m, 1H). ^{13}C NMR (99 MHz, $CDCl_3$, δ): 14.1 (CH_3), 15.5 (CH_3), 16.9 (br, B–*C*H), 22.7 (CH_2), 24.68 (CH_3), 24.72 (CH_3), 29.0 (CH_2), 29.3 (CH_2), 29.6 (CH_2), 29.8 (CH_2), 31.9 (CH_2), 33.2 (CH_2), 82.7 (*C*). HRMS–EI (*m*/*z*): [M]$^+$ calcd for $C_{16}H_{33}O_2B$, 268.2577; found, 268.2581. $[\alpha]_D^{22}$ +7.7 (c 1.0, $CHCl_3$, 99% ee). The ee value was determined by HPLC analysis of the corresponding ester after

$NaBO_3 \cdot 4H_2O$ oxidation and esterification with *p*-nitrobenzoyl chloride of (*S*)-**3c** in comparison of the racemic sample. Daicel CHIRALPAK® IC-3, 2-PrOH/hexane = 0.5/99.5, 0.50 mL/min, 40 ºC, *R* isomer: t_R = 33.1 min., *S* isomer: t_R = 35.7 min.

(*S*)-2-(Dodecan-2-yl)-4,4,5,5-tetramethyl-1,3,2-dioxaborolane [(*S*)-3d][12].

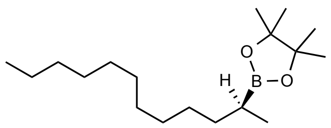

(*S*)-**3d**

According to the general borylation procedure, the reaction was conducted with 3 mol % $[Cu(MeCN)_4]BF_4$/(*S*)-Quinox-*t*OctAd$_2$ and 84.2 mg (0.500 mmol) of **1d**. The product (*S*)-**3d** was obtained in 57% yield (113 mg, 0.27 mmol, colorless oil) with 97% ee, **3**/**4** = 88:12. The regioselectivity was determined by GC analysis using the crude material. The regioisomer **4d** was removed by flash column chromatography (SiO_2, CH_2Cl_2/hexane, 0:100–32:68). The absolute configuration of (*S*)-**3d** was confirmed using the value of optical rotation of the previous report[12].

^1H NMR (392 MHz, $CDCl_3$, δ): 0.88 (t, *J* = 6.7 Hz, 3H), 0.92–1.07 (m, 4H), 1.20–1.35 (m, 17H), 1.24 (s, 12H), 1.36–1.49 (m, 1H). ^{13}C NMR (99 MHz, $CDCl_3$, δ): 14.1 (*C*H_3), 15.5 (*C*H_3), 17.0 (br, B–*C*H), 22.7 (*C*H_2), 24.70 (*C*H_3), 24.73 (*C*H_3), 29.0 (*C*H_2), 29.4 (*C*H_2), 29.6 (*C*H_2), 29.9 (*C*H_2), 31.9 (*C*H_2), 33.2 (*C*H_2), 82.7 (*C*). HRMS–EI (*m*/*z*): $[M]^+$ calcd for $C_{18}H_{37}BO_2$, 296.2890; found, 296.2889. $[\alpha]_D^{23}$ +3.0 (c 1.0, $CHCl_3$, 97% ee). The ee value was determined by HPLC analysis of the corresponding ester after $NaBO_3 \cdot 4H_2O$ oxidation and esterification with *p*-bromobenzoyl chloride of (*S*)-**3d** in comparison of the racemic sample. Daicel CHIRALPAK® OZ-3, 2-PrOH/hexane = 0.1/99.9, 0.50 mL/min, 40 ºC, *R* isomer: t_R = 18.6 min., *S* isomer: t_R = 20.0 min.

(*S*)-4,4,5,5-Tetramethyl-2-(1-phenylpropan-2-yl)-1,3,2-dioxaborolane [(*S*)-3e][14,15].

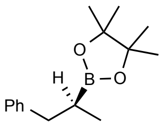

(*S*)-**3e**

According to the general borylation procedure, the reaction was conducted with 5 mol % $[Cu(MeCN)_4]BF_4$/(*S*)-Quinox-*t*OctAd$_2$ and 59.1 mg (0.500 mmol) of **1e**. The

product (*S*)-**3e** was obtained in 88% yield (109 mg, 0.44 mmol, colorless oil) with 99% ee, **3**/**4** = 85:15. The regioselectivity was determined by GC analysis using the crude material.

(*S*)-**3e** (*branched*)[14]: ^{1}H NMR (392 MHz, $CDCl_3$, δ): 0.96 (d, *J* = 7.6 Hz, 3H), 1.18 (s, 6H), 1.19 (s, 6H), 1.32–1.42 (m, 1H), 2.54 (dd, *J* = 8.5, 13.5 Hz, 1H), 2.81 (dd, *J* = 7.4, 13.7 Hz, 1H), 7.13–7.28 (m, 5H). ^{13}C NMR (99 MHz, $CDCl_3$, δ): 15.1 (CH_3), 24.7 (CH_3), 38.9 (CH_2), 82.9 (*C*), 125.5 (*C*H), 128.0 (*C*H), 128.9 (*C*H), 142.3 (*C*). **4e** (*linear*)[15]: ^{1}H NMR (392 MHz, $CDCl_3$, δ): 0.83 (t, *J* = 7.9 Hz, 2H), 1.24 (s, 12H), 1.73 (dt, *J* = 7.9, 15.7 Hz, 2H), 2.61 (t, *J* = 7.9 Hz, 2H), 7.13–7.28 (m, 5H). ^{13}C NMR (99 MHz, $CDCl_3$, δ): 24.8 (CH_3), 26.1 (CH_2), 38.6 (CH_2), 82.9 (*C*), 125.5 (*C*H), 128.1 (*C*H), 128.5 (*C*H), 142.7(*C*). The carbon directly attached to the boron atom was not detected, likely due to quadrupolar relaxation. HRMS–EI (*m*/*z*): [M]$^+$ calcd for $C_{15}H_{23}BO_2$, 246.1794; found, 246.1794. $[\alpha]_D^{26}$ +3.7 (c 1.0, $CHCl_3$, 99% ee). The ee value was determined by HPLC analysis of the corresponding alcohol after $NaBO_3$•$4H_2O$ oxidation of (*S*)-**3e** in comparison of the racemic sample. Daicel CHIRALPAK® OD-3, 2-PrOH/hexane = 5/95, 0.50 mL/min, 40 ºC, *S* isomer: t_R = 12.8 min., *R* isomer: t_R = 13.9 min.

(*S*)-Dimethyl(phenyl)[2-(4,4,5,5-tetramethyl-1,3,2-dioxaborolan-2-yl)propyl]silane [(*S*)-3f][16].

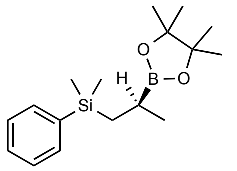

(*S*)-**3f**

According to the general borylation procedure, the reaction was conducted with 5 mol % $[Cu(MeCN)_4]BF_4$/(*S*)-Quinox-*t*OctAd$_2$ and 88.2 mg (0.500 mmol) of **1f** at –20 ºC. The product (*S*)-**3f** was obtained in 60% yield (91.6 mg, 0.30 mmol, colorless oil) with 98% ee, **3**/**4** = 93:7. The regioselectivity was determined by GC analysis using the crude material. The absolute configuration of (*S*)-**3f** was confirmed using the optical rotation value of the previous report[16].

^{1}H NMR (392 MHz, $CDCl_3$, δ): 0.27 (s, 6H), 0.66–0.71 (m, 1H), 0.99 (d, *J* = 7.2 Hz, 3H), 1.03–1.16 (m, 2H), 1.20 (s, 12H), 7.31–7.35 (m, 3H), 7.51–7.53 (m, 2H). ^{13}C NMR (99 MHz, $CDCl_3$, δ): –2.30 (CH_3), –2.25 (CH_3), 11.9 (br, B–*C*H), 18.8 (CH_2), 19.5 (*C*H), 24.69 (CH_3), 24.73 (CH_3), 82.8 (*C*), 127.6 (*C*H), 128.6 (*C*H), 133.6 (*C*H), 140.1 (*C*). HRMS-ESI (*m*/*z*): [M+Na]$^+$ calcd for $C_{17}H_{29}O_2BSiNa$, 327.1922; found, 327.1926. $[\alpha]_D^{26}$ +5.0 (c 1.0, $CHCl_3$, 98% ee). The ee value was determined by HPLC analysis of the

corresponding alcohol after $NaBO_3$•$4H_2O$ oxidation of (*S*)-**3f** in comparison of the racemic sample. Daicel CHIRALPAK® OZ-3, 2-PrOH/hexane = 0.5/99.5, 0.50 mL/min, 40 ºC, *R* isomer: t_R = 35.4 min., *S* isomer: t_R = 36.5 min.

(*S*)-2-(6-Chlorohexan-2-yl)-4,4,5,5-tetramethyl-1,3,2-dioxaborolane [(*S*)-3g][11,17].

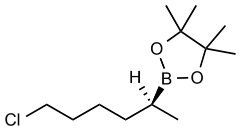

(*S*)-**3g**

According to the general borylation procedure, the reaction was conducted with 5 mol % $[Cu(MeCN)_4]BF_4$/(*S*)-Quinox-*t*OctAd$_2$ and 59.3 mg (0.50 mmol) of **1g**. The product (*S*)-**3g** was obtained in 89% yield (110 mg, 0.45 mmol, colorless oil) with 98% ee, **3**/**4** = 86:14. The regioselectivity was determined by GC analysis using the crude material. The absolute configuration of (*S*)-**3g** was confirmed using the optical rotation value of the previous report[11].

(*S*)-**3g** (*branched*)[11]: ^{1}H NMR (392 MHz, $CDCl_3$, δ): 0.96–1.08 (m, 4H), 1.20–1.36 (m, 1H), 1.24 (s, 12 H), 1.39–1.52 (m, 3H), 1.76 (dt, *J* = 7.1 Hz, 14.2 Hz, 2H), 3.53 (t, *J* = 7.0 Hz, 2H). ^{13}C NMR (99 MHz, $CDCl_3$, δ): 15.4 (CH_3), 16.9 (br, B–*C*H), 24.67 (CH_3), 24.72 (CH_3), 26.1 (CH_2), 32.3 (CH_2), 32.8 (CH_2), 45.1 (CH_2), 82.8 (*C*). **4g** (*linear*)[17]: ^{1}H NMR (392 MHz, $CDCl_3$, δ): 0.78 (t, *J* = 7.2 Hz, 2H), 1.20–1.36 (m, 2H), 1.25 (s, 12 H), 1.39–1.52 (m, 4H), 1.76 (dt, *J* = 7.1 Hz, 14.2 Hz, 2H), 3.52 (t, *J* = 6.7 Hz, 2H). ^{13}C NMR (99 MHz, $CDCl_3$, δ): 16.9 (br, B–CH_2), 23.7 (CH_2), 24.8 (CH_3), 26.6 (CH_2), 31.5 (CH_2), 32.5 (CH_2), 45.1 (CH_2), 82.8 (*C*). HRMS–EI (*m*/*z*): $[M]^+$ calcd for $C_{12}H_{24}BClO_2$, 246.1560; found, 246.1562. $[\alpha]_D^{22}$ +4.6 (c 1.0, $CHCl_3$, 98% ee). The ee value was determined by HPLC analysis of the corresponding ester after $NaBO_3$•$4H_2O$ oxidation and esterification with benzoyl chloride of (*S*)-**3g** in comparison of the racemic sample. Daicel CHIRALPAK® AD-3, 2-PrOH/hexane = 1/99, 0.50 mL/min, 40 ºC, *R* isomer: t_R = 13.1 min., *S* isomer: t_R = 14.7 min.

(*S*)-2-[4-(4-Bromo-2-fluorophenyl)butan-2-yl]-4,4,5,5-tetramethyl-1,3,2-dioxaborolane [(*S*)-3h].

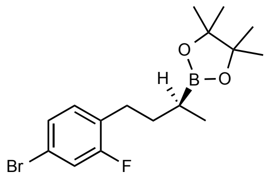

(*S*)-**3h**

According to the general borylation procedure, the reaction was conducted with 3 mol % $[Cu(MeCN)_4]BF_4$/(*S*)-Quinox-*t*OctAd$_2$ and 115 mg (0.50 mmol) of **1h**. The product (*S*)-**3h** was obtained in 73% yield (131 mg, 0.37 mmol, colorless oil) with 99% ee, **3**/**4** = 84:16. The regioisomer **4h** was removed by flash column chromatography (SiO_2, Et_2O/hexane, 0:100–4:96). The regioselectivity was determined by GC analysis using the crude material.

^{1}H NMR (392 MHz, $CDCl_3$, δ): 1.01–1.09 (m, 4H), 1.25 (s, 12H), 1.49–1.58 (m, 1H), 1.69–1.78 (m, 1H), 2.54–2.66 (m, 2H), 7.06 (t, *J* = 8.1 Hz, 1H), 7.15–7.19 (m, 2H). ^{13}C NMR (99 MHz, $CDCl_3$, δ): 15.3 (CH_3), 16.7 (br, B–*C*H), 24.67 (CH_3), 24.73 (CH_3), 27.8 (CH_2), 33.5 (CH_2), 82.9 (*C*), 118.7 (d, *J* = 25.4 Hz, *C*H), 119.1 (d, *J* = 9.4 Hz, *C*), 127.0 (d, *J* = 3.8 Hz, *C*H), 128.9 (d, *J* = 16.0 Hz, *C*), 131.7 (d, *J* = 5.6 Hz, *C*H), 160.8 (d, *J* = 249.0 Hz, *C*). HRMS–EI (*m*/*z*): $[M]^+$ calcd for $C_{16}H_{23}BBrFO_2$, 356.0962; found, 356.0961. $[\alpha]_D^{26}$ +8.2 (c 1.0, $CHCl_3$, 99% ee). The ee value was determined by HPLC analysis of the corresponding alcohol after $NaBO_3$•$4H_2O$ oxidation of (*S*)-**3h** in comparison of the racemic sample. Daicel CHIRALPAK® OD-3, 2-PrOH/hexane = 1/99, 0.50 mL/min, 40 °C, *S* isomer: t_R = 42.6 min., *R* isomer: t_R = 44.6 min.

***tert*-Butyl (*S*)-(9-(4,4,5,5-tetramethyl-1,3,2-dioxaborolan-2-yl)decyl)carbamate [(*S*)-3i].**

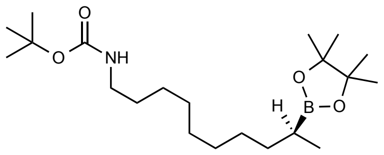

(*S*)-**3i**

According to the general borylation procedure, the reaction was conducted with 5 mol % $[Cu(MeCN)_4]BF_4$/(*S*)-Quinox-*t*OctAd$_2$ and 128 mg (0.50 mmol) of **1i** at –10 °C. The product (*S*)-**3i** was obtained in 93% yield (178.5 mg, 0.47 mmol, colorless oil) with

97% ee, **3**/**4** = 87:13. The regioselectivity was determined by GC analysis using the crude material.

^{1}H NMR (392 MHz, $CDCl_3$, δ):0.92–1.07 (m, 4H), 1.19–1.36 (m, 12H), 1.24 (s, 12H), 1.38–1.52 (m, 11H), 3.02–3.15 (m, 2H), 4.49 (br-s, N*H*, 1H). ^{13}C NMR (99 MHz, $CDCl_3$, δ): 15.5 (CH_3), 17.0 (br, B–*C*H), 24.66 (CH_3), 24.69 (CH_3), 26.8 (CH_2), 28.4 (CH_3), 28.9 (CH_2), 29.2 (CH_2), 29.5 (CH_2), 29.7 (CH_2), 30.0 (CH_2), 33.1 (CH_2), 40.6 (CH_2), 78.9 (*C*), 82.7 (*C*), 155.9 (*C*). HRMS–ESI (*m*/*z*): $[M+Na]^+$ calcd for $C_{21}H_{42}BO_4NNa$, 406.3103; found, 406.3101. $[\alpha]_D^{22}$ +3.50 (c 1.0, $CHCl_3$, 97% ee). The ee value was determined by HPLC analysis of the corresponding ester after $NaBO_3$•$4H_2O$ oxidation and esterification with *p*-nitrobenzoyl chloride of (*S*)-**3i** in comparison of the racemic sample. Daicel CHIRALPAK® AD-3, 2-PrOH/hexane = 1/99, 0.50 mL/min, 40 ºC, *S* isomer: t_R = 48.3 min., *R* isomer: t_R = 51.5 min.

Ethyl (*S*)-10-(4,4,5,5-tetramethyl-1,3,2-dioxaborolan-2-yl)undecanoate [(*S*)-3j].

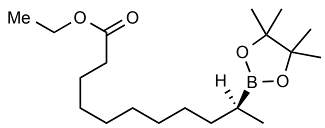

(*S*)-**3j**

According to the general borylation procedure, the reaction was conducted with 5 mol % $[Cu(MeCN)_4]BF_4$/(*S*)-Quinox-*t*OctAd$_2$ and 106 mg (0.50 mmol) of **1j**. The product (*S*)-**3j** was obtained in 91% yield (155 mg, 0.46 mmol, colorless oil) with 98% ee, **3**/**4** = 90:10. The regioselectivity was determined by GC analysis using the crude material.

^{1}H NMR (392 MHz, $CDCl_3$, δ): 0.95–1.09 (m, 4H), 1.24 (s, 12H), 1.24–1.43 (m, 15H), 1.57–1.63 (m, 2H), 2.28 (t, *J* = 7.6 Hz, 2H), 4.12 (q, *J* = 7.2 Hz, 2H). ^{13}C NMR (99 MHz, $CDCl_3$, δ): 14.2 (CH_3), 15.5 (CH_3), 17.0 (br, B–*C*H), 24.6 (CH_3), 24.7 (CH_3), 24.9 (CH_2), 28.9 (CH_2), 29.1 (CH_2), 29.2 (CH_2), 29.4 (CH_2), 29.7 (CH_2), 33.2 (CH_2), 34.3 (CH_2), 60.1 (CH_2), 82.7 (*C*), 173.9 (*C*). HRMS–EI (*m*/*z*): [M]+ calcd for $C_{19}H_{37}BO_4$, 340.2788; found, 340.2788. $[\alpha]_D^{22}$ +6.8 (c 1.0, $CHCl_3$, 98% ee). The ee value was determined by HPLC analysis of the corresponding ester after $NaBO_3$•$4H_2O$ oxidation and esterification with *p*-nitrobenzoyl chloride of (*S*)-**3j** in comparison of the racemic sample. Daicel CHIRALPAK® OJ-3, 2-PrOH/hexane = 2/98, 0.50 mL/min, 40 ºC, *S* isomer: t_R = 26.7 min., *R* isomer: t_R = 46.0 min.

(*S*)-9-(4,4,5,5-Tetramethyl-1,3,2-dioxaborolan-2-yl)decyl benzoate [(*S*)-3k][8].

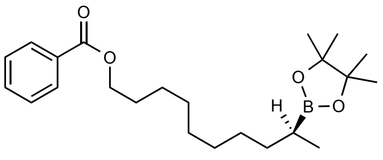

(*S*)-**3k**

According to the general borylation procedure, the reaction was conducted with 5 mol % [Cu(MeCN)$_4$]BF$_4$/(*S*)-Quinox-*t*OctAd$_2$ and 130 mg (0.50 mmol) of **1k**. The product (*S*)-**3k** was obtained in 85% yield (166 mg, 0.43 mmol, colorless oil) with 96% ee, **3**/**4** = 90:10. The regioselectivity was determined by GC analysis using the crude material.

^{1}H NMR (392 MHz, CDCl$_3$, δ): 0.92–1.07 (m, 4H), 1.23 (s, 12H), 1.19–1.50 (m, 12H), 1.76 (dt, *J* = 7.1 Hz, 14.4 Hz, 2H), 4.31 (t, *J* = 6.7 Hz, 2H), 7.41–7.48 (m, 2H), 7.52–7.58 (m, 1H), 8.02–8.08 (m, 2H). ^{13}C NMR (99 MHz, CDCl$_3$, δ): 15.5 (*C*H$_3$), 17.0 (br, B–*C*H), 24.66 (*C*H$_3$), 24.70 (*C*H$_3$), 26.0 (*C*H$_2$), 28.7 (*C*H$_2$), 28.9 (*C*H$_2$), 29.2 (*C*H$_2$), 29.4 (*C*H$_2$), 29.7 (*C*H$_2$), 33.2 (*C*H$_2$), 65.1 (*C*H$_2$), 82.7 (*C*), 128.3 (*C*H), 129.5 (*C*H), 130.5 (*C*), 132.7 (*C*H), 166.6 (*C*). HRMS–EI (*m*/*z*): [M]$^+$ calcd for, C$_{23}$H$_{37}$BO$_4$, 388.2789; found, 388.2804. $[\alpha]_D^{27}$ +2.4 (c 1.0, CHCl$_3$, 96% ee). The ee value was determined by HPLC analysis of the corresponding ester after NaBO$_3$•4H$_2$O oxidation and esterification with *p*-nitrobenzoyl chloride of (*S*)-**3k** in comparison of the racemic sample. Daicel CHIRALPAK® OD-3, 2-PrOH/hexane = 1/99, 0.50 mL/min, 40 ºC, *R* isomer: t_R = 43.4 min., *S* isomer: t_R = 46.8 min.

(*S*)-7-(4,4,5,5-Tetramethyl-1,3,2-dioxaborolan-2-yl)octyl acetate [(*S*)-3l].

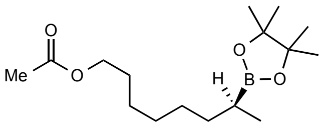

(*S*)-**3l**

According to the general borylation procedure, the reaction was conducted with 5 mol % [Cu(MeCN)$_4$]BF$_4$/(*S*)-Quinox-*t*OctAd$_2$ and 85.1 mg (0.50 mmol) of **1l**. The product (*S*)-**3l** was obtained in 80% yield (120 mg, 0.40 mmol, colorless oil) with 96% ee, **3**/**4** = 89:11. The regioselectivity was determined by GC analysis using the crude material.

^{1}H NMR (392 MHz, CDCl$_3$, δ): 0.93–1.07 (m, 4H), 1.20–1.49 (m, 8H), 1.24 (s, 12H), 1.55–1.66 (m, 2H), 2.04 (s, 3H), 4.04 (t, *J* = 6.7 Hz, 2H). ^{13}C NMR (99 MHz, CDCl$_3$, δ): 15.4 (*C*H$_3$), 17.0 (br-s, B–*C*H), 20.9 (*C*H$_3$), 24.6 (*C*H$_3$), 24.7 (*C*H$_3$), 25.8 (*C*H$_2$), 28.5 (*C*H$_2$),

28.7 (CH_2), 29.4 (CH_2), 33.0 (CH_2), 64.6 (CH_2), 82.7 (*C*), 171.1 (*C*). HRMS–ESI (*m*/*z*): $[M+Na]^+$ calcd for $C_{16}H_{31}BO_4Na$, 321.2208; found, 321.2208. $[\alpha]_D^{27}$ +4.0 (c 1.0, $CHCl_3$, 96% ee). The ee value was determined by HPLC analysis of the corresponding ester after $NaBO_3$•$4H_2O$ oxidation and esterification with *p*-nitrobenzoyl chloride of (*S*)-**3l** in comparison of the racemic sample. Daicel CHIRALPAK® AD-3, 2-PrOH/hexane = 2/98, 0.50 mL/min, 40 ºC, *R* isomer: t_R = 43.8 min., *S* isomer: t_R = 45.6 min.

(*S*)-4,4,5,5-Tetramethyl-2-(10-((3-methylbut-2-en-1-yl)oxy)decan-2-yl)-1,3,2-dioxaborolane [(*S*)-3m][8].

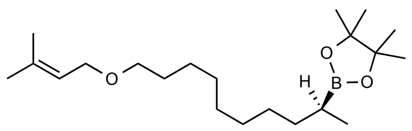

(*S*)-**3m**

According to the general borylation procedure, the reaction was conducted with 5 mol % $[Cu(MeCN)_4]BF_4$/(*S*)-Quinox-*t*OctAd$_2$ and 112 mg (0.50 mmol) of **1m**. The product (*S*)-**3m** was obtained in 71% yield (125 mg, 0.35 mmol, colorless oil) with 95% ee, **3**/**4** = 88:12. The regioselectivity was determined by GC analysis using the crude material.

^{1}H NMR (392 MHz, $CDCl_3$, δ): 0.92–1.07 (m, 4H), 1.24 (s, 12H), 1.20–1.47 (m, 12H), 1.52–1.62 (m, 2H), 1.67 (s, 3H), 1.74 (s, 3H), 3.39 (t, *J* = 6.7 Hz, 2H), 3.93 (d, *J* = 7.2 Hz, 2H), 5.33–5.38 (m, 1H). ^{13}C NMR (99 MHz, $CDCl_3$, δ): 15.5 (CH_3), 17.0 (br, B–*C*H), 17.9 (CH_3), 24.6 (CH_3), 24.7 (CH_3), 25.7 (CH_3), 26.1 (CH_2), 28.9 (CH_2), 29.4 (CH_2), 29.5 (CH_2), 29.7 (CH_2), 29.8 (CH_2), 33.2 (CH_2), 67.1 (CH_2), 70.3 (CH_2), 82.7 (*C*), 121.3 (*C*H), 136.5 (*C*). HRMS–EI (*m*/*z*): $[M]^+$ calcd for, $C_{21}H_{41}BO_3$, 352.3153; found, 352.3158. $[\alpha]_D^{27}$ +4.0 (c 1.0, $CHCl_3$, 95% ee). The ee value was determined by HPLC analysis of the corresponding ester after $NaBO_3$•$4H_2O$ oxidation and esterification with *p*-bromobenzoyl chloride of (*S*)-**3m** in comparison of the racemic sample. Daicel CHIRALPAK® OD-3, 2-PrOH/hexane = 0.5/99.5, 0.50 mL/min, 40 ºC, *R* isomer: t_R = 14.7 min., *S* isomer: t_R = 29.1 min.

(*S*)-Methyldiphenyl{[4-(4,4,5,5-tetramethyl-1,3,2-dioxaborolan-2-yl)pentyl]oxy} silane [(*S*)-3n][8].

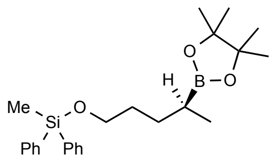

(*S*)-**3n**

According to the general borylation procedure, the reaction was conducted with 5 mol % [Cu(MeCN)$_4$]BF$_4$/(*S*)-Quinox-*t*OctAd$_2$ and 141 mg (0.50 mmol) of **1n**. The product (*S*)-**3n** was obtained in 54% yield (111 mg, 0.27 mmol, colorless oil) with 95% ee, **3**/**4** = 90:10. The regioselectivity was determined by GC analysis using the crude material.

^{1}H NMR (392 MHz, CDCl$_3$, δ): 0.62 (s, 3H), 0.94–1.05 (m, 4H), 1.22 (s, 12H), 1.26–1.50 (m, 2H), 1.54–1.64 (m, 2H), 3.68 (t, *J* = 6.3 Hz, 2H), 7.34–7.42 (m, 6H), 7.57–7.60 (m, 4H). ^{13}C NMR (99 MHz, CDCl$_3$, δ): –3.0 (*C*H$_3$), 15.5 (*C*H$_3$), 16.8 (br, B–*C*H), 24.68 (*C*H$_3$), 24.70 (*C*H$_3$), 29.2 (*C*H$_2$), 31.9 (*C*H$_2$), 63.9 (*C*H$_2$), 82.7 (*C*), 127.7 (*C*H), 129.6 (*C*H), 134.3 (*C*H), 136.3 (*C*). HRMS–EI (*m*/*z*): [M]$^+$ calcd for $C_{24}H_{35}BSiO_3$, 410.2453; found, 410.2444. $[\alpha]_D^{22}$ + 4.0 (c 1.0, CHCl$_3$, 95% ee). The ee value was determined by HPLC analysis of the corresponding alcohol after NaBO$_3$•4H$_2$O oxidation of (*S*)-**3n** in comparison of the racemic sample. Daicel CHIRALPAK® OD-3, 2-PrOH/hexane = 3/97, 0.50 mL/min, 40 ºC, *R* isomer: t_R = 16.4 min., *S* isomer: t_R = 17.3 min.

(*S*)-2-(1-Cyclohexylpropan-2-yl)-4,4,5,5-tetramethyl-1,3,2-dioxaborolane [(*S*)-3o] [8,18].

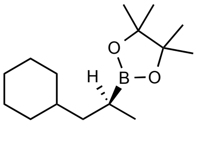

(*S*)-**3o**

According to the general borylation procedure, the reaction was conducted with 5 mol % [Cu(MeCN)$_4$]BF$_4$/(*S*)-Quinox-*t*OctAd$_2$ and 62.1 mg (0.50 mmol) of **1o**. The product (*S*)-**3o** was obtained in 67% yield (84.7 mg, 0.34 mmol, colorless oil) with 95% ee, **3**/**4** = 83:17. The regioselectivity was determined by GC analysis using the crude material.

(*S*)-**3o** (*branched*) (22): ^{1}H NMR (392 MHz, CDCl$_3$, δ): 0.79–0.94 (m, 4H), 1.06–1.44 (m, 19H), 1.58–1.70 (m, 6H). ^{13}C NMR (99 MHz, CDCl$_3$, δ): 13.9 (br, B–*C*H), 15.7 (*C*H$_3$), 24.7 (*C*H$_3$), 26.4 (*C*H$_2$), 26.5 (*C*H$_2$), 26.7 (*C*H$_2$), 33.1 (*C*H$_2$), 33.6 (*C*H$_2$), 36.5 (*C*H), 40.7

(CH_2), 82.7 (C). **4o** (*linear*) (50): ^{1}H NMR (392 MHz, $CDCl_3$, δ): 0.74 (t, J = 6.7 Hz, 2H), 0.79–0.94 (m, 2H), 1.06–1.44 (m, 19H), 1.58–1.70 (m, 6H). ^{13}C NMR (99 MHz, $CDCl_3$, δ): 13.9 (br, B–CH), 21.3 (CH_2), 24.8 (CH_3), 26.4 (CH_2), 26.7 (CH_2), 33.4 (CH_2), 37.4 (CH), 40.4 (CH_2), 82.8 (C). HRMS–EI (*m*/*z*): $[M]^+$ calcd for, $C_{15}H_{29}BO_2$, 252.2263; found, 252.2268. $[\alpha]_D^{23}$ +2.9 (c 1.0, $CHCl_3$, 95% ee). The ee value was determined by HPLC analysis of the corresponding ester after $NaBO_3$•$4H_2O$ oxidation and esterification with *p*-nitrobenzoyl chloride of (*S*)-**3o** in comparison of the racemic sample. Daicel CHIRALPAK® OZ-3, 2-PrOH/hexane = 0.5/99.5, 0.50 mL/min, 40 ºC, *R* isomer: t_R = 25.8 min., *S* isomer: t_R = 27.6 min.

Borylation of Mixture of Octenes and Functionalization of Borylation Products

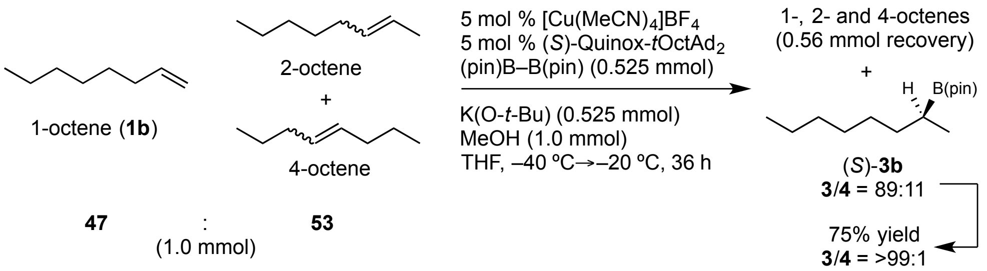

1-Octene selective asymmetric Markovnikov hydroboration. [Cu(MeCN)4]BF4 (7.9 mg, 0.025 mmol), bis(pinacolato)diboron (**2**) (133 mg, 0.53 mmol) and (*S*)-Quinox-tOctAd2 (14.7 mg, 0.025 mmol) were placed in an oven-dried reaction vial. After the vial was sealed with a screw cap containing a Teflon™-coated rubber septum, the vial was connected to a vacuum/nitrogen manifold through a needle. It was evacuated and then backfilled with nitrogen. This cycle was repeated three times. Dry THF (1.48 mL) and K(O-*t*-Bu)/THF (1.00 M, 0.53 mL, 0.53 mmol) were added in the vial through the rubber septum using a syringe. After stirring for 30 min at –40 ºC, the mixture of octenes [112 mg, 1.0 mmol, 47% 1-octene (**1b**)] and methanol (0.040 mL, 1.0 mmol) were added to the mixture at –40 °C. After stirring for 1 h, the mixture was warmed to –20 ºC and stirred for 35 h. The reaction mixture was then passed through a short silica gel (Φ: 10 mm, height of the silica-gel column: 30 mm) eluting with Et_2O. The crude material was purified by flash column chromatography (SiO_2, CH_2Cl_2/hexane, 0:100–32:68) to give the corresponding alkylboronate (*S*)-**3c** (91.8 mg, 0.38 mmol, 75% yield based on an amount of 1-octene in the mixture).

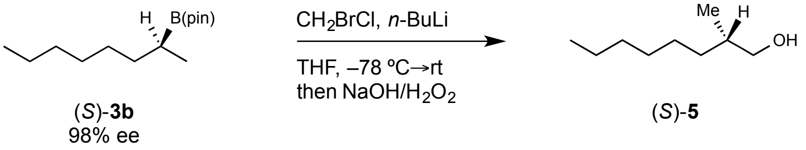

Synthesis of (*S*)-2-methyloctan-1-ol [(*S*)-5][19]. In an oven- dried reaction vial, (*S*)-**3b** (48.0 mg, 0.2 mmol) and bromochloromethane (51.8 mg, 0.4 mmol) were dissolved in dry THF (1.5 mL) under nitrogen atmosphere. After the mixture was cooled to –78 °C, a solution of *n*-BuLi in hexane (1.55 M, 194 µL, 0.3 mmol) was added dropwise. The mixture was stirred at –78 °C for 30 min, and then stirring at room temperature for 2 hours. The reaction mixture was quenched by addition of water and extracted three times with Et_2O. The combined organic layer was dried over $MgSO_4$. After filtration, the resulting crude material was submitted to the subsequent reaction without further purification. In a reaction vial,

the crude material of above reaction was dissolved in the mixture of THF (1.0 mL) and aqueous NaOH solution (3.0 M, 1.0 mL). Aqueous H_2O_2 solution (30%, 0.5 mL) was then added at 0 ºC. After stirring for 1 hours, the reaction mixture was quenched by saturated $Na_2S_2O_3$ solution at 0 ºC and extracted with Et_2O. The organic layer was dried over MgSO4. After filtration, the crude mixture was purified by flash column chromatography (SiO_2, Et_2O/hexane, 0:100–32:68) to afford the corresponding alcohol (25.7 mg, 0.18 mmol, 89%, 2 steps) as a colorless oil.

^{1}H NMR (392 MHz, $CDCl_3$, δ): 0.88 (t, J = 6.7 Hz, 3H), 0.92 (d, J = 6.3 Hz, 3H), 1.06–1.14 (m, O*H*, 1H), 1.21–1.48 (m, 10H), 1.55–1.67 (m, 1H), 3.42 (dd, J = 6.5, 10.8 Hz, 1H), 3.51 (dd, J = 5.8, 10.3 Hz, 1H). ^{13}C NMR (99 MHz, $CDCl_3$, δ): 14.0 (*C*H_3), 16.5 (*C*H_3), 22.6 (*C*H_2), 26.9 (*C*H_2), 29.6 (*C*H_2), 31.8 (*C*H_2), 33.1 (*C*H_2), 35.7 (*C*H), 68.3 (*C*H_2). HRMS-ESI (*m*/*z*): $[M+Na]^+$ calcd for $C_9H_{20}O_1Na$, 167.1412; found, 167.1412. $[\alpha]_D^{23}$ –14 (c 1.0, $CHCl_3$, 98% ee). The ee value was determined by HPLC analysis of the corresponding ester after esterification of (*S*)-**5** in comparison of the racemic sample. Daicel CHIRALPAK® OD-3, 2-PrOH/hexane = 0.1/99.9, 0.50 mL/min, 40 ºC, *R* isomer: t_R = 17.4 min., *S* isomer: t_R = 19.3 min. The absolute configuration of (*S*)-**5** was confirmed by the optical rotation value of the previous report[19].

Computational Study

Calculation method. All calculations were performed with Gaussian 09 (revision C.01) program package [20]. Geometry optimizations and transition state (TS) calculations were carried out at ωB97XD level of theory[21] with SDD basis set for Cu and 6-311G(d,p) basis set for the other atoms in the gas phase. Frequency calculations were conducted at the same level of theory on the optimized geometries to check the all the stationary points as either minima or transition states. The intrinsic reaction coordinate (IRC) was calculated for the transition states to confirm that the structures were indeed connected by two relevant minimas. Molecular structures were drawn using the Mercury 3.5 program.

Activation energy value analyses. According to the previous work for copper(I)-catalyzed hydroboration with diboron reagent, the reaction proceeds through the borylcupration from the π-complex (**II**) to give the alkylcopper(I) intermediate (**III**) followed by the protonation of **III** with alcohol to give the borylation products (**3** and **4**) and the copper alkoxide (**IV**) (Fig. S3). The transition state of the borylcupration steps (**TS**) are the highest stationary point in each routs and are the rate- and stereoselectivity determining step [8,12,22–26]. We thus calculated the π-complex (**II**), the transition state structures (**TS**) and the alkylcopper(I) intermediate (**III**) for the eight reaction pathways with (*S*)-Quinox-*t*Bu$_3$ and (*S*)-Quinox-Ad$_3$ respectively. The predicted stereoselectivities for the ligands were calculated from the activation energy values of **TS**s by Supplementary Equations (1) and (2) (Supplementary Figures 4–7).

$$ee_{pre}(\%) = \frac{\sum e^{-\frac{\Delta G_S}{RT}} - \sum e^{-\frac{\Delta G_R}{RT}}}{\sum e^{-\frac{\Delta G_S}{RT}} + \sum e^{-\frac{\Delta G_R}{RT}}} \times 100 \qquad (1)$$

$$\mathbf{3/4}_{pre} = \frac{\sum e^{-\frac{\Delta G_S}{RT}} + \sum e^{-\frac{\Delta G_R}{RT}}}{\sum e^{-\frac{\Delta G_S}{RT}} + \sum e^{-\frac{\Delta G_R}{RT}} + \sum e^{-\frac{\Delta G_L}{RT}}} \qquad (2)$$

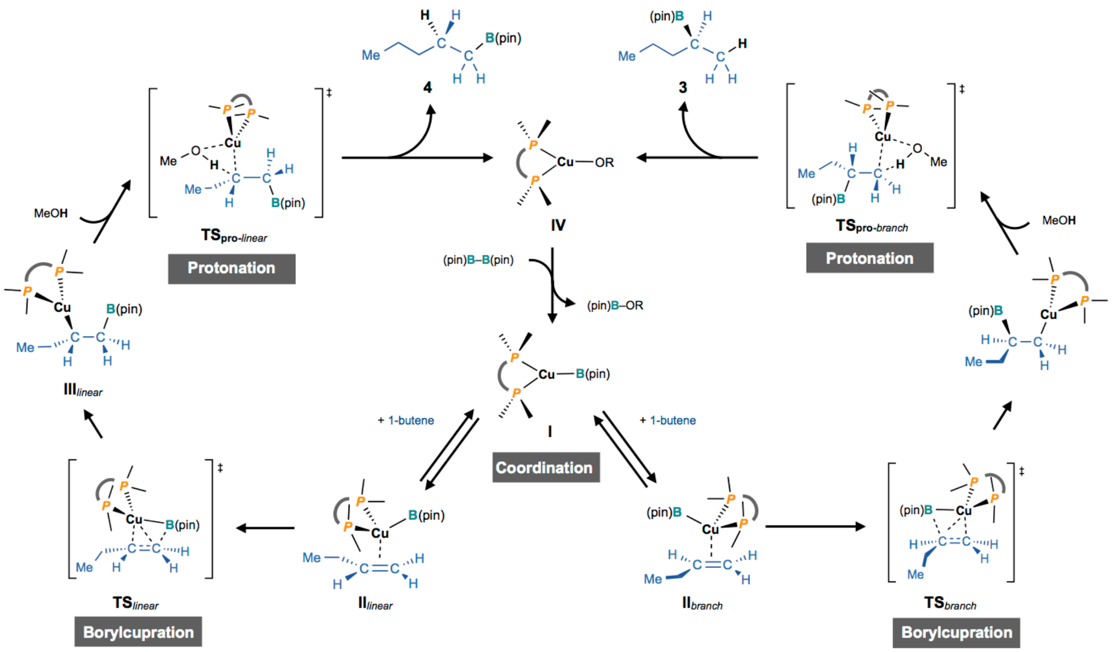

Supplementary Figure 3. Proposed reaction pathways for the branched product **3** and the linear product **4.**

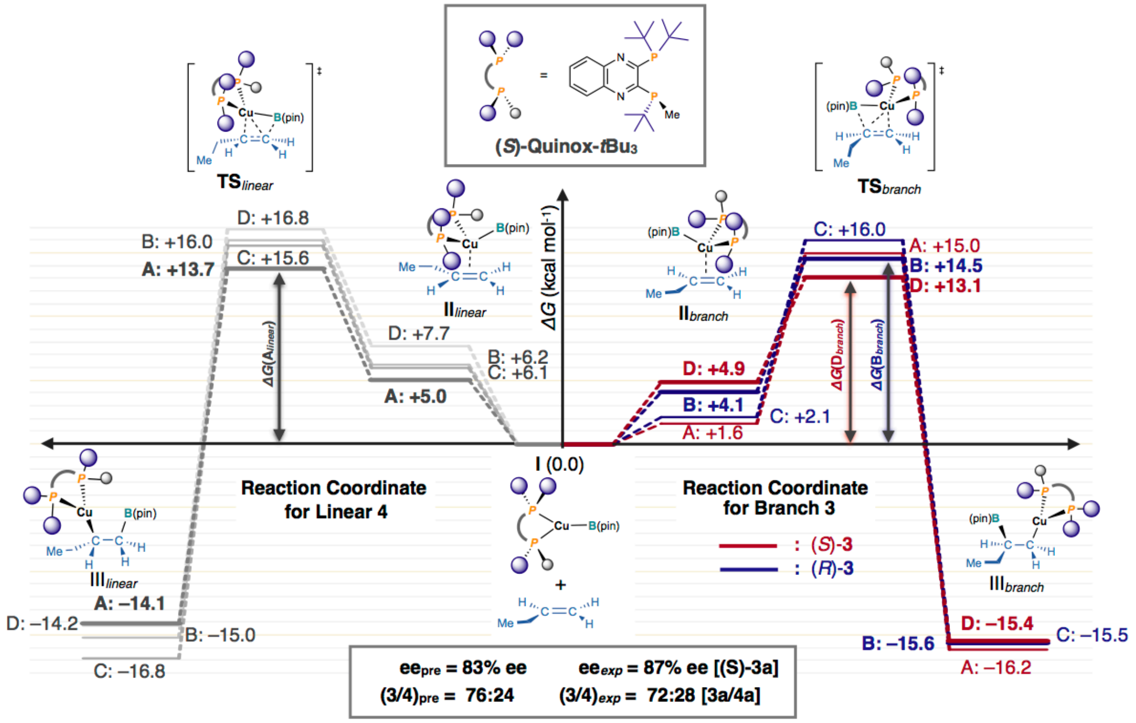

Supplementary Figure 4. Energy level diagram of borylcupration step with (*S*)-Quinox-*t*Bu$_3$.

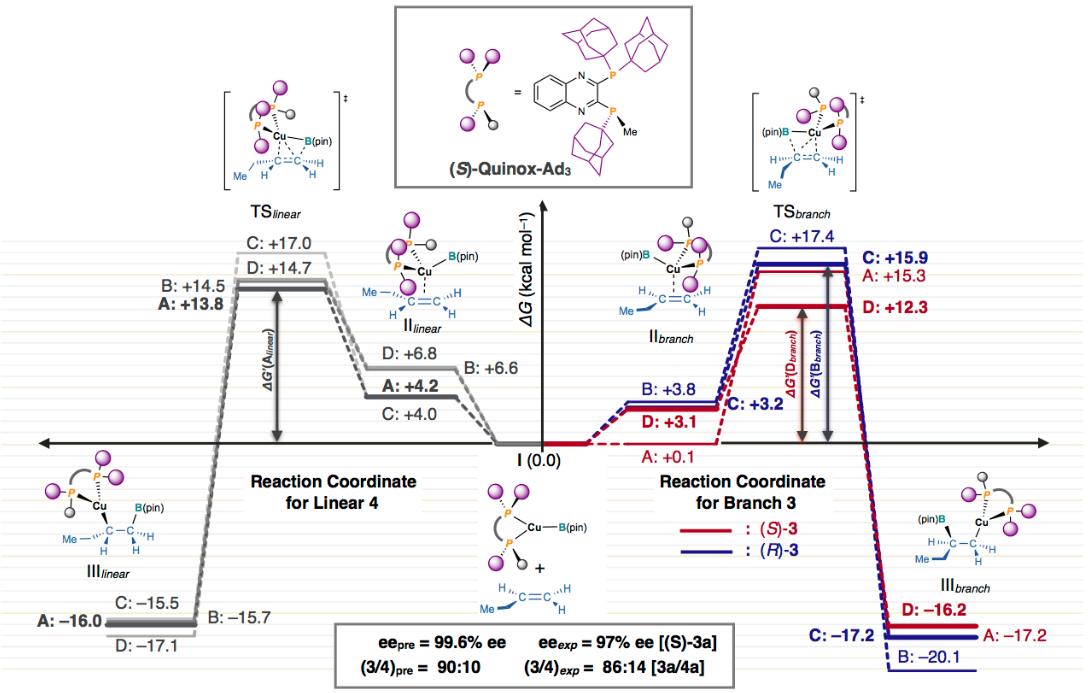

Supplementary Figure 5. Energy level diagram of borylcupration step with (*S*)-Quinox-Ad$_3$.

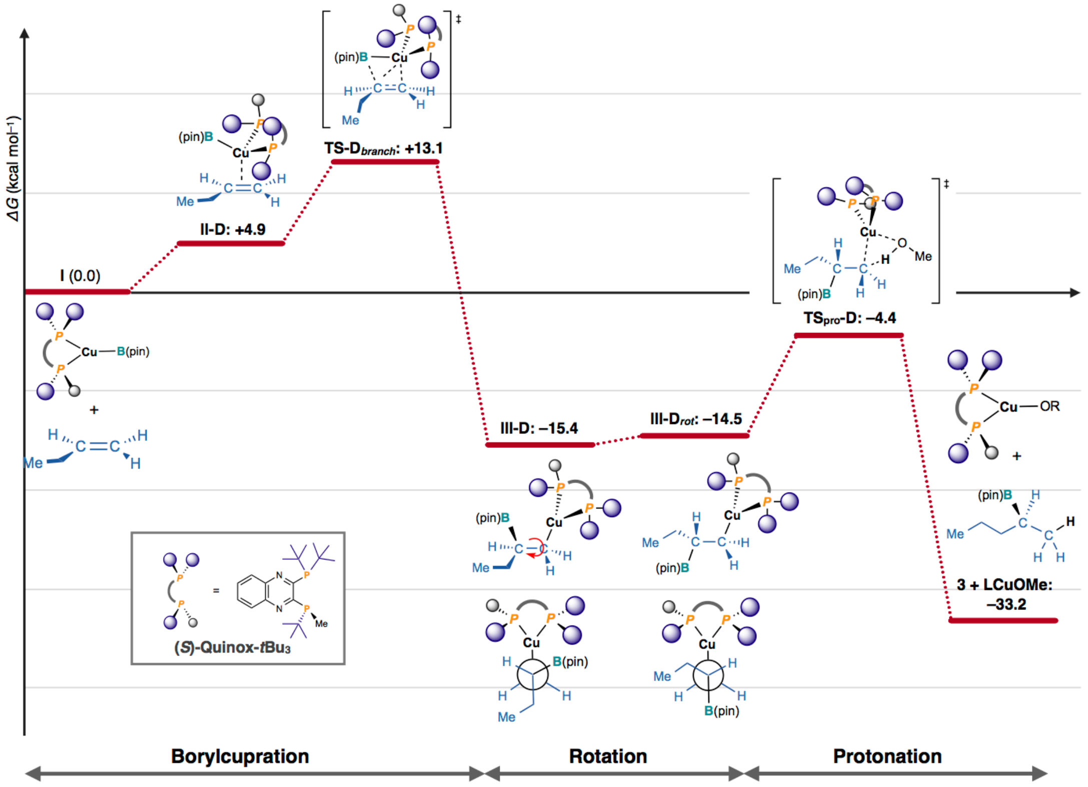

Supplementary Figure 6. Energy level diagram of reaction coordinate including protonation of alkylcopper(I) intermediate with (*S*)-Quinox-*t*Bu$_3$.

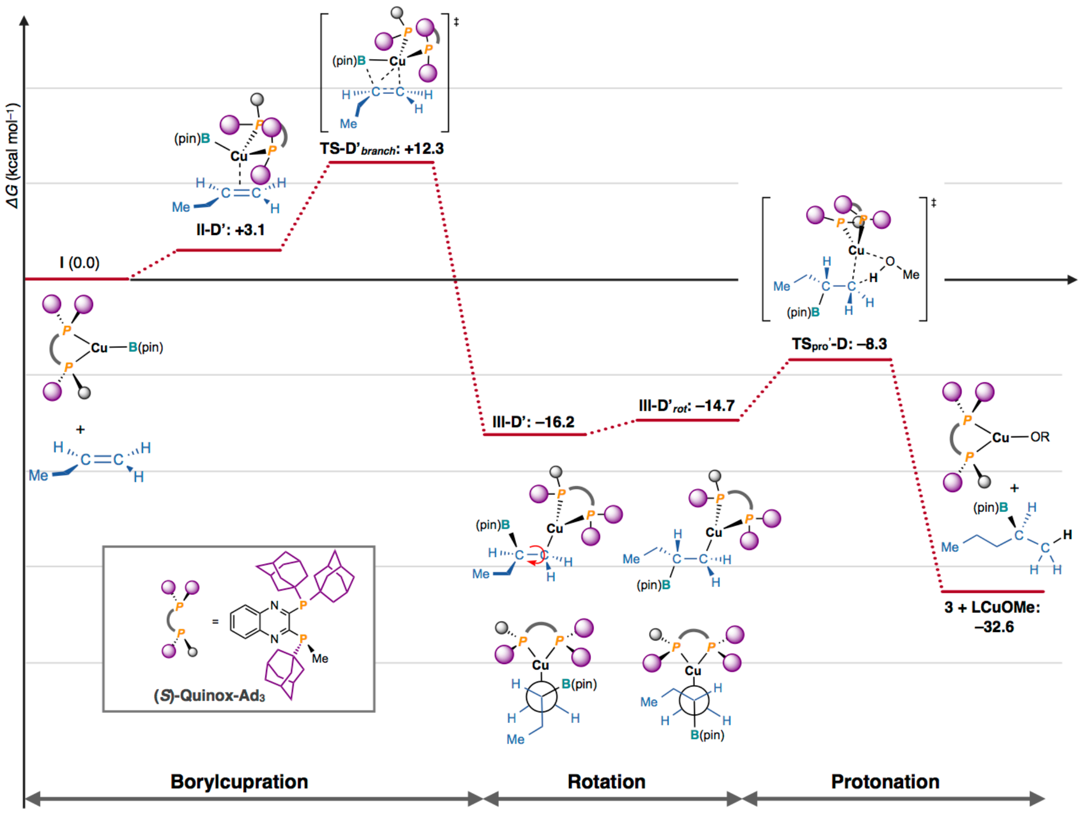

Supplementary Figure 7. Energy level diagram of reaction coordinate including protonation of alkylcopper(I) intermediate with (*S*)-Quinox-Ad3.

Analysis of optimized transition state structures and NCI Plots. The transition state (TS) structures of the borylcupration of 1-butene were obtained by the DFT calculation study. We then conducted the analysis of these structures focused on the steric congestion and strain to understand the origin of the regio- and enantioselectivity of the enantioselective Markovnikov hydroboration of aliphatic terminal alkenes. Furthermore, non-covalent interactions (NCI) analysis which is the visualization method of noncovalent interactions[27,28] were greatly helpful for searching steric congestion area in the TS structures.

TS-Abranch (+1.9)

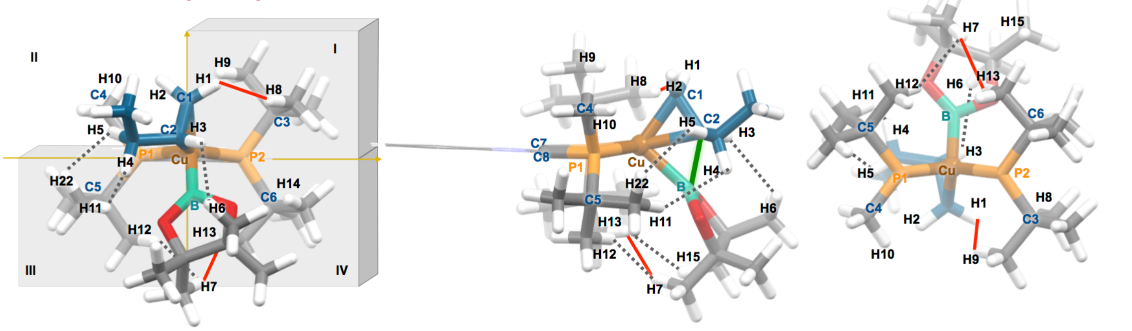

Front view　　Side view　　Back view

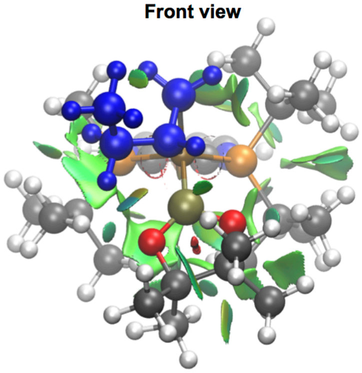

NCI plot

atom distance: H1–H8 = 2.08, H3–H6 = 2.58, H4–H11 = 2.59, H5–H11 = 2.68, H7–H12 = 2.51, H7–H13 = 2.04, H13–H15 = 2.66, C3–C4 = 5.51, C5–C6 = 5.51

bond length: P1–Cu = 2.26, P2–Cu = 2.26, C1–Cu = 2.01, C2–Cu = 2.06, Cu–B = 2.09, C2–B = 1.94

bond angle: B–C2–C1 = 126.90°, C3–P2–C6 = 111.42°, C6–P2–C7 = 102.76°, C7–P2–C3 = 102.89°, C4–P1–C5 = 104.06°, C5–P1–C8 = 105.12°, C8–P1–C4 = 99.87°

dihedral angle: B–Cu–C1–C2 = –14.68°

TS-Bbranch (+1.4)

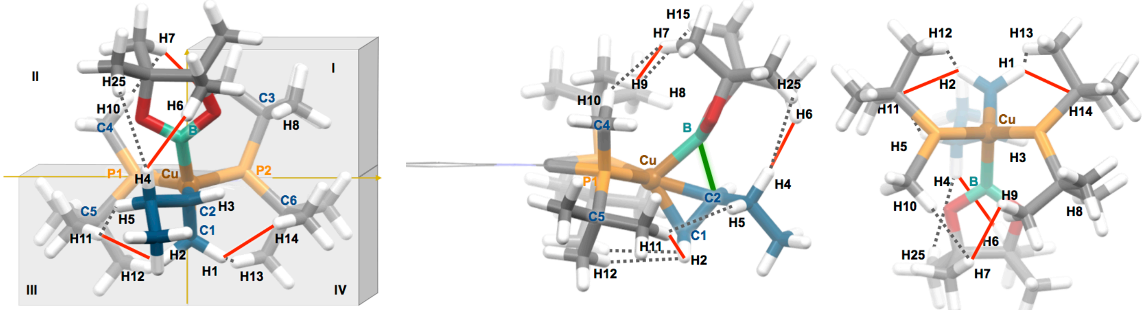

Front view　　Side view　　Back view

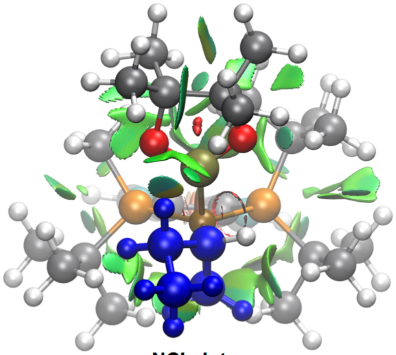

NCI plot

atom distance: H1–H13 = 2.46, H1–H14 = 2.11, H2–H11 = 2.26, H2–H12 = 2.56, H4–H6 = 2.29, H4–H25 = 2.65, H5–H11 = 2.34, H7–H9 = 2.09, H7–H10 = 2.51, H9–H15 = 2.62, C3–C4 = 4.95, C5–C6 = 5.91

bond length: P1–Cu = 2.24, P2–Cu = 2.28, C1–Cu = 2.00, C2–Cu = 2.06, Cu–B = 2.06, C2–B = 2.01

bond angle: B–C2–C1 = 127.22°, C3–P2–C6 = 112.01°, C6–P2–C7 = 104.86°, C7–P2–C3 = 100.46°, C4–P1–C5 = 104.71°, C5–P1–C8 = 104.48°, C8–P1–C4 = 100.89°

dihedral angle: B–Cu–C1–C2 = –3.08°

TS-C$_{branch}$ (+2.9)

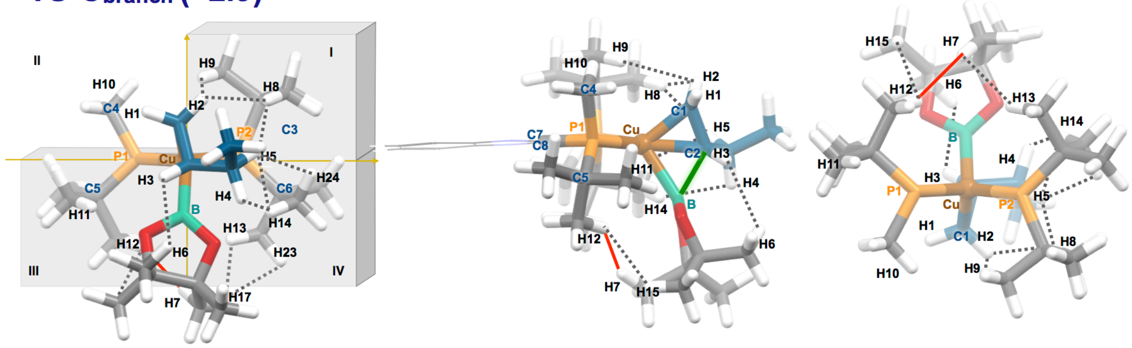

Front view   Side view   Back view

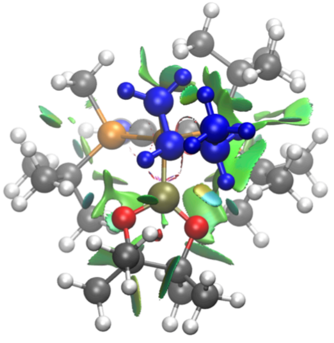

NCI plot

atom distance: H2–H8 = 2.42, H2–H9 = 2.56, H3–H6 = 2.53, H4–H14 = 2.56, H5–H8 = 2.32, H5–H14 = 2.58, H5–H24 = 2.66, H7–H12 = 2.17, H7–H13 = 2.38, H12–H15 = 2.53, H13–H17 = 2.56, C3–C4 = 4.88, C5–C6 = 6.00

bond length: P1–Cu = 2.25, P2–Cu = 2.28, C1–Cu = 2.00, C2–Cu = 2.07, Cu–B = 2.11, C2–B = 1.90

bond angle: B–C2–C1 = 127.30º, C3–P2–C6 = 112.40º, C6–P2–C7 = 103.92º, C7–P2–C3 = 101.02º, C4–P1–C5 = 103.69º, C5–P1–C8 = 105.40º, C8–P1–C4 = 99.81º

dihedral angle: B–Cu–C1–C2 = 14.54º

TS-D$_{branch}$ (+0.0)

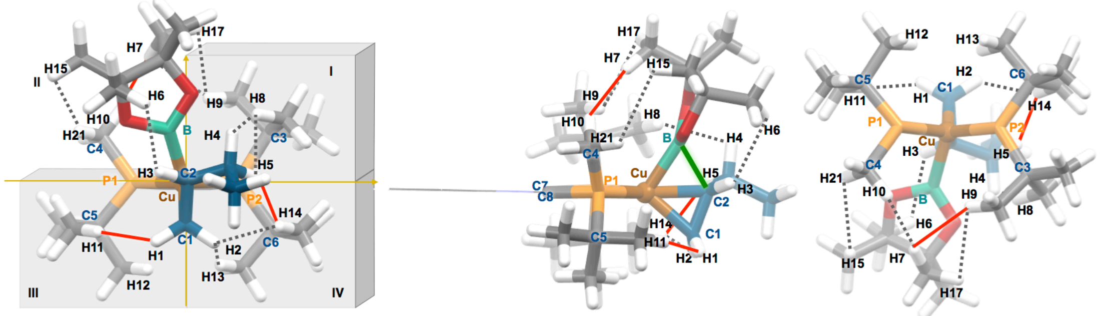

Front view   Side view   Back view

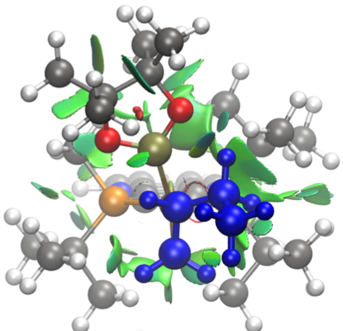

NCI plot

atom distance: H1–H11 = 2.24, H2–H13 = 2.47, H2–H14 = 2.38, H3–H6 = 2.49, H4–H8 = 2.60, H5–H8 = 2.60, H5–H14 = 2.25, H7–H9 = 2.42, H7–H10 = 2.24, H9–H17 = 2.64, H15–H21 = 2.67, C3–C4 = 5.32, C5–C6 = 5.59

bond length: P1–Cu = 2.23, P2–Cu = 2.28, C1–Cu = 2.01, C2–Cu = 2.05, Cu–B = 2.07, C2–B = 1.98

bond angle: B–C2–C1 = 125.46º, C3–P2–C6 = 112.89º, C6–P2–C7 = 102.86º, C7–P2–C3 = 102.27º, C4–P1–C5 = 104.59º, C5–P1–C8 = 105.56º, C8–P1–C4 = 99.55º

dihedral angle: B–Cu–C1–C2 = –17.33º

TS-A$_{linear}$ (+0.6)

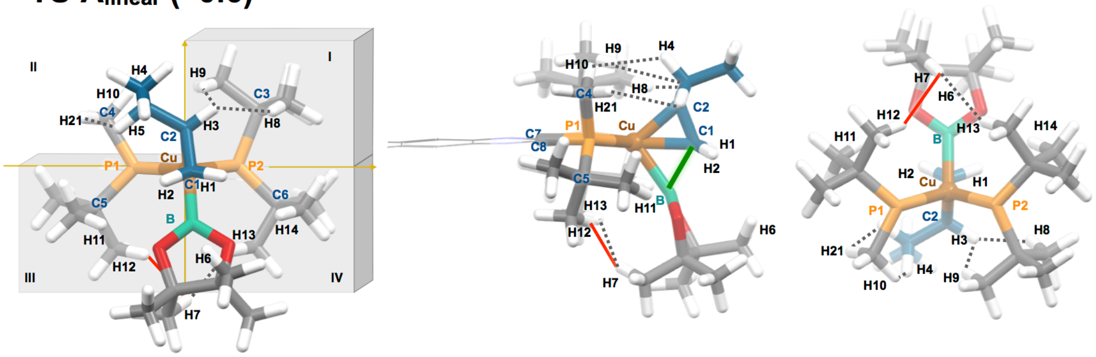

Front view Side view Back view

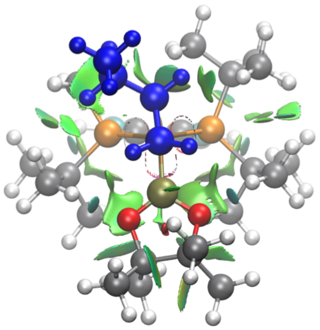

NCI plot

atom distance: H3–H8 = 2.34, H3–H9 = 2.54, H4–H10 = 2.59, H5–H21 = 2.63, H7–H12 = 2.28, H7–H13 = 2.37, C3–C4 = 4.91, C5–C6 = 5.96

bond length: P1–Cu = 2.25, P2–Cu = 2.27, C1–Cu = 2.04, C2–Cu = 2.01, Cu–B = 2.12, C1–B = 1.88

bond angle: B–C1–C2 = 132.48°, C3–P2–C6 = 112.75°, C6–P2–C7 = 104.38°, C7–P2–C3 = 100.52°, C4–P1–C5 = 103.93°, C5–P1–C8 = 104.37°, C8–P1–C4 = 100.27°

dihedral angle: B–Cu–C2–C1 = 3.55°

TS-B$_{linear}$ (+2.9)

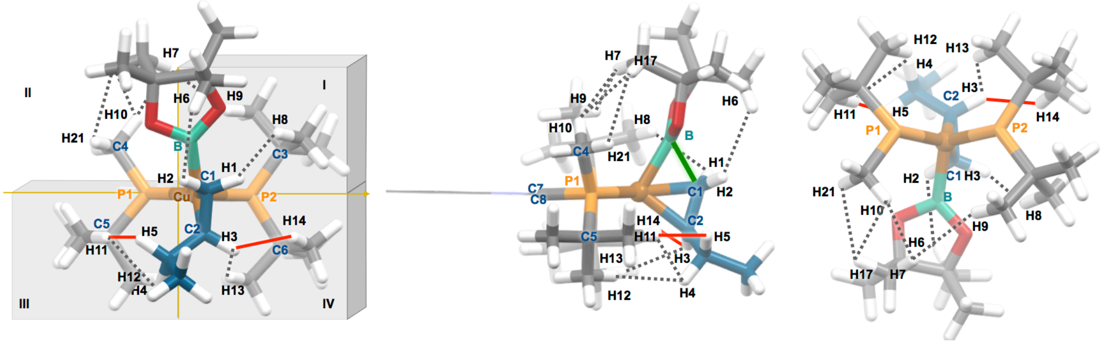

Front view Side view Back view

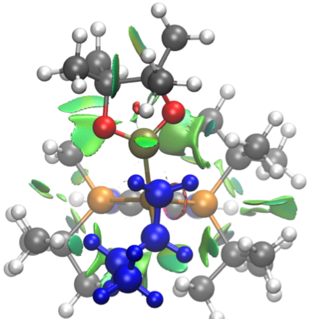

NCI plot

atom distance: H1–H8 = 2.69, H2–H6 = 2.54, H3–H13 = 2.35, H3–H14 = 2.25, H4–H11 = 2.51, H4–H12 = 2.37, H5–H11 = 2.25, H7–H9 = 2.61, H7–H10 = 2.36, H10–H21 = 2.56, H17–H21 = 2.57, C3–C4 = 5.42, C5–C6 = 5.54

bond length: P1–Cu = 2.23, P2–Cu = 2.30, C1–Cu = 2.02, C2–Cu = 2.03, Cu–B = 2.08, C1–B = 1.96

bond angle: B–C1–C2 = 130.09°, C3–P2–C6 = 112.10°, C6–P2–C7 = 102.78°, C7–P2–C3 = 102.59°, C4–P1–C5 = 104.27°, C5–P1–C8 = 105.25°, C8–P1–C4 = 99.37°

dihedral angle: B–Cu–C2–C1 = –12.24°

TS-C$_{linear}$ (+2.5)

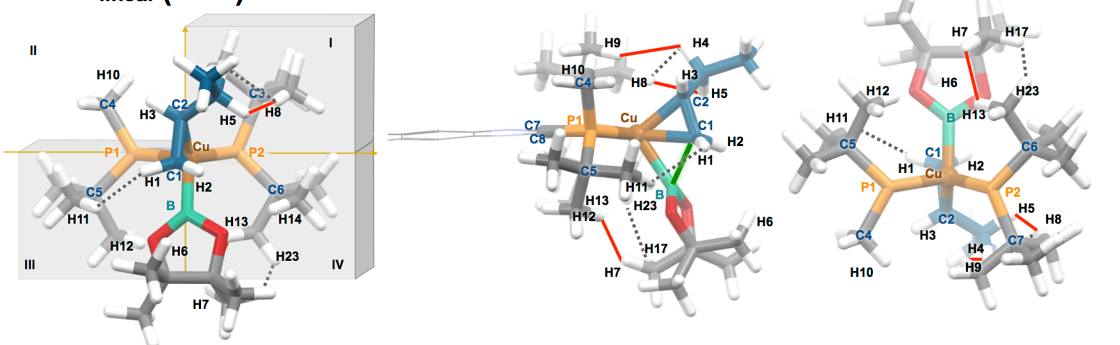

Front view — Side view — Back view

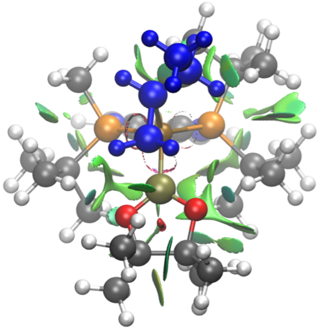

NCI plot

atom distance: H1–H11 = 2.66, H4–H8 = 2.44, H4–H9 = 2.29, H5–H8 = 2.18, H7–H13 = 2.17, H17–H23 = 2.66, C3–C4 = 4.94, C5–C6 = 5.95

bond length: P1–Cu = 2.30, P2–Cu = 2.25, C1–Cu = 2.04, C2–Cu = 2.03, Cu–B = 2.12, C1–B = 1.88

bond angle: B–C1–C2 = 132.75°; C3–P2–C6 = 111.97°, C6–P2–C7 = 103.84°, C7–P2–C3 = 100.55°; C4–P1–C5 = 104.19°, C5–P1–C8 = 104.67°, C8–P1–C4 = 99.93°

dihedral angle: B–Cu–C2–C1 = –6.72°

TS-D$_{linear}$ (+3.6)

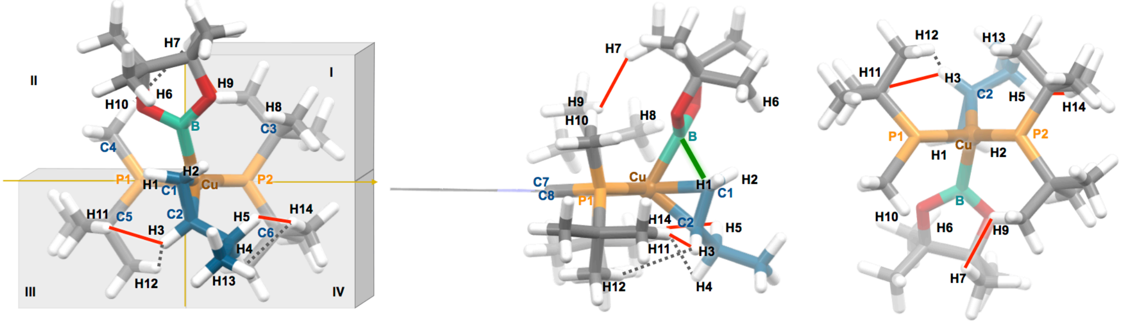

Front view — Side view — Back view

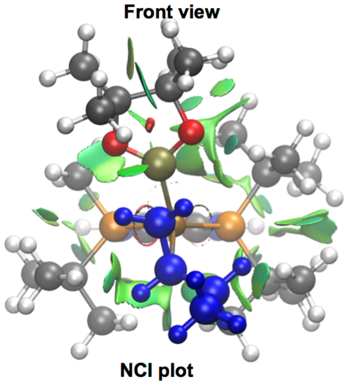

NCI plot

atom distance: H3–H11 = 2.15, H3–H12 = 2.64, H4–H13 = 2.19, H4–H14 = 2.48, H5–H14 = 2.14, H7–H9 = 2.21, C3–C4 = 5.41, C5–C6 = 5.55

bond length: P1–Cu = 2.28, P2–Cu = 2.26, C1–Cu = 2.03, C2–Cu = 2.03, Cu–B = 2.09, C1–B = 1.95

bond angle: B–C1–C2 = 131.91°; C3–P2–C6 = 112.00°, C6–P2–C7 = 102.33°, C7–P2–C3 = 102.48°; C4–P1–C5 = 104.21°, C5–P1–C8 = 105.53°, C8–P1–C4 = 99.13°

dihedral angle: B–Cu–C2–C1 = 2.22°

Supplementary Figure 8. Transition state structures with (*S*)-Quinox-*t*Bu$_3$. In the back views, the quinoxaline backbone was omitted for clarity (H–H distance < 2.3 Å: red line; 2.3 Å < H–H distance < 2.7 Å: gray dotted line).

TS-A'$_{branch}$ (+3.0)

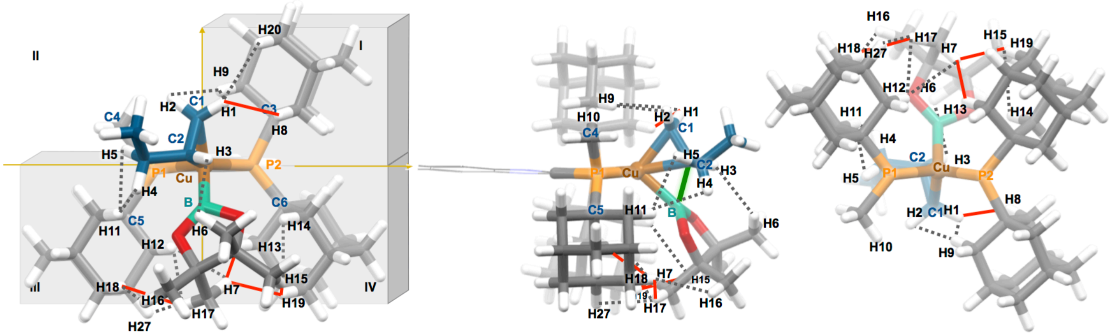

Front view   Side view   Back view

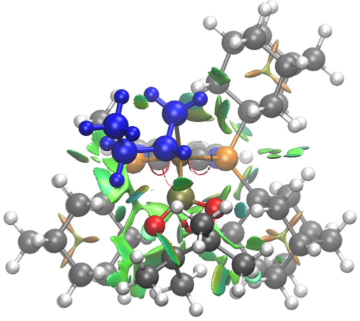

NCI plot

atom distance:
H1–H8 = 2.23
H1–H9 = 2.56
H1–H20 = 2.60
H2–H9 = 2.66
H3–H6 = 2.48
H4–H11 = 2.42
H5–H11 = 2.43
H7–H12 = 2.55
H7–H13 = 2.21
H7–H19 = 2.05
H12–H17 = 2.50
H14–H15 = 2.69
H15–H19 = 2.06
H16–H18 = 2.45
H17–H18 = 2.16
H17–H27 = 2.55
C3–C4 = 5.34
C5–C6 = 5.55

bond length:
P1–Cu = 2.28
P2–Cu = 2.26
C1–Cu = 2.01
C2–Cu = 2.07
Cu–B = 2.11
C2–B = 1.90

bond angle:
B–C2–C1 = 127.18°
C3–P2–C6 = 114.02°
C6–P2–C7 = 102.33°
C7–P2–C3 = 100.80°
C4–P1–C5 = 104.72°
C5–P1–C8 = 104.65°
C8–P1–C4 = 99.61°

dihedral angle: B–Cu–C1–C2 = –15.67°

TS-B'$_{branch}$ (+5.1)

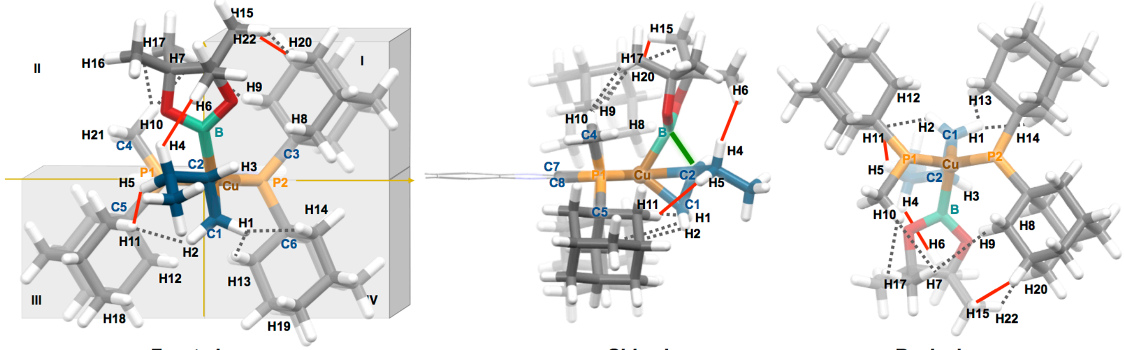

Front view   Side view   Back view

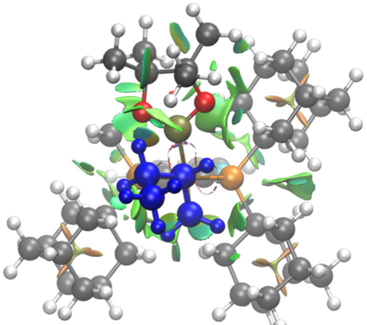

NCI plot

atom distance:
H1–H13 = 2.61
H1–H14 = 2.30
H2–H11 = 2.34
H4–H6 = 2.09
H5–H11 = 2.20
H7–H9 = 2.39
H7–H10 = 2.50
H10–H17 = 2.33
H15–H20 = 2.11
H20–H22 = 2.40
C3–C4 = 5.52
C5–C6 = 5.41

bond length:
P1–Cu = 2.26
P2–Cu = 2.27
C1–Cu = 1.99
C2–Cu = 2.08
Cu–B = 2.11
C2–B = 1.93

bond angle:
B–C2–C1 = 128.68°
C3–P2–C6 = 112.09°
C6–P2–C7 = 101.10°
C7–P2–C3 = 104.57°
C4–P1–C5 = 104.36°
C5–P1–C8 = 106.00°
C8–P1–C4 = 98.49°

dihedral angle: B–Cu–C1–C2 = 1.37°

TS-C'$_{branch}$ (+3.6)

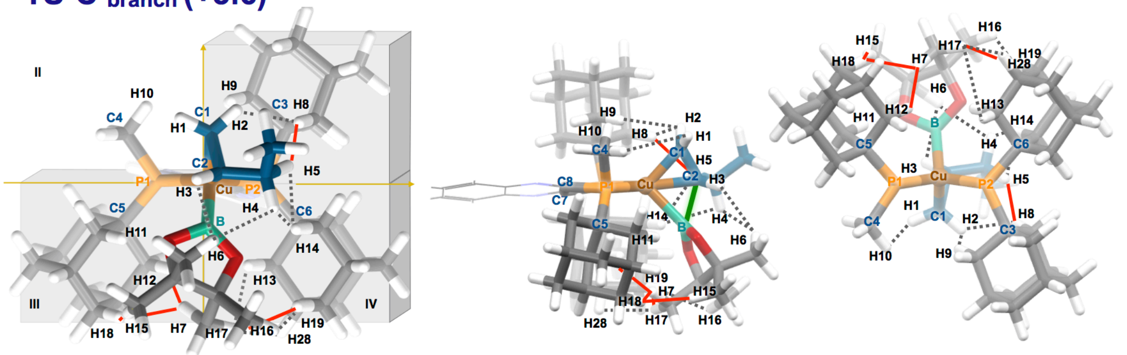

Front view   Side view   Back view

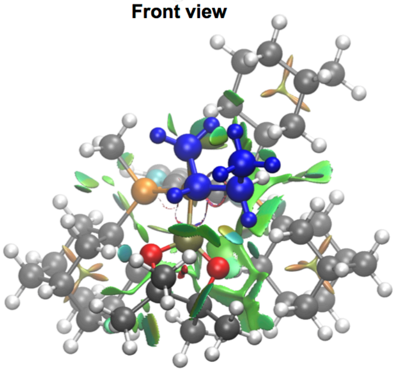

NCI plot

atom distance:
H2–H8 = 2.34
H2–H9 = 2.36
H3–H6 = 2.48
H4–H6 = 2.61
H4–H14 = 2.51
H5–H8 = 2.16
H5–H14 = 2.39
H7–H12 = 2.13
H7–H18 = 2.17
H13–H17 = 2.53
H15–H18 = 2.02
H16–H19 = 2.31
H17–H19 = 2.15
H17–H28 = 2.63
C3–C4 = 5.13
C5–C6 = 5.72

bond length:
P1–Cu = 2.24
P2–Cu = 2.30
C1–Cu = 2.00
C2–Cu = 2.07
Cu–B = 2.12
C2–B = 1.88

bond angle:
B–C2–C1 = 127.51º
C3–P2–C6 = 113.50º
C6–P2–C7 = 104.97º
C7–P2–C3 = 98.99º
C4–P1–C5 = 104.53º
C5–P1–C8 = 101.32º
C8–P1–C4 = 101.18º

dihedral angle: B–Cu–C1–C2 = 15.39º

TS-D'$_{branch}$ (+0.0)

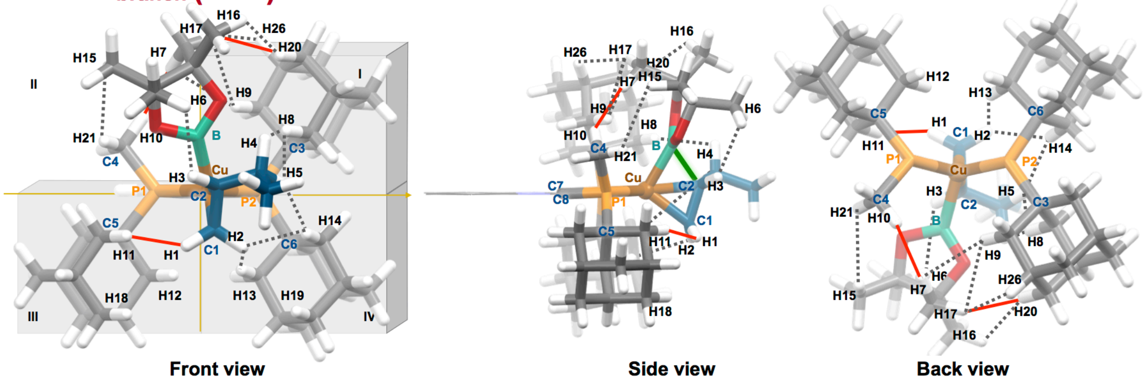

Front view   Side view   Back view

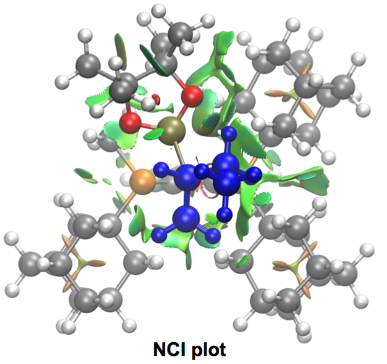

NCI plot

atom distance:
H1–H11 = 2.27
H2–H13 = 2.40
H2–H14 = 2.52
H3–H6 = 2.48
H4–H8 = 2.43
H5–H8 = 2.41
H5–H14 = 2.36
H7–H10 = 2.18
H7–H9 = 2.53
H9–H17 = 2.52
H15–H21 = 2.68
H16–H20 = 2.52
H17–H20 = 2.19
H17–H26 = 2.50
C3–C4 = 5.44
C5–C6 = 5.46

bond length:
P1–Cu = 2.23
P2–Cu = 2.28
C1–Cu = 2.01
C2–Cu = 2.05
Cu–B = 2.07
C2–B = 1.98

bond angle:
B–C2–C1 = 125.50º
C3–P2–C6 = 112.73º
C6–P2–C7 = 102.48º
C7–P2–C3 = 103.08º
C4–P1–C5 = 104.68º
C5–P1–C8 = 105.71º
C8–P1–C4 = 99.69º

dihedral angle: B–Cu–C1–C2 = –17.36º

TS-A'$_{linear}$ (+1.5)

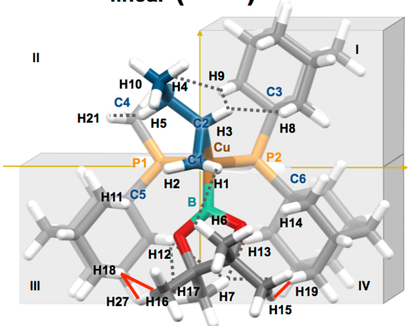

Front view

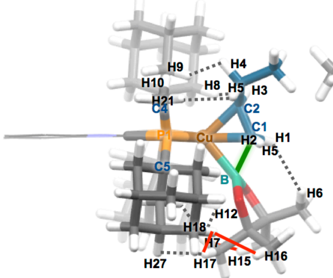

Side view

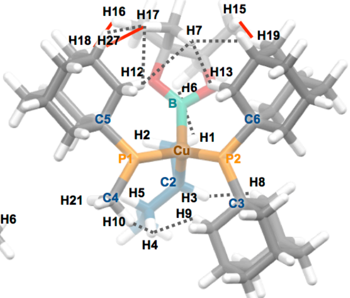

Back view

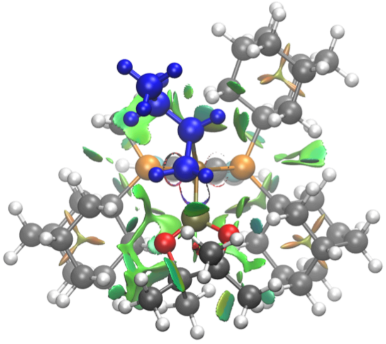

NCI plot

atom distance: H1–H6 = 2.46, H3–H8 = 2.37, H3–H9 = 2.54, H4–H9 = 2.39, H4–H10 = 2.58, H5–H21 = 2.44, H7–H12 = 2.67, H7–H13 = 2.31, H7–H19 = 2.39, H12–H17 = 2.41, H17–H18 = 2.23, H17–H27 = 2.47, H16–H18 = 2.22, H15–H19 = 1.97, C3–C4 = 4.98, C5–C6 = 5.85

bond length: P1–Cu = 2.29, P2–Cu = 2.25, C1–Cu = 2.05, C2–Cu = 2.02, Cu–B = 2.14, C2–B = 1.85

bond angle: B–C1–C2 = 132.95º, C3–P2–C6 = 113.15º, C6–P2–C7 = 103.44º, C7–P2–C3 = 100.63º, C4–P1–C5 = 104.42º, C5–P1–C8 = 105.20º, C8–P1–C4 = 99.03º

dihedral angle: B–Cu–C2–C1 = –8.13

TS-B'$_{linear}$ (+2.2)

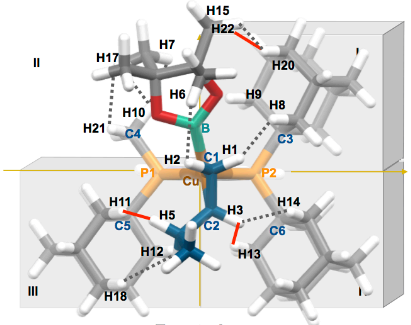

Front view

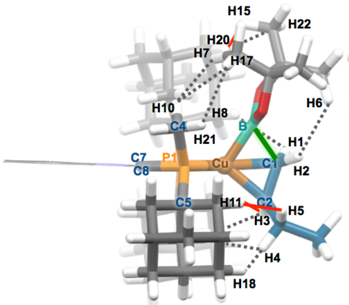

Side view

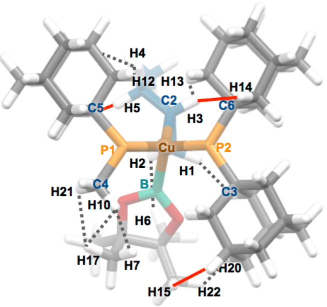

Back view

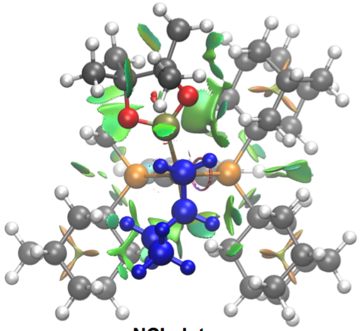

NCI plot

atom distance: H1–H8 = 2.63, H2–H6 = 2.44, H3–H13 = 2.36, H3–H14 = 2.13, H4–H12 = 2.30, H4–H18 = 2.38, H5–H11 = 2.02, H7–H10 = 2.39, H10–H17 = 2.64, H15–H20 = 2.29, H17–H21 = 2.46, H20–H22 = 2.48, C3–C4 = 5.37, C5–C6 = 5.51

bond length: P1–Cu = 2.23, P2–Cu = 2.30, C1–Cu = 2.02, C2–Cu = 2.03, Cu–B = 2.09, C2–B = 1.95

bond angle: B–C1–C2 = 130.31º, C3–P2–C6 = 113.38º, C6–P2–C7 = 102.57º, C7–P2–C3 = 102.70º, C4–P1–C5 = 104.40º, C5–P1–C8 = 105.27º, C8–P1–C4 = 99.29º

dihedral angle: B–Cu–C2–C1 = –12.76º

TS-C'$_{linear}$ (+4.7)

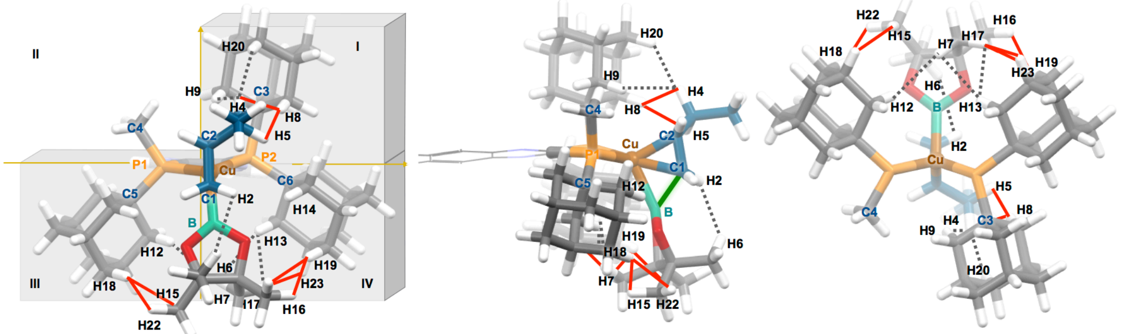

Front view   Side view   Back view

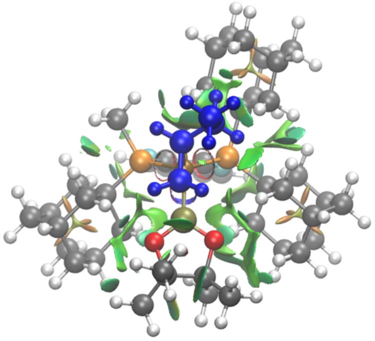

NCI plot

atom distance:
H2–H6 = 2.57
H4–H8 = 2.29
H4–H9 = 2.40
H4–H20 = 2.30
H5–H8 = 2.21
H7–H12 = 2.45
H7–H13 = 2.49
H13–H17 = 2.31
H15–H18 = 2.26
H16–H19 = 2.21
H17–H19 = 2.19
H17–H23 = 2.29
H18–H22 = 2.27
C3–C4 = 4.71
C5–C6 = 6.16

bond length:
P1–Cu = 2.27
P2–Cu = 2.28
C1–Cu = 2.05
C2–Cu = 2.02
Cu–B = 2.16
C2–B = 1.85

bond angle:
B–C1–C2 = 134.31º
C3–P2–C6 = 110.58º
C6–P2–C7 = 105.77º
C7–P2–C3 = 99.53º
C4–P1–C5 = 102.81º
C5–P1–C8 = 104.46º
C8–P1–C4 = 101.18º

dihedral angle: B–Cu–C2–C1 = –2.96º

TS-D'$_{linear}$ (+2.4)

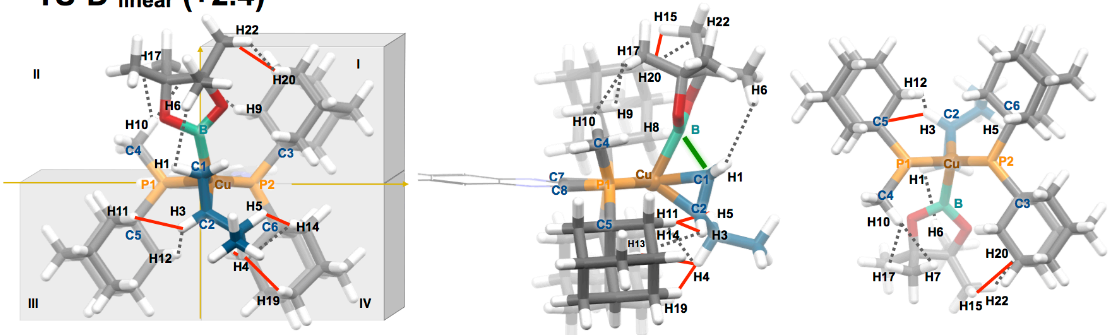

Front view   Side view   Back view

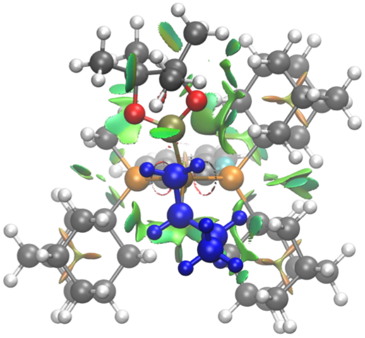

NCI plot

atom distance:
H1–H6 = 2.63
H3–H11 = 2.18
H3–H12 =2.67
H4–H13 = 2.22
H4–H14 = 2.57
H4–H19 = 2.23
H5–H14 = 2.13
H7–H9 = 2.40
H7–H10 = 2.59
H10–H17 = 2.32
H15–H20 = 2.22
H20–H22 = 2.45
C3–C4 = 5.56
C5–C6 = 5.42

bond length:
P1–Cu = 2.27
P2–Cu = 2.27
C1–Cu = 2.03
C2–Cu = 2.03
Cu–B = 2.09
C2–B = 1.95

bond angle:
B–C1–C2 = 131.97º
C3–P2–C6 = 111.31º
C6–P2–C7 = 100.94º
C7–P2–C3 = 104.46º
C4–P1–C5 = 104.24º
C5–P1–C8 = 107.06º
C8–P1–C4 = 98.22º

dihedral angle: B–Cu–C2–C1 = –3.46º

Supplementary Figure 9. Transition state structures with (*S*)-Quinox-Ad$_3$. In the back views, the quinoxaline backbone was omitted for clarity (H–H distance < 2.3 Å: red line; 2.3 Å < H–H distance < 2.7 Å: gray dotted line).

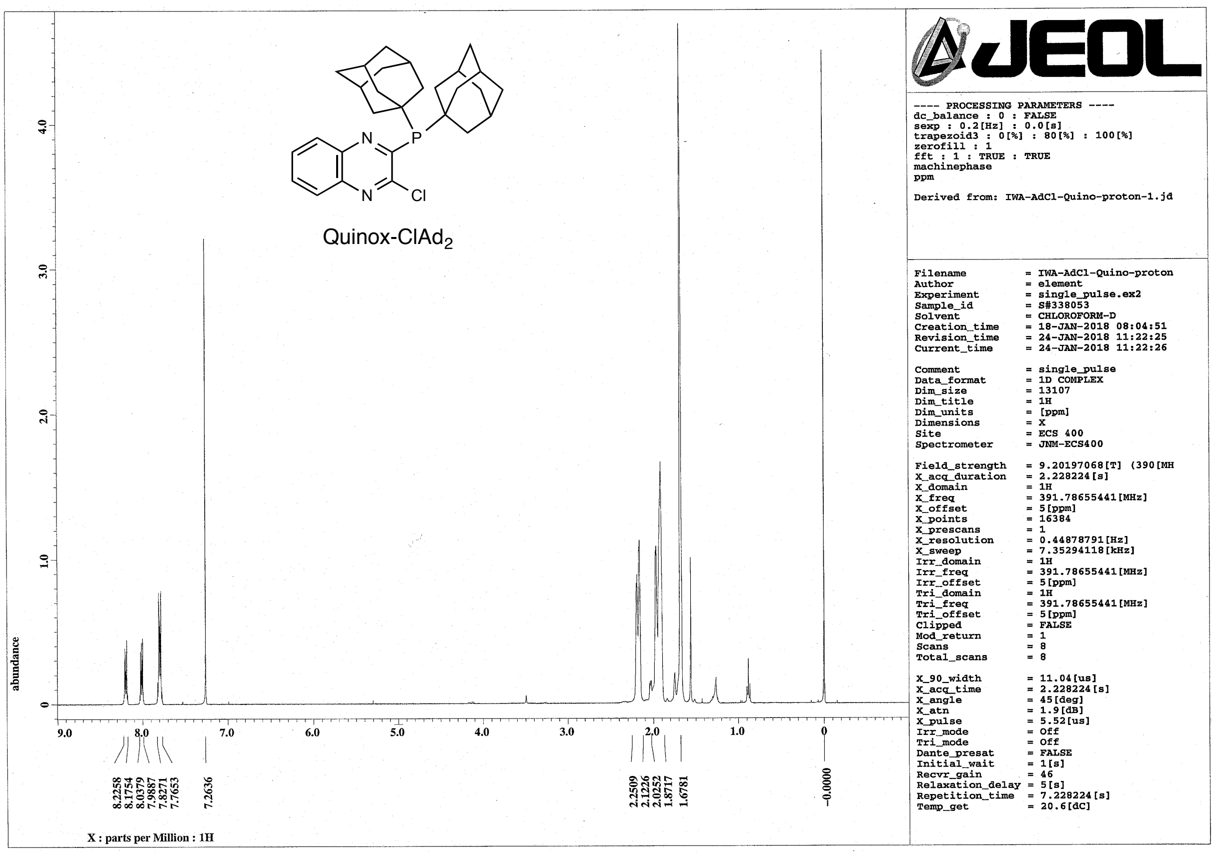

Supplementary Figure 10. ^{1}H NMR spectrum of Quinox-ClAd$_2$.

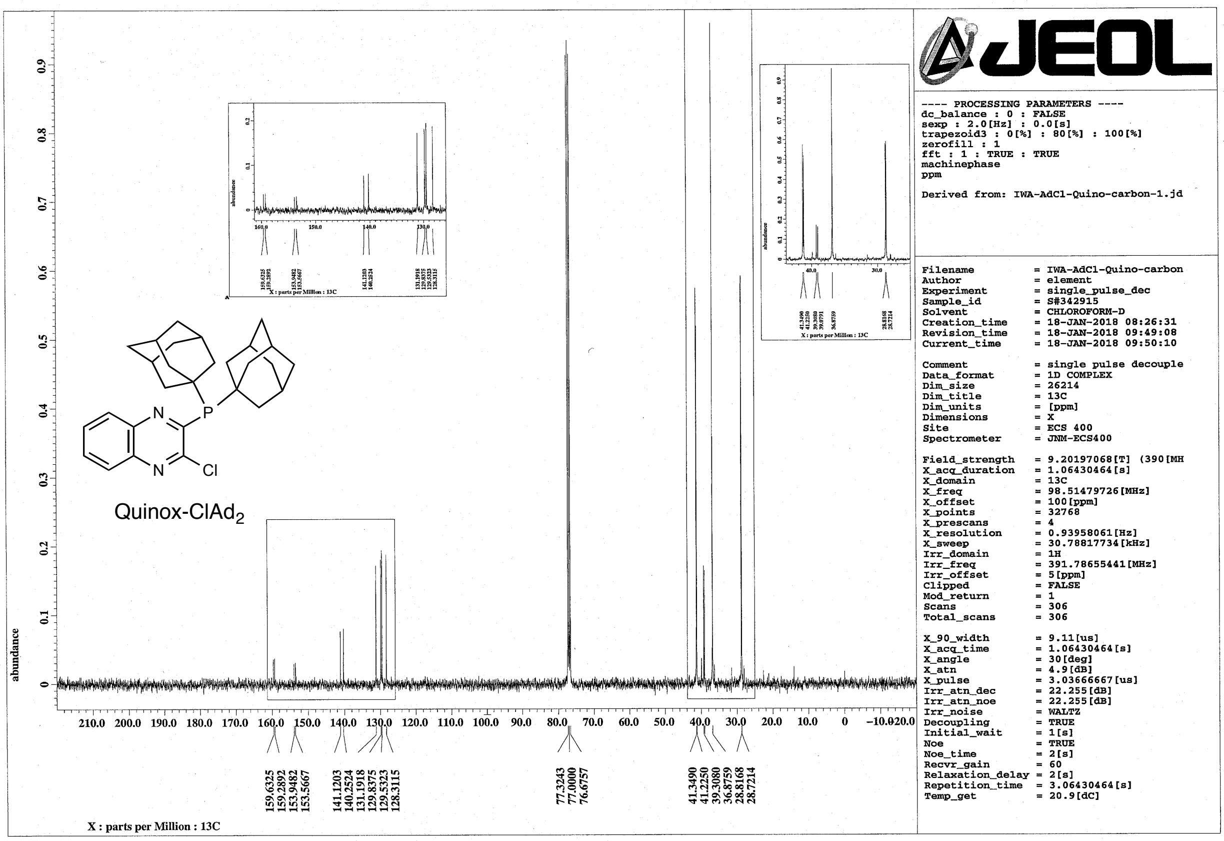

Supplementary Figure 11. ^{13}C NMR spectrum of Quinox-ClAd$_2$.

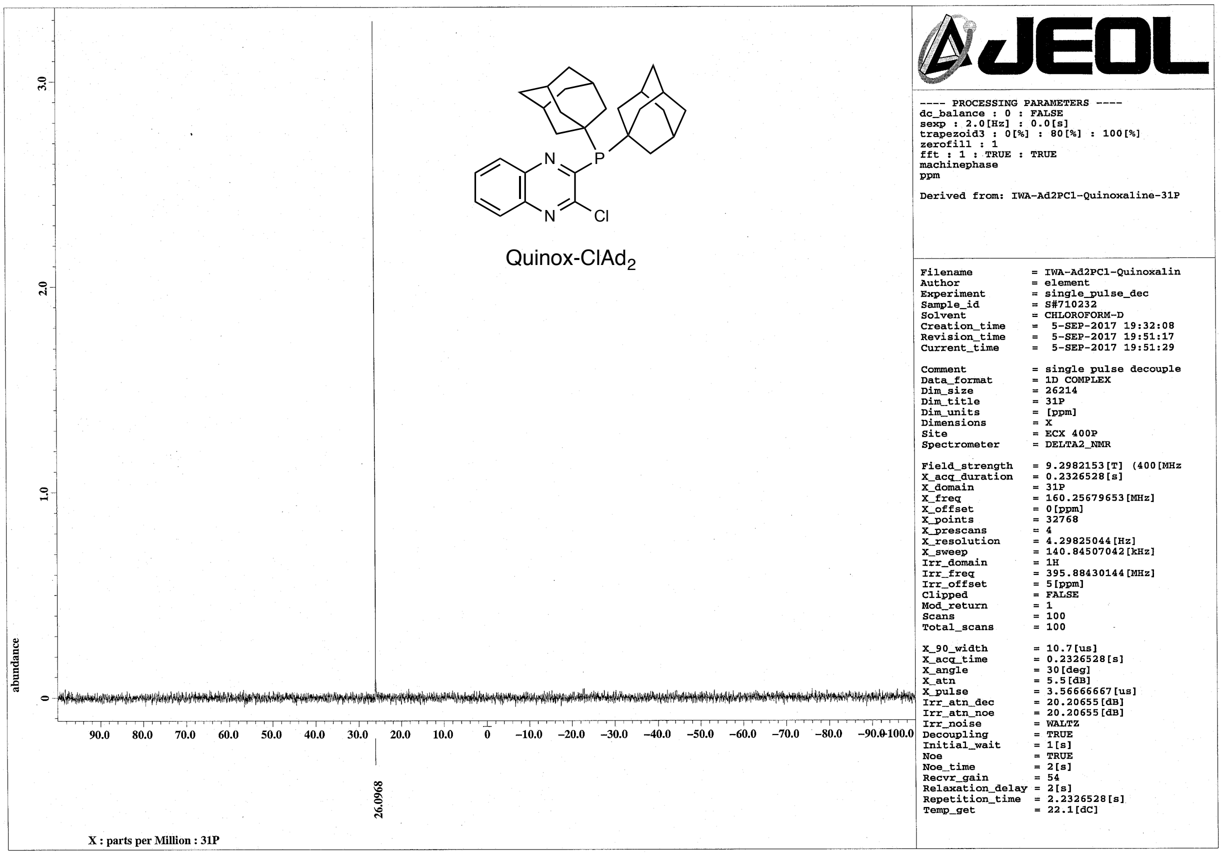

Supplementary Figure 12. ^{31}P NMR spectrum of Quinox-ClAd$_2$.

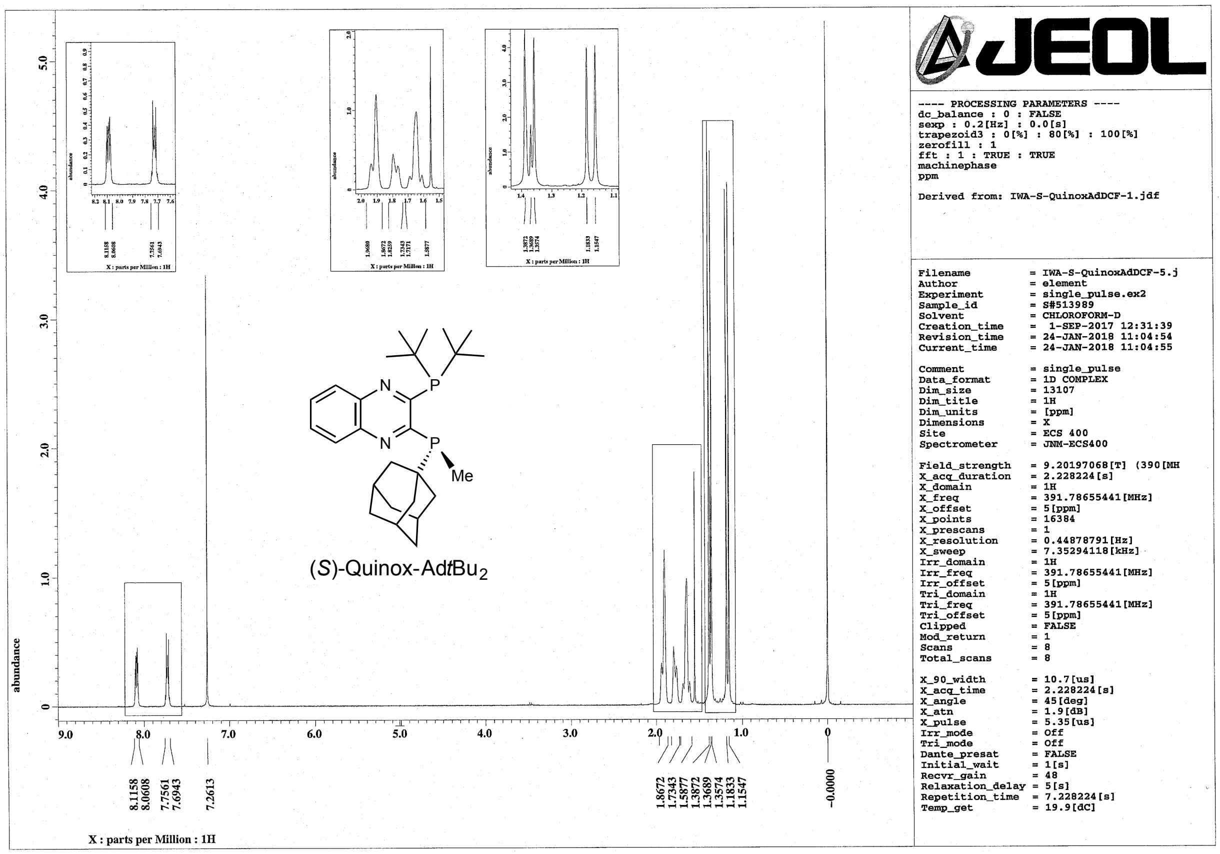

Supplementary Figure 13. ^{1}H NMR spectrum of (*S*)-Quinox-Ad*t*Bu$_2$.

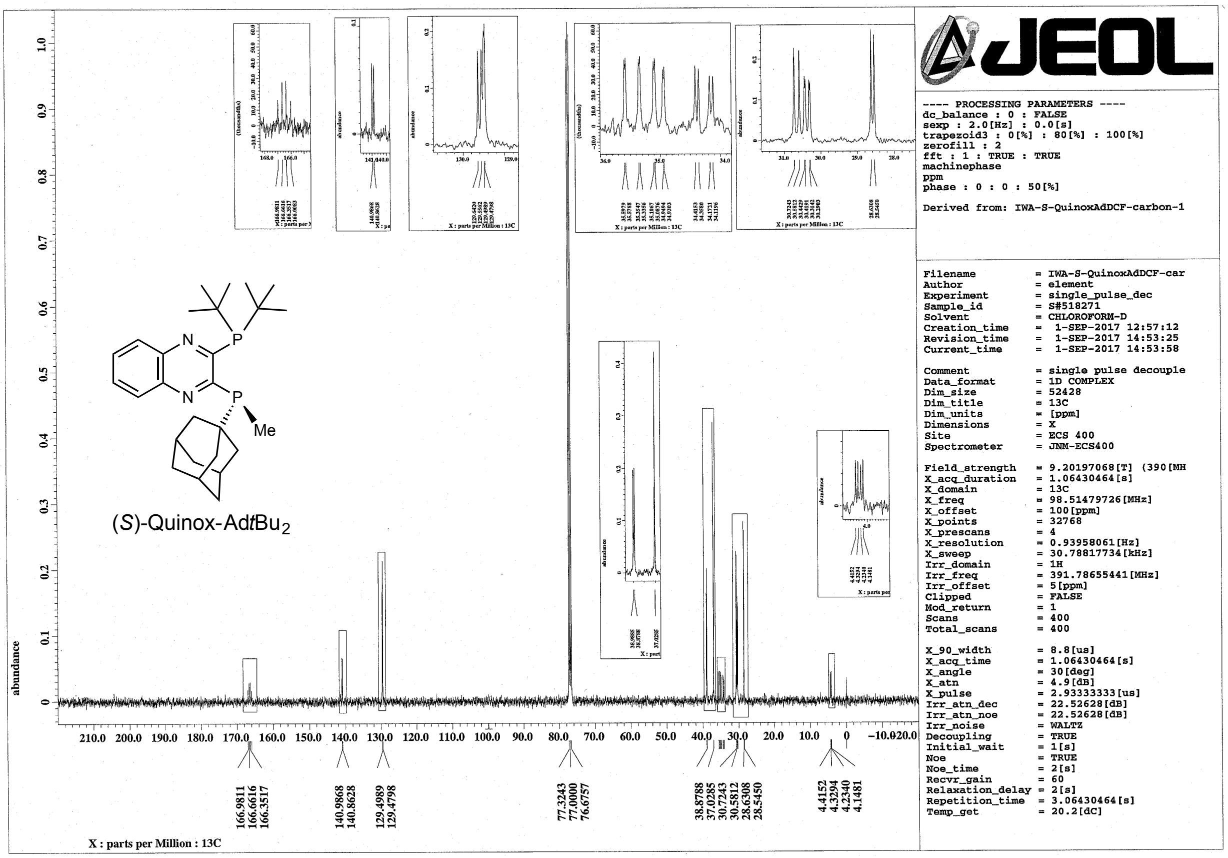

Supplementary Figure 14. ^{13}C NMR spectrum of (*S*)-Quinox-Ad*t*Bu$_2$.

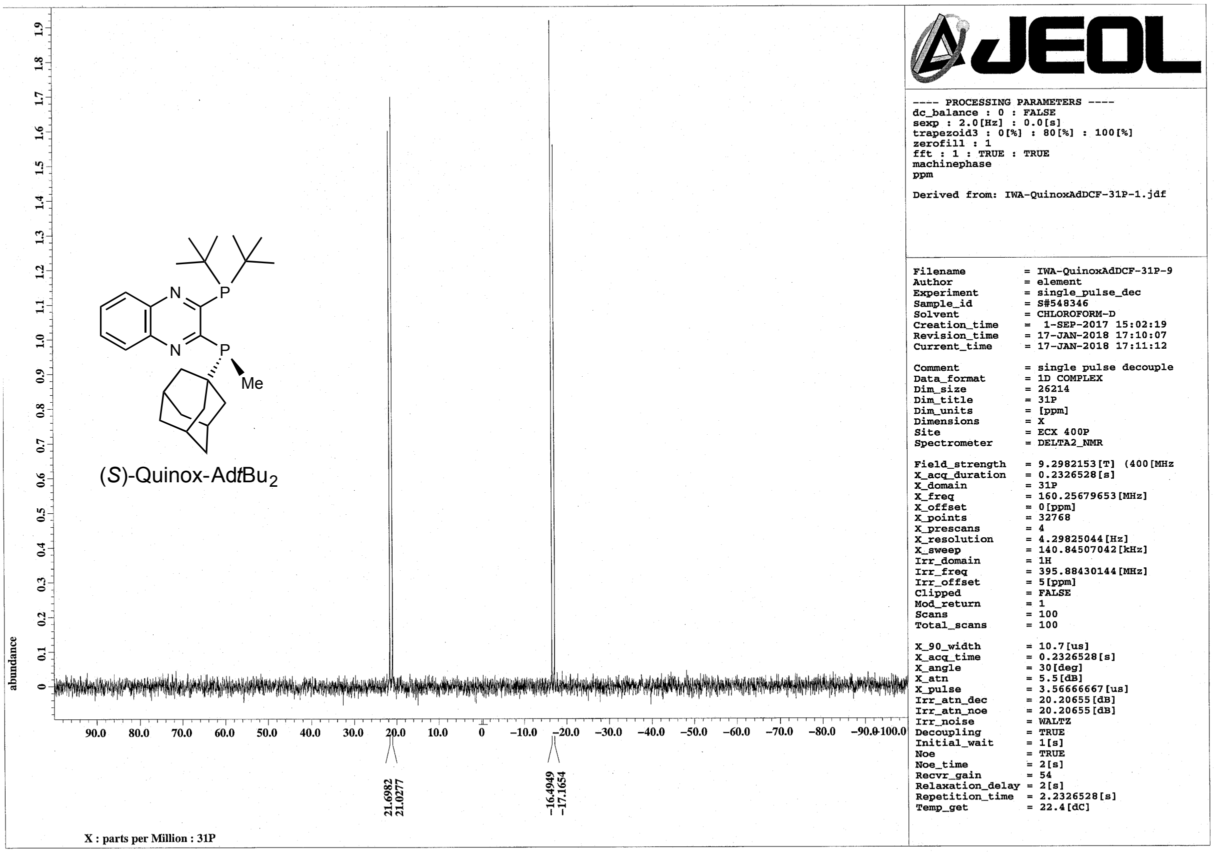

Supplementary Figure 15. ^{31}P NMR spectrum of (*S*)-Quinox-Ad*t*Bu$_2$.

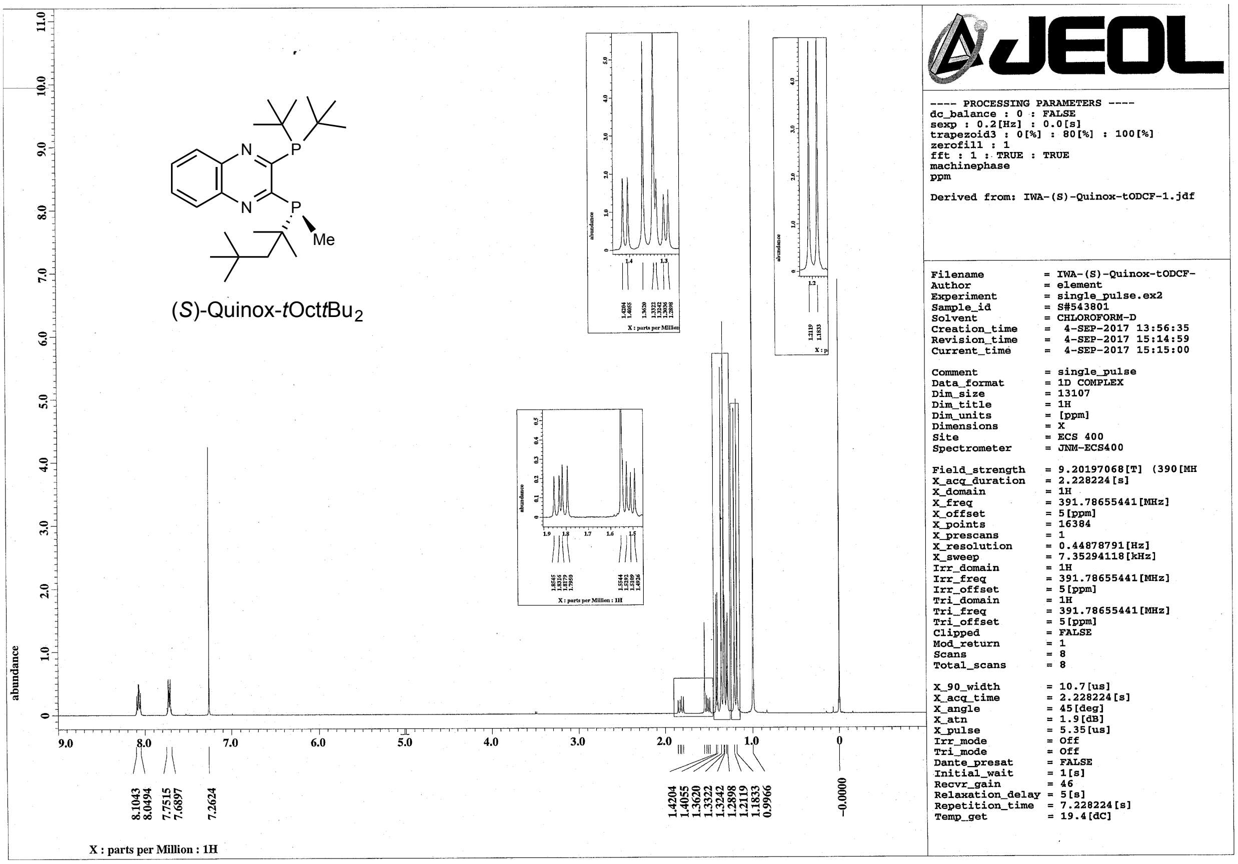

Supplementary Figure 16. ^{1}H NMR spectrum of (*S*)-Quinox-*t*Oct*t*Bu$_2$.

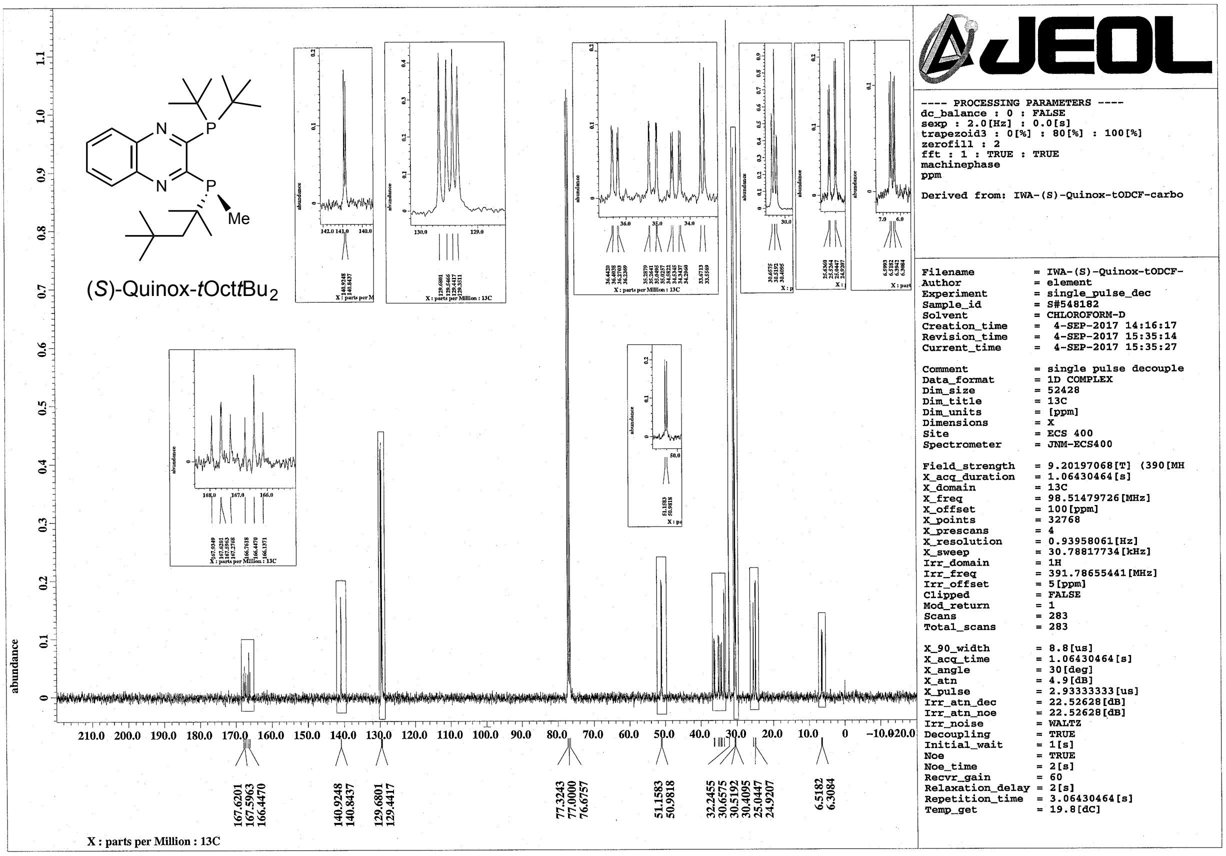

Supplementary Figure 17. ^{13}C NMR spectrum of (*S*)-Quinox-*t*Oct*t*Bu$_2$.

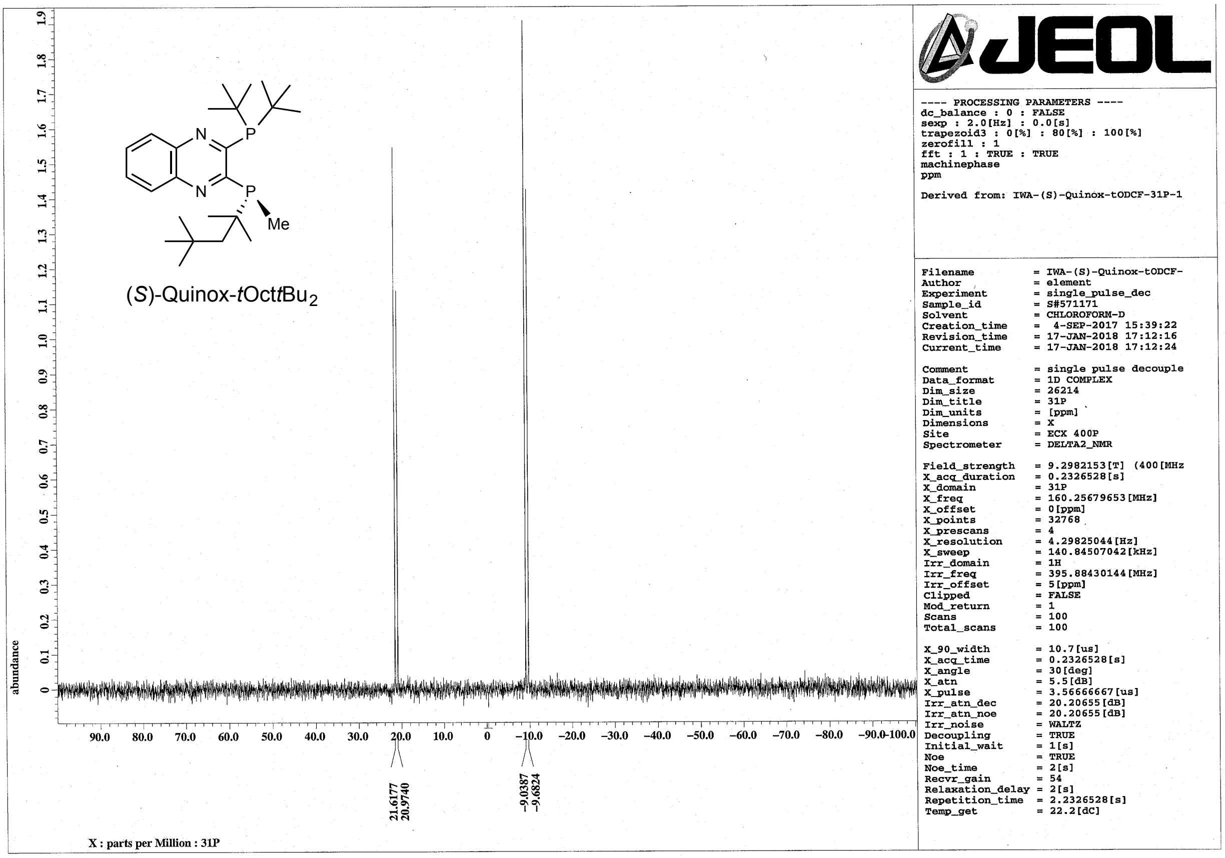

Supplementary Figure 18. ^{31}P NMR spectrum of (*S*)-Quinox-*t*Oct*t*Bu$_2$.

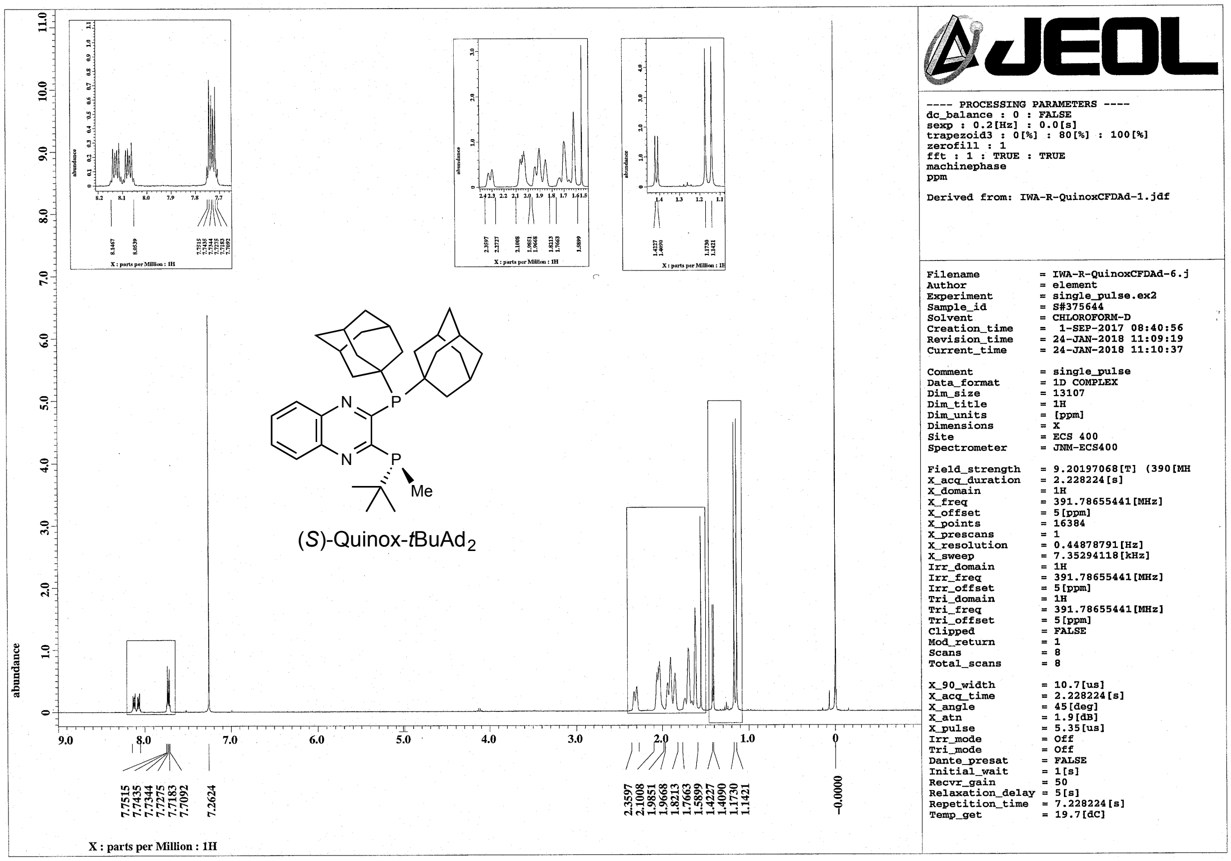

Supplementary Figure 19. ^{1}H NMR spectrum of (*S*)-Quinox-*t*BuAd$_2$.

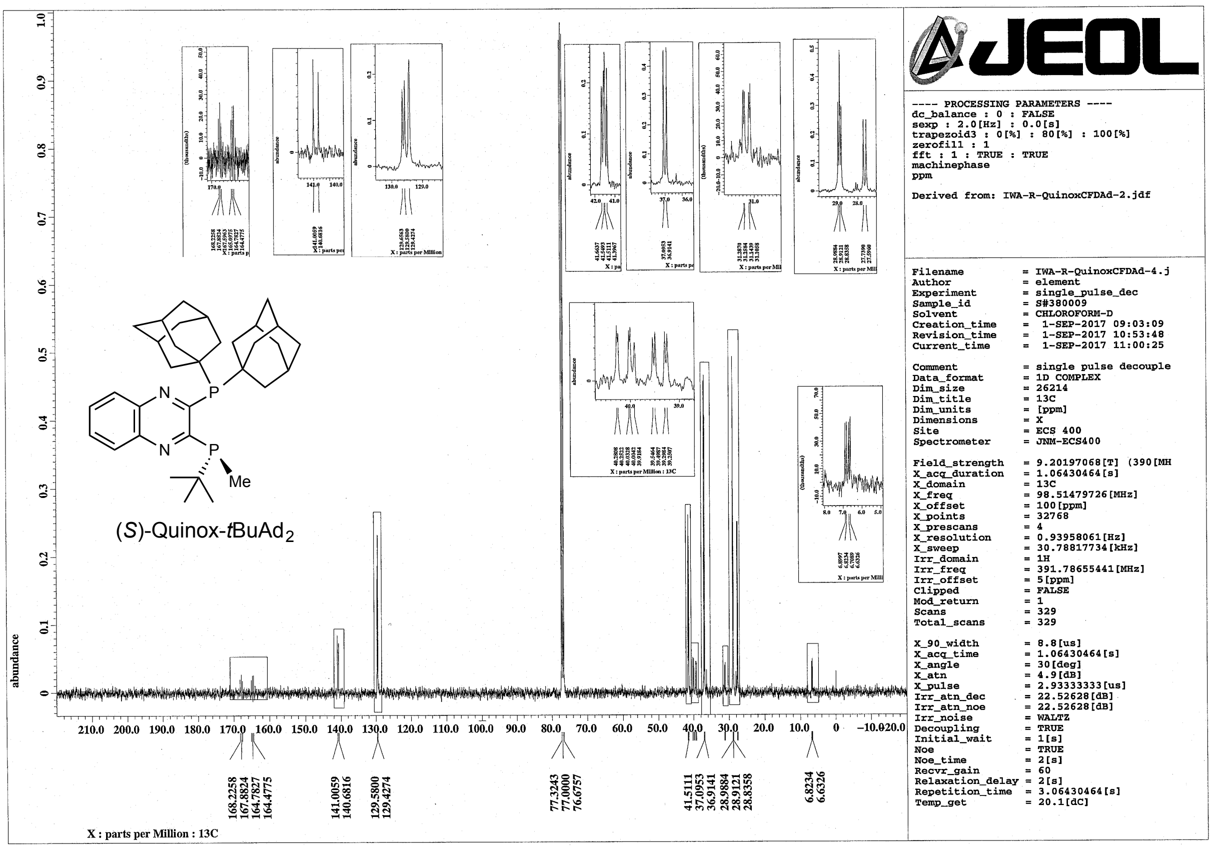

Supplementary Figure 20. ^{13}C NMR spectrum of (*S*)-Quinox-*t*BuAd$_2$.

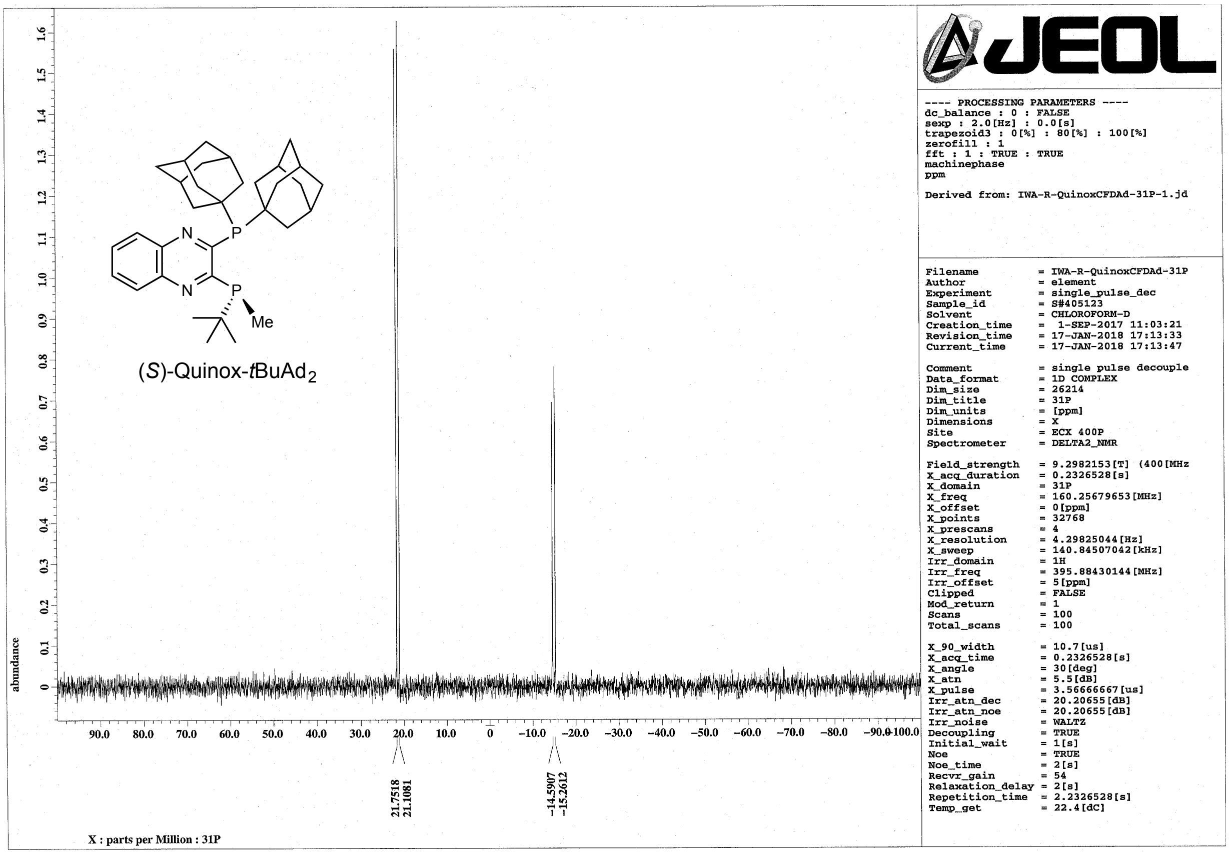

Supplementary Figure 21. ^{31}P NMR spectrum of (*S*)-Quinox-*t*BuAd$_2$.

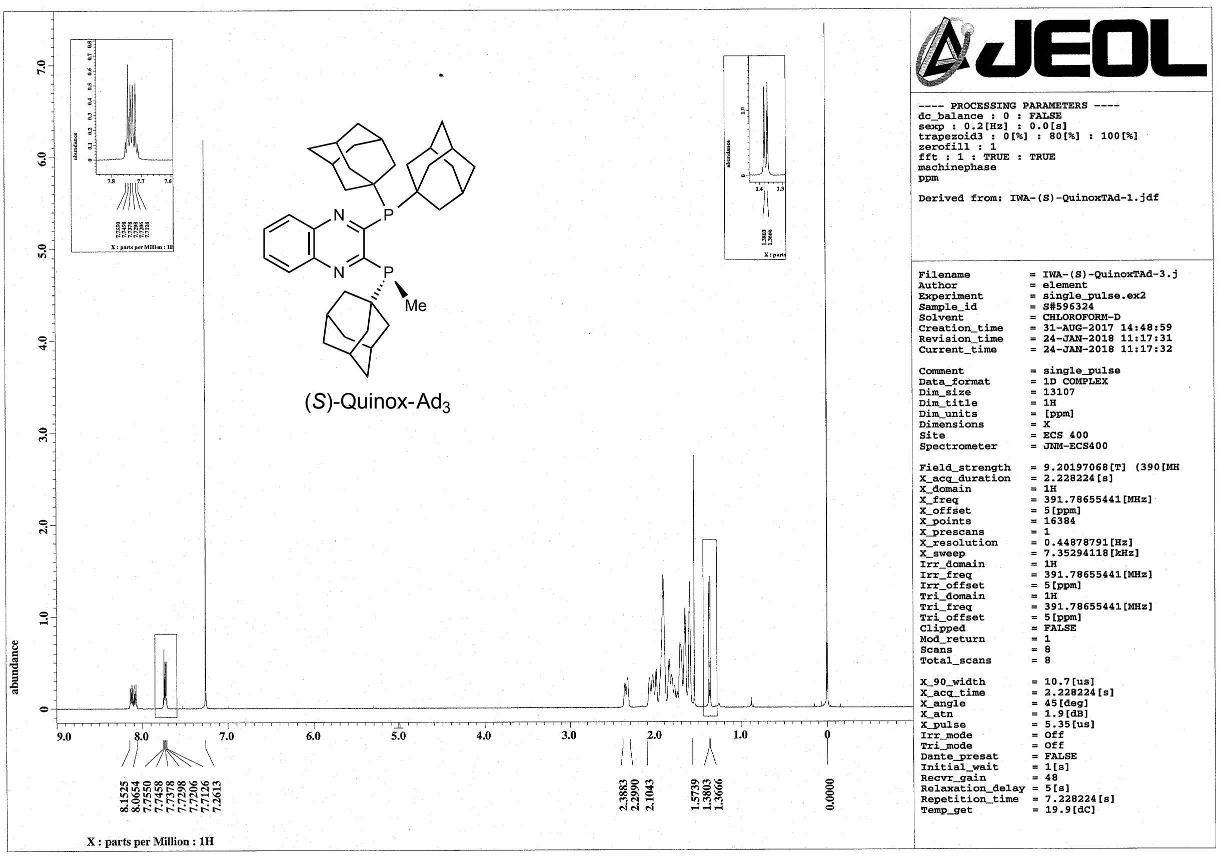

Supplementary Figure 22. ^{1}H NMR spectrum of (*S*)-Quinox-Ad$_3$.

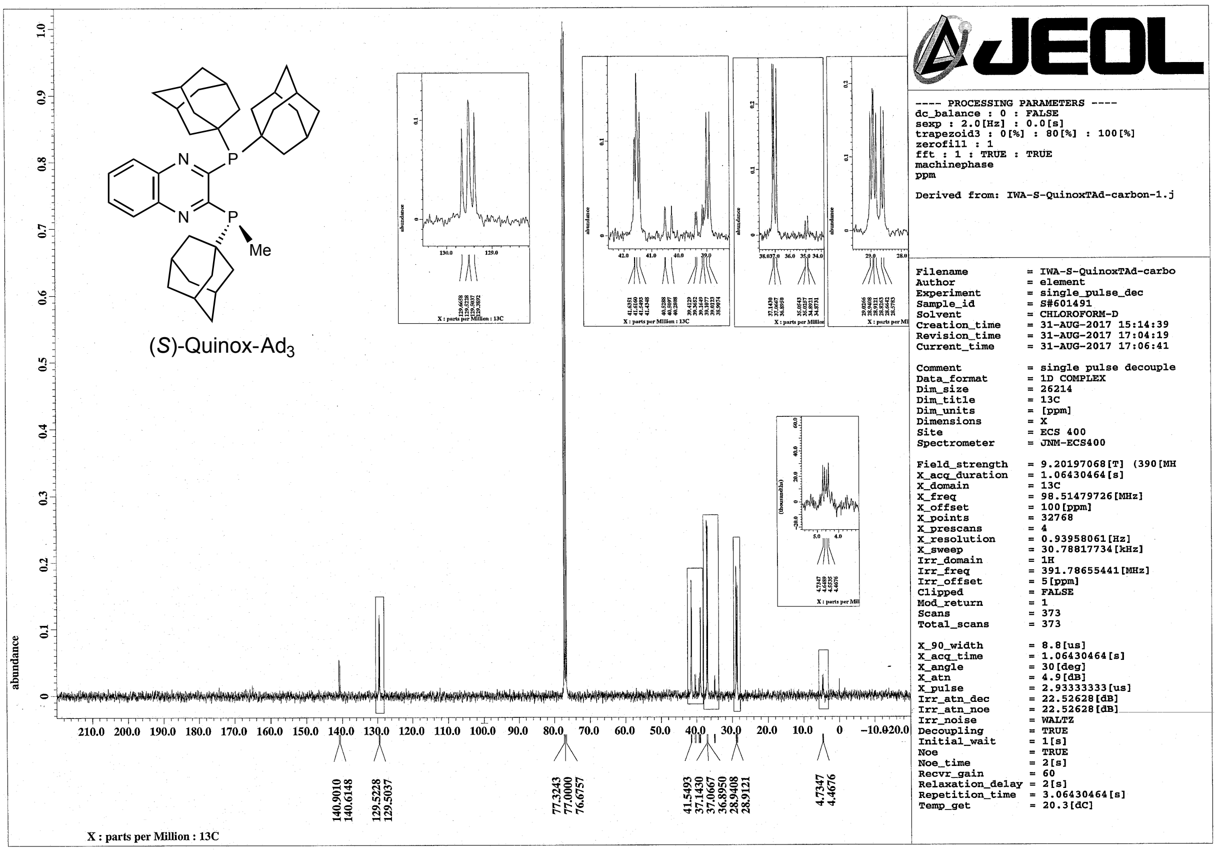

Supplementary Figure 23. ^{13}C NMR spectrum of (*S*)-Quinox-Ad$_3$.

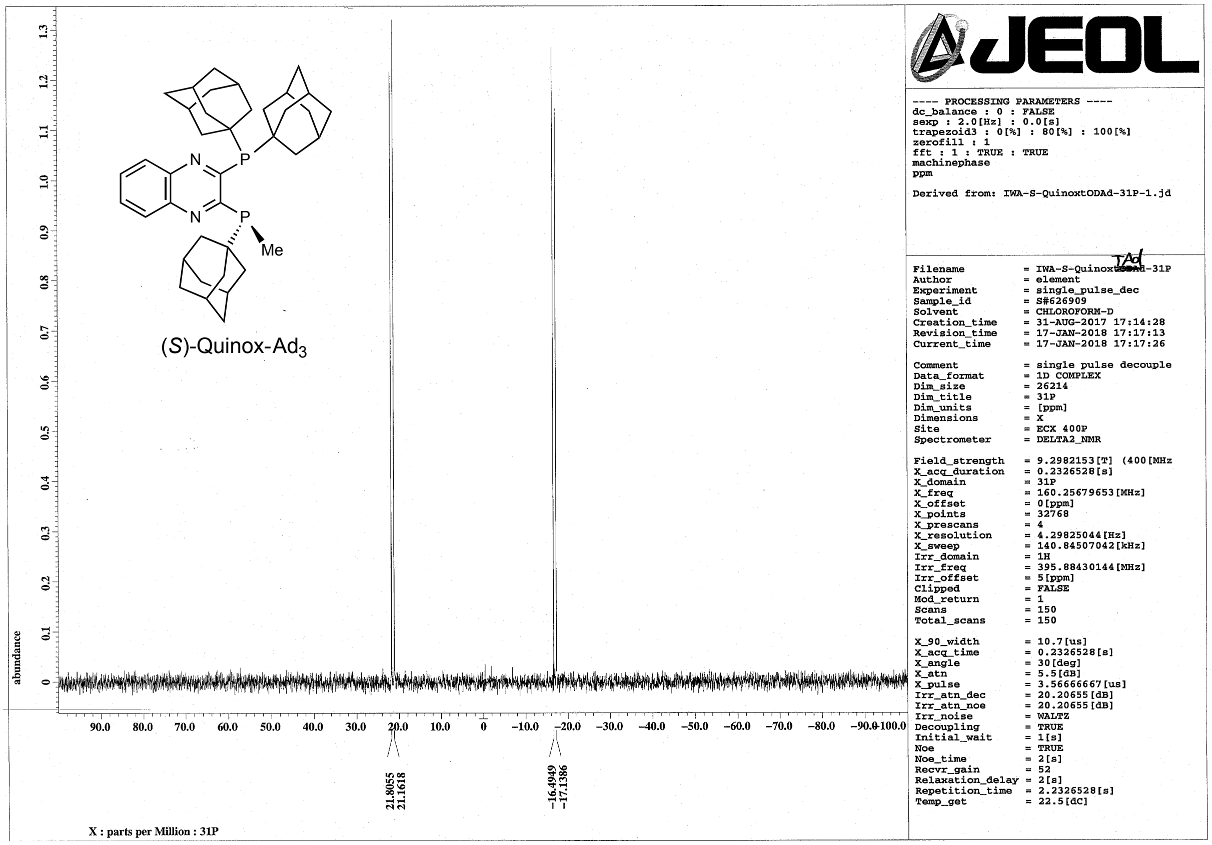

Supplementary Figure 24. ^{31}P NMR spectrum of (*S*)-Quinox-Ad$_3$.

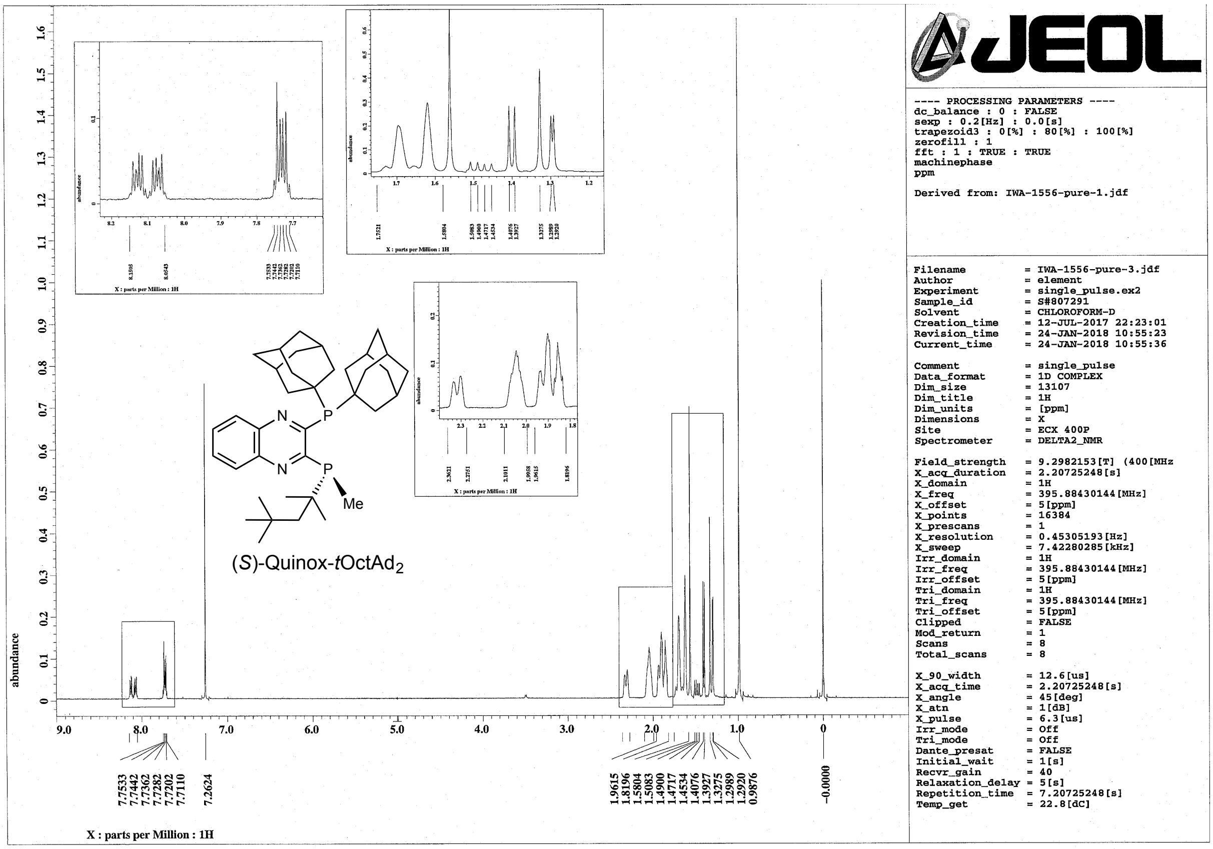

Supplementary Figure 25. ^{1}H NMR spectrum of (*S*)-Quinox-*t*OctAd$_2$.

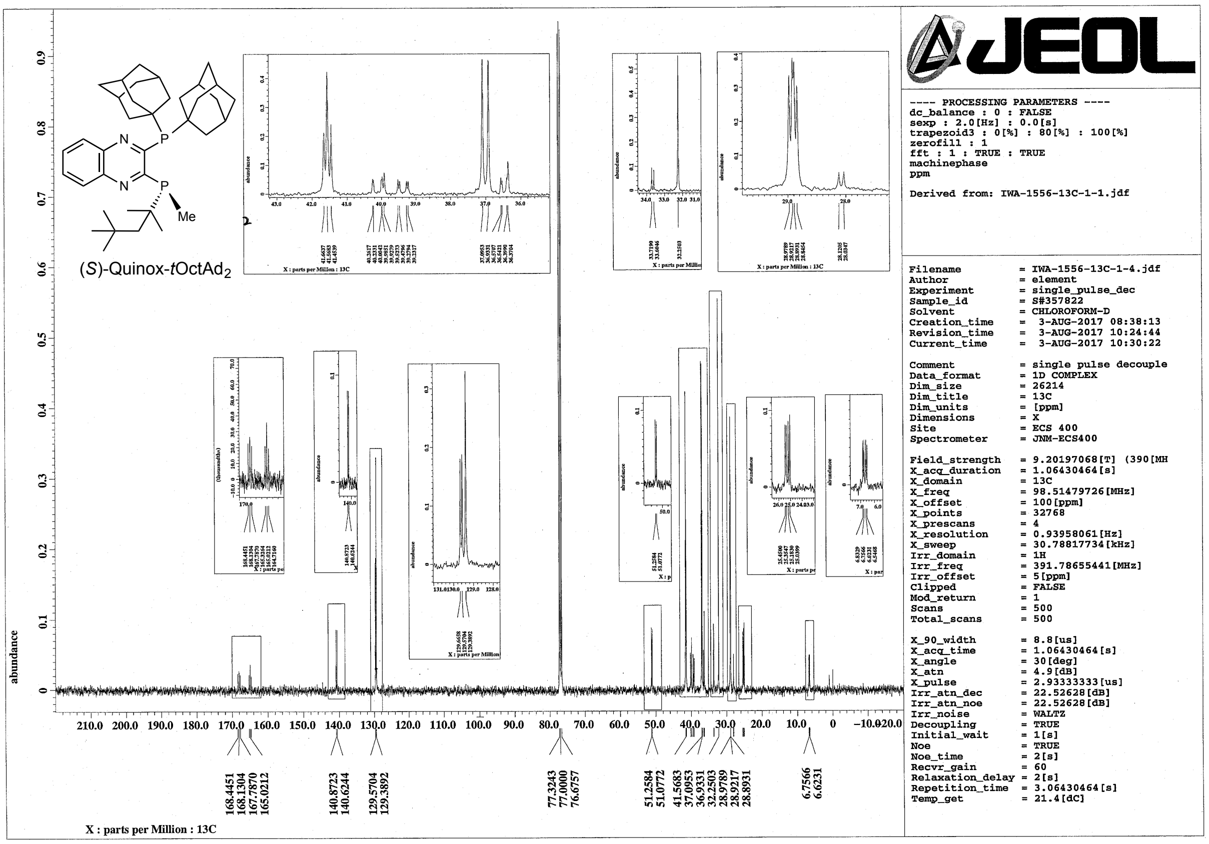

Supplementary Figure 26. ^{13}C NMR spectrum of (*S*)-Quinox-*t*OctAd$_2$.

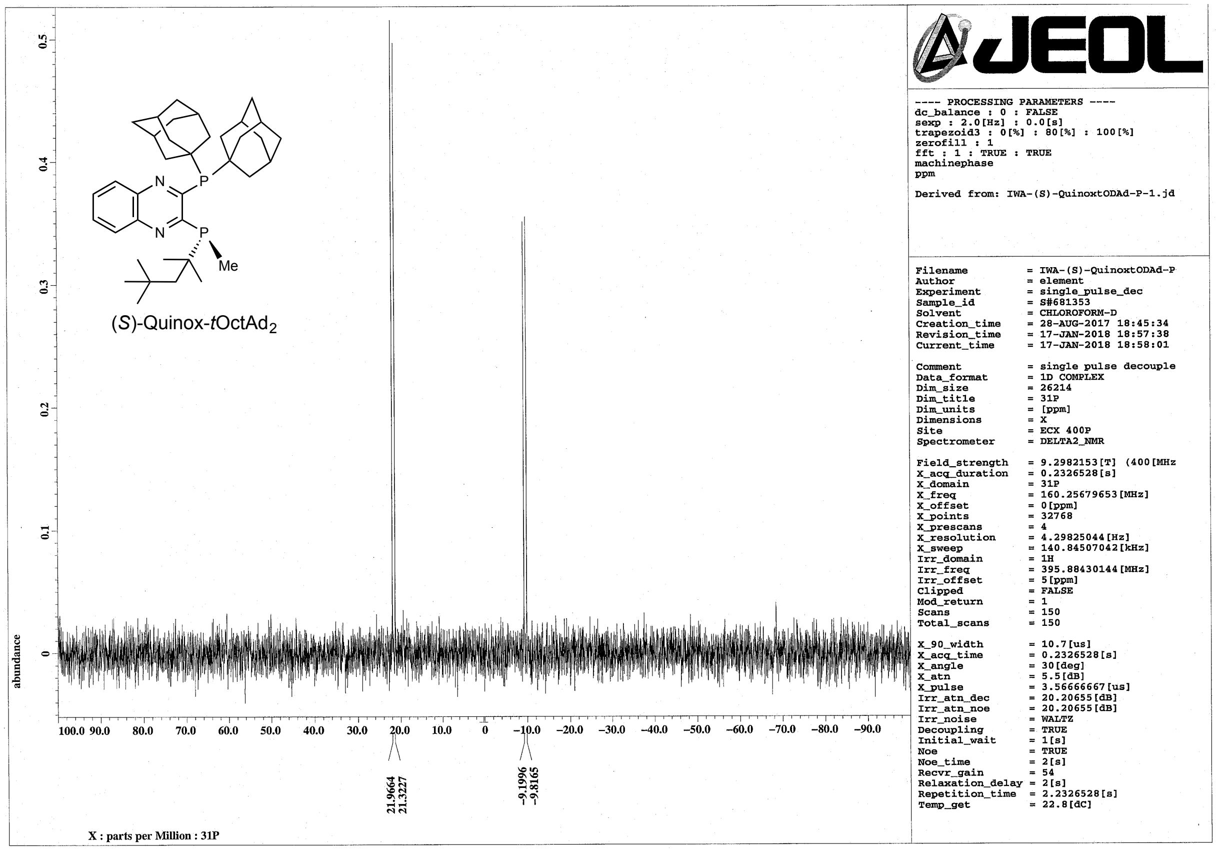

Supplementary Figure 27. ^{31}P NMR spectrum of (*S*)-Quinox-*t*OctAd$_2$.

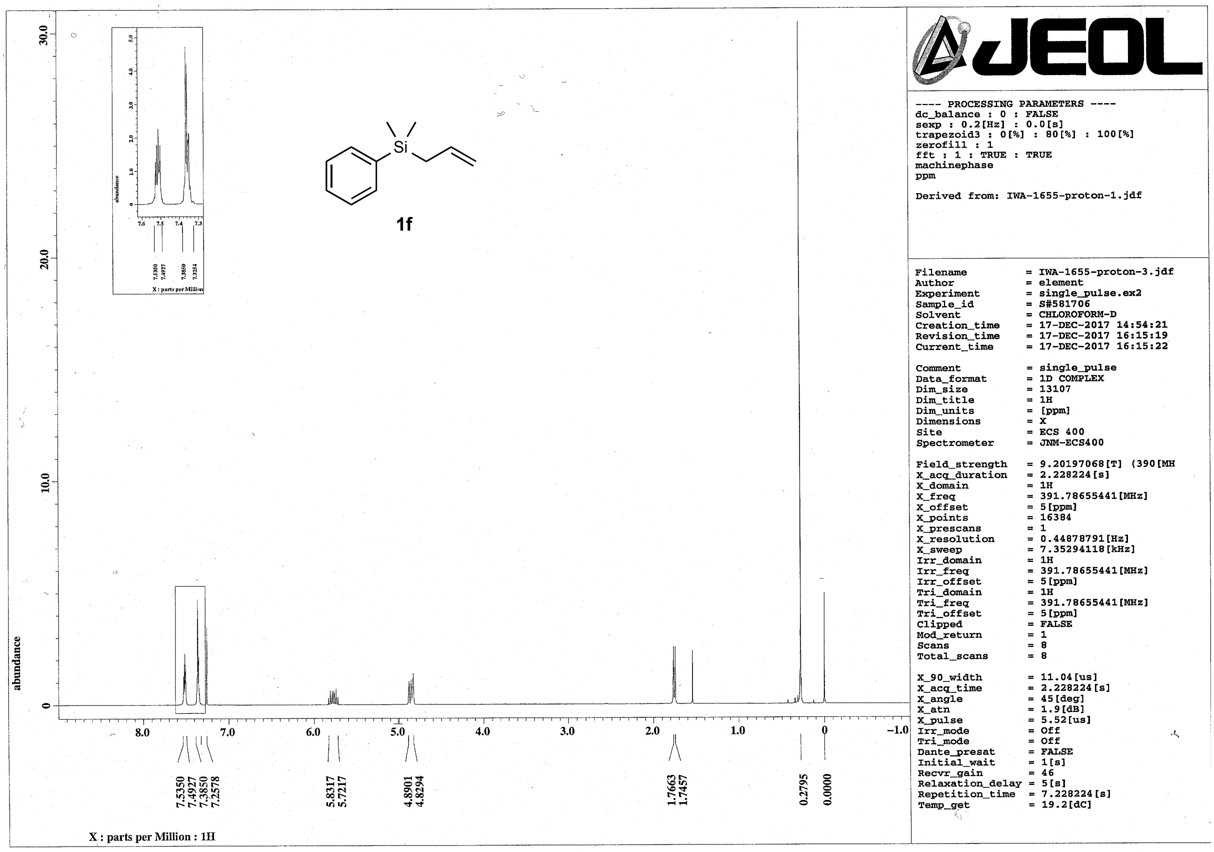

Supplementary Figure 28. ^{1}H NMR spectrum of **1f**.

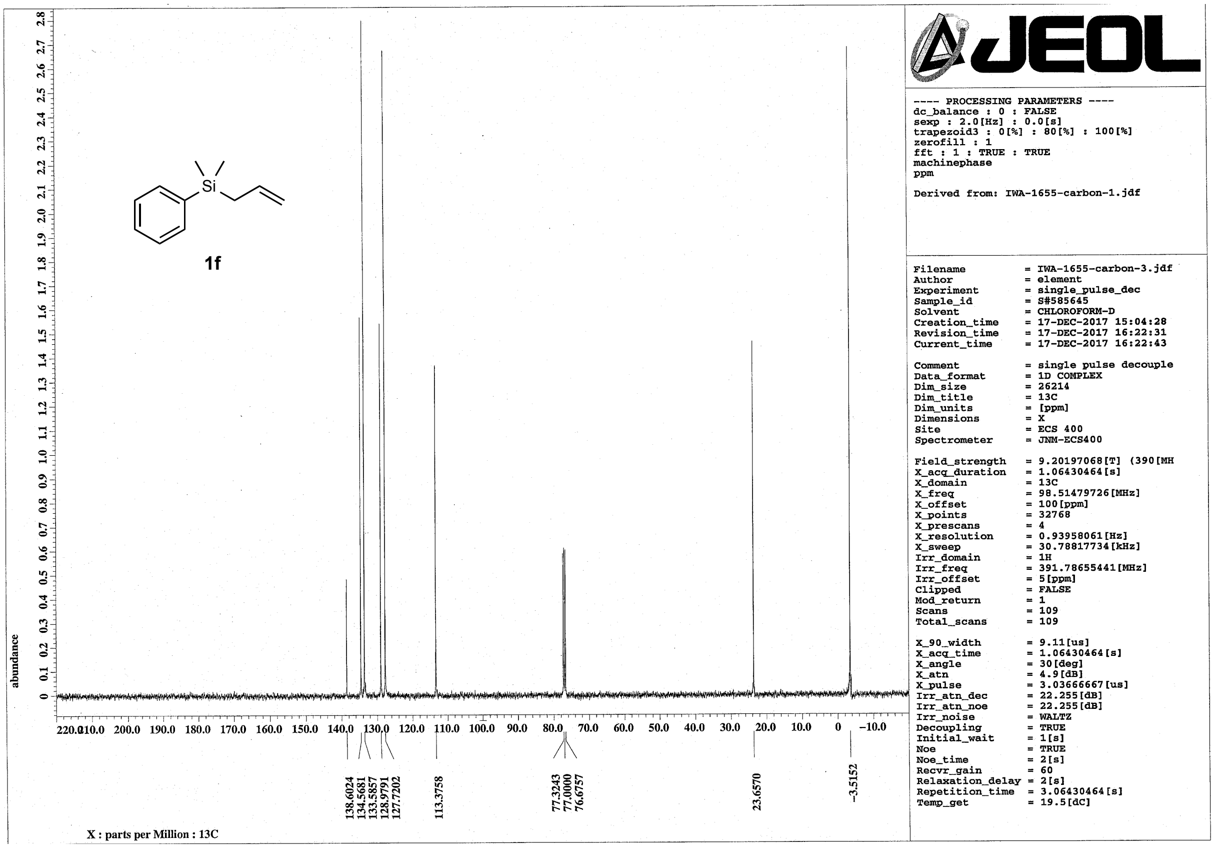

Supplementary Figure 29. ^{13}C NMR spectrum of **1f**.

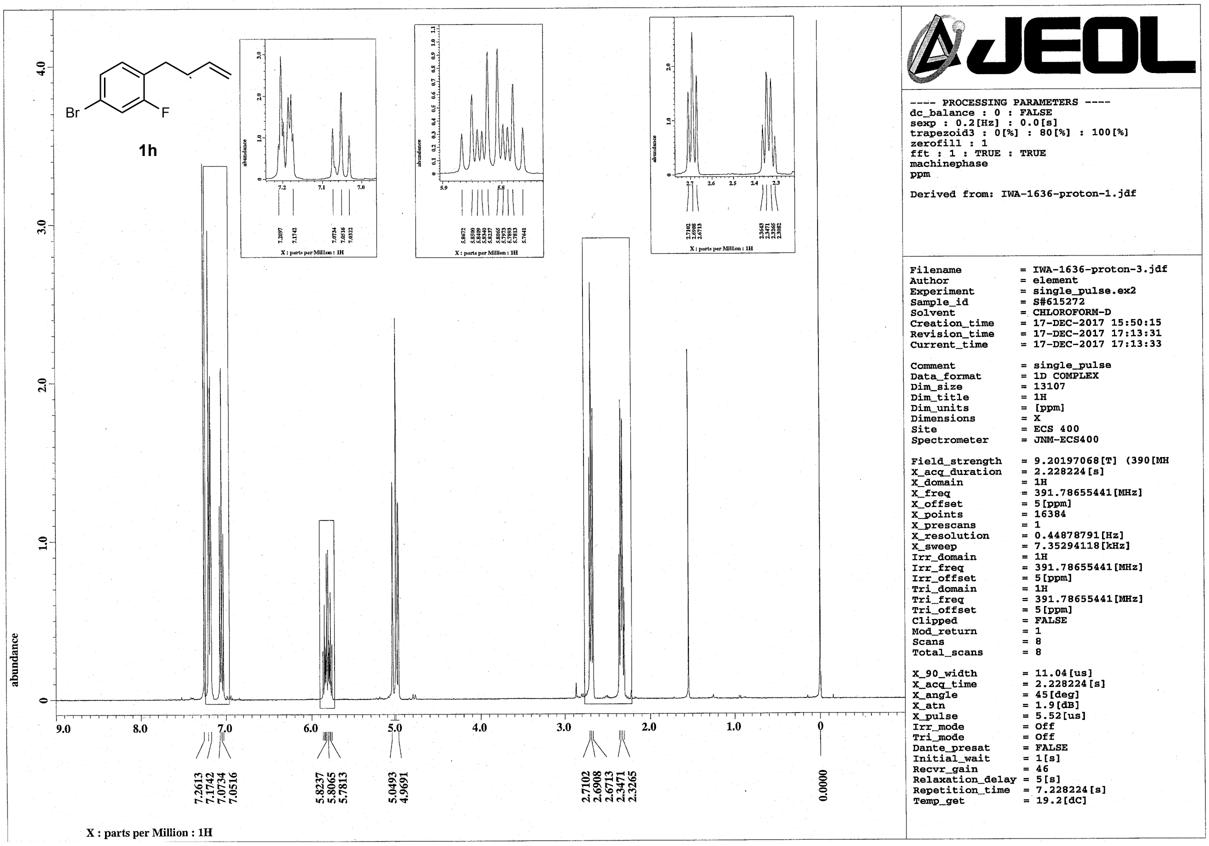

Supplementary Figure 30. ^{1}H NMR spectrum of **1h**.

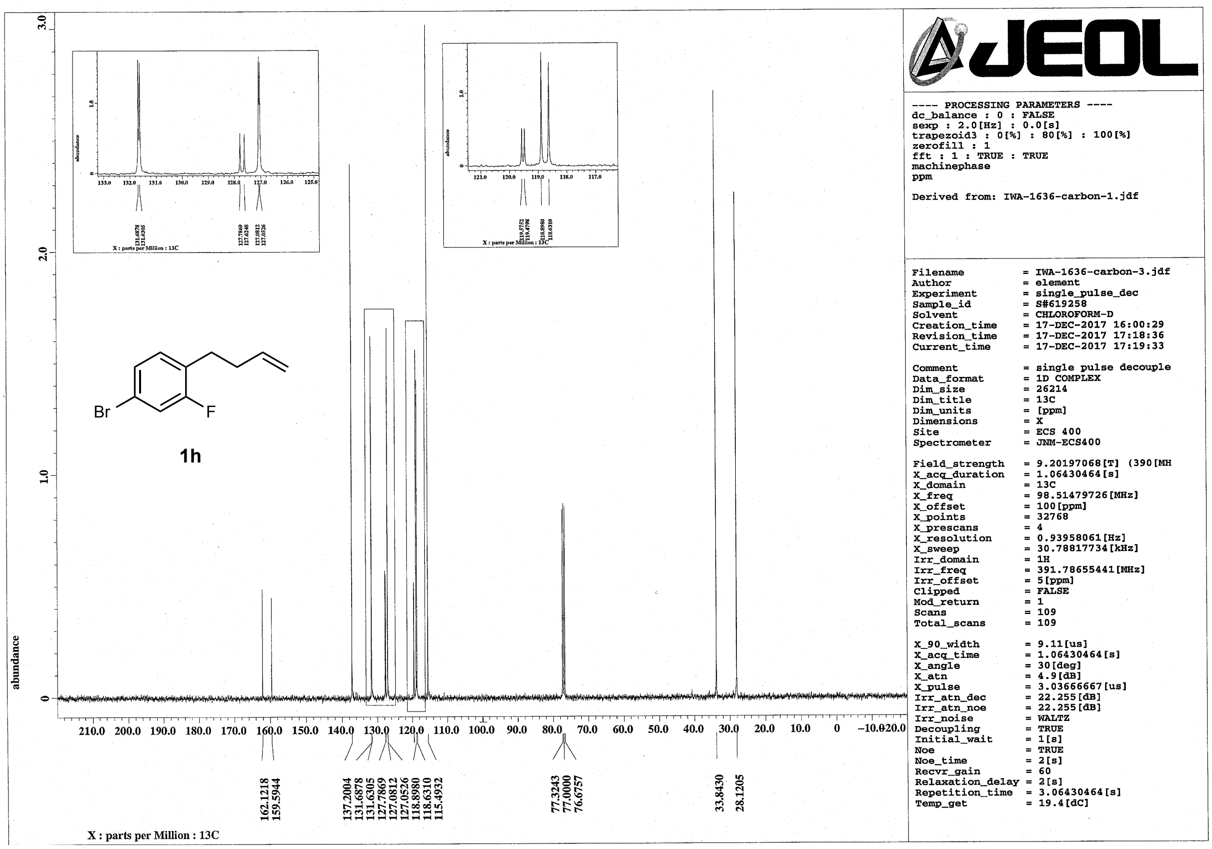

Supplementary Figure 31. ^{13}C NMR spectrum of **1h**.

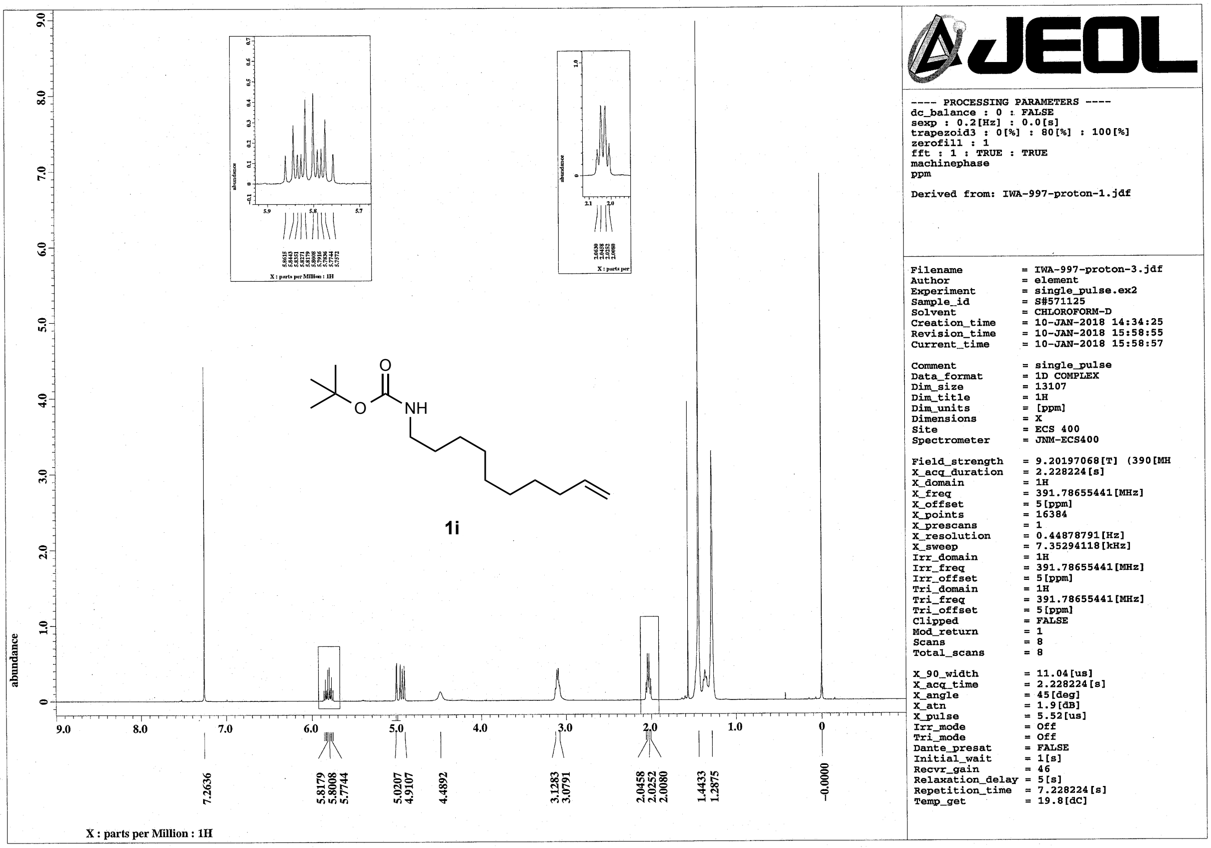

Supplementary Figure 32. ^{1}H NMR spectrum of **1i**.

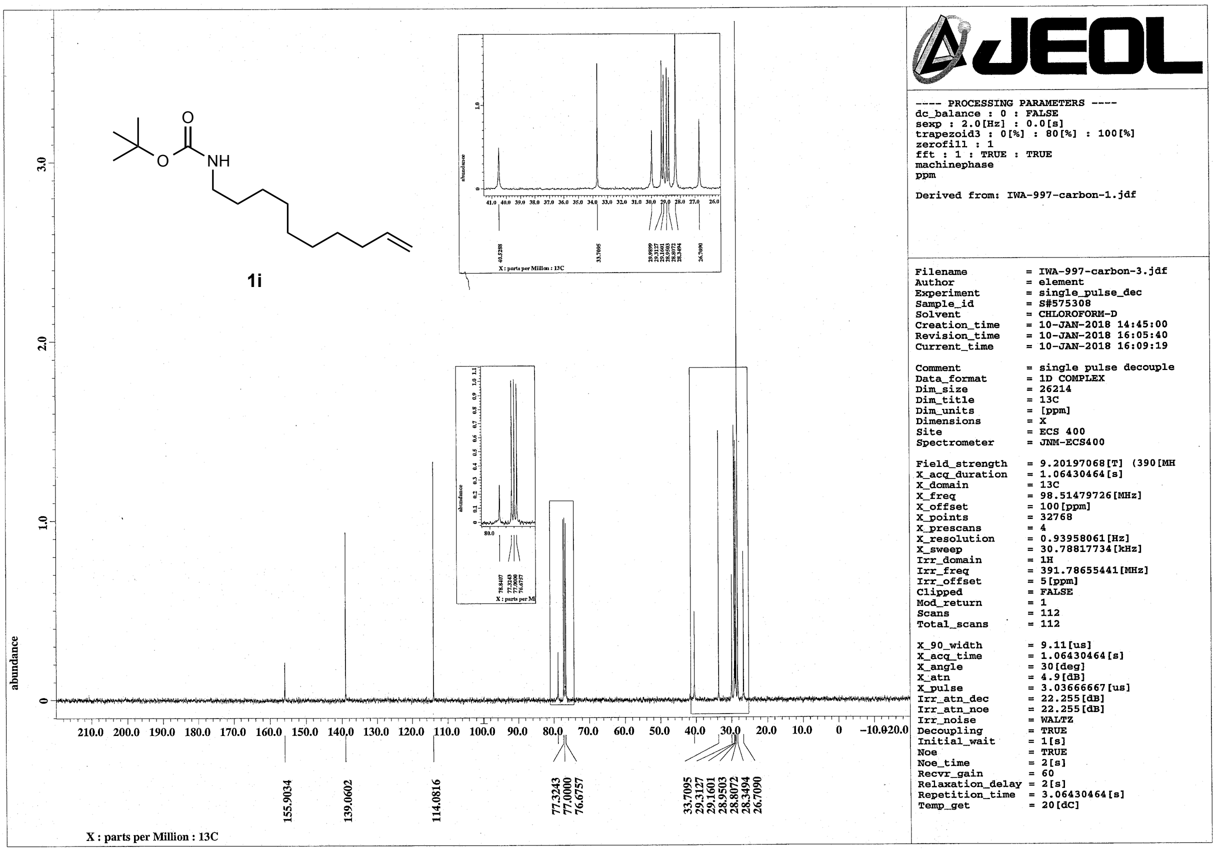

Supplementary Figure 33. ^{13}C NMR spectrum of **1i**.

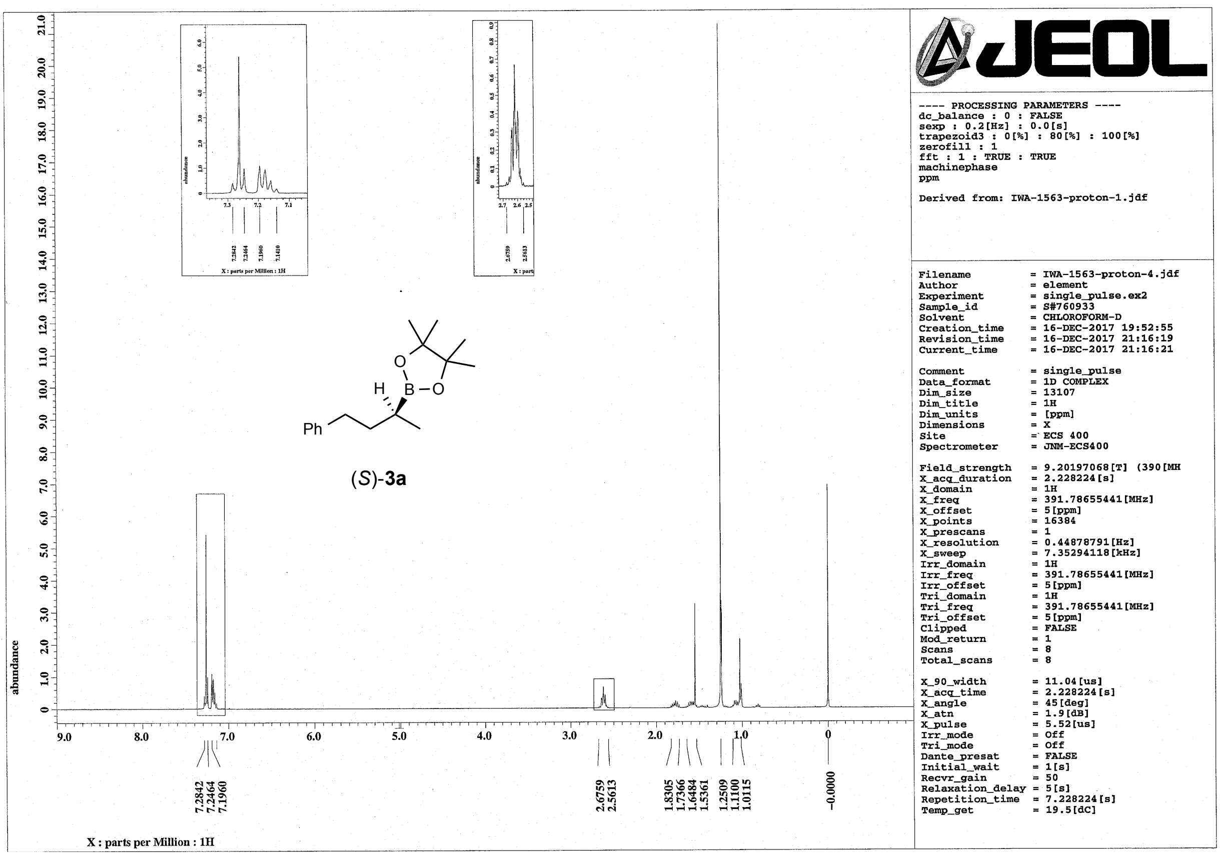

Supplementary Figure 34. ^{1}H NMR spectrum of (*S*)-**3a**.

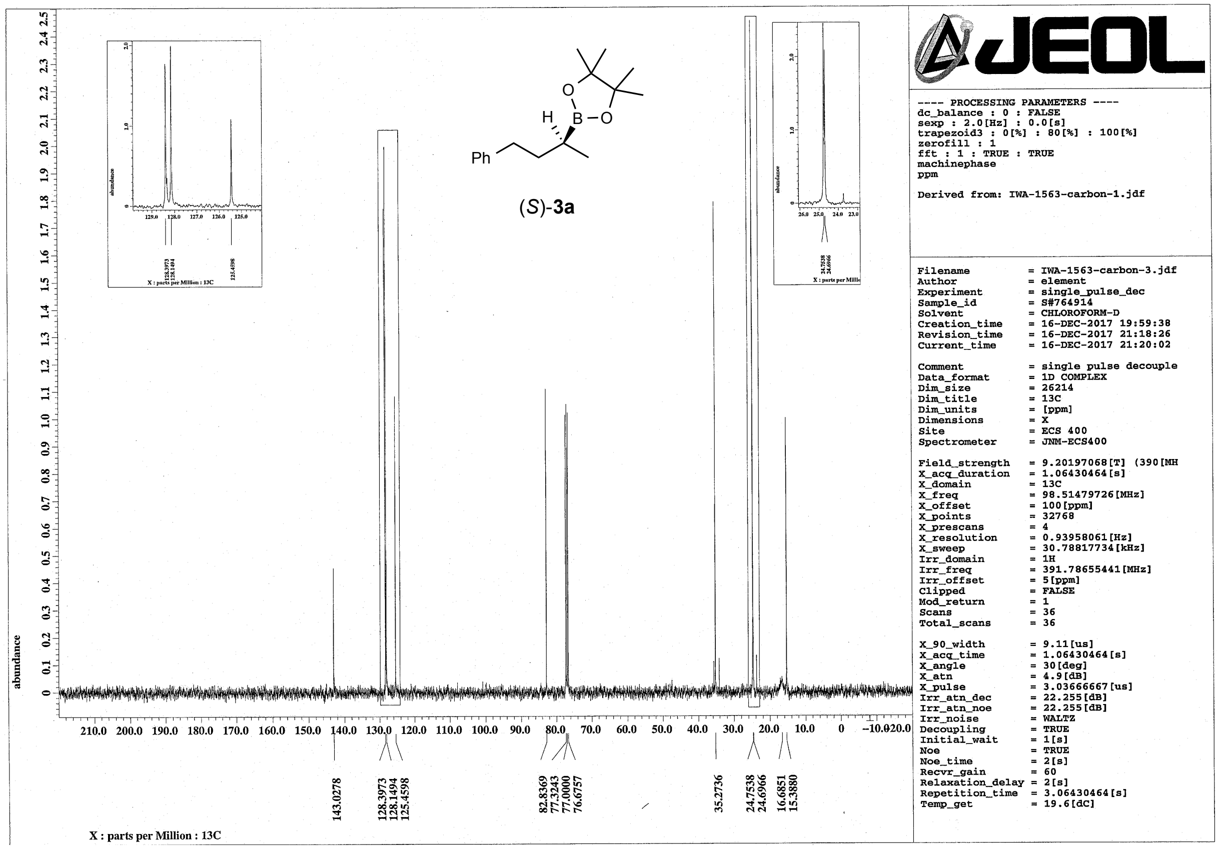

Supplementary Figure 35. ^{13}C NMR spectrum of (*S*)-**3a**.

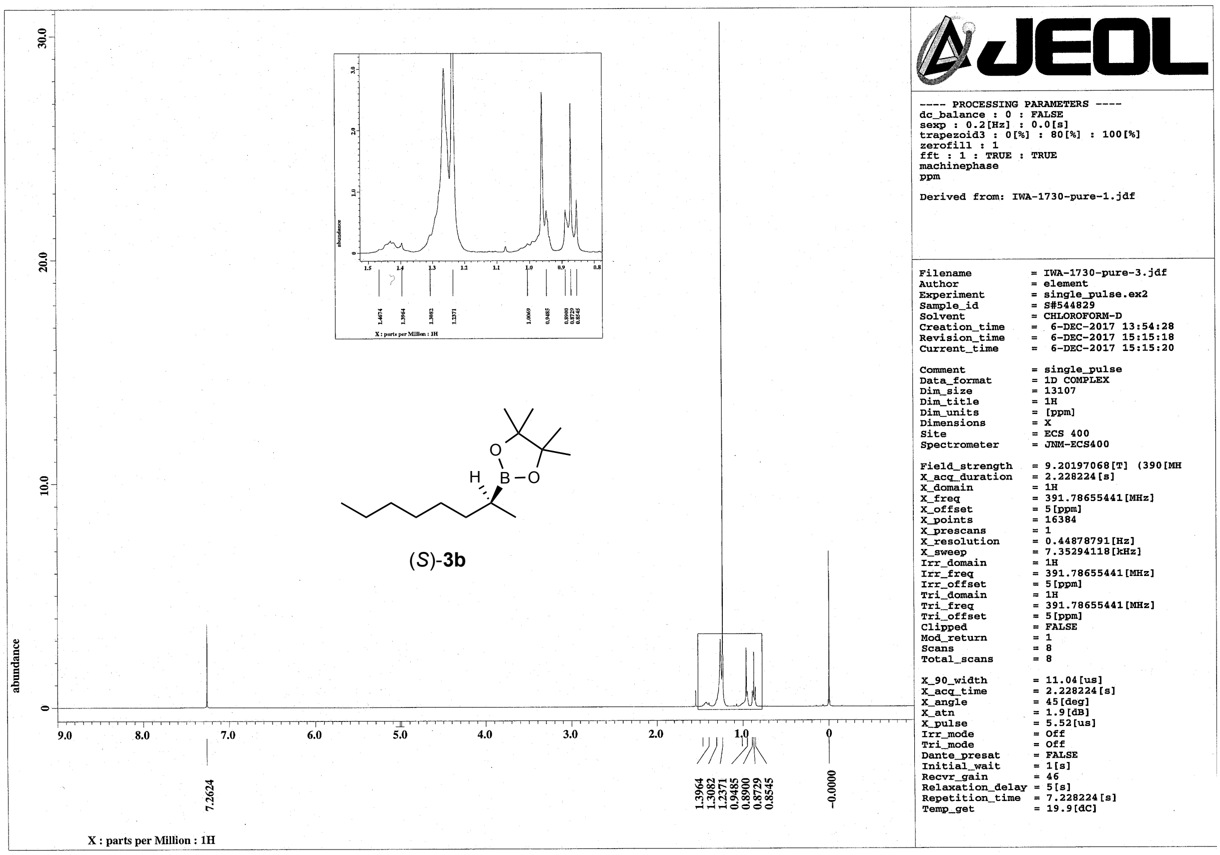

Supplementary Figure 36. ^{1}H NMR spectrum of (*S*)-**3b**.

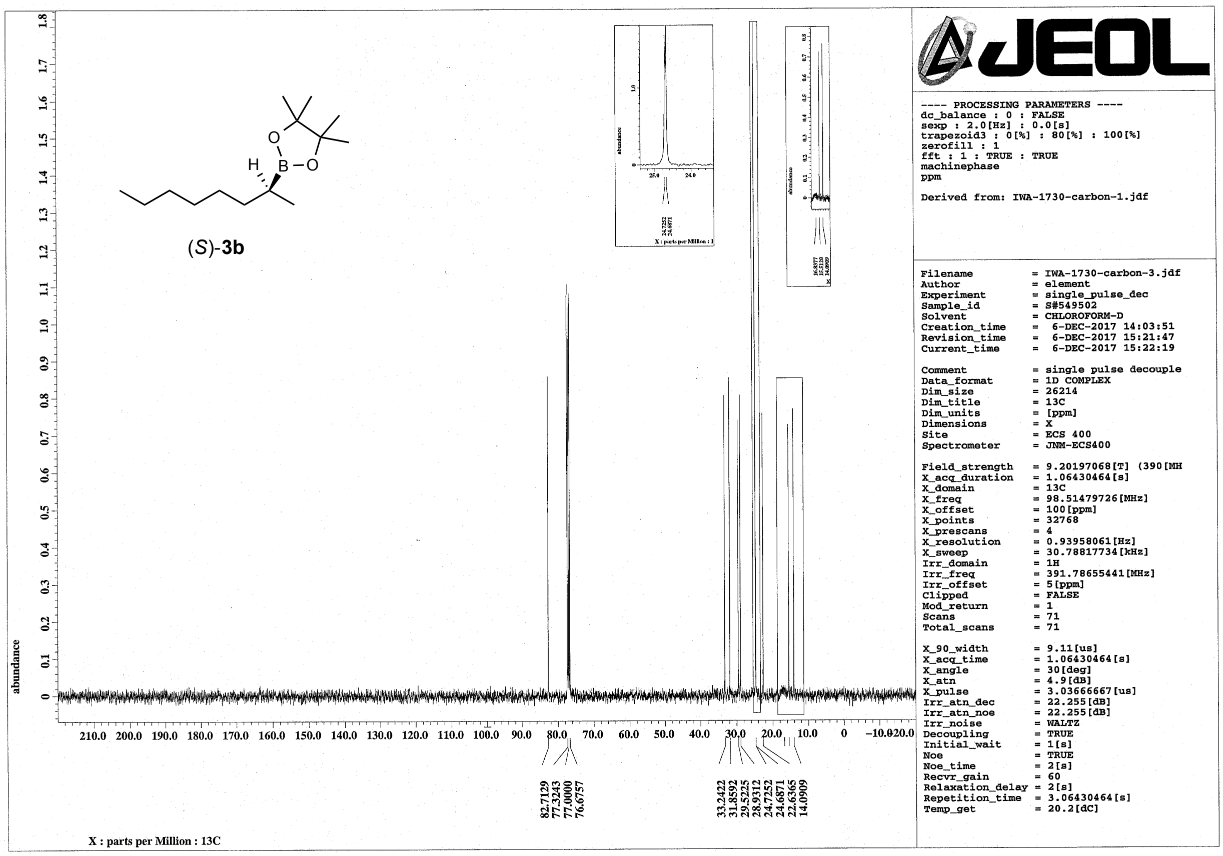

Supplementary Figure 37. ^{13}C NMR spectrum of (*S*)-**3b**.

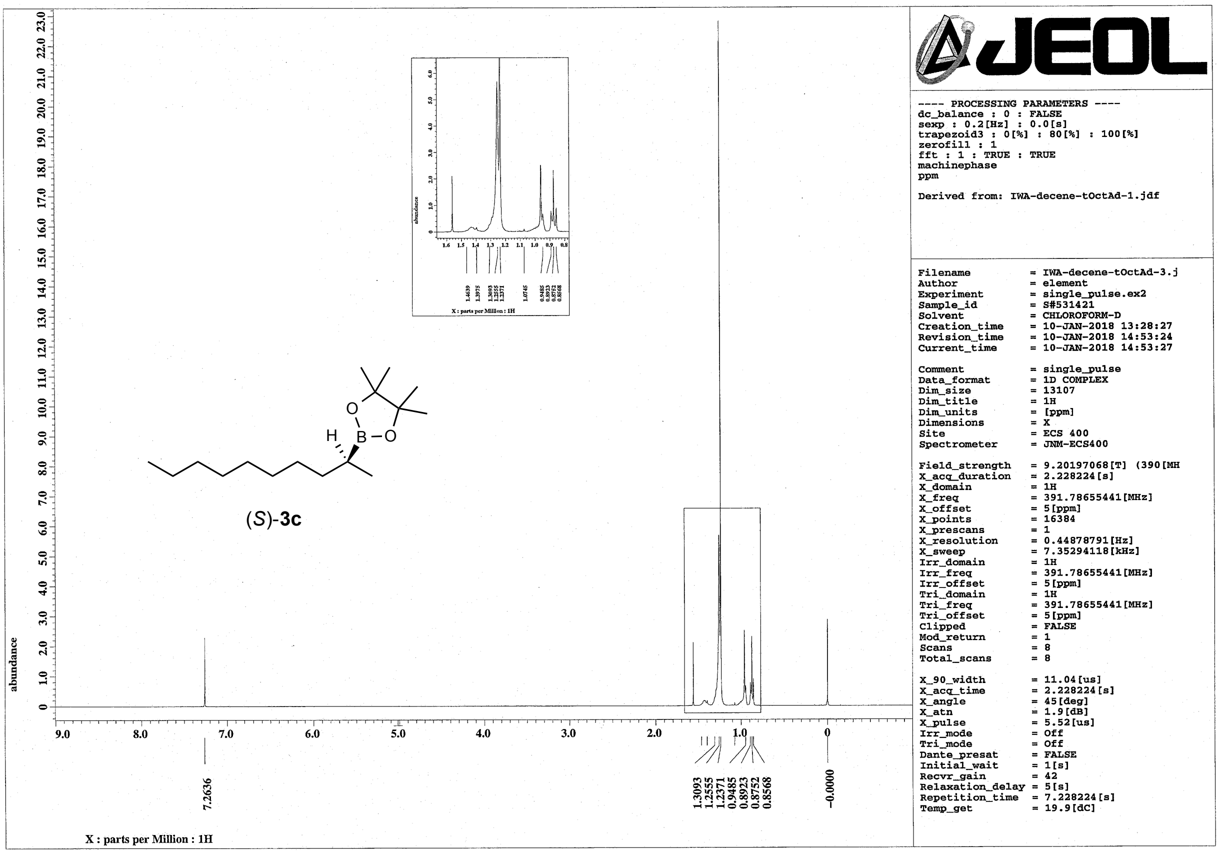

Supplementary Figure 38. ^{1}H NMR spectrum of (*S*)-**3c**.

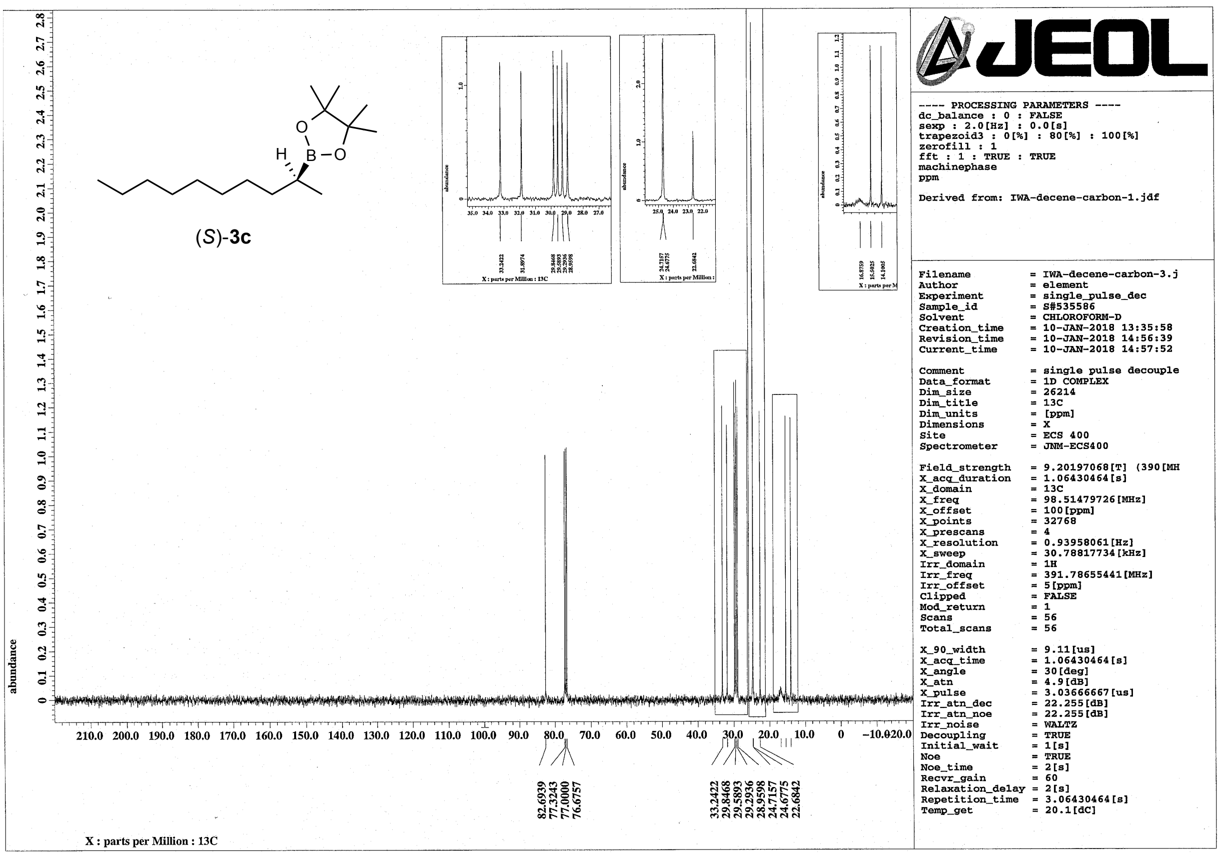

Supplementary Figure 39. ^{13}C NMR spectrum of (*S*)-**3c**.

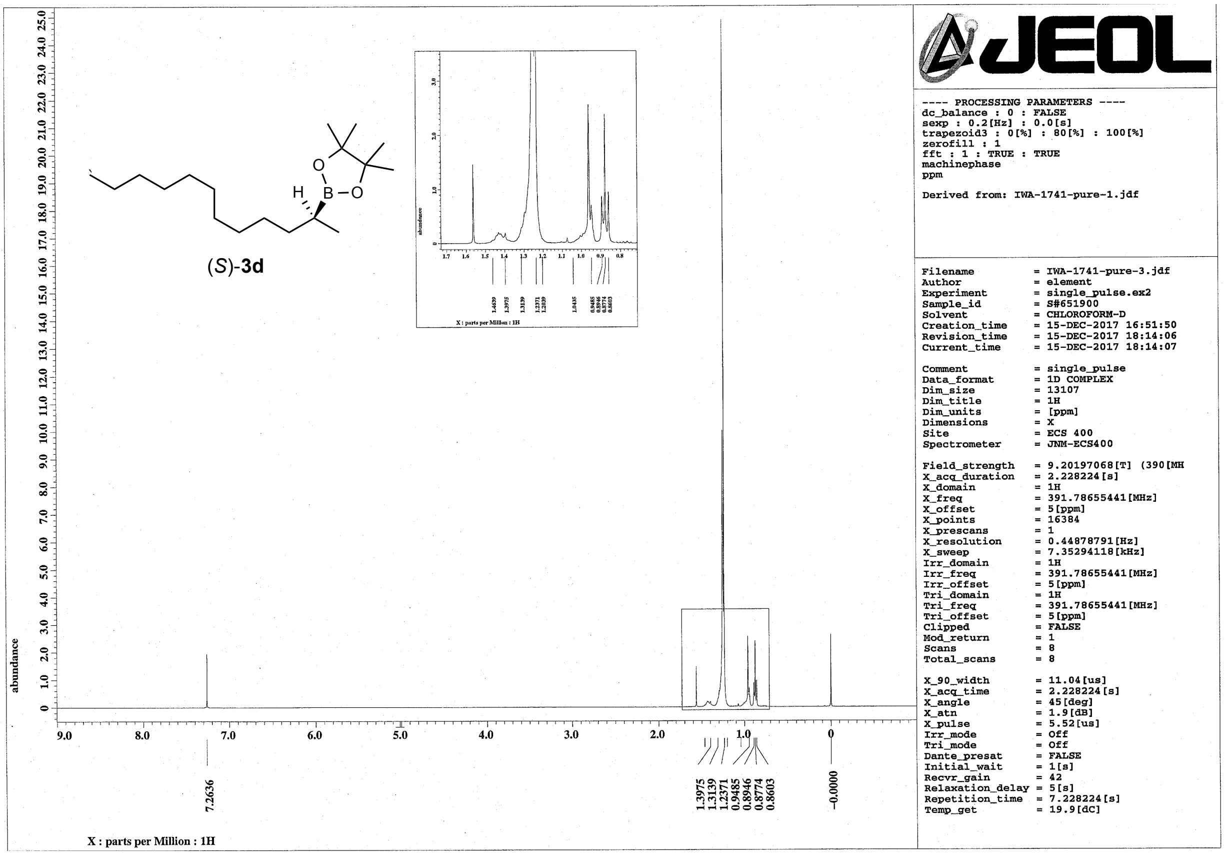

Supplementary Figure 40. ^{1}H NMR spectrum of (*S*)-**3d**.

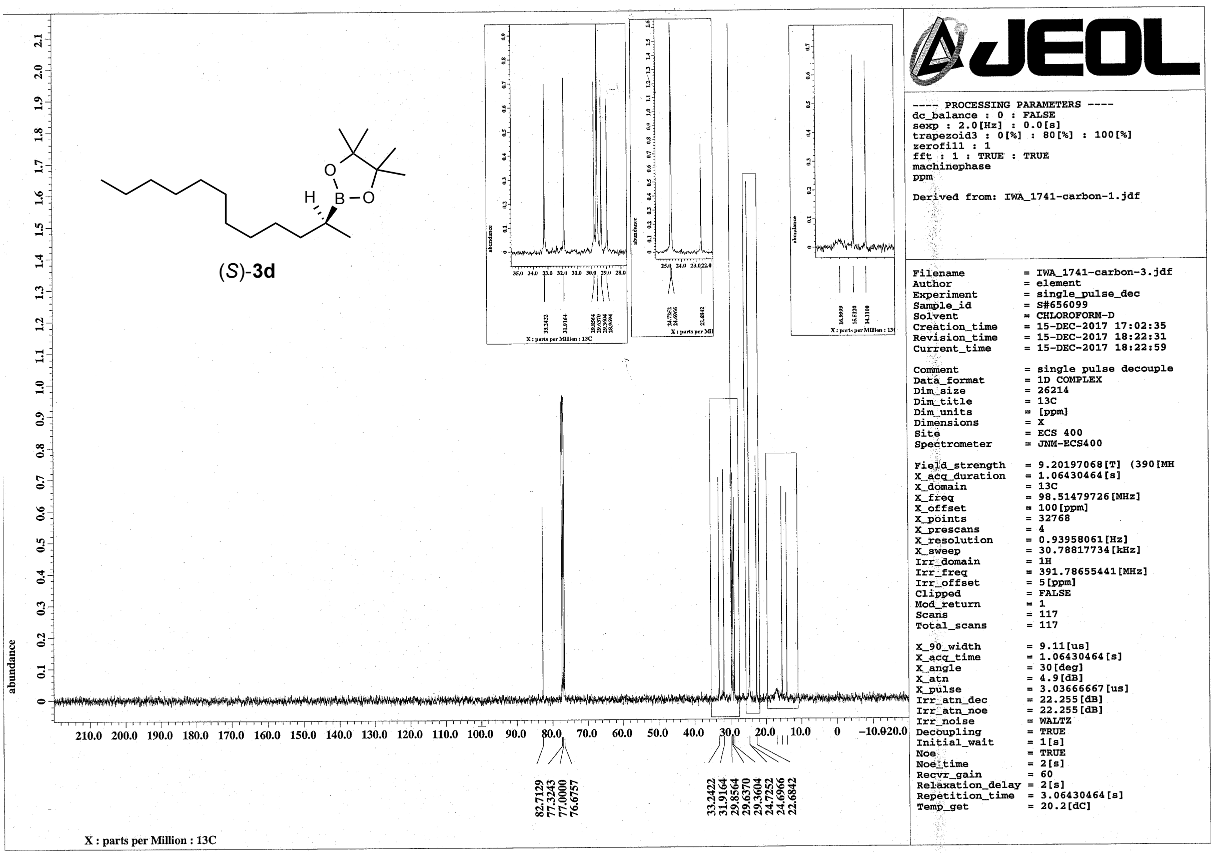

Supplementary Figure 41. ^{13}C NMR spectrum of (*S*)-**3d**.

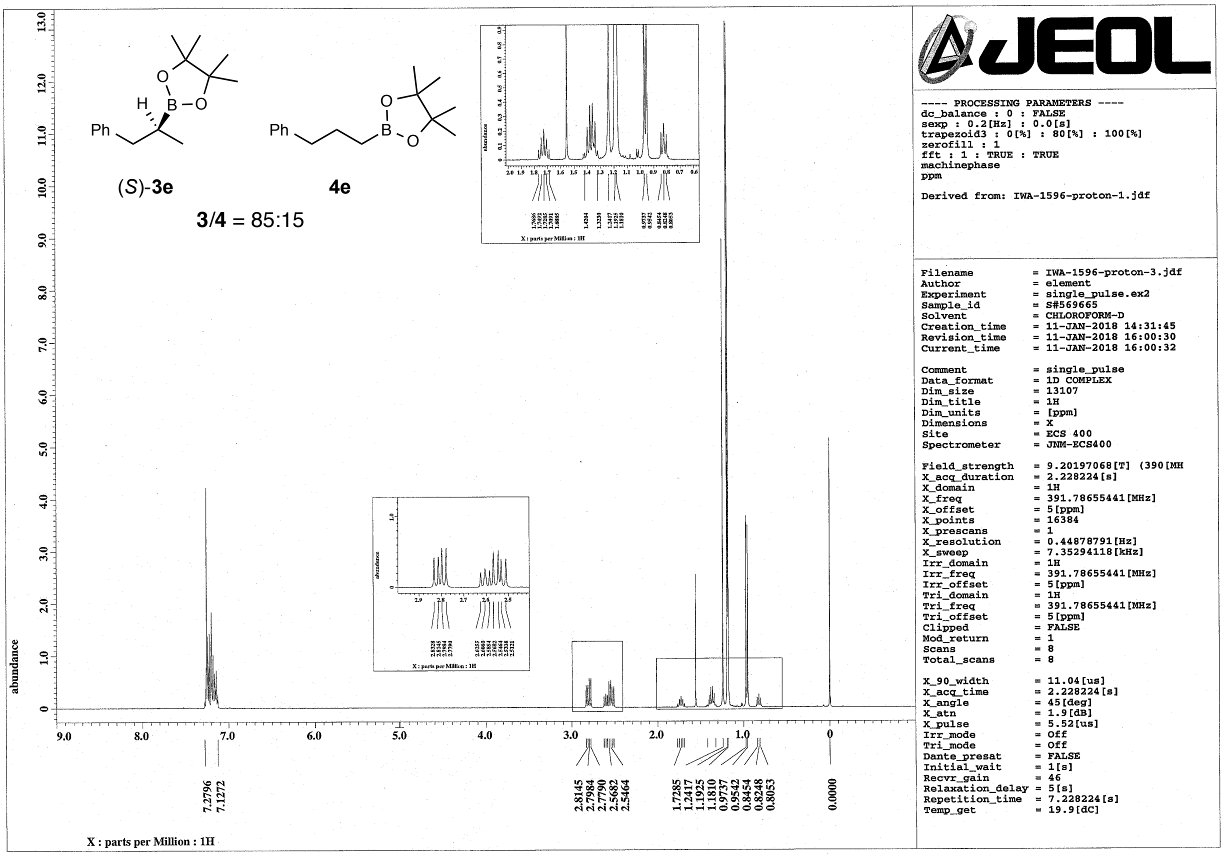

Supplementary Figure 42. ^{1}H NMR spectrum of (*S*)-**3e** and **4e**.

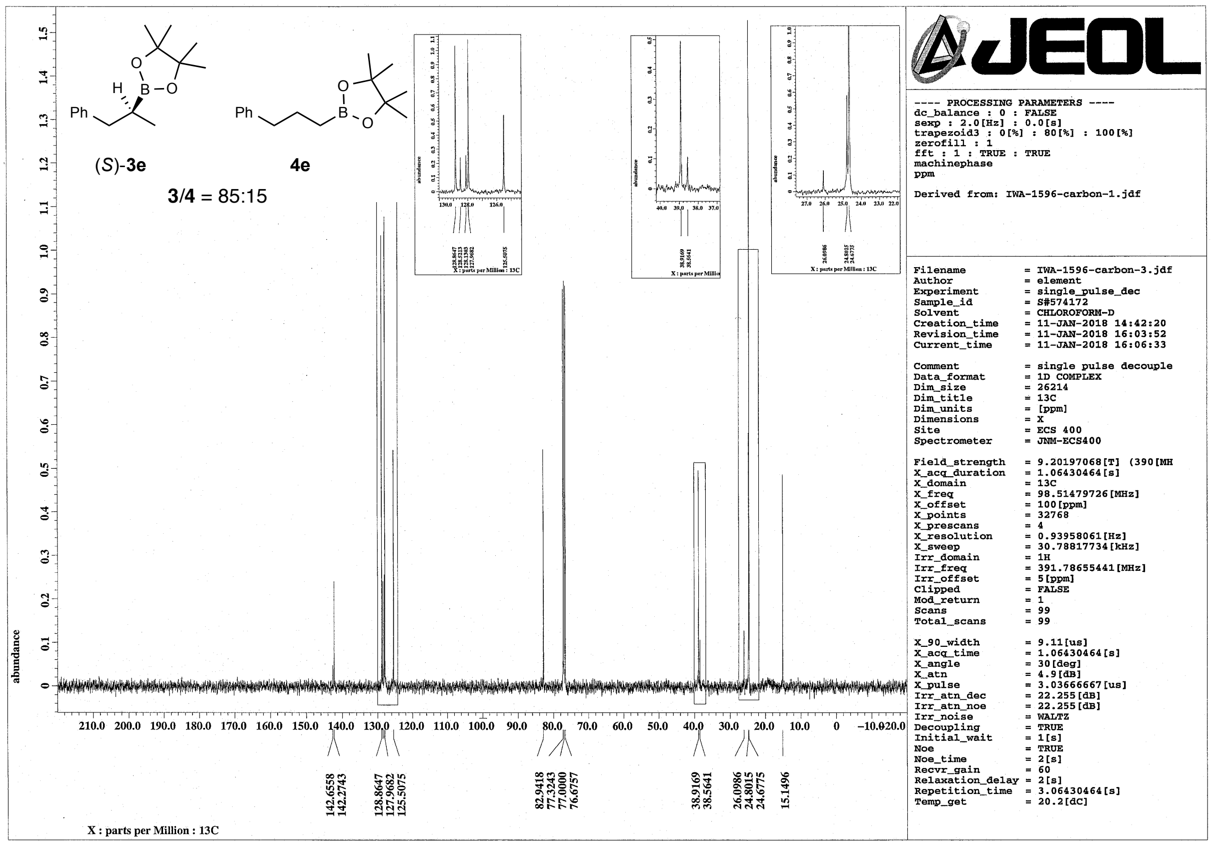

Supplementary Figure 43. ^{13}C NMR spectrum of (*S*)-**3e** and **4e**.

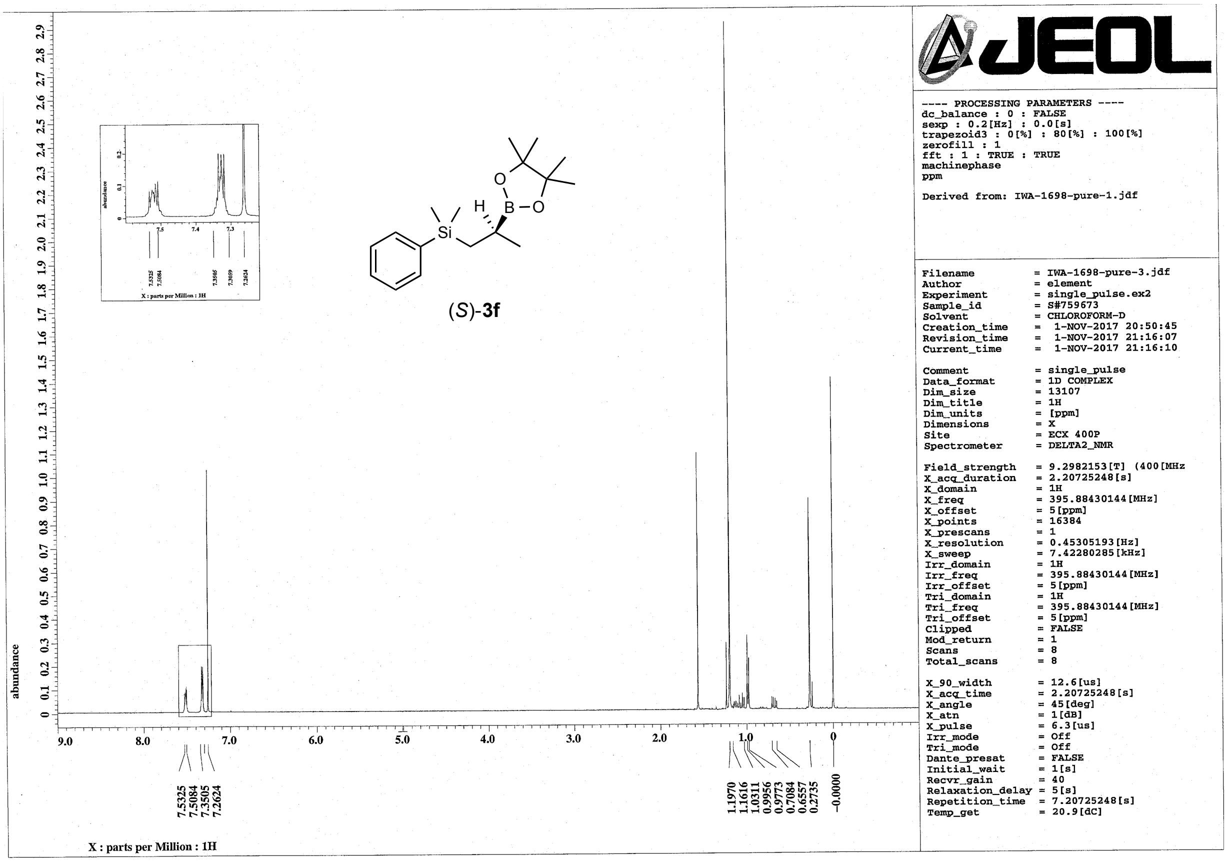

Supplementary Figure 44. ^{1}H NMR spectrum of (*S*)-**3f**.

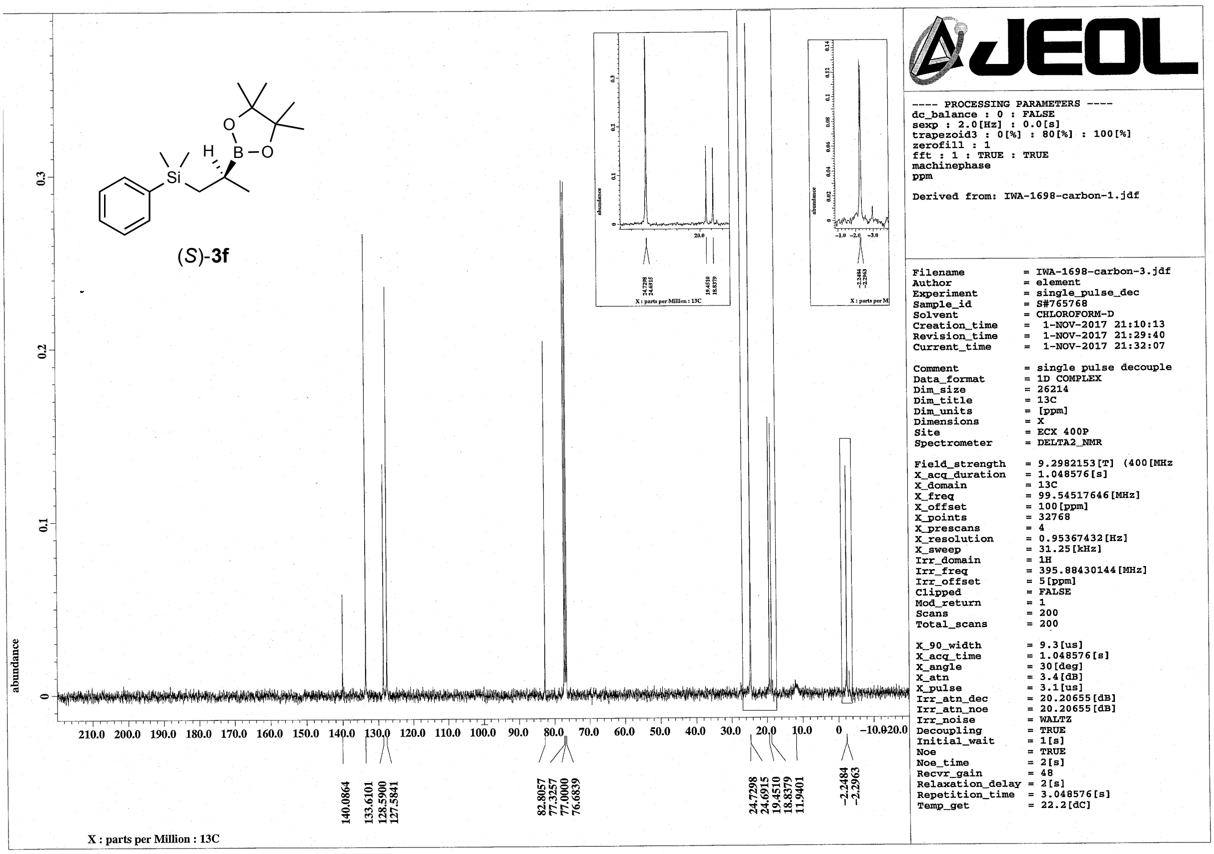

Supplementary Figure 45. ^{13}C NMR spectrum of (*S*)-**3f**.

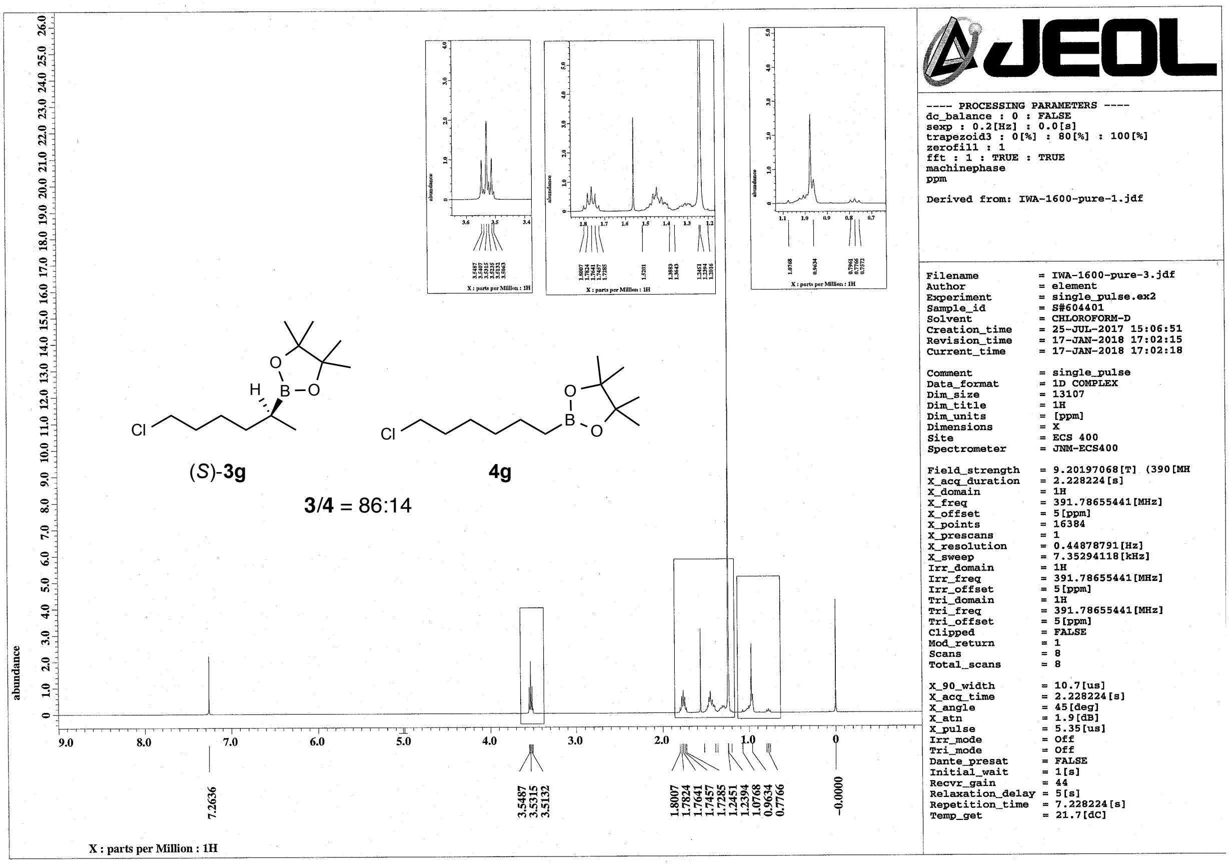

Supplementary Figure 46. ^{1}H NMR spectrum of (*S*)-**3g** and **4g**.

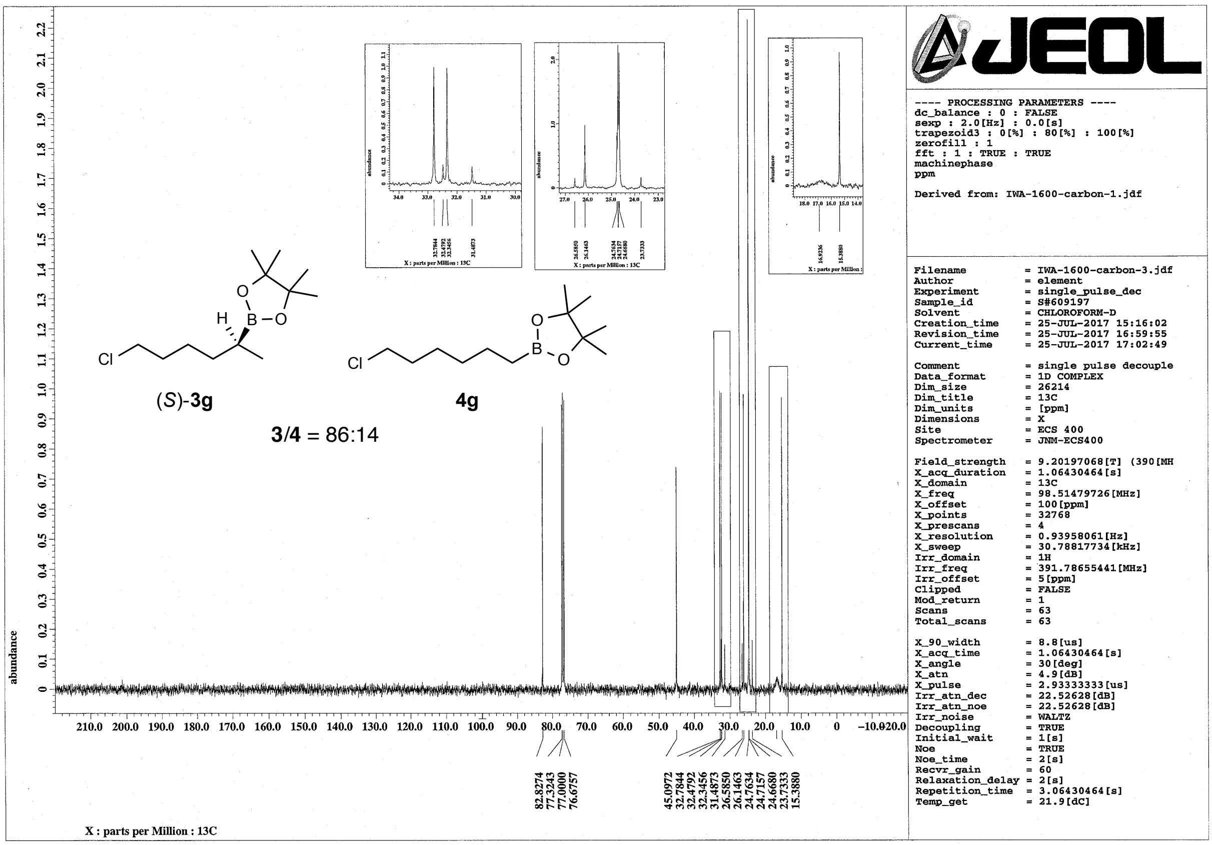

Supplementary Figure 47. ^{13}C NMR spectrum of (*S*)-**3g** and **4g**.

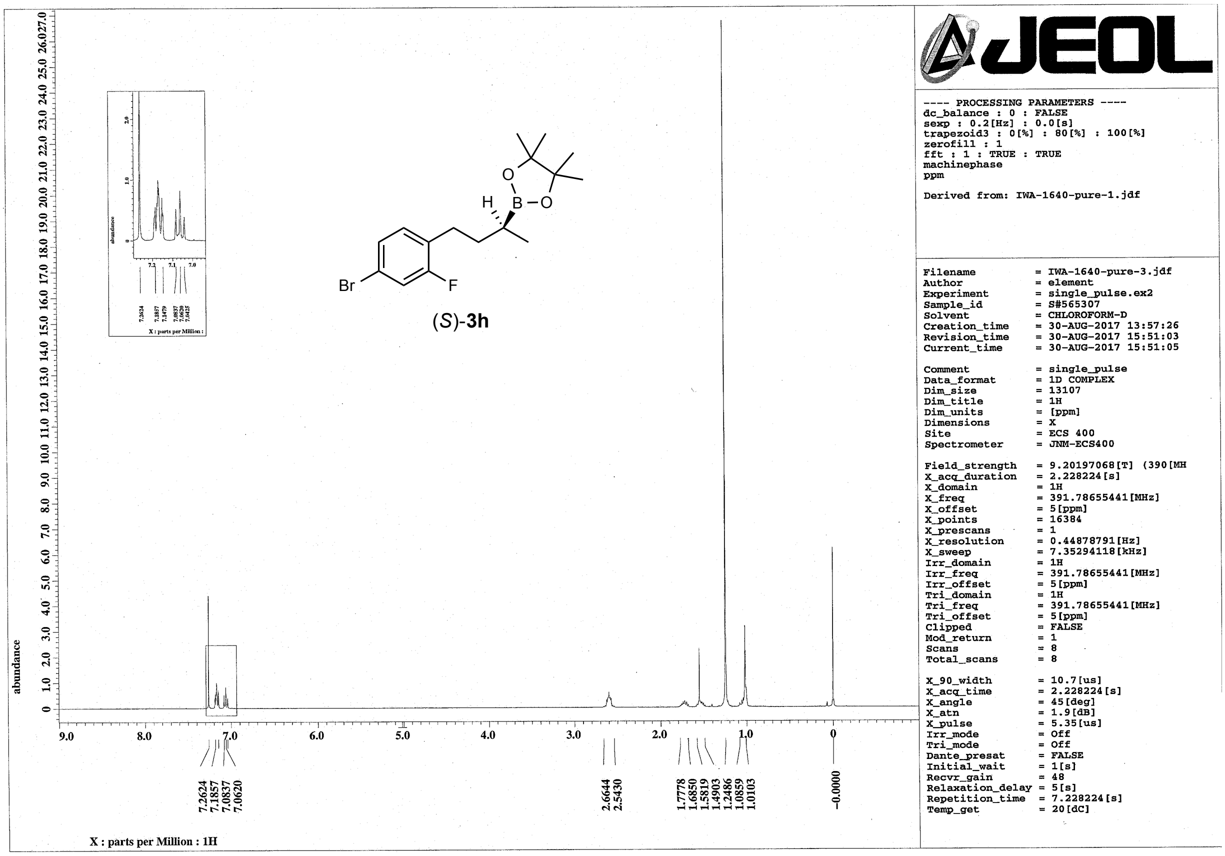

Supplementary Figure 48. ^{1}H NMR spectrum of (*S*)-**3h**.

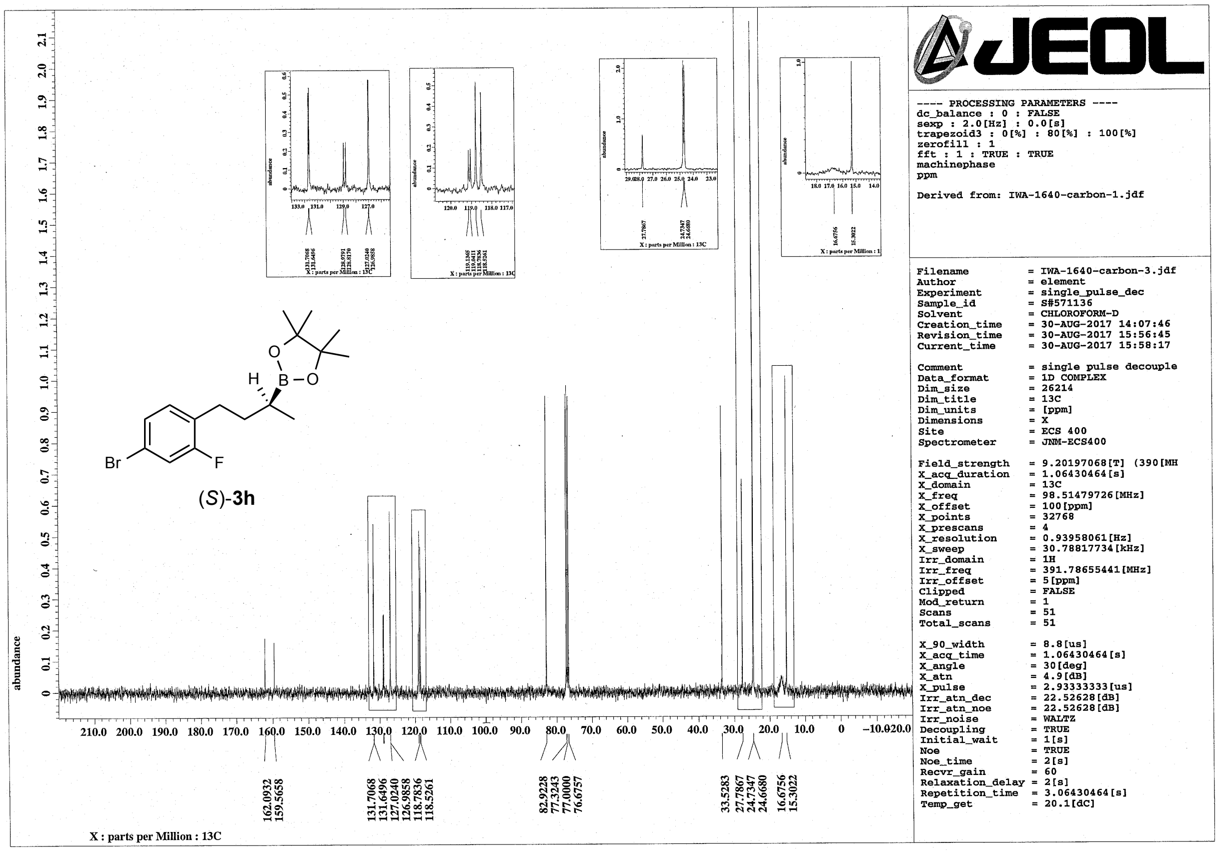

Supplementary Figure 49. ^{13}C NMR spectrum of (*S*)-**3h**.

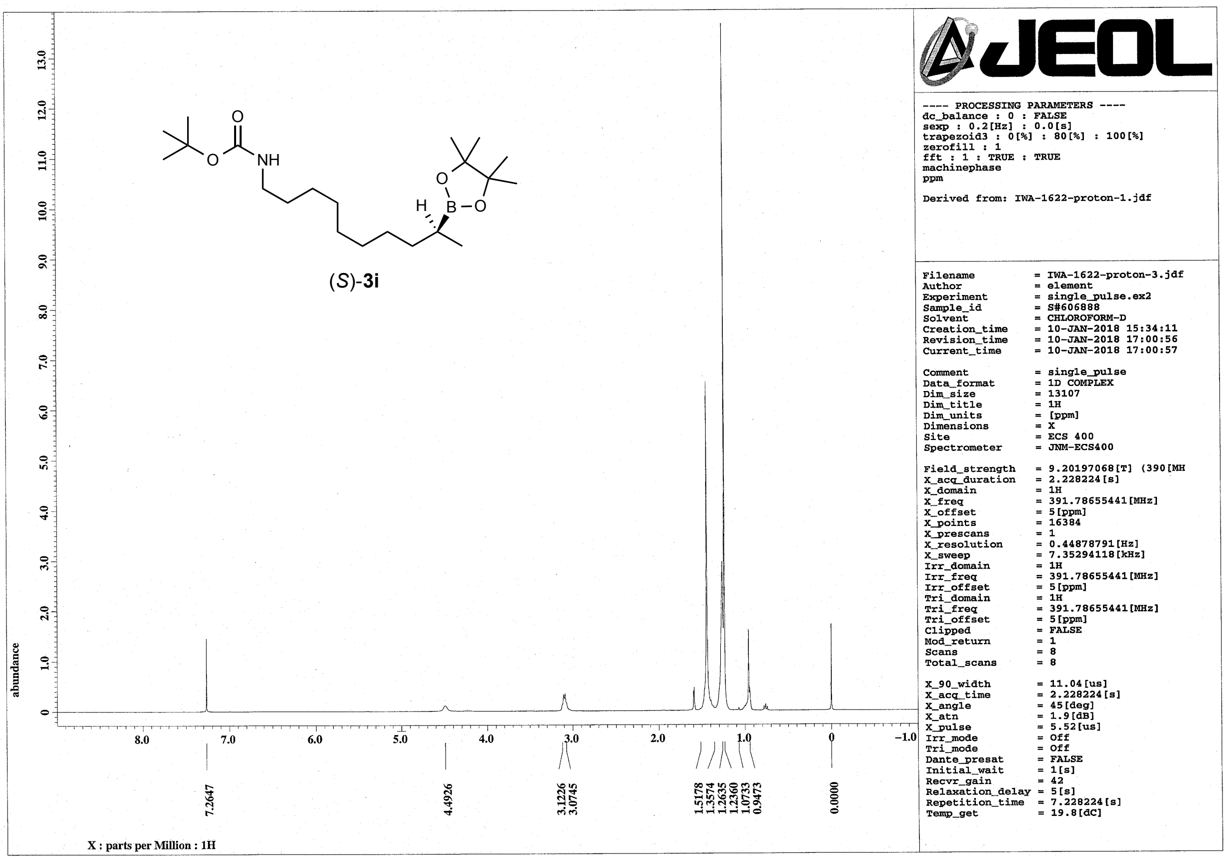

Supplementary Figure 50. ^{1}H NMR spectrum of (*S*)-**3i**.

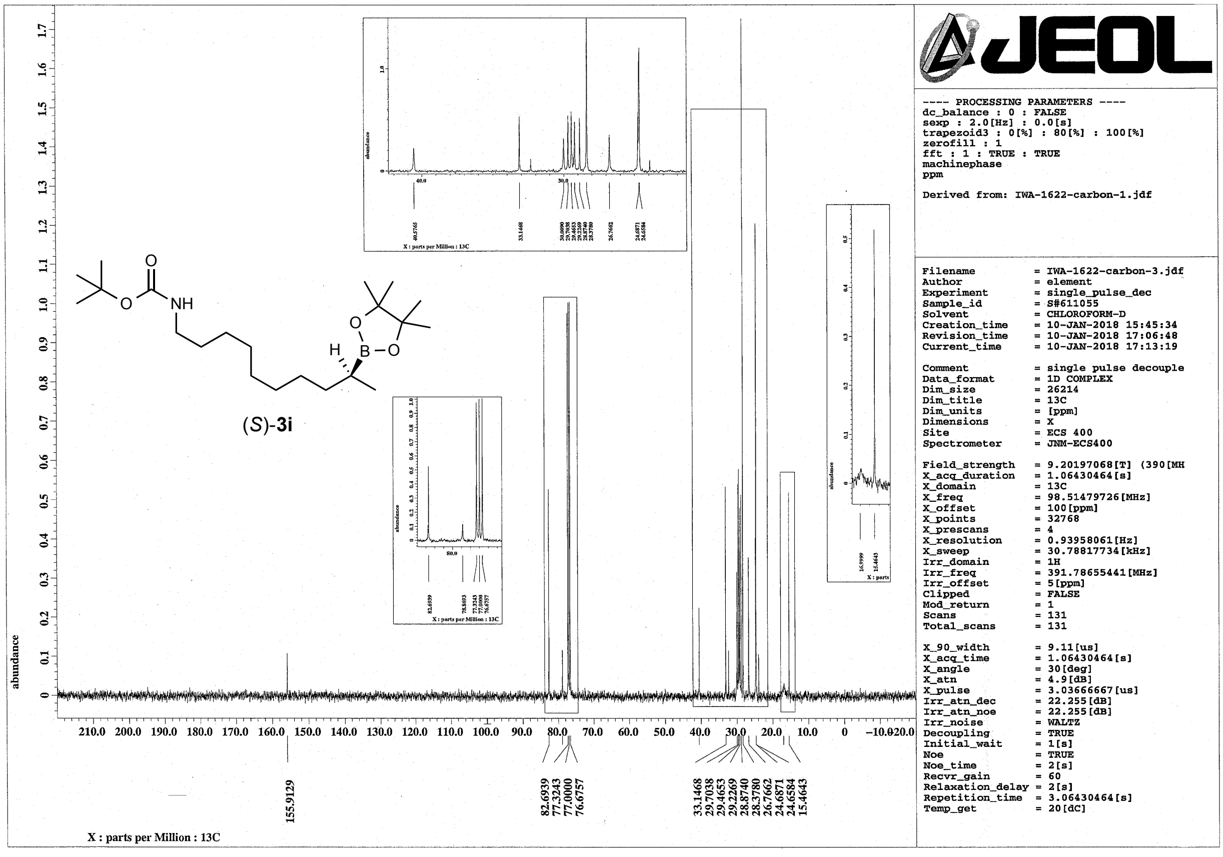

Supplementary Figure 51. ^{13}C NMR spectrum of (*S*)-**3i**.

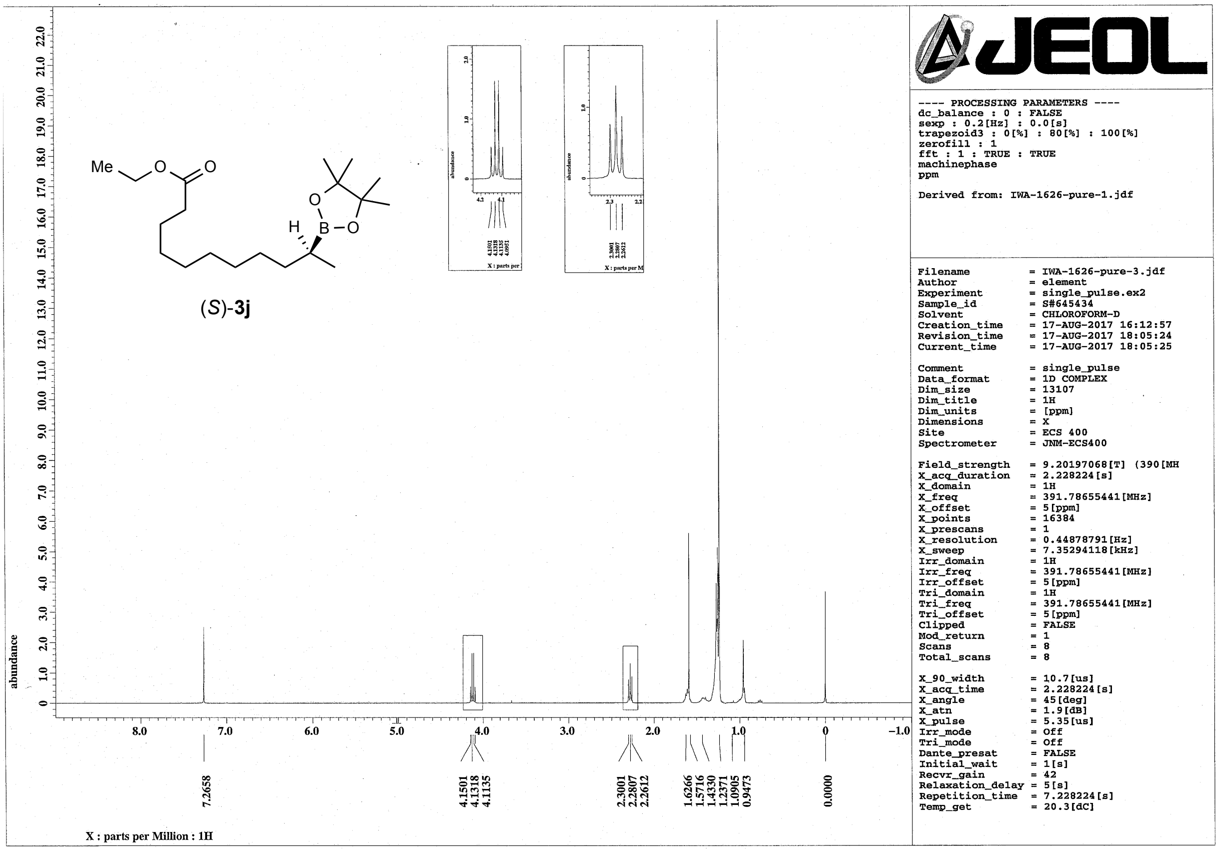

Supplementary Figure 52. ^{1}H NMR spectrum of (*S*)-**3j**.

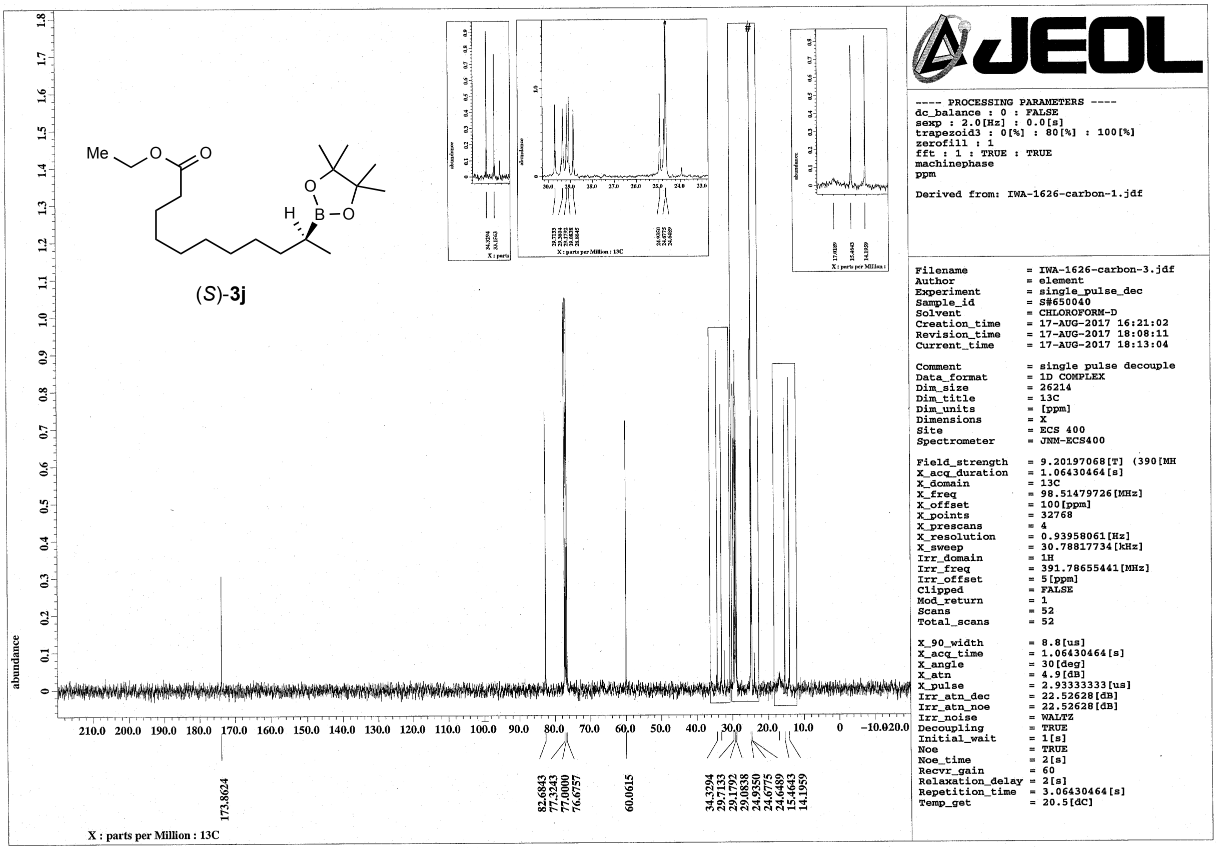

Supplementary Figure 53. ^{13}C NMR spectrum of (*S*)-**3j**.

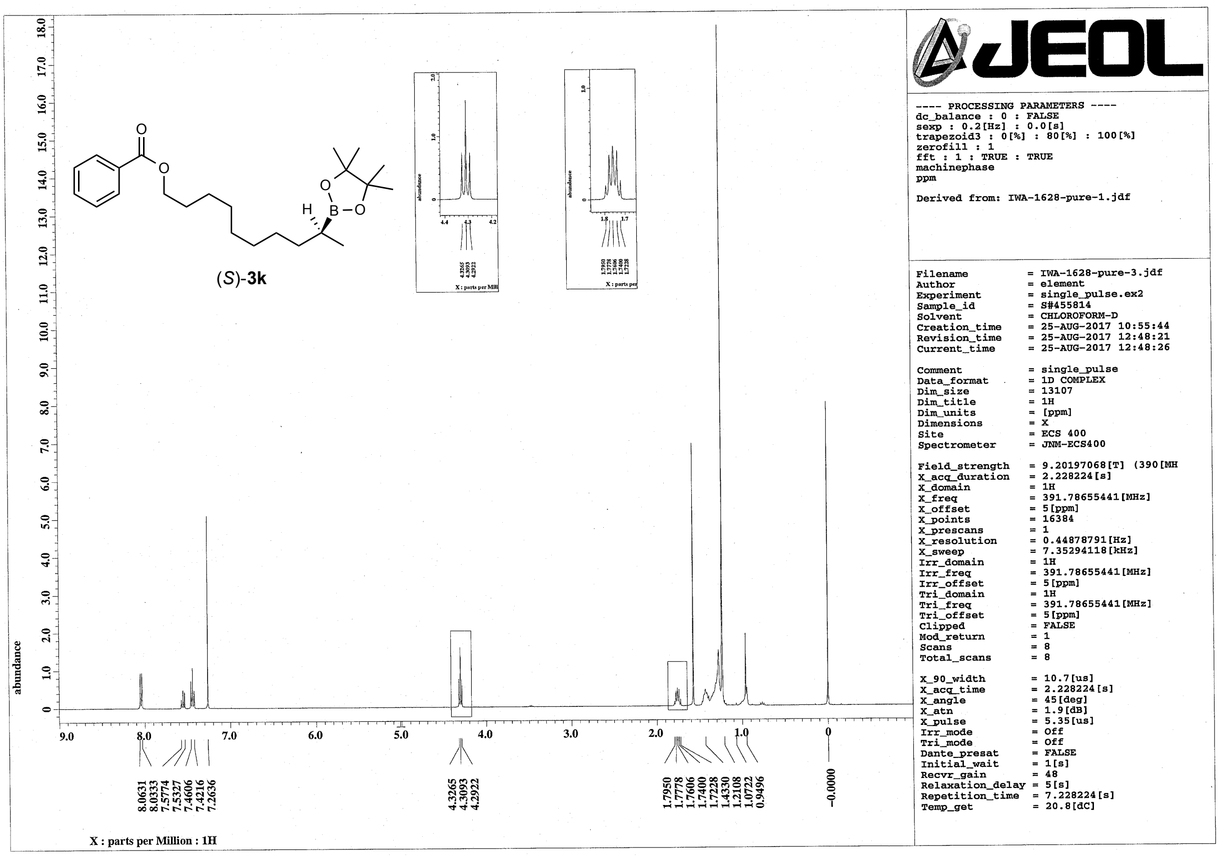

Supplementary Figure 54. ^{1}H NMR spectrum of (*S*)-**3k**.

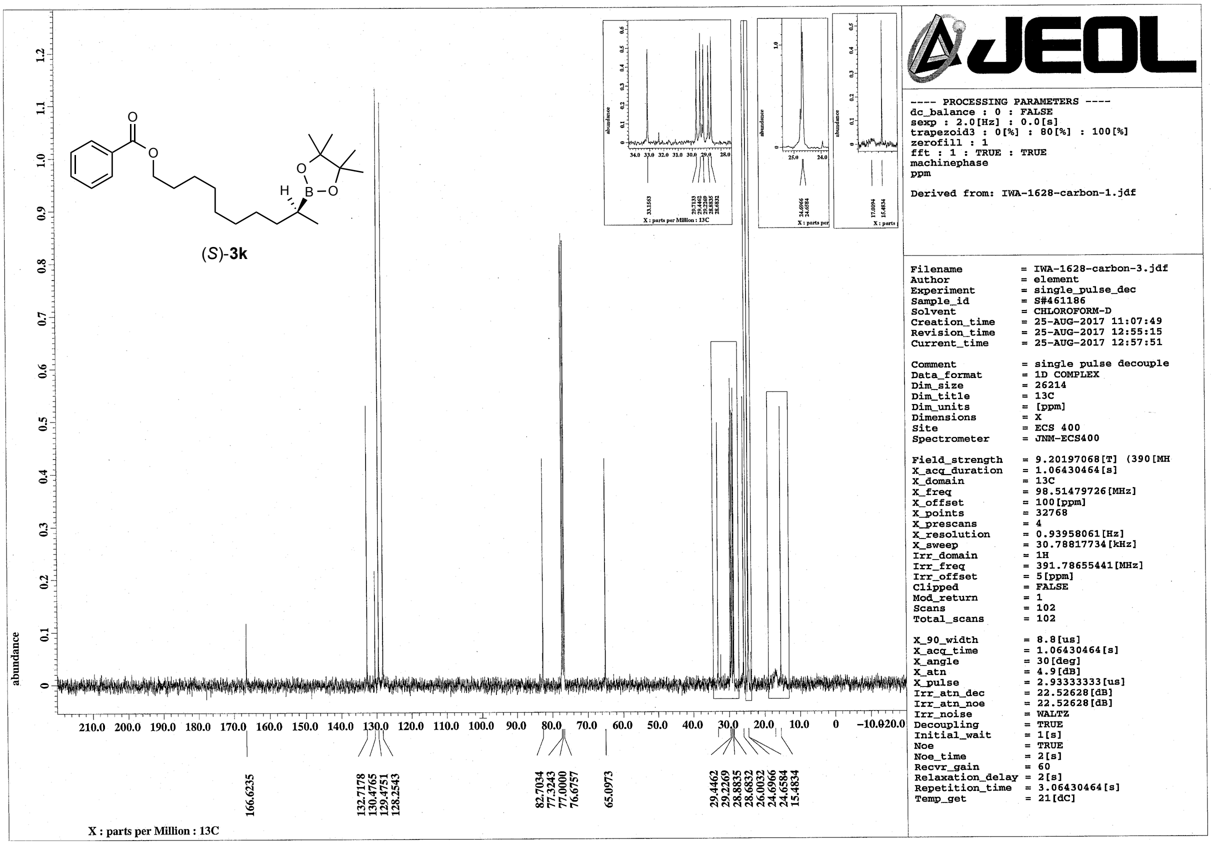

Supplementary Figure 55. ^{13}C NMR spectrum of (*S*)-**3k**.

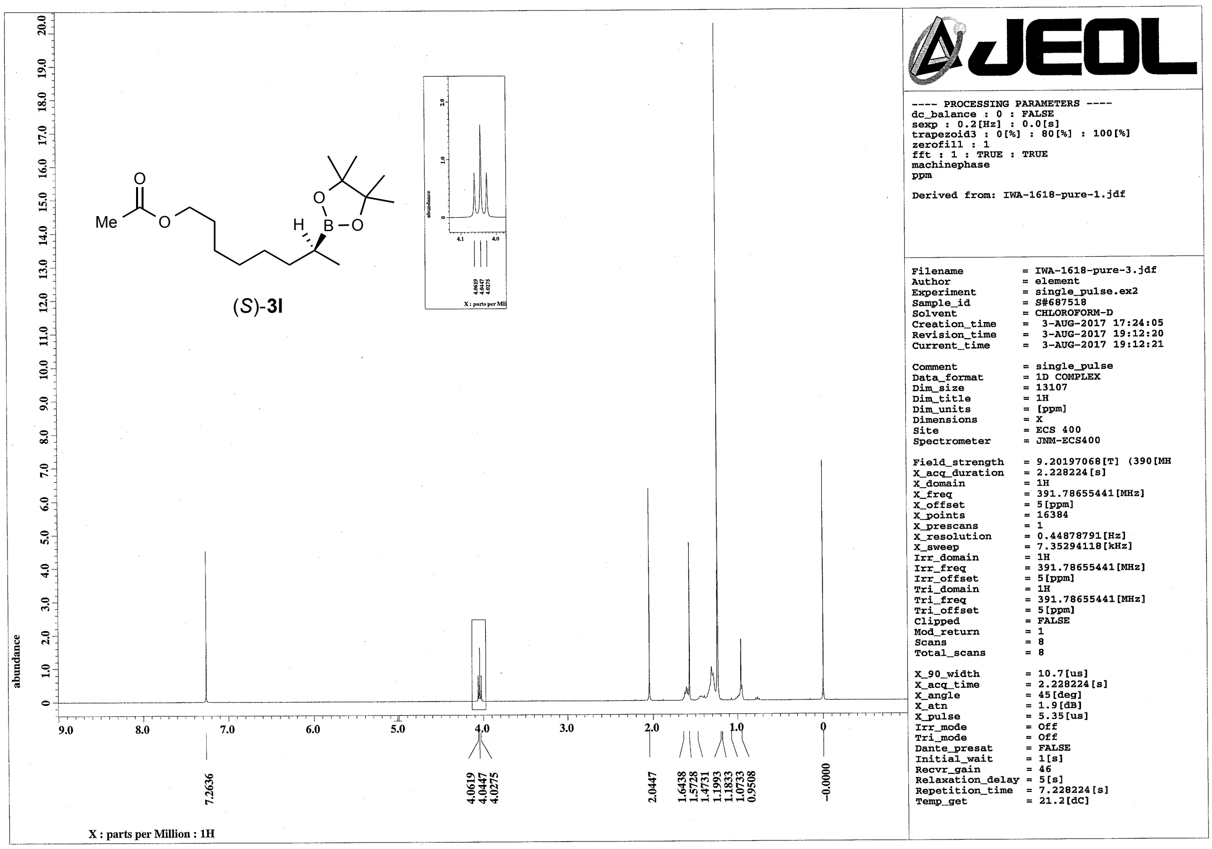

Supplementary Figure 56. ^{1}H NMR spectrum of (*S*)-**3l**.

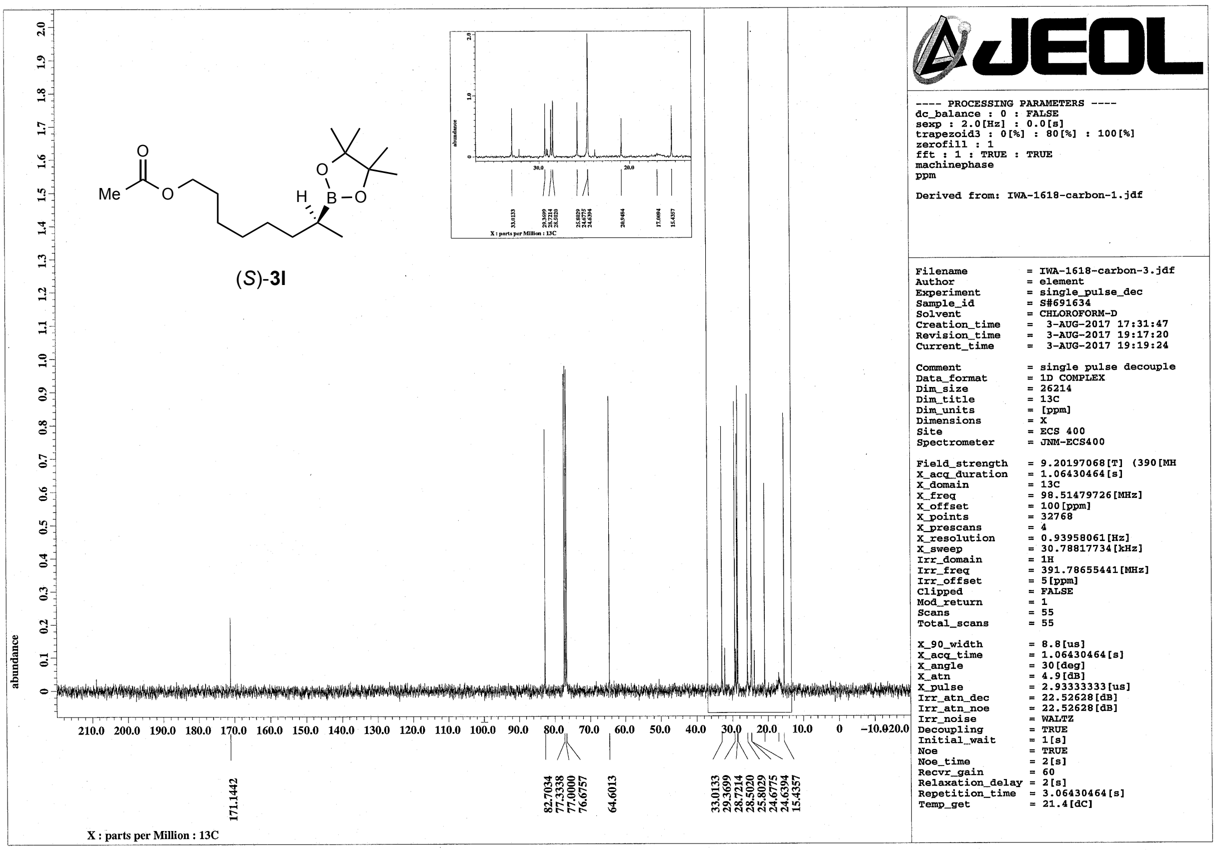

Supplementary Figure 57. ^{13}C NMR spectrum of (*S*)-**3l**.

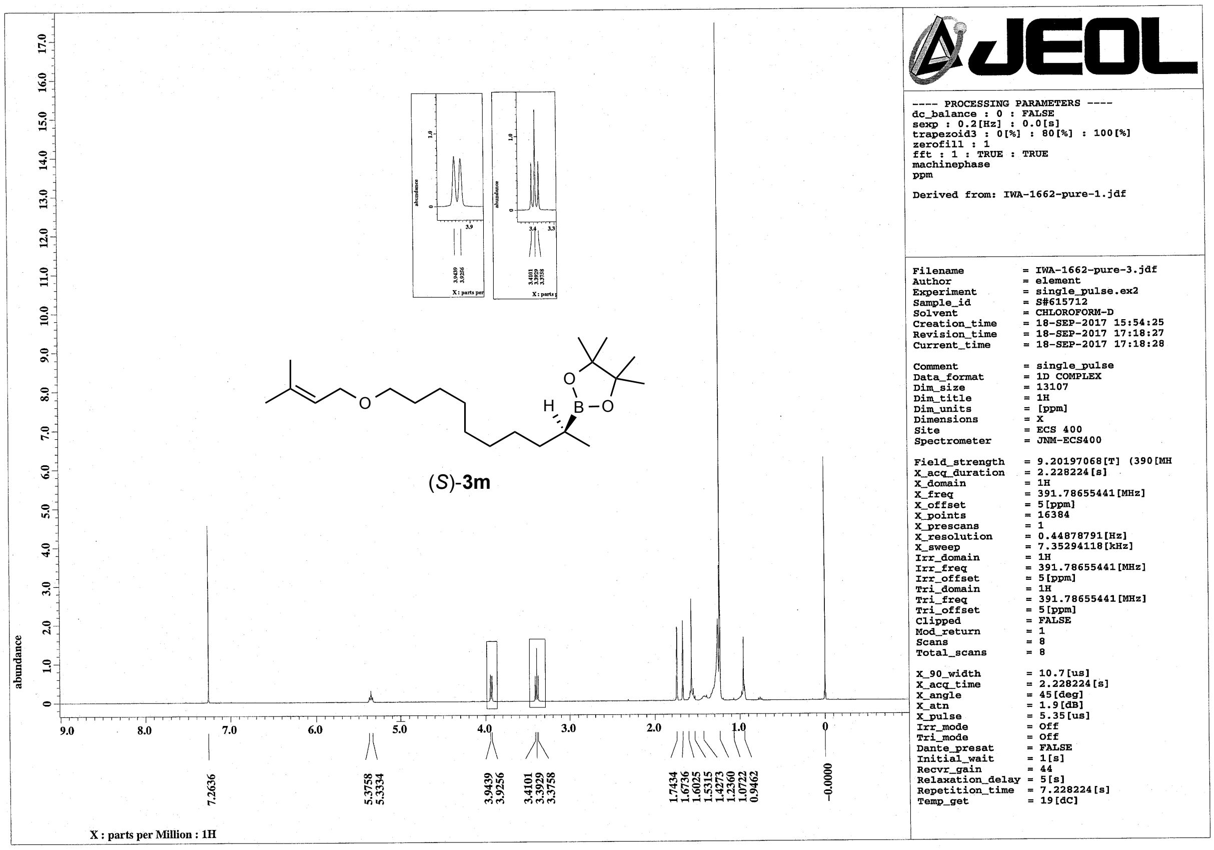

Supplementary Figure 58. ^{1}H NMR spectrum of (*S*)-**3m**.

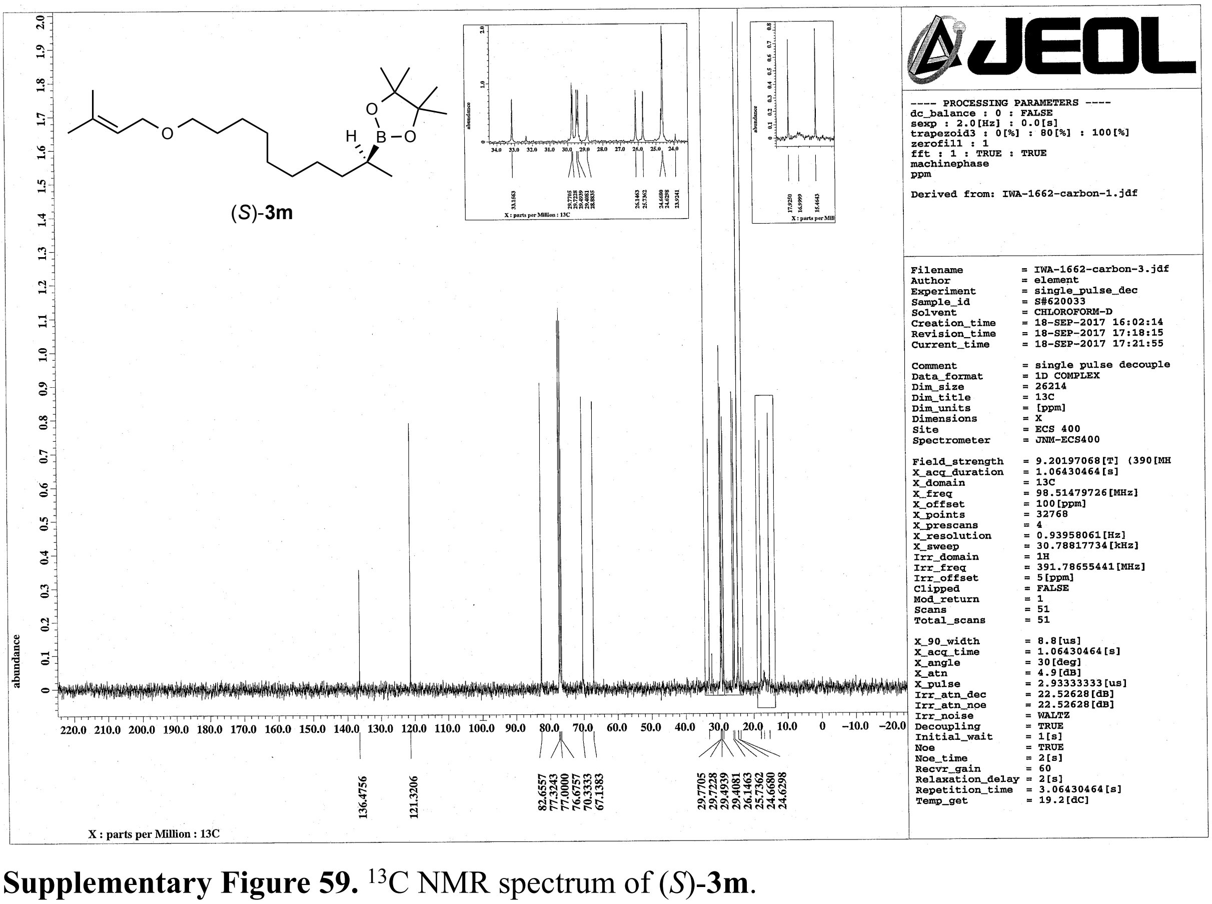

Supplementary Figure 59. ^{13}C NMR spectrum of (*S*)-**3m**.

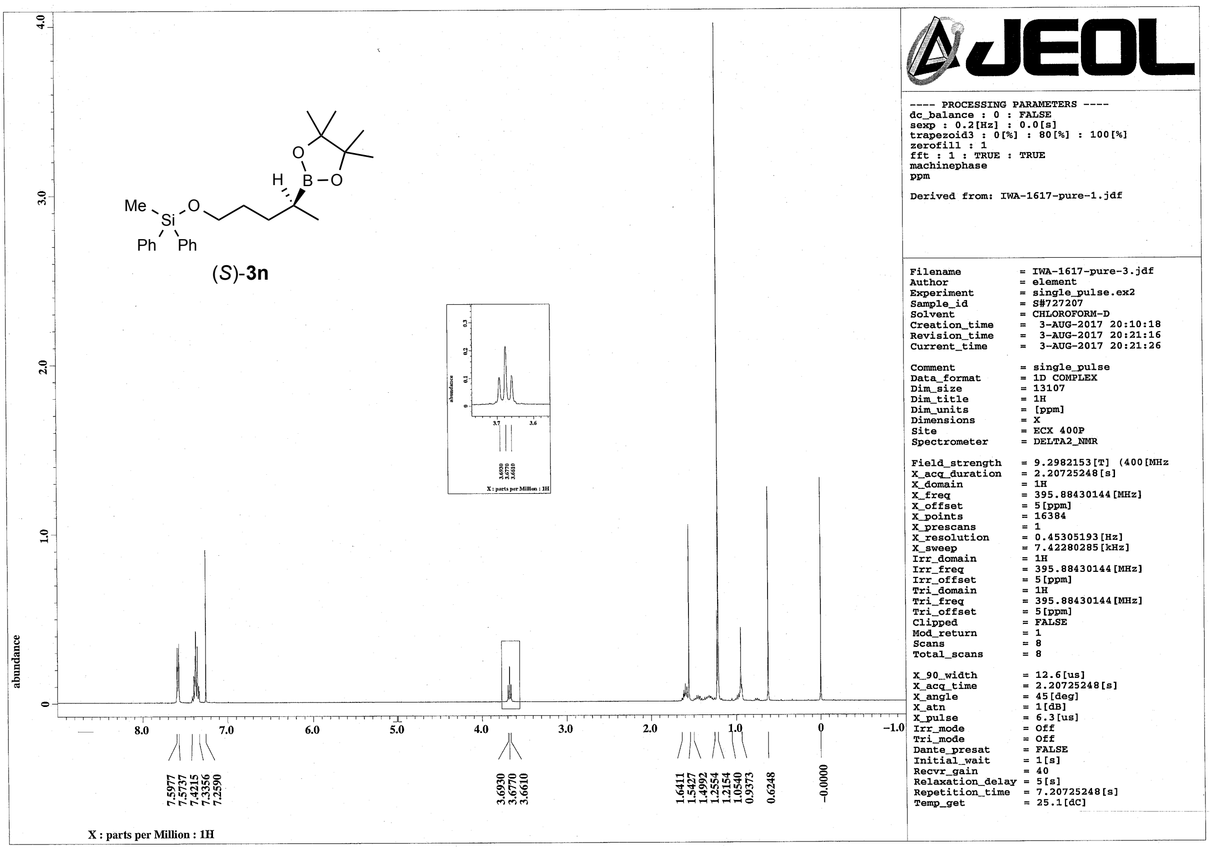

Supplementary Figure 60. ^{1}H NMR spectrum of (*S*)-**3n**.

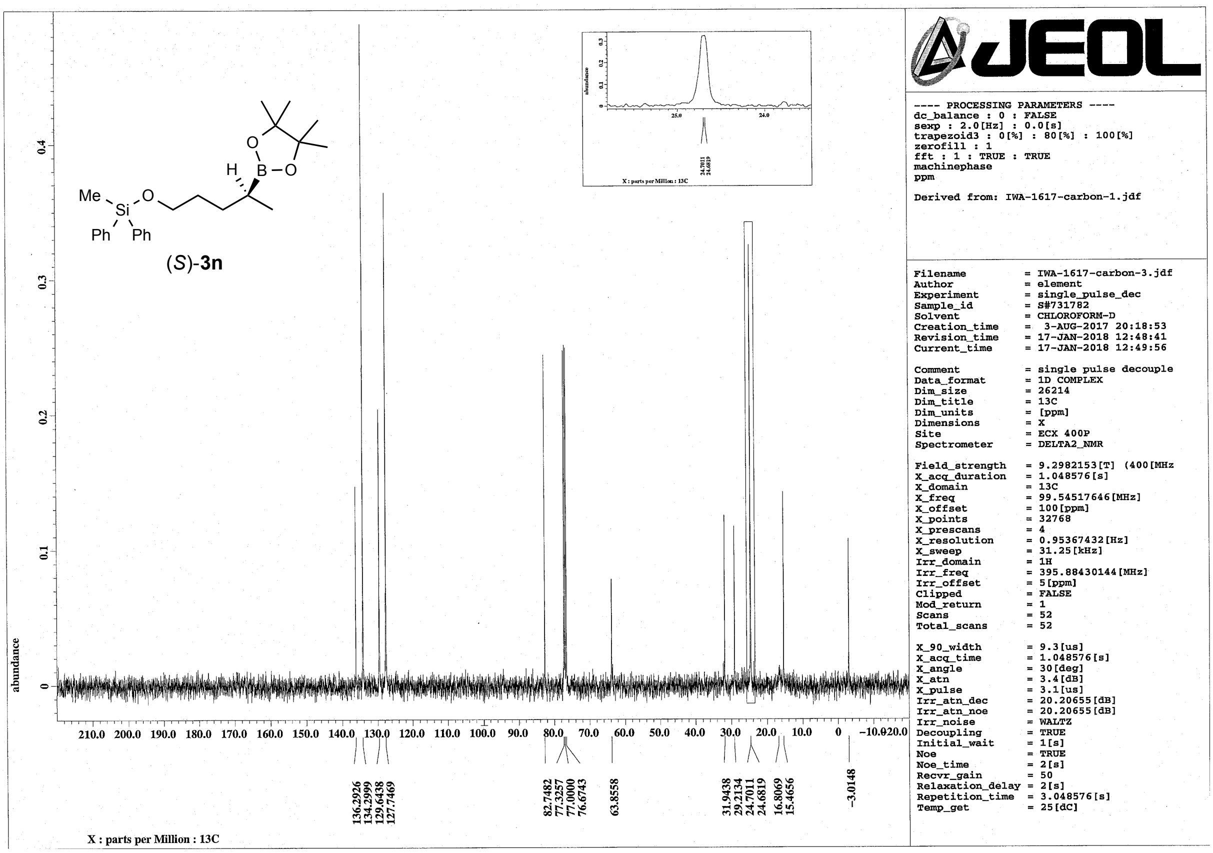

Supplementary Figure 61. ^{13}C NMR spectrum of (*S*)-**3n**.

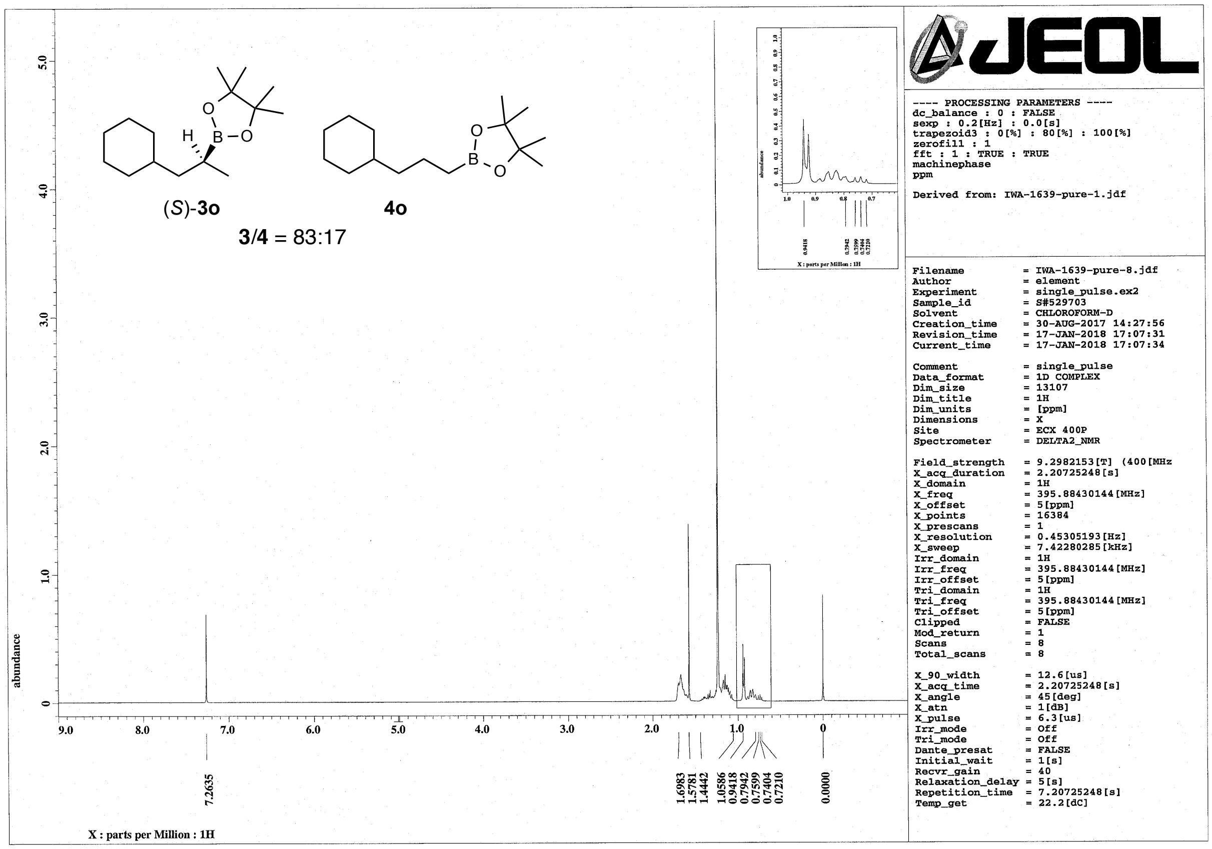

Supplementary Figure 62. ^{1}H NMR spectrum of (*S*)-**3o** and **4o**.

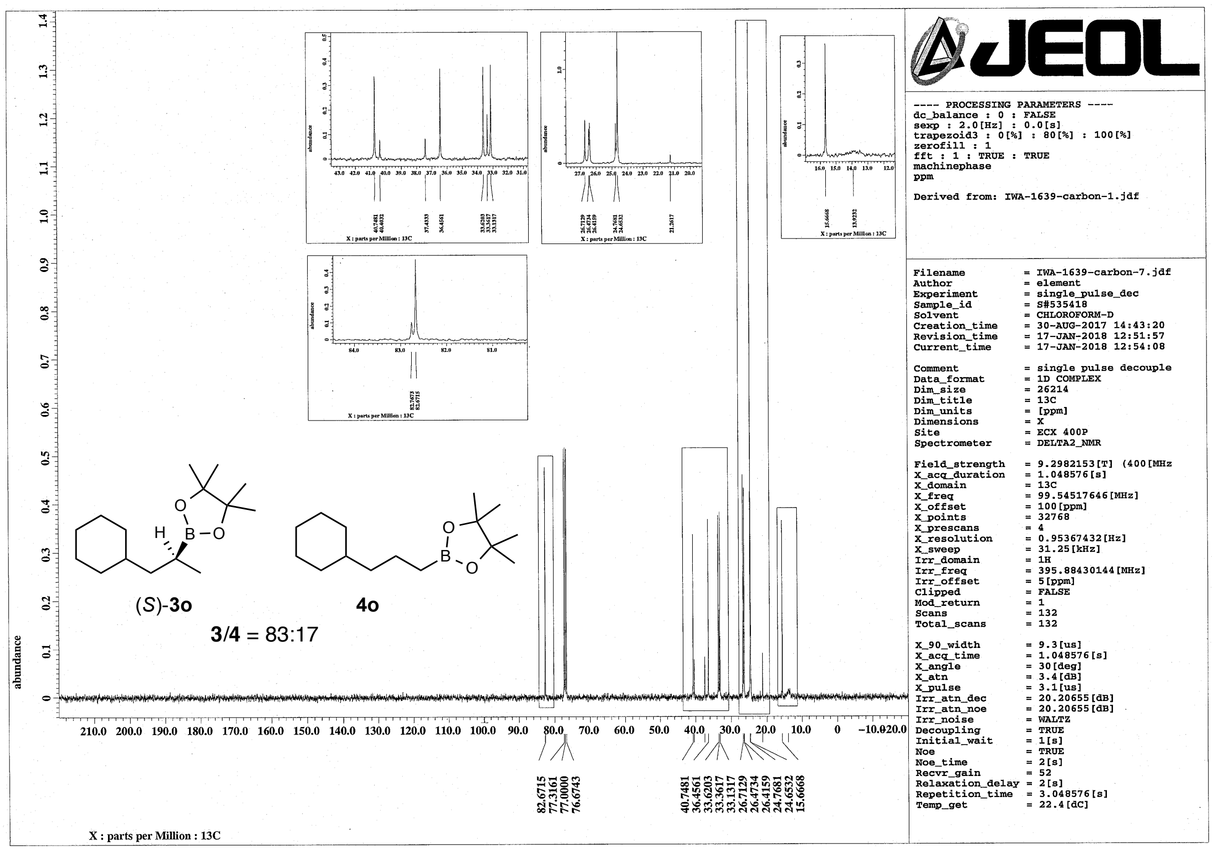

Supplementary Figure 63. ^{13}C NMR spectrum of (*S*)-**3o** and **4o**.

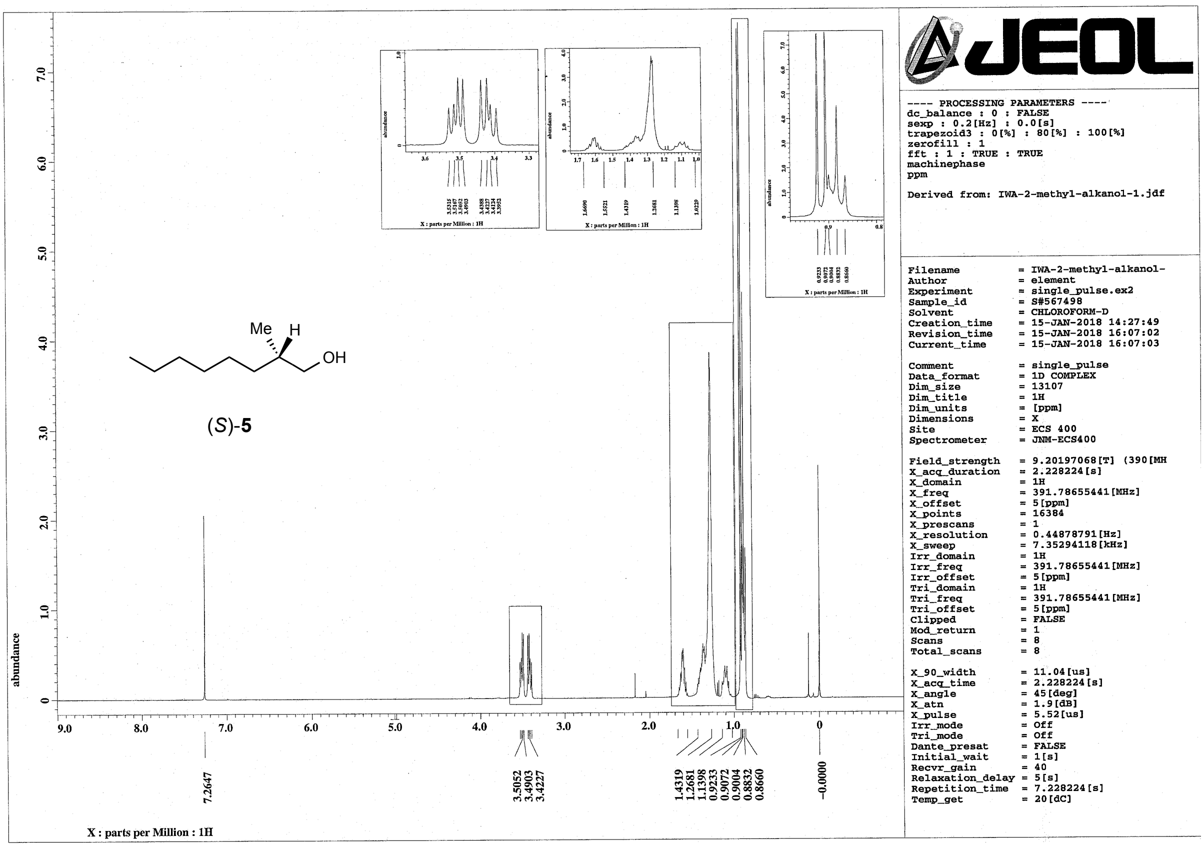

Supplementary Figure 64. ^{1}H NMR spectrum of (*S*)-**5**.

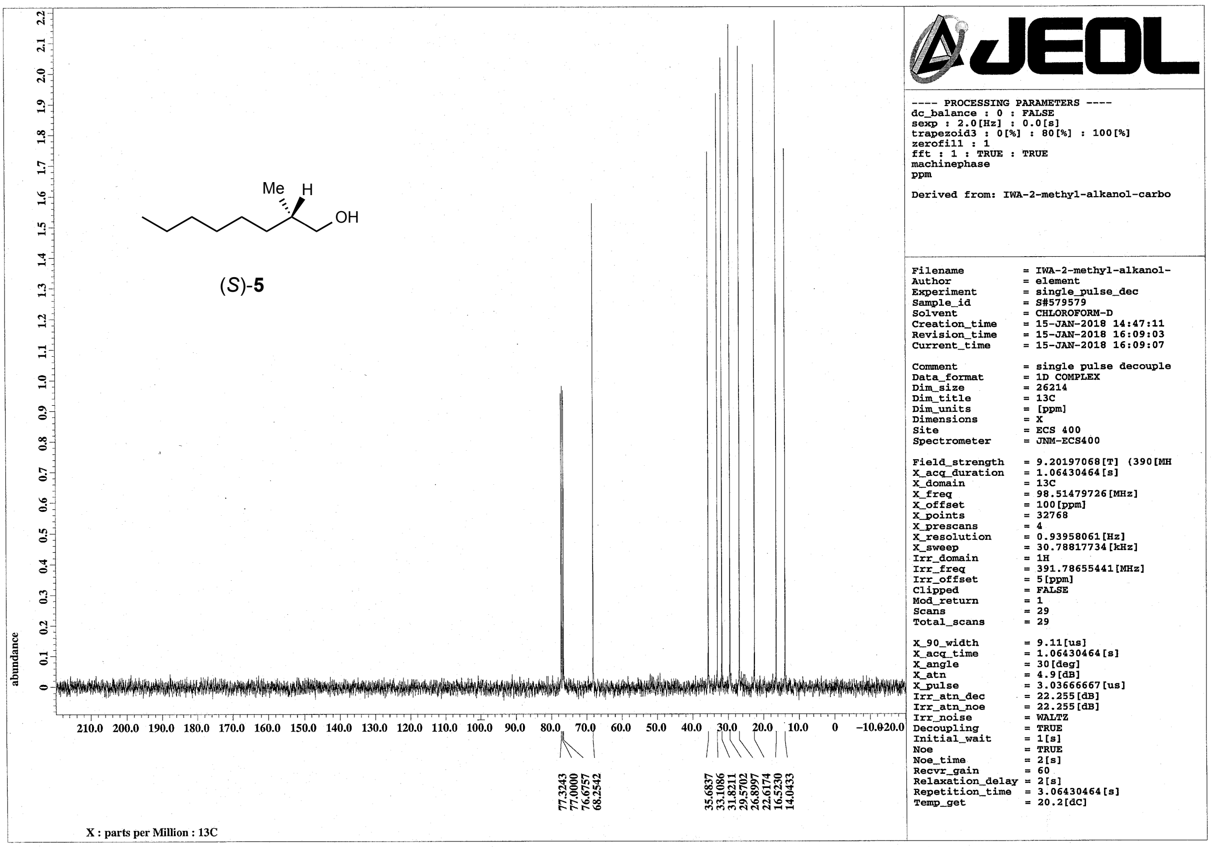

Supplementary Figure 65. ^{13}C NMR spectrum of (*S*)-**5**.

D-2000: Isocratic HPLC Series: 1785 Report Name: modified System: Sys 1

D-2000 Elite HPLC System Manager Report

Analyzed Date and Time: 2017/12/14 12:36
Reported Date and Time: 2018/01/23 16:27
Processed Date and Time: 2018/01/23 16:27
Data Path: C:\WIN32APP\D2000HSM\Isocratic\DATA\1785\
Processing Method: 02/98 iPrOH/Hexane
System (acquisition): Sys 1
Series: 1785
Application(data): Isocratic HPLC
Vial Number: 161
Sample Name: IWA-3a-rac
Vial Type: UNK
Injection from this vial: 1 of 1
Volume: 10.0 ul
Sample Description:

Chrom Type: HPLC Channel : 1

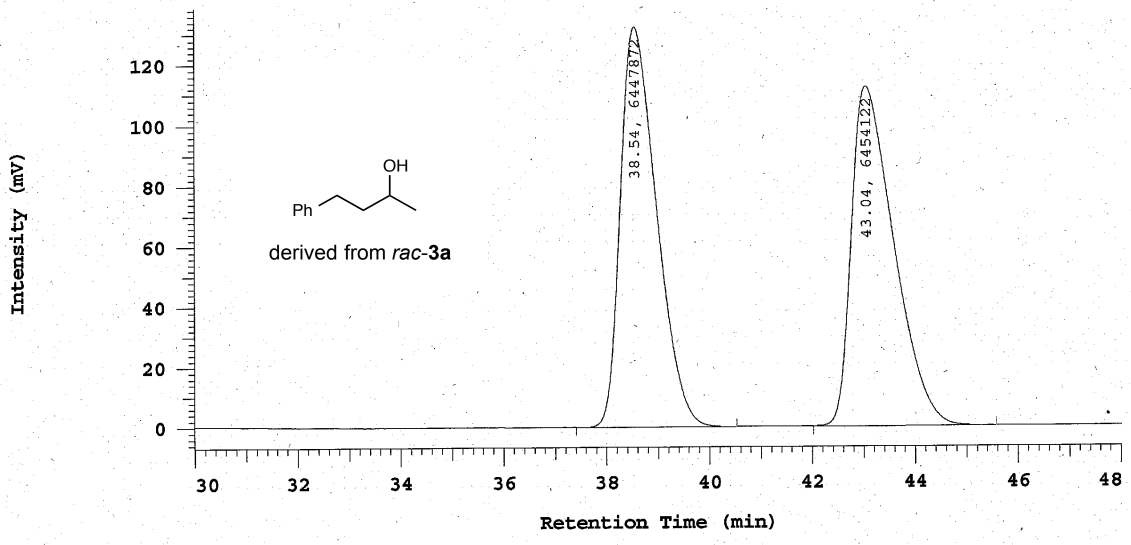

Processing Method: 02/98 iPrOH/Hexane
Column Type: ~~OD-H-2~~ OZ-3
Method Developer: Administrator
Pump A: L-2130
Pump A Solvent A: Hexane
Pump A Solvent B: 10/90 iPrOH/Hexane
Pump A Solvent C: iPrOH
Pump A Solvent D: EtOH
Method Description:

Chrom Type: HPLC Channel : 1
Peak Quantitation: AREA
Calculation Method: AREA%

No.	RT	Area	Area %
1	38.54	6447872	49.976
2	43.04	6454122	50.024
		12901994	100.000

Peak rejection level: 0

Supplementary Figure 66. HPLC trace of the alcohol derived from *rac*-**3a**.

D-2000: Isocratic HPLC Series: 1786 Report Name: modified System: Sys 1

D-2000 Elite HPLC System Manager Report

Analyzed Date and Time: 2017/12/14 13:48
Reported Date and Time: 2018/01/23 16:09
Processed Date and Time: 2018/01/23 16:06
Data Path: C:\WIN32APP\D2000HSM\Isocratic\DATA\1786\
Processing Method: 02/98 iPrOH/Hexane
System (acquisition): Sys 1
Application(data): Isocratic HPLC
Sample Name: IWA-1564-s-Quinox-tODAd-40-3
Injection from this vial: 1 of 1
Sample Description:
Series: 1786
Vial Number: 162
Vial Type: UNK
Volume: 10.0 ul

Chrom Type: HPLC Channel : 1

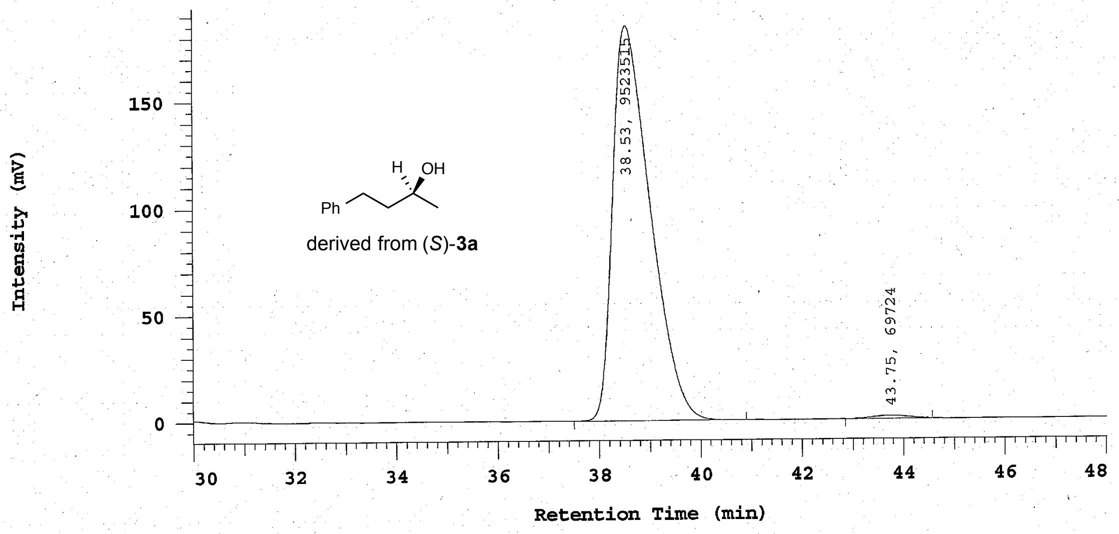

Processing Method: 02/98 iPrOH/Hexane
Column Type: ~~OD-H-2~~ OZ-3
Method Developer: Administrator
Pump A: L-2130
Pump A Solvent A: Hexane
Pump A Solvent B: 10/90 iPrOH/Hexane
Pump A Solvent C: iPrOH
Pump A Solvent D: EtOH
Method Description:

Chrom Type: HPLC Channel : 1
Peak Quantitation: AREA
Calculation Method: AREA%

No.	RT	Area	Area %
1	38.53	9523515	99.273
2	43.75	69724	0.727
		9593239	100.000

Peak rejection level: 0

Supplementary Figure 67. HPLC trace of the alcohol derived from (*S*)-**3a**.

D-2000: Isocratic HPLC Series: 1781 Report Name: modified System: Sys 1

D-2000 Elite HPLC System Manager Report

Analyzed Date and Time: 2017/12/11 13:10   Reported Date and Time: 2018/01/23 16:16

Processed Date and Time: 2018/01/23 16:16

Data Path: C:\WIN32APP\D2000HSM\Isocratic\DATA\1781\

Processing Method: 0.1/99.9 iPrOH/Hexane

System (acquisition): Sys 1   Series: 1781

Application(data): Isocratic HPLC   Vial Number: 182

Sample Name: IWA-1731-rac   Vial Type: UNK

Injection from this vial: 1 of 1   Volume: 10.0 ul

Sample Description:

Chrom Type: HPLC Channel : 1

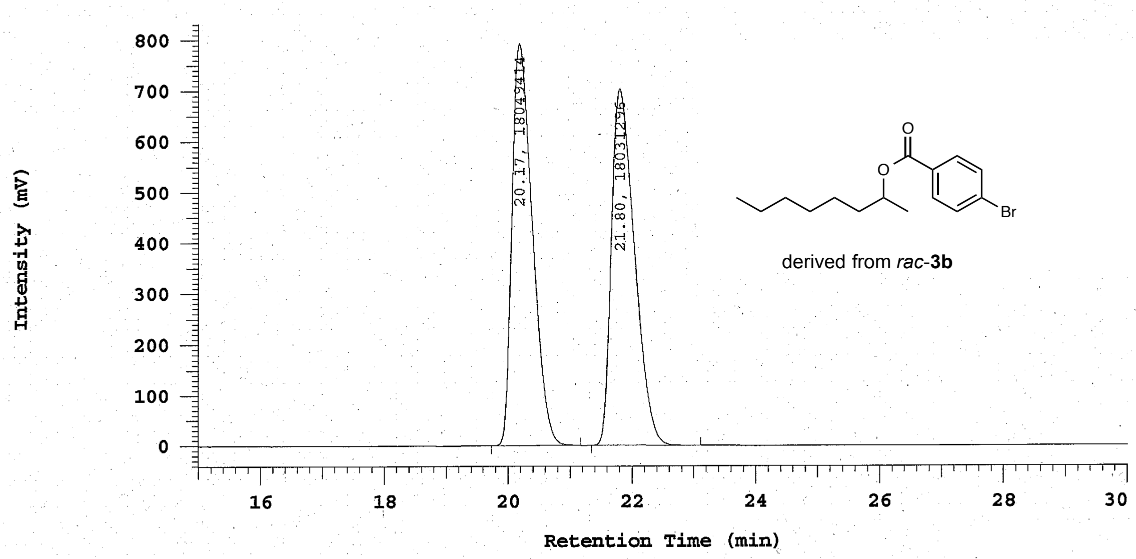

Processing Method: 0.1/99.9 iPrOH/Hexane

Column Type: ~~OD-H-2~~ OZ-3   Method Developer: Administrator

Pump A: L-2130

Pump A Solvent A: Hexane   Pump A Solvent B: 10/90 iPrOH/Hexane

Pump A Solvent C: iPrOH   Pump A Solvent D: EtOH

Method Description:

Chrom Type: HPLC Channel : 1

Peak Quantitation: AREA

Calculation Method: AREA%

No.	RT	Area	Area %
1	20.17	18049414	50.025
2	21.80	18031296	49.975
		36080710	100.000

Peak rejection level: 0

Supplementary Figure 68. HPLC trace of the ester derived from *rac*-**3b**.

D-2000 Elite HPLC System Manager Report

Analyzed Date and Time: 2017/12/11 12:09
Reported Date and Time: 2018/01/23 16:17
Processed Date and Time: 2018/01/23 16:17
Data Path: C:\WIN32APP\D2000HSM\Isocratic\DATA\1780\
Processing Method: 0.1/99.9 iPrOH/Hexane
System (acquisition): Sys 1
Series: 1780
Application(data): Isocratic HPLC
Vial Number: 181
Sample Name: IWA-1739-s-QuinoxtODAd-40-3
Vial Type: UNK
Volume: 10.0 ul
Injection from this vial: 1 of 1
Sample Description:

Chrom Type: HPLC Channel : 1

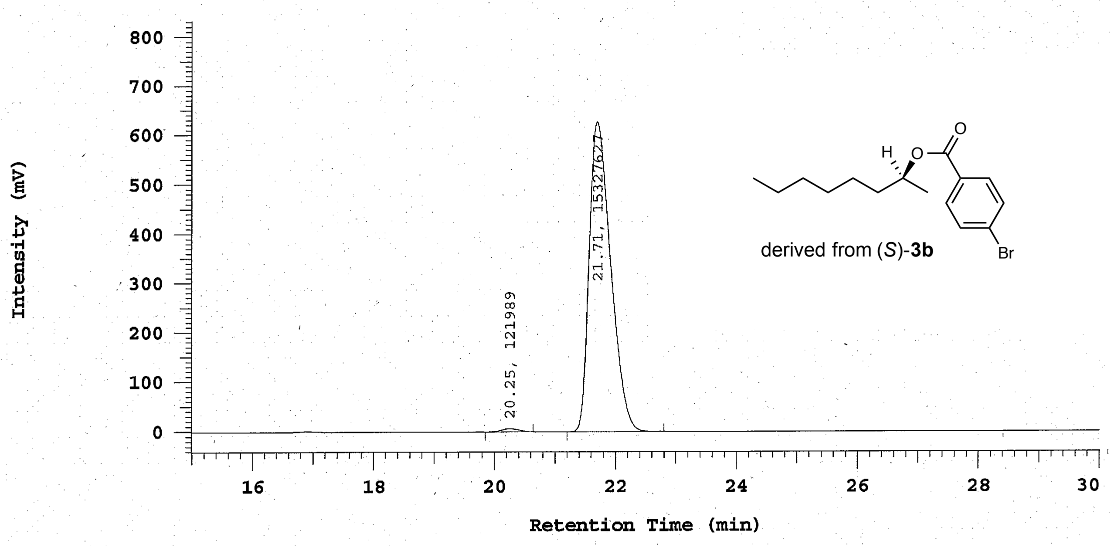

Processing Method: 0.1/99.9 iPrOH/Hexane
Column Type: ~~OD-H-2~~ OZ-3
Method Developer: Administrator
Pump A: L-2130
Pump A Solvent A: Hexane
Pump A Solvent B: 10/90 iPrOH/Hexane
Pump A Solvent C: iPrOH
Pump A Solvent D: EtOH
Method Description:

Chrom Type: HPLC Channel : 1
Peak Quantitation: AREA
Calculation Method: AREA%

No.	RT	Area	Area %
1	20.25	121989	0.790
2	21.71	15327627	99.210
		15449616	100.000

Peak rejection level: 0

Supplementary Figure 69. HPLC trace of the ester derived from (*S*)-**3b**.

D-2000: Isocratic HPLC Series: 1691 Report Name: modified System: Sys 1

D-2000 Elite HPLC System Manager Report

Analyzed Date and Time: 2017/11/07 13:04 Reported Date and Time: 2018/01/23 16:20

Processed Date and Time: 2018/01/23 16:20

Data Path: C:\WIN32APP\D2000HSM\Isocratic\DATA\1691\

Processing Method: 0.5/99.5 iPrOH/Hexane

System (acquisition): Sys 1 Series: 1691

Application(data): Isocratic HPLC Vial Number: 163

Sample Name: IWA-1582-rac Vial Type: UNK

Injection from this vial: 1 of 1 Volume: 10.0 ul

Sample Description:

Chrom Type: HPLC Channel : 1

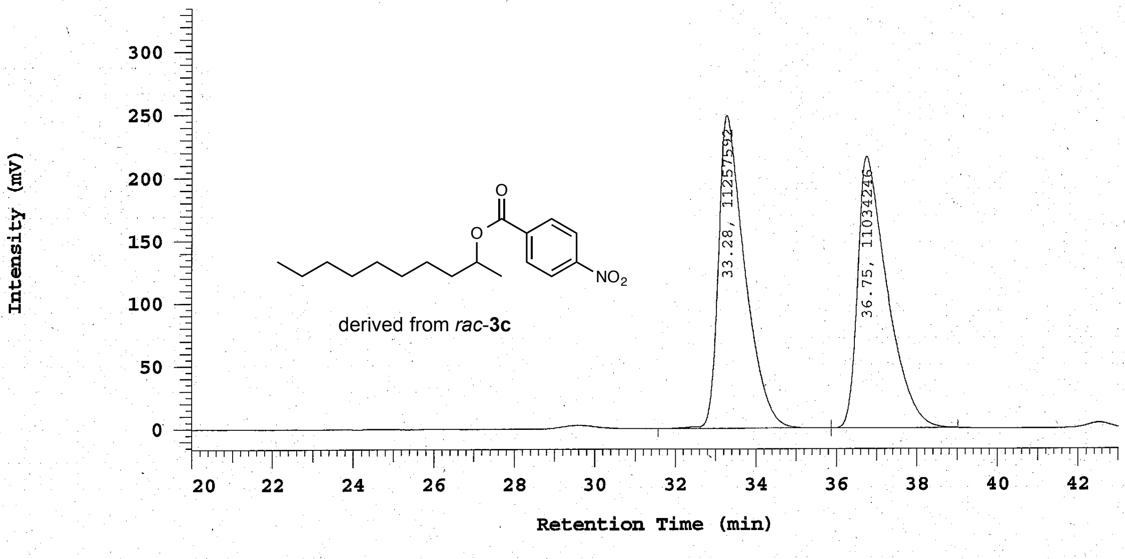

Processing Method: 0.5/99.5 iPrOH/Hexane

Column Type: ~~[illegible]~~2 IC-3 Method Developer: Administrator

Pump A: L-2130

Pump A Solvent A: Hexane Pump A Solvent B: 10/90 iPrOH/Hexane

Pump A Solvent C: iPrOH Pump A Solvent D: EtOH

Method Description:

Chrom Type: HPLC Channel : 1

Peak Quantitation: AREA

Calculation Method: AREA%

No.	RT	Area	Area %
1	33.28	11257592	50.501
2	36.75	11034246	49.499
		22291838	100.000

Peak rejection level: 0

Supplementary Figure 70. HPLC trace of the ester derived from *rac*-**3c**.

D-2000: Isocratic HPLC Series: 1689 Report Name: modified System: Sys 1

D-2000 Elite HPLC System Manager Report

Analyzed Date and Time: 2017/11/07 10:38
Reported Date and Time: 2018/01/23 16:24
Processed Date and Time: 2018/01/23 16:24
Data Path: C:\WIN32APP\D2000HSM\Isocratic\DATA\1689\
Processing Method: 0.5/99.5 iPrOH/Hexane
System (acquisition): Sys 1
Series: 1689
Application(data): Isocratic HPLC
Vial Number: 161
Sample Name: IwA-1582-rac-0711
Vial Type: UNK
Injection from this vial: 1 of 1
Volume: 10.0 ul
Sample Description:

Chrom Type: HPLC Channel : 1

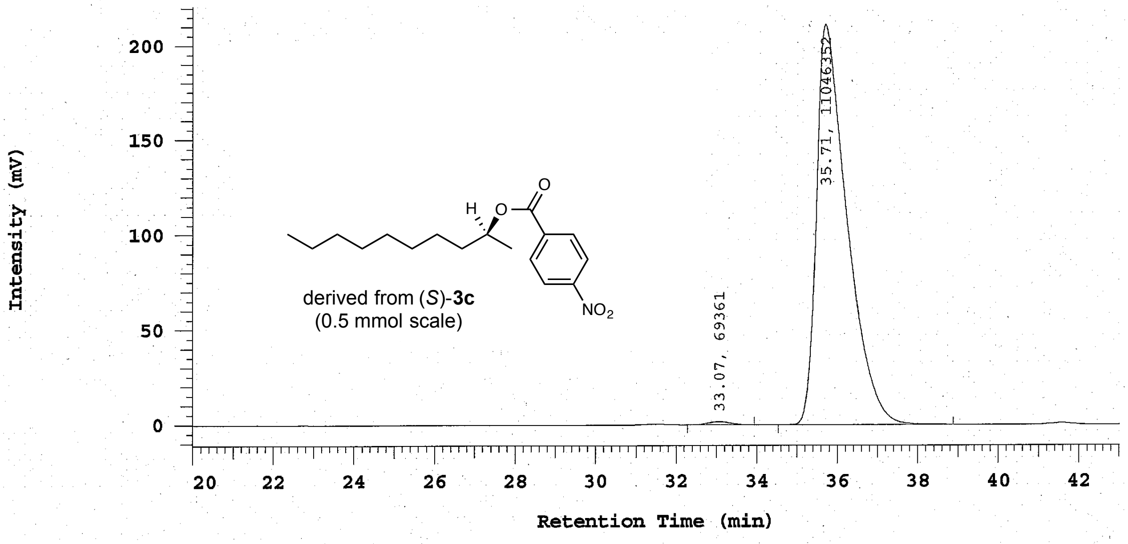

Processing Method: 0.5/99.5 iPrOH/Hexane
Column Type: ~~OD-H 2~~ IC-3
Method Developer: Administrator
Pump A: L-2130
Pump A Solvent A: Hexane
Pump A Solvent B: 10/90 iPrOH/Hexane
Pump A Solvent C: iPrOH
Pump A Solvent D: EtOH
Method Description:

Chrom Type: HPLC Channel : 1
Peak Quantitation: AREA
Calculation Method: AREA%

No.	RT	Area	Area %
1	33.07	69361	0.624
2	35.71	11046352	99.376
		11115713	100.000

Peak rejection level: 0

Supplementary Figure 71. HPLC trace of the ester derived from (*S*)-**3c** (0.5 mmol scale).

D-2000: Isocratic HPLC Series: 1690 Report Name: modified System: Sys 1

D-2000 Elite HPLC System Manager Report

Analyzed Date and Time: 2017/11/07 11:56 Reported Date and Time: 2018/01/23 16:23

Processed Date and Time: 2018/01/23 16:23

Data Path: C:\WIN32APP\D2000HSM\Isocratic\DATA\1690\

Processing Method: 0.5/99.5 iPrOH/Hexane

System (acquisition): Sys 1 Series: 1690

Application(data): Isocratic HPLC Vial Number: 162

Sample Name: IWA-1705-5mmol scale Vial Type: UNK

Injection from this vial: 1 of 1 Volume: 10.0 ul

Sample Description:

Chrom Type: HPLC Channel : 1

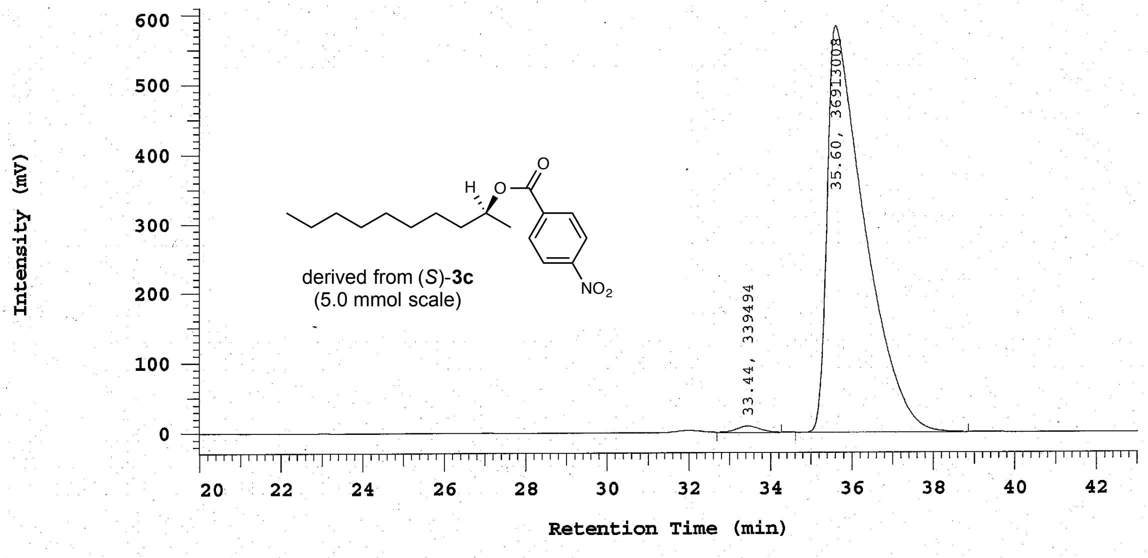

Processing Method: 0.5/99.5 iPrOH/Hexane

Column Type: ~~OD-H-2~~ IC-3 Method Developer: Administrator

Pump A: L-2130

Pump A Solvent A: Hexane Pump A Solvent B: 10/90 iPrOH/Hexane

Pump A Solvent C: iPrOH Pump A Solvent D: EtOH

Method Description:

Chrom Type: HPLC Channel : 1

Peak Quantitation: AREA

Calculation Method: AREA%

No.	RT	Area	Area %
1	33.44	339494	0.911
2	35.60	36913008	99.089
		37252502	100.000

Peak rejection level: 0

Supplementary Figure 72. HPLC trace of the ester derived from (*S*)-**3c** (5.0 mmol scale).

D-2000: Isocratic HPLC Series: 1791 Report Name: modified System: Sys 1

D-2000 Elite HPLC System Manager Report

Analyzed Date and Time: 2017/12/16 16:54 Reported Date and Time: 2017/12/16 18:48

Processed Date and Time: 2017/12/16 18:48

Data Path: C:\WIN32APP\D2000HSM\Isocratic\DATA\1791\

Processing Method: 0.1/99.9 iPrOH/Hexane

System (acquisition): Sys 1 Series: 1791

Application(data): Isocratic HPLC Vial Number: 162

Sample Name: IWA-1743-rac Vial Type: UNK

Injection from this vial: 1 of 1 Volume: 10.0 ul

Sample Description:

Chrom Type: HPLC Channel : 1

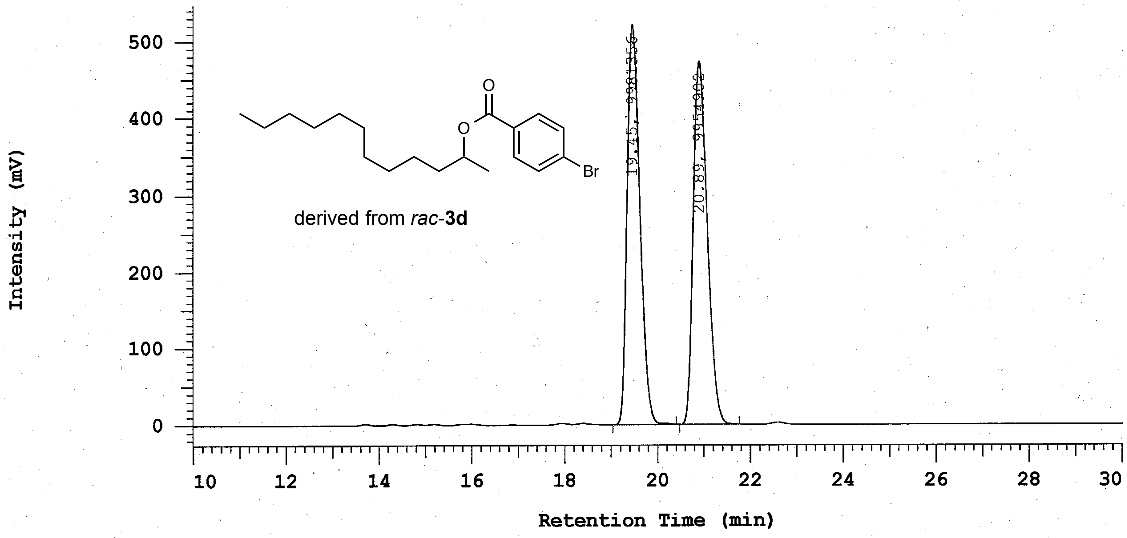

Processing Method: 0.1/99.9 iPrOH/Hexane

Column Type: ~~OD-H-2~~ OZ-3 Method Developer: Administrator

Pump A: L-2130

Pump A Solvent A: Hexane Pump A Solvent B: 10/90 iPrOH/Hexane

Pump A Solvent C: iPrOH Pump A Solvent D: EtOH

Method Description:

Chrom Type: HPLC Channel : 1

Peak Quantitation: AREA

Calculation Method: AREA%

No.	RT	Area	Area %
1	19.45	9981356	50.066
2	20.89	9954902	49.934
		19936258	100.000

Peak rejection level: 0

Supplementary Figure 73. HPLC trace of the ester derived from *rac*-**3d**.

D-2000: Isocratic HPLC Series: 1792 Report Name: modified System: Sys 1

D-2000 Elite HPLC System Manager Report

Analyzed Date and Time: 2017/12/16 17:55
Reported Date and Time: 2017/12/16 18:47
Processed Date and Time: 2017/12/16 18:47
Data Path: C:\WIN32APP\D2000HSM\Isocratic\DATA\1792\
Processing Method: 0.1/99.9 iPrOH/Hexane
System (acquisition): Sys 1
Series: 1792
Application(data): Isocratic HPLC
Vial Number: 161
Sample Name: IWA-1745-t-ODAd-40-3
Vial Type: UNK
Injection from this vial: 1 of 1
Volume: 10.0 ul
Sample Description:

Chrom Type: HPLC Channel : 1

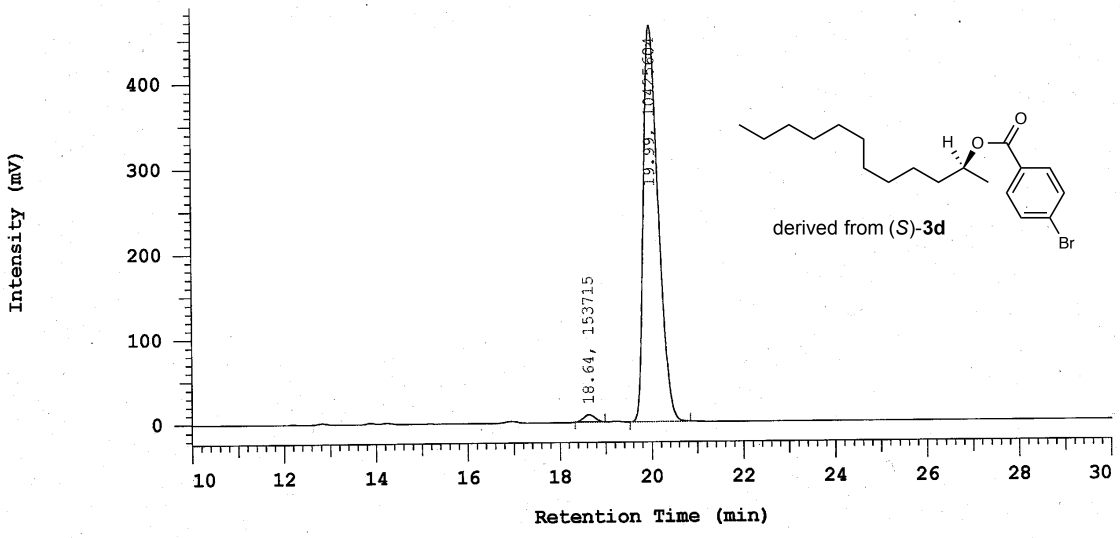

Processing Method: 0.1/99.9 iPrOH/Hexane
Column Type: ~~OD-H~~2 OZ-3
Method Developer: Administrator
Pump A: L-2130
Pump A Solvent A: Hexane
Pump A Solvent B: 10/90 iPrOH/Hexane
Pump A Solvent C: iPrOH
Pump A Solvent D: EtOH
Method Description:

Chrom Type: HPLC Channel : 1
Peak Quantitation: AREA
Calculation Method: AREA%

No.	RT	Area	Area %
1	18.64	153715	1.453
2	19.99	10425604	98.547
		10579319	100.000

Peak rejection level: 0

Supplementary Figure 74. HPLC trace of the ester derived from (*S*)-**3d**.

D-2000: Isocratic HPLC Series: 1492 Report Name: modified System: Sys 1

D-2000 Elite HPLC System Manager Report

Analyzed Date and Time: 2017/07/23 21:35
Reported Date and Time: 2018/01/23 21:53
Processed Date and Time: 2018/01/23 21:53
Data Path: C:\WIN32APP\D2000HSM\Isocratic\DATA\1492\
Processing Method: 05/95 iPrOH/Hexane
System (acquisition): Sys 1
Series: 1492
Application(data): Isocratic HPLC
Vial Number: 171
Sample Name: IWA-1388-rac
Vial Type: UNK
Injection from this vial: 1 of 1
Volume: 10.0 ul
Sample Description:

Chrom Type: HPLC Channel : 1

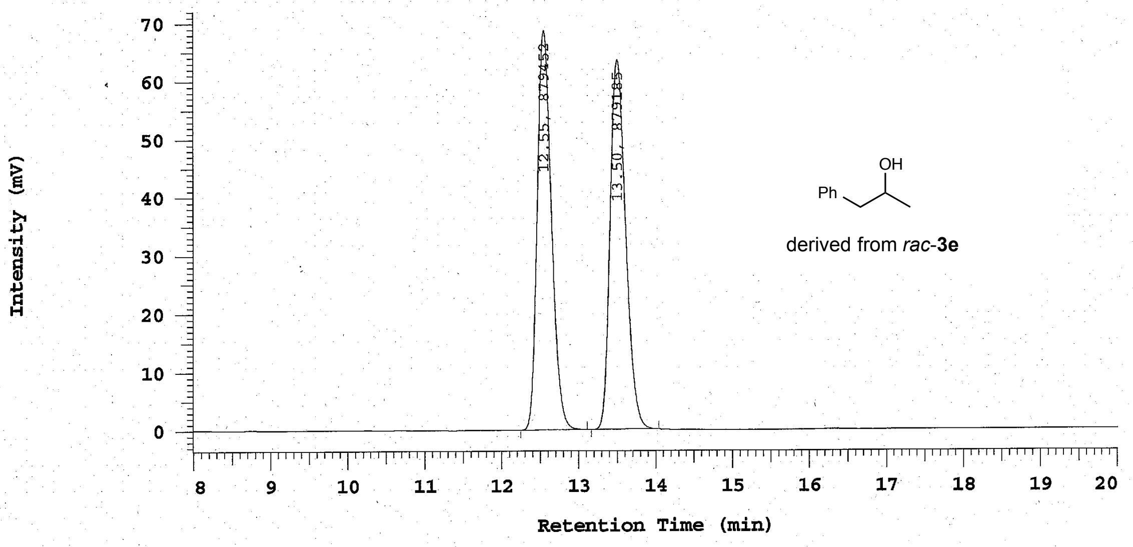

Processing Method: 05/95 iPrOH/Hexane
Column Type: ~~OD-H~~2 OD-3
Method Developer: Administrator
Pump A: L-2130
Pump A Solvent A: Hexane
Pump A Solvent B: 10/90 iPrOH/Hexane
Pump A Solvent C: iPrOH
Pump A Solvent D: iPrOH
Method Description:

Chrom Type: HPLC Channel : 1
Peak Quantitation: AREA
Calculation Method: AREA%

No.	RT	Area	Area %
1	12.55	879452	50.008
2	13.50	879185	49.992
		1758637	100.000

Peak rejection level: 0

Supplementary Figure 75. HPLC trace of the alcohol derived from *rac*-**3e**.

D-2000: Isocratic HPLC Series: 1493 Report Name: modified System: Sys 1

D-2000 Elite HPLC System Manager Report

Analyzed Date and Time: 2017/07/23 22:43 Reported Date and Time: 2018/01/23 21:53

Processed Date and Time: 2018/01/23 21:53

Data Path: C:\WIN32APP\D2000HSM\Isocratic\DATA\1493\

Processing Method: 05/95 iPrOH/Hexane

System (acquisition): Sys 1 Series: 1493

Application(data): Isocratic HPLC Vial Number: 172

Sample Name: IWA-1597-S-Quinox-tODAd-40-5 Vial Type: UNK

Volume: 10.0 ul

Injection from this vial: 1 of 1

Sample Description:

Chrom Type: HPLC Channel : 1

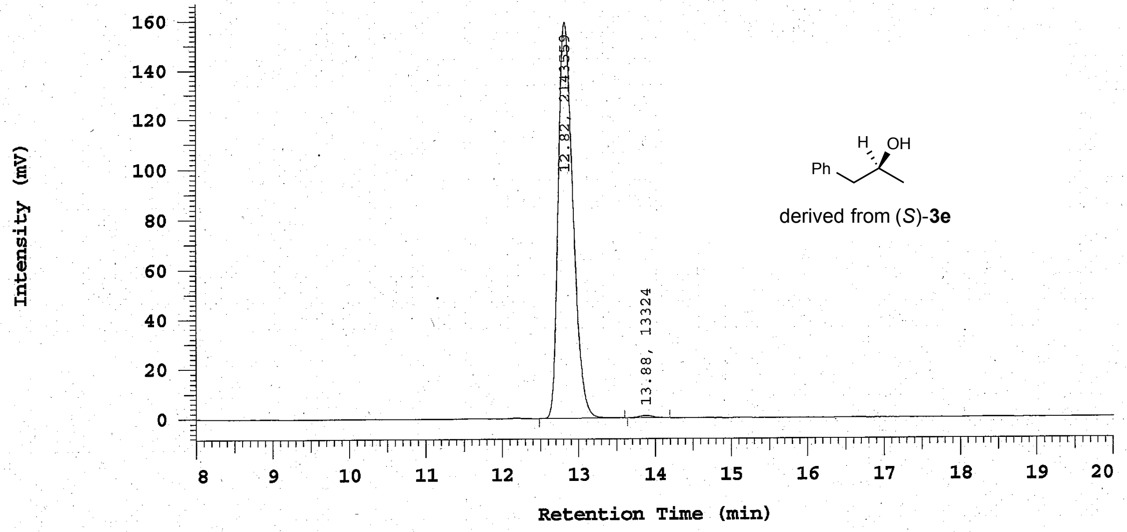

Processing Method: 05/95 iPrOH/Hexane

Column Type: ~~[illegible]~~ OD-3 Method Developer: Administrator

Pump A: L-2130

Pump A Solvent A: Hexane Pump A Solvent B: 10/90 iPrOH/Hexane

Pump A Solvent C: iPrOH Pump A Solvent D: iPrOH

Method Description:

Chrom Type: HPLC Channel : 1

Peak Quantitation: AREA

Calculation Method: AREA%

No.	RT	Area	Area %
1	12.82	2143559	99.382
2	13.88	13324	0.618
		2156883	100.000

Peak rejection level: 0

Supplementary Figure 76. HPLC trace of the alcohol derived from (*S*)-**3e**.

D-2000: Isocratic HPLC Series: 1674 Report Name: modified System: Sys 1

D-2000 Elite HPLC System Manager Report

Analyzed Date and Time: 2017/11/01 19:32
Reported Date and Time: 2018/01/23 21:57
Processed Date and Time: 2018/01/23 21:57
Data Path: C:\WIN32APP\D2000HSM\Isocratic\DATA\1674\
Processing Method: 0.5/99.5 iPrOH/Hexane

System (acquisition): Sys 1
Series: 1674
Application(data): Isocratic HPLC
Vial Number: 161
Sample Name: IWA-1659-rac
Vial Type: UNK
Injection from this vial: 1 of 1
Volume: 10.0 ul
Sample Description:

Chrom Type: HPLC Channel : 1

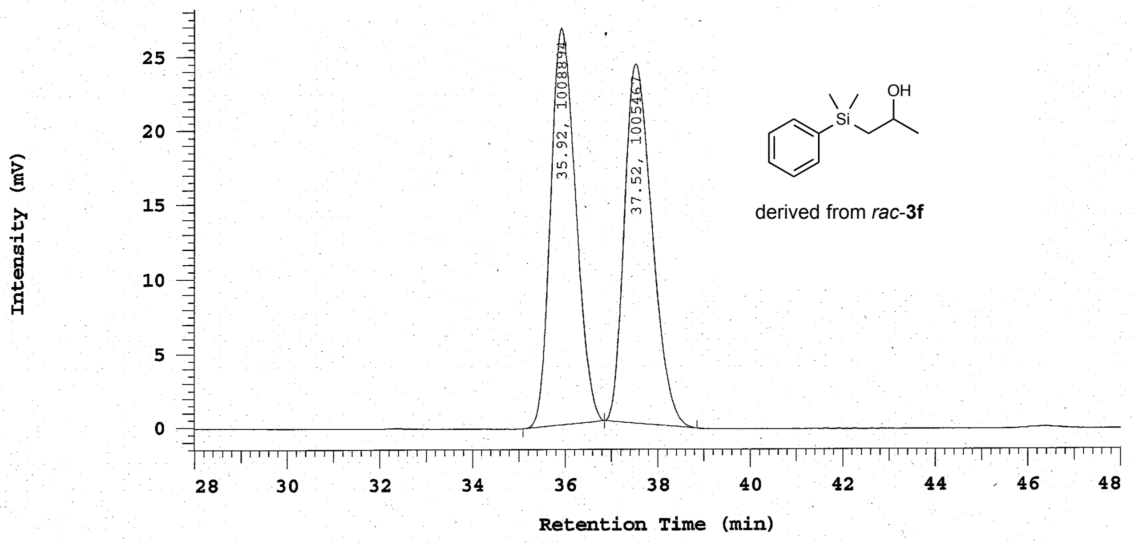

Processing Method: 0.5/99.5 iPrOH/Hexane
Column Type: ~~OD-H 2~~ OZ-3
Method Developer: Administrator
Pump A: L-2130
Pump A Solvent A: Hexane
Pump A Solvent B: 10/90 iPrOH/Hexane
Pump A Solvent C: iPrOH
Pump A Solvent D: EtOH
Method Description:

Chrom Type: HPLC Channel : 1
Peak Quantitation: AREA
Calculation Method: AREA%

No.	RT	Area	Area %
1	35.92	1008894	50.085
2	37.52	1005467	49.915
		2014361	100.000

Peak rejection level: 0

Supplementary Figure 77. HPLC trace of the alcohol derived from *rac*-**3f**.

D-2000: Isocratic HPLC Series: 1675 Report Name: modified System: Sys 1

D-2000 Elite HPLC System Manager Report

Analyzed Date and Time: 2017/11/01 20:56
Reported Date and Time: 2018/01/23 21:57
Processed Date and Time: 2018/01/23 21:57
Data Path: C:\WIN32APP\D2000HSM\Isocratic\DATA\1675\
Processing Method: 0.5/99.5 iPrOH/Hexane
System (acquisition): Sys 1
Series: 1675
Application(data): Isocratic HPLC
Vial Number: 162
Sample Name: IWA-1699-S-QuinoxtODAd-20
Vial Type: UNK
Injection from this vial: 1 of 1
Volume: 10.0 ul
Sample Description:

Chrom Type: HPLC Channel : 1

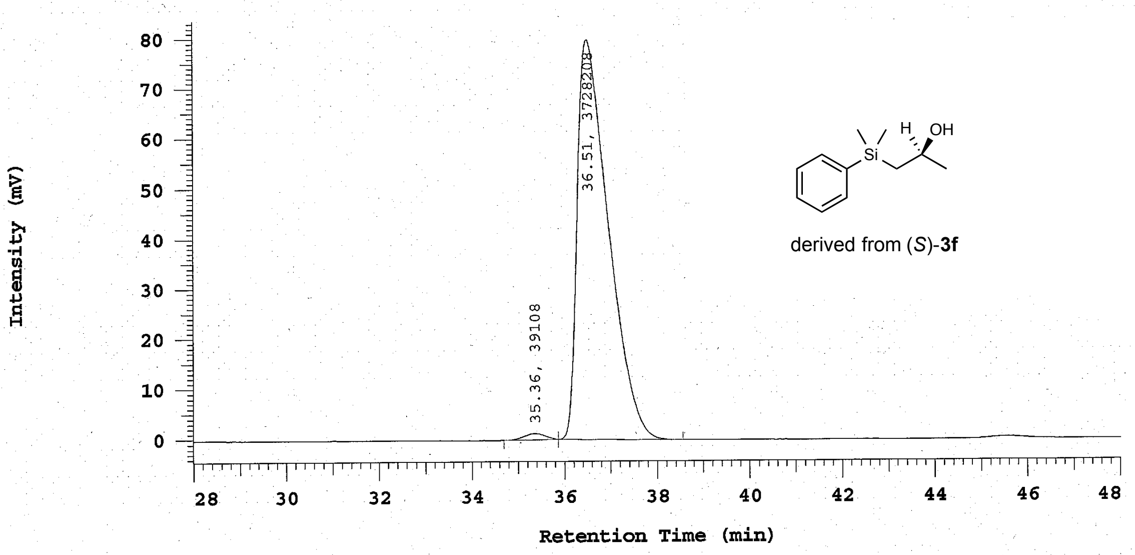

Processing Method: 0.5/99.5 iPrOH/Hexane
Column Type: ~~OD-H-2~~ OZ-3
Method Developer: Administrator
Pump A: L-2130
Pump A Solvent A: Hexane
Pump A Solvent B: 10/90 iPrOH/Hexane
Pump A Solvent C: iPrOH
Pump A Solvent D: EtOH
Method Description:

Chrom Type: HPLC Channel : 1
Peak Quantitation: AREA
Calculation Method: AREA%

No.	RT	Area	Area %
1	35.36	39108	1.038
2	36.51	3728208	98.962
		3767316	100.000

Peak rejection level: 0

Supplementary Figure 78. HPLC trace of the alcohol derived from (*S*)-**3f**.

D-2000: Isocratic HPLC Series: 1501 Report Name: modified System: Sys 1

D-2000 Elite HPLC System Manager Report

Analyzed Date and Time: 2017/07/26 10:17 Reported Date and Time: 2018/01/23 21:59

Processed Date and Time: 2018/01/23 21:59

Data Path: C:\WIN32APP\D2000HSM\Isocratic\DATA\1501\

Processing Method: 01/99 iPrOH/Hexane

System (acquisition): Sys 1 Series: 1501

Application(data): Isocratic HPLC Vial Number: 171

Sample Name: IWA-1542-rac Vial Type: UNK

Injection from this vial: 1 of 1 Volume: 10.0 ul

Sample Description:

Chrom Type: HPLC Channel : 1

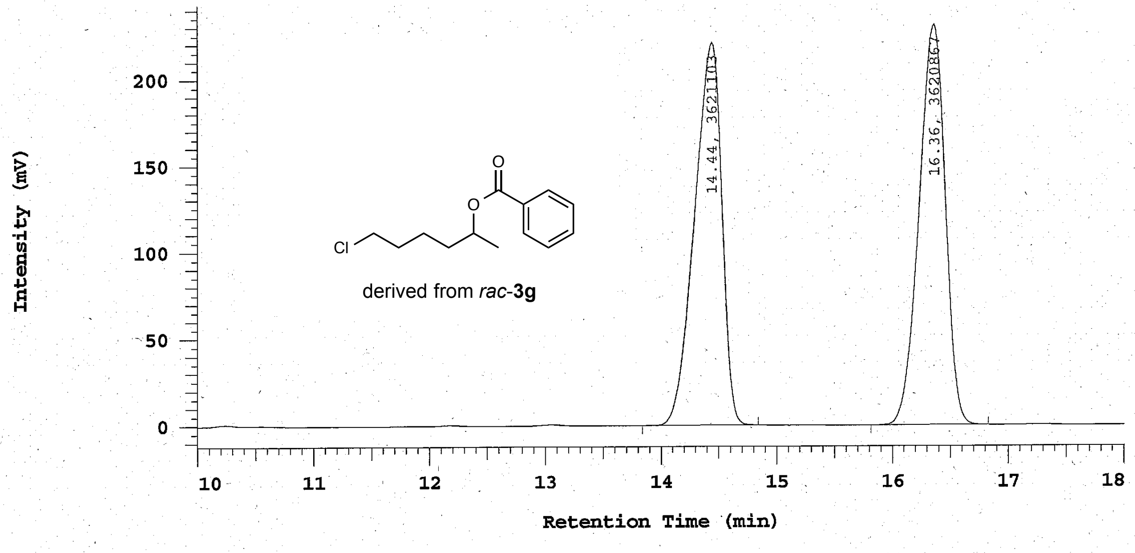

Processing Method: 01/99 iPrOH/Hexane

Column Type: ~~OD-H2~~ AD-3 Method Developer: Administrator

Pump A: L-2130

Pump A Solvent A: Hexane Pump A Solvent B: 10/90 iPrOH/Hexane

Pump A Solvent C: iPrOH Pump A Solvent D: EtOH

Method Description:

Chrom Type: HPLC Channel : 1

Peak Quantitation: AREA

Calculation Method: AREA%

No.	RT	Area	Area %
1	14.44	3621103	50.002
2	16.36	3620867	49.998
		7241970	100.000

Peak rejection level: 0

Supplementary Figure 79. HPLC trace of the ester derived from *rac*-**3g**.

D-2000: Isocrati c HPLC   Series: 1502   Report Name: modified   System: Sys 1

D-2000 Elite HPLC System Manager Report

Analyzed Date and Time: 2017/07/26 11:41   Reported Date and Time: 2018/01/23 21:59

Processed Date and Time: 2018/01/23 21:59

Data Path: C:\WIN32APP\D2000HSM\Isocratic\DATA\1502\

Processing Method: 01/99 iPrOH/Hexane

System (acquisition): Sys 1   Series: 1502

Application(data): Isocratic HPLC   Vial Number: 172

Sample Name: IWA-1604-S-QuinoxtODAd-40-5   Vial Type: UNK

Volume: 10.0 ul

Injection from this vial: 1 of 1

Sample Description:

Chrom Type: HPLC Channel : 1

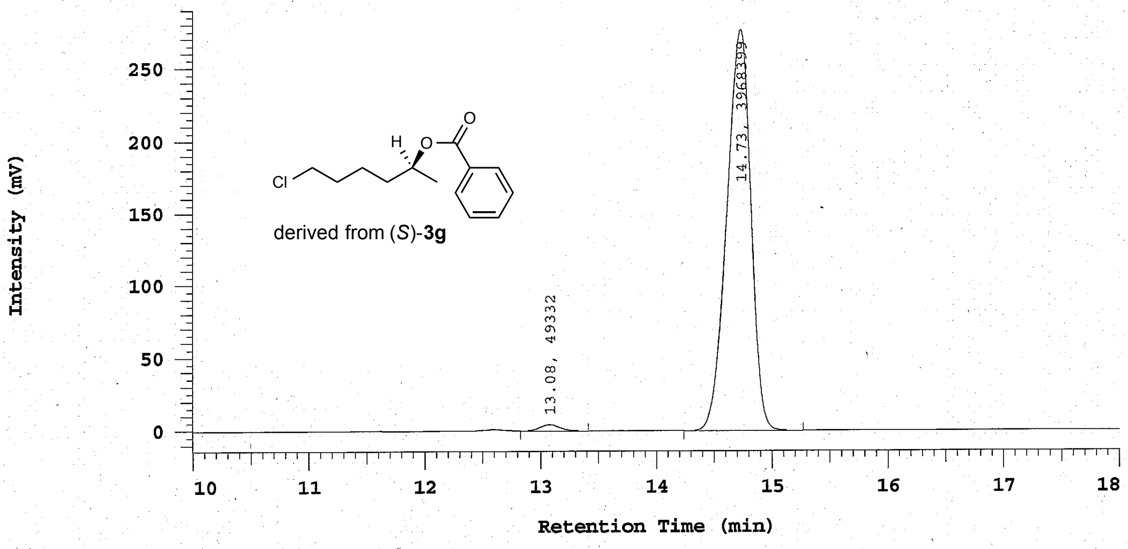

Processing Method: 01/99 iPrOH/Hexane

Column Type: ~~OD-H-2~~ AD-3   Method Developer: Administrator

Pump A: L-2130

Pump A Solvent A: Hexane   Pump A Solvent B: 10/90 iPrOH/Hexane

Pump A Solvent C: iPrOH   Pump A Solvent D: EtOH

Method Description:

Chrom Type: HPLC Channel : 1

Peak Quantitation: AREA

Calculation Method: AREA%

No.	RT	Area	Area %
1	13.08	49332	1.228
2	14.73	3968399	98.772
		4017731	100.000

Peak rejection level: 0

Supplementary Figure 80. HPLC trace of the ester derived from (*S*)-**3g**.

D-2000: Isocratic HPLC Series: 1565 Report Name: modified System: Sys 1

D-2000 Elite HPLC System Manager Report

Analyzed Date and Time: 2017/08/30 19:57
Reported Date and Time: 2018/01/23 22:01
Processed Date and Time: 2018/01/23 22:01
Data Path: C:\WIN32APP\D2000HSM\Isocratic\DATA\1565\
Processing Method: 01/99 iPrOH/Hexane

System (acquisition): Sys 1
Application(data): Isocratic HPLC
Sample Name: IWA-1638-OD-1%-1
Injection from this vial: 1 of 1
Sample Description:

Series: 1565
Vial Number: 171
Vial Type: UNK
Volume: 10.0 ul

Chrom Type: HPLC Channel : 1

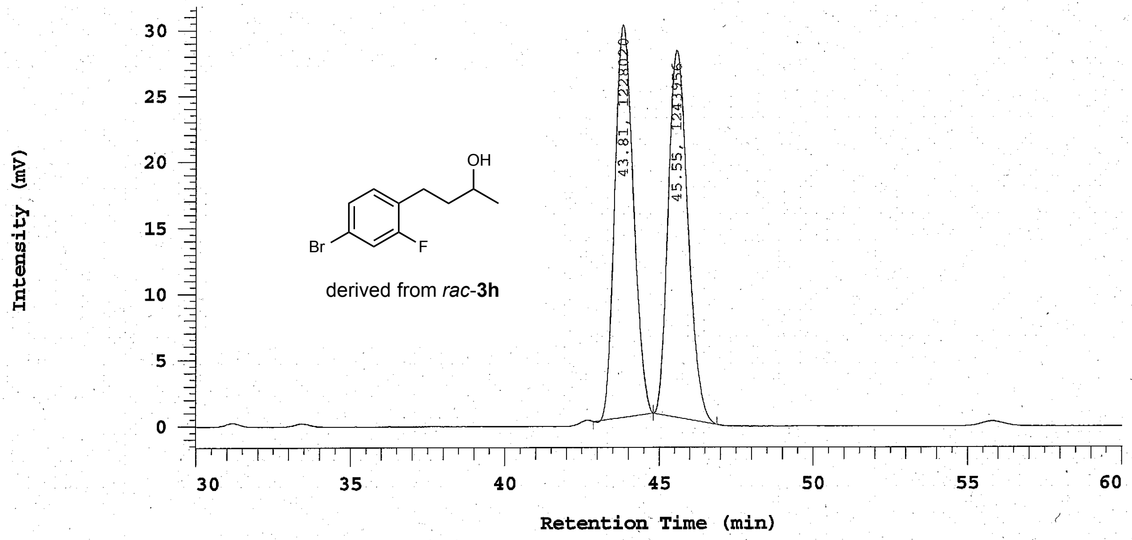

Processing Method: 01/99 iPrOH/Hexane
Column Type: ~~OD-H-2~~ OD-3
Method Developer: Administrator
Pump A: L-2130
Pump A Solvent A: Hexane
Pump A Solvent B: 10/90 iPrOH/Hexane
Pump A Solvent C: iPrOH
Pump A Solvent D: EtOH
Method Description:

Chrom Type: HPLC Channel : 1
Peak Quantitation: AREA
Calculation Method: AREA%

No.	RT	Area	Area %
1	43.81	1228020	49.678
2	45.55	1243956	50.322
		2471976	100.000

Peak rejection level: 0

Supplementary Figure 81. HPLC trace of the alcohol derived from *rac*-**3h**.

D-2000: Isocratic HPLC Series: 1566 Report Name: modified System: Sys 1

D-2000 Elite HPLC System Manager Report

Analyzed Date and Time: 2017/08/30 21:18
Reported Date and Time: 2018/01/23 22:02
Processed Date and Time: 2018/01/23 22:01
Data Path: C:\WIN32APP\D2000HSM\Isocratic\DATA\1566\
Processing Method: 01/99 iPrOH/Hexane
System (acquisition): Sys 1
Series: 1566
Application(data): Isocratic HPLC
Vial Number: 172
Sample Name: IWA-1642-S-QuinoxtODAd-40-3-A
Vial Type: UNK
Volume: 10.0 ul
Injection from this vial: 1 of 1
Sample Description:

Chrom Type: HPLC Channel : 1

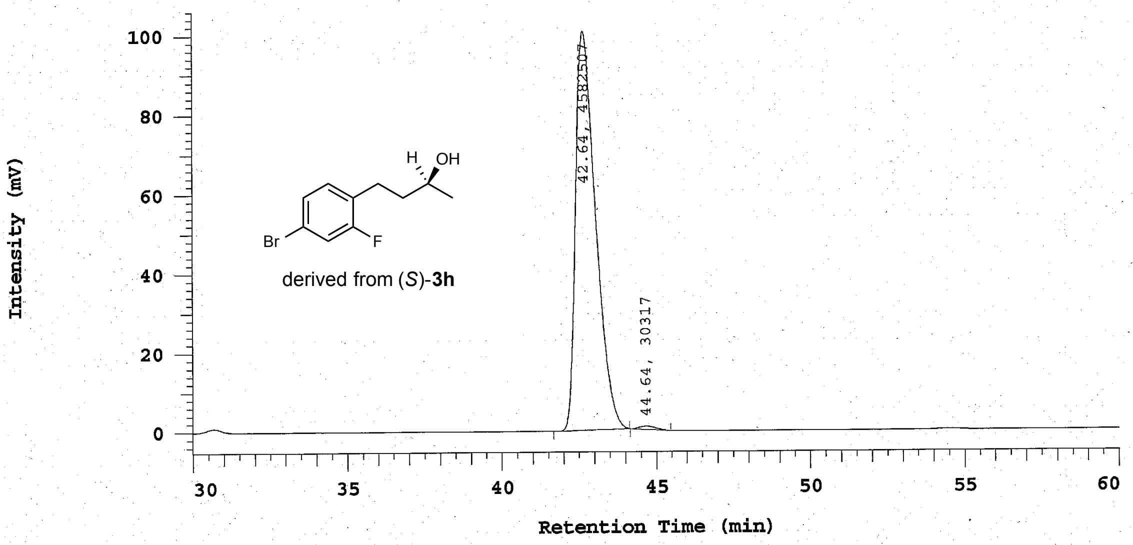

Processing Method: 01/99 iPrOH/Hexane
Column Type: ~~OD-H~~2 OD-3
Method Developer: Administrator
Pump A: L-2130
Pump A Solvent A: Hexane
Pump A Solvent B: 10/90 iPrOH/Hexane
Pump A Solvent C: iPrOH
Pump A Solvent D: EtOH
Method Description:

Chrom Type: HPLC Channel : 1
Peak Quantitation: AREA
Calculation Method: AREA%

No.	RT	Area	Area %
1	42.64	4582507	99.343
2	44.64	30317	0.657
		4612824	100.000

Peak rejection level: 0

Supplementary Figure 82. HPLC trace of the alcohol derived from (*S*)-**3h**.

D-2000 Elite HPLC System Manager Report

Analyzed Date and Time: 2017/08/06 16:09
Reported Date and Time: 2018/01/17 16:03
Processed Date and Time: 2018/01/17 16:02
Data Path: C:\WIN32APP\D2000HSM\Isocratic\DATA\1536\
Processing Method: 01/99 iPrOH/Hexane
System (acquisition): Sys 1
Series: 1536
Application(data): Isocratic HPLC
Vial Number: 171
Sample Name: IWA-1624-rac
Vial Type: UNK
Injection from this vial: 1 of 1
Volume: 10.0 ul
Sample Description:

Chrom Type: HPLC Channel : 1

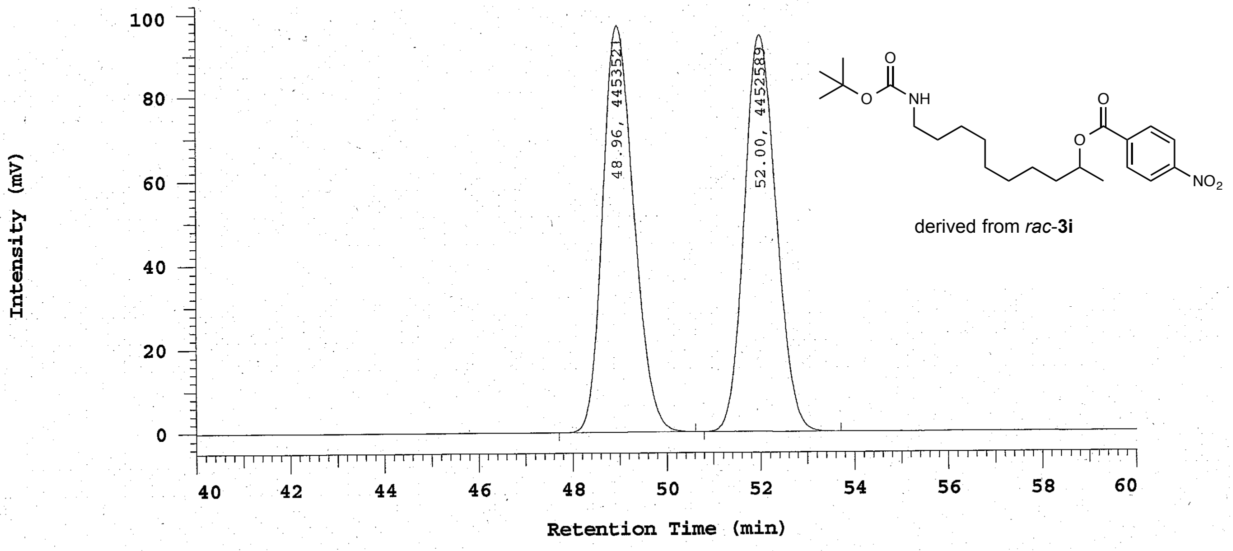

Processing Method: 01/99 iPrOH/Hexane
Column Type: ~~OD-H~~2 AD-3
Method Developer: Administrator
Pump A: L-2130
Pump A Solvent A: Hexane
Pump A Solvent B: 10/90 iPrOH/Hexane
Pump A Solvent C: iPrOH
Pump A Solvent D: iPrOH
Method Description:

Chrom Type: HPLC Channel : 1
Peak Quantitation: AREA
Calculation Method: AREA%

No.	RT	Area	Area %
1	48.96	4453521	50.005
2	52.00	4452589	49.995
		8906110	100.000

Peak rejection level: 0

Supplementary Figure 83. HPLC trace of the ester derived from *rac*-**3i**.

D-2000 Elite HPLC System Manager Report

Analyzed Date and Time: 2017/08/06 17:42
Reported Date and Time: 2018/01/17 16:03
Processed Date and Time: 2018/01/17 16:03
Data Path: C:\WIN32APP\D2000HSM\Isocratic\DATA\1537\
Processing Method: 01/99 iPrOH/Hexane
System (acquisition): Sys 1
Series: 1537
Application(data): Isocratic HPLC
Vial Number: 172
Sample Name: IWA-1624-S-QuinoxTODAD-10-5
Vial Type: UNK
Volume: 10.0 ul
Injection from this vial: 1 of 1
Sample Description:

Chrom Type: HPLC Channel : 1

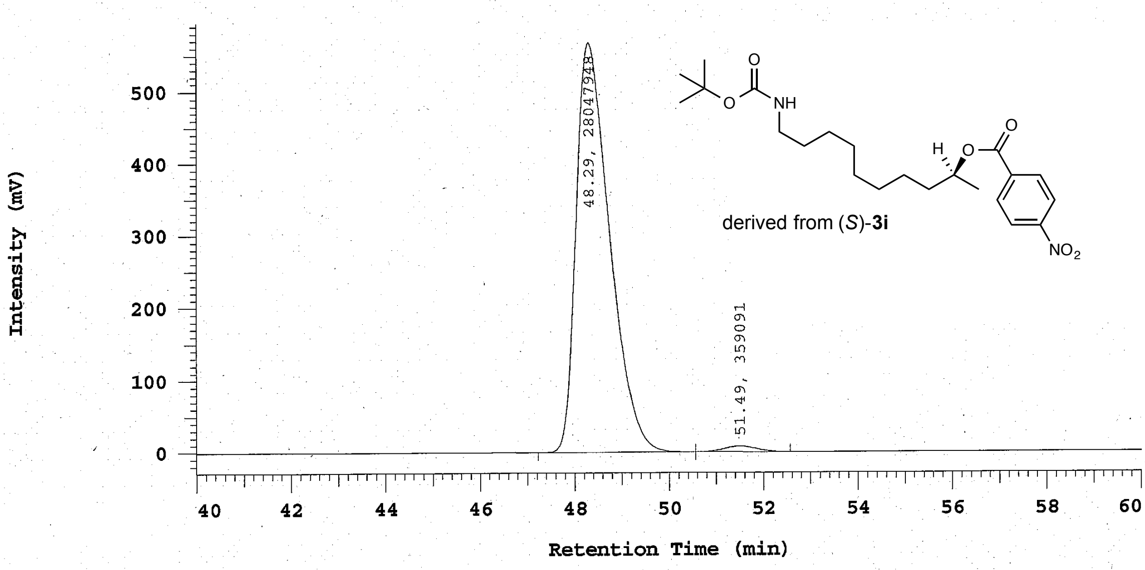

Processing Method: 01/99 iPrOH/Hexane
Column Type: ~~OD-H-2~~ AD-3
Method Developer: Administrator
Pump A: L-2130
Pump A Solvent A: Hexane
Pump A Solvent B: 10/90 iPrOH/Hexane
Pump A Solvent C: iPrOH
Pump A Solvent D: iPrOH
Method Description:

Chrom Type: HPLC Channel : 1
Peak Quantitation: AREA
Calculation Method: AREA%

No.	RT	Area	Area %
1	48.29	28047948	98.736
2	51.49	359091	1.264
		28407039	100.000

Peak rejection level: 0

Supplementary Figure 84. HPLC trace of the ester derived from (*S*)-**3i**.

D-2000: Isocratic HPLC Series: 1542 Report Name: modified System: Sys 1

D-2000 Elite HPLC System Manager Report

Analyzed Date and Time: 2017/08/24 19:16
Reported Date and Time: 2018/01/23 22:06
Processed Date and Time: 2018/01/23 22:06
Data Path: C:\WIN32APP\D2000HSM\Isocratic\DATA\1542\
Processing Method: 02/98 iPrOH/Hexane

System (acquisition): Sys 1
Application(data): Isocratic HPLC
Sample Name: IWA-1615-rac
Injection from this vial: 1 of 1
Sample Description:

Series: 1542
Vial Number: 171
Vial Type: UNK
Volume: 10.0 ul

Chrom Type: HPLC Channel : 1

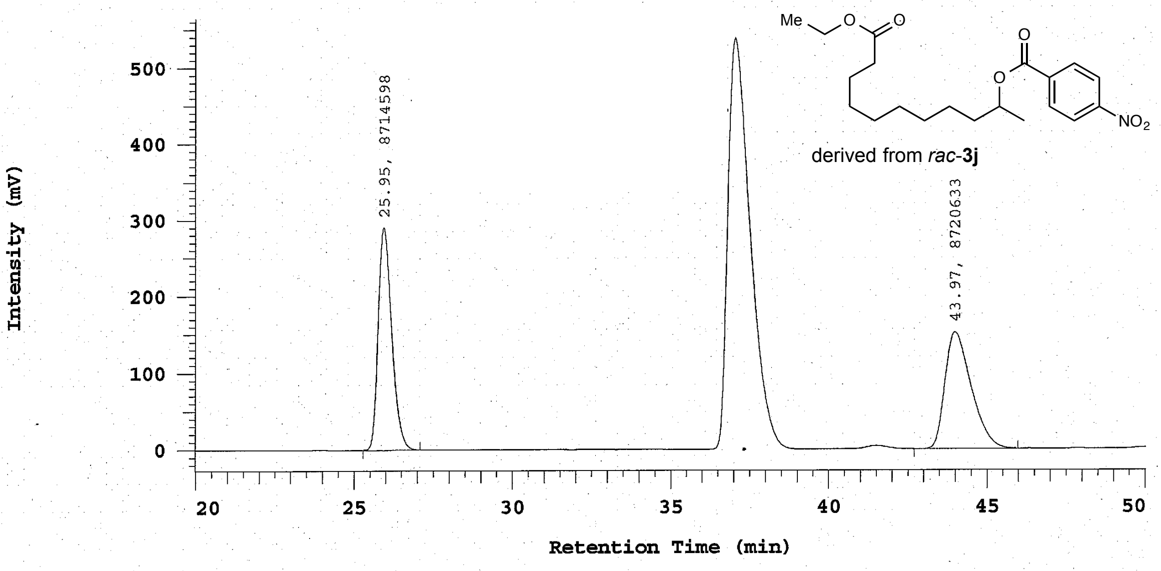

Processing Method: 02/98 iPrOH/Hexane
Column Type: ~~OD-H~~2 OJ-3
Method Developer: Administrator
Pump A: L-2130
Pump A Solvent A: Hexane
Pump A Solvent B: 10/90 iPrOH/Hexane
Pump A Solvent C: iPrOH
Pump A Solvent D: EtOH
Method Description:

Chrom Type: HPLC Channel : 1
Peak Quantitation: AREA
Calculation Method: AREA%

No.	RT	Area	Area %
1	25.95	8714598	49.983
2	43.97	8720633	50.017
		17435231	100.000

Peak rejection level: 0

Supplementary Figure 85. HPLC trace of the ester derived from *rac*-**3j**.

D-2000: Isocratic HPLC Series: 1543 Report Name: modified System: Sys 1

D-2000 Elite HPLC System Manager Report

Analyzed Date and Time: 2017/08/24 20:28
Reported Date and Time: 2018/01/23 22:07
Processed Date and Time: 2018/01/23 22:07
Data Path: C:\WIN32APP\D2000HSM\Isocratic\DATA\1543\
Processing Method: 02/98 iPrOH/Hexane
System (acquisition): Sys 1
Series: 1543
Application(data): Isocratic HPLC
Vial Number: 172
Sample Name: IWA-1629-S-QuinoxtODAd-40-5
Vial Type: UNK
Volume: 10.0 ul
Injection from this vial: 1 of 1
Sample Description:

Chrom Type: HPLC Channel : 1

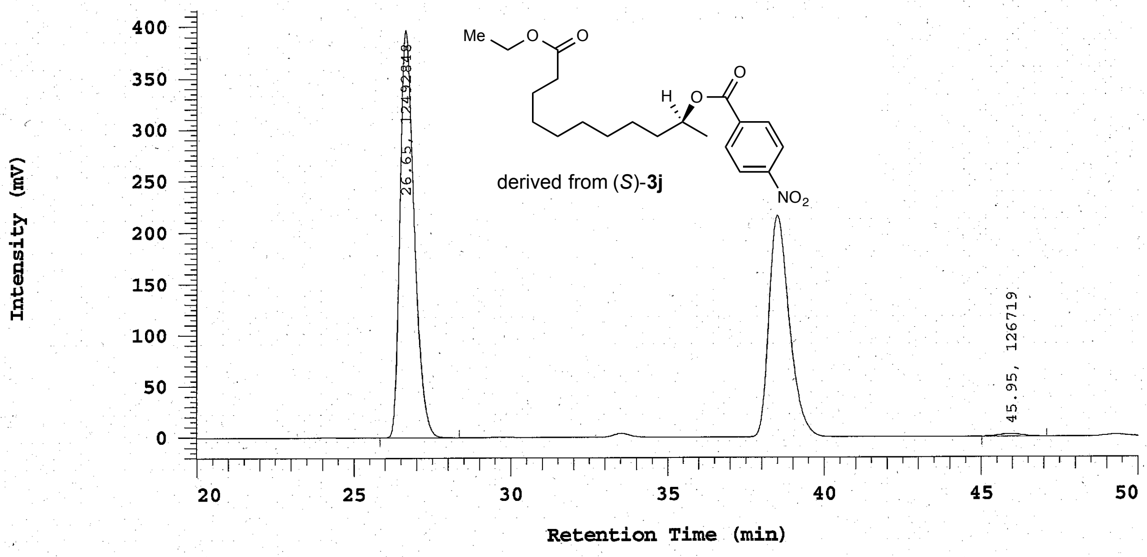

Processing Method: 02/98 iPrOH/Hexane
Column Type: ~~OD-H-2~~ OJ-3
Method Developer: Administrator
Pump A: L-2130
Pump A Solvent A: Hexane
Pump A Solvent B: 10/90 iPrOH/Hexane
Pump A Solvent C: iPrOH
Pump A Solvent D: EtOH
Method Description:

Chrom Type: HPLC Channel : 1
Peak Quantitation: AREA
Calculation Method: AREA%

No.	RT	Area	Area %
1	26.65	12492848	98.996
2	45.95	126719	1.004
		12619567	100.000

Peak rejection level: 0

Supplementary Figure 86. HPLC trace of the ester derived from (*S*)-**3j**.

D-2000 Elite HPLC System Manager Report

Analyzed Date and Time: 2017/08/26 10:00
Reported Date and Time: 2018/01/17 16:15
Processed Date and Time: 2018/01/17 16:15
Data Path: C:\WIN32APP\D2000HSM\Isocratic\DATA\1549\
Processing Method: 01/99 iPrOH/Hexane
System (acquisition): Sys 1
Application(data): Isocratic HPLC
Sample Name: IWA-1633-S-QuinoxtODAd-40-5
Injection from this vial: 1 of 1
Sample Description:
Series: 1549
Vial Number: 171
Vial Type: UNK
Volume: 10.0 ul

Chrom Type: HPLC Channel : 1

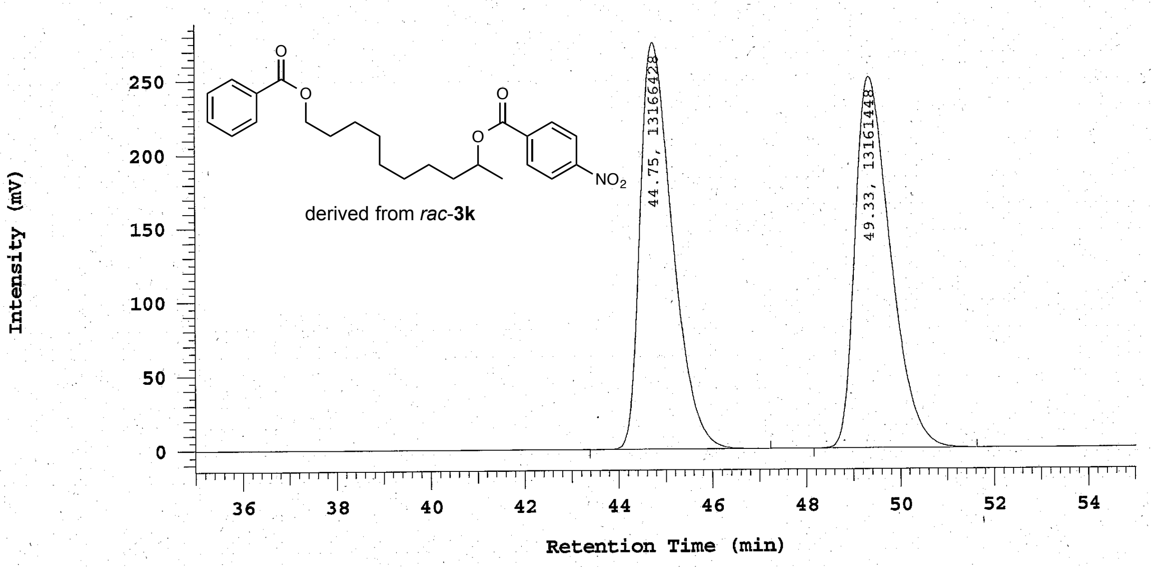

Processing Method: 01/99 iPrOH/Hexane
Column Type: ~~OD-H-2~~ OD-3
Method Developer: Administrator
Pump A: L-2130
Pump A Solvent A: Hexane
Pump A Solvent B: 10/90 iPrOH/Hexane
Pump A Solvent C: iPrOH
Pump A Solvent D: EtOH
Method Description:

Chrom Type: HPLC Channel : 1
Peak Quantitation: AREA
Calculation Method: AREA%

No.	RT	Area	Area %
1	44.75	13166428	50.009
2	49.33	13161448	49.991
		26327876	100.000

Peak rejection level: 0

Supplementary Figure 87. HPLC trace of the ester derived from *rac*-**3k**.

D-2000: Isocratic HPLC Series: 1550 Report Name: modified System: Sys 1

D-2000 Elite HPLC System Manager Report

Analyzed Date and Time: 2017/08/26 11:06 Reported Date and Time: 2018/01/17 16:15

Processed Date and Time: 2018/01/17 16:15

Data Path: C:\WIN32APP\D2000HSM\Isocratic\DATA\1550\

Processing Method: 01/99 iPrOH/Hexane

System (acquisition): Sys 1 Series: 1550

Application(data): Isocratic HPLC Vial Number: 172

Sample Name: IWA-1633-s-QuinoxtODAd-40-5-c Vial Type: UNK

Volume: 10.0 ul

Injection from this vial: 1 of 1

Sample Description:

Chrom Type: HPLC Channel : 1

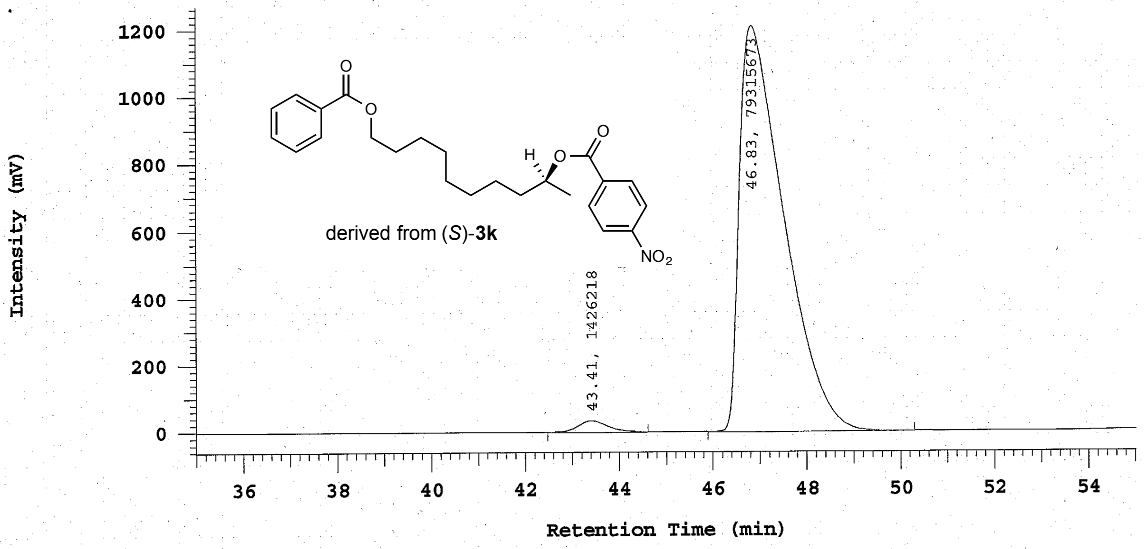

Processing Method: 01/99 iPrOH/Hexane

Column Type: ~~OD-H2~~ OD-3 Method Developer: Administrator

Pump A: L-2130

Pump A Solvent A: Hexane Pump A Solvent B: 10/90 iPrOH/Hexane

Pump A Solvent C: iPrOH Pump A Solvent D: EtOH

Method Description:

Chrom Type: HPLC Channel : 1

Peak Quantitation: AREA

Calculation Method: AREA%

No.	RT	Area	Area %
1	43.41	1426218	1.766
2	46.83	79315673	98.234
		80741891	100.000

Peak rejection level: 0

Supplementary Figure 88. HPLC trace of the ester derived from (*S*)-**3k**.

D-2000 Elite HPLC System Manager Report

Analyzed Date and Time: 2017/08/04 11:08 Reported Date and Time: 2018/01/23 22:13

Processed Date and Time: 2018/01/23 22:13

Data Path: C:\WIN32APP\D2000HSM\Isocratic\DATA\1531\

Processing Method: 02/98 iPrOH/Hexane

System (acquisition): Sys 1 Series: 1531

Application(data): Isocratic HPLC Vial Number: 171

Sample Name: IWA-1609-rac Vial Type: UNK

Injection from this vial: 1 of 1 Volume: 10.0 ul

Sample Description:

Chrom Type: HPLC Channel : 1

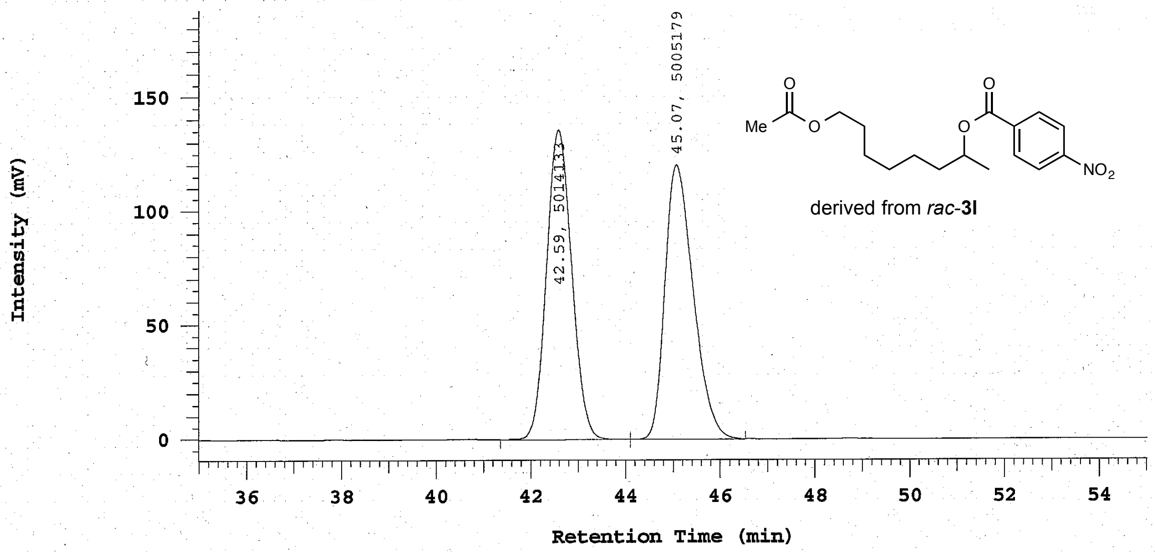

Processing Method: 02/98 iPrOH/Hexane

Column Type: ~~OD-H-2~~ AD-3 Method Developer: Administrator

Pump A: L-2130

Pump A Solvent A: Hexane Pump A Solvent B: 10/90 iPrOH/Hexane

Pump A Solvent C: iPrOH Pump A Solvent D: EtOH

Method Description:

Chrom Type: HPLC Channel : 1

Peak Quantitation: AREA

Calculation Method: AREA%

No.	RT	Area	Area %
1	42.59	5014133	50.045
2	45.07	5005179	49.955
		10019312	100.000

Peak rejection level: 0

Supplementary Figure 89. HPLC trace of the ester derived from *rac*-**3l**.

D-2000: Isocratic HPLC Series: 1532 Report Name: modified System: Sys 1

D-2000 Elite HPLC System Manager Report

Analyzed Date and Time: 2017/08/04 12:29 Reported Date and Time: 2018/01/23 22:14
Processed Date and Time: 2018/01/23 22:13
Data Path: C:\WIN32APP\D2000HSM\Isocratic\DATA\1532\
Processing Method: 02/98 iPrOH/Hexane

System (acquisition): Sys 1
Application(data): Isocratic HPLC
Sample Name: IWA-1621-S-QuinoxtODAd-40-5
Injection from this vial: 1 of 1
Sample Description:

Series: 1532
Vial Number: 172
Vial Type: UNK
Volume: 10.0 ul

Chrom Type: HPLC Channel : 1

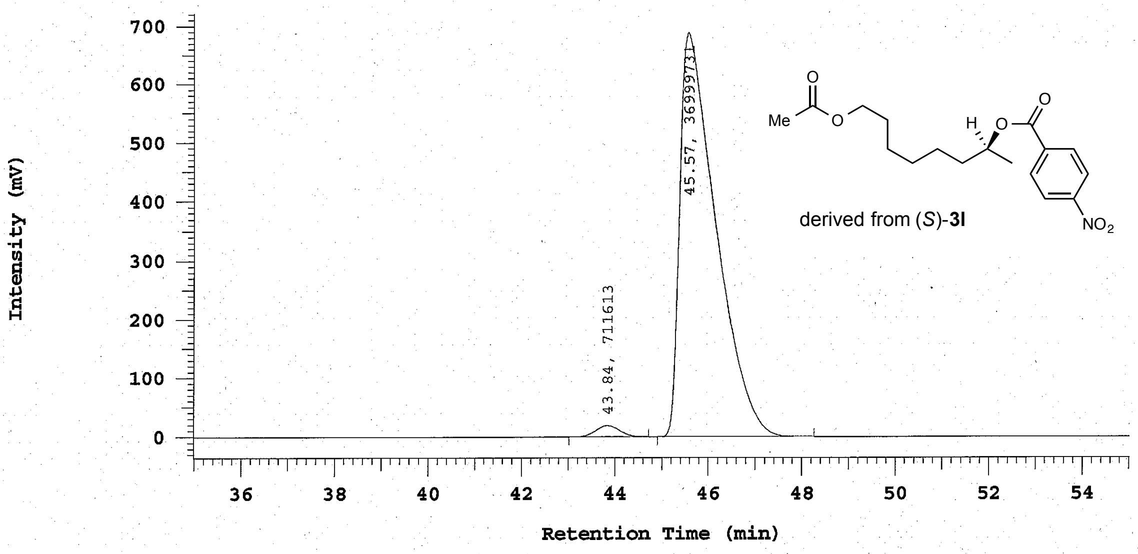

Processing Method: 02/98 iPrOH/Hexane
Column Type: ~~OD-H~~2 AD-3 Method Developer: Administrator
Pump A: L-2130
Pump A Solvent A: Hexane
Pump A Solvent B: 10/90 iPrOH/Hexane
Pump A Solvent C: iPrOH
Pump A Solvent D: EtOH
Method Description:

Chrom Type: HPLC Channel : 1
Peak Quantitation: AREA
Calculation Method: AREA%

No.	RT	Area	Area %
1	43.84	711613	1.887
2	45.57	36999731	98.113
		37711344	100.000

Peak rejection level: 0

Supplementary Figure 90. HPLC trace of the ester derived from (*S*)-**3l**.

D-2000: Isocratic HPLC Series: 1598 Report Name: modified System: Sys 1

D-2000 Elite HPLC System Manager Report

Analyzed Date and Time: 2017/09/19 10:47
Reported Date and Time: 2018/01/23 22:24
Processed Date and Time: 2018/01/23 22:24
Data Path: C:\WIN32APP\D2000HSM\Isocratic\DATA\1598\
Processing Method: 0.5/99.5 iPrOH/Hexane

System (acquisition): Sys 1
Application(data): Isocratic HPLC
Sample Name: IWA-1657-rac
Injection from this vial: 1 of 1
Sample Description:

Series: 1598
Vial Number: 171
Vial Type: UNK
Volume: 10.0 ul

Chrom Type: HPLC Channel : 1

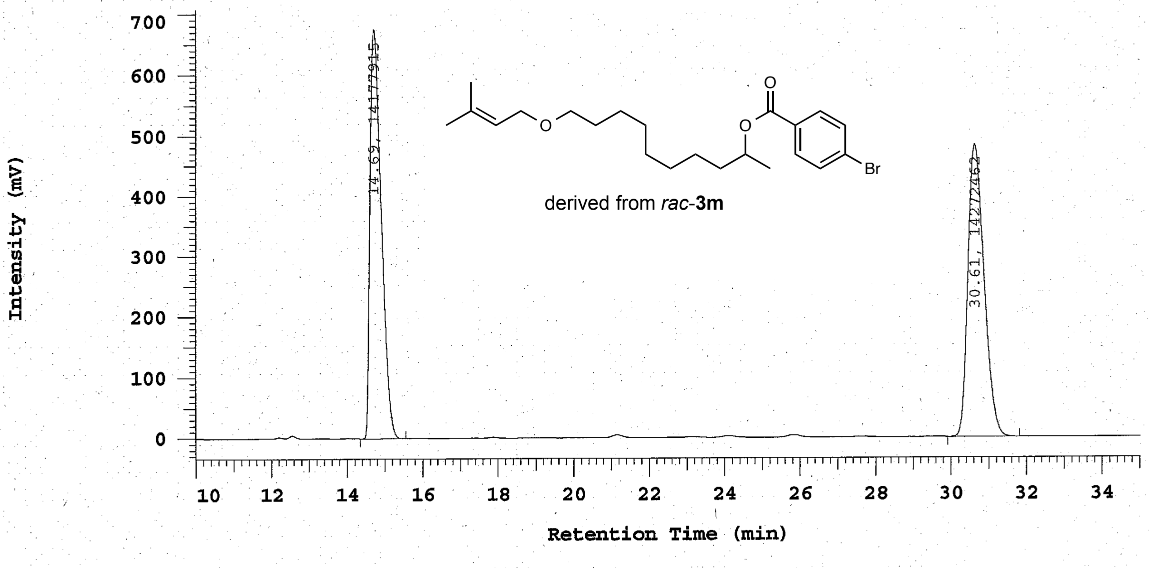

Processing Method: 0.5/99.5 iPrOH/Hexane
Column Type: ~~OD-H-2~~ OD-3
Method Developer: Administrator
Pump A: L-2130
Pump A Solvent A: Hexane
Pump A Solvent B: 10/90 iPrOH/Hexane
Pump A Solvent C: iPrOH
Pump A Solvent D: EtOH
Method Description:

Chrom Type: HPLC Channel : 1
Peak Quantitation: AREA
Calculation Method: AREA%

No.	RT	Area	Area %
1	14.69	14177915	49.834
2	30.61	14272462	50.166
		28450377	100.000

Peak rejection level: 0

Supplementary Figure 91. HPLC trace of the ester derived from *rac*-**3m**.

D-2000: Isocratic HPLC Series: 1599 Report Name: modified System: Sys 1

D-2000 Elite HPLC System Manager Report

Analyzed Date and Time: 2017/09/19 12:02 Reported Date and Time: 2018/01/23 22:25

Processed Date and Time: 2018/01/23 22:25

Data Path: C:\WIN32APP\D2000HSM\Isocratic\DATA\1599\

Processing Method: 0.5/99.5 iPrOH/Hexane

System (acquisition): Sys 1 Series: 1599

Application(data): Isocratic HPLC Vial Number: 172

Sample Name: IWA-1664 Vial Type: UNK

Injection from this vial: 1 of 1 Volume: 10.0 ul

Sample Description:

Chrom Type: HPLC Channel : 1

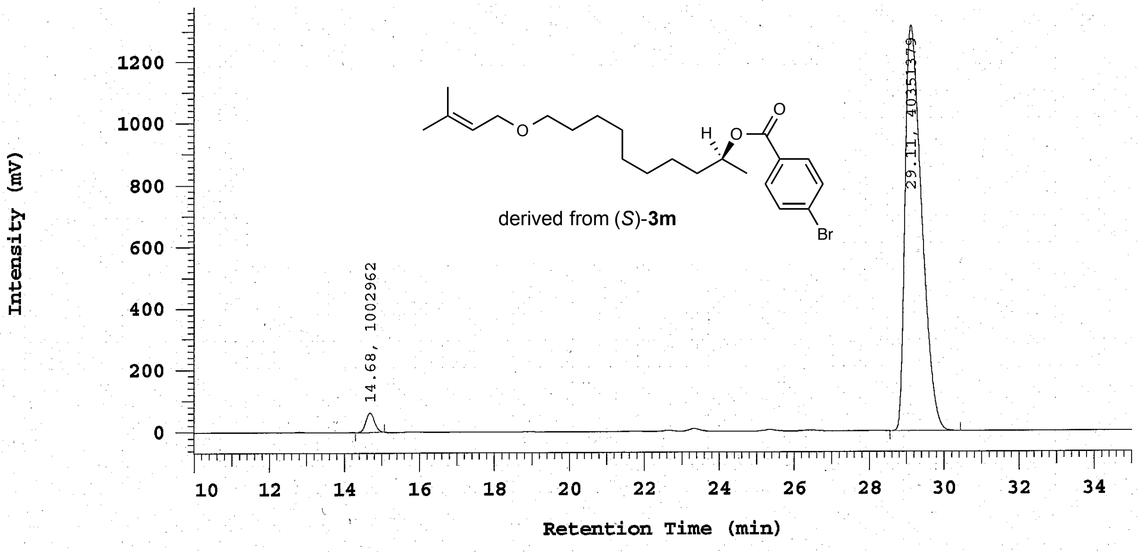

Processing Method: 0.5/99.5 iPrOH/Hexane

Column Type: ~~OD-H 2~~ OD-3 Method Developer: Administrator

Pump A: L-2130

Pump A Solvent A: Hexane Pump A Solvent B: 10/90 iPrOH/Hexane

Pump A Solvent C: iPrOH Pump A Solvent D: EtOH

Method Description:

Chrom Type: HPLC Channel : 1

Peak Quantitation: AREA

Calculation Method: AREA%

No.	RT	Area	Area %
1	14.68	1002962	2.425
2	29.11	40351379	97.575
		41354341	100.000

Peak rejection level: 0

Supplementary Figure 92. HPLC trace of the ester derived from (*S*)-**3m**.

D-2000: Isocratic HPLC Series: 1529 Report Name: modified System: Sys 1

D-2000 Elite HPLC System Manager Report

Analyzed Date and Time: 2017/08/03 23:59
Reported Date and Time: 2018/01/23 22:26
Processed Date and Time: 2018/01/23 22:26
Data Path: C:\WIN32APP\D2000HSM\Isocratic\DATA\1529\
Processing Method: 03/97 iPrOH/Hexane

System (acquisition): Sys 1
Series: 1529
Application(data): Isocratic HPLC
Vial Number: 171
Sample Name: IWA-1671-rac
Vial Type: UNK
Injection from this vial: 1 of 1
Volume: 10.0 ul
Sample Description:

Chrom Type: HPLC Channel : 1

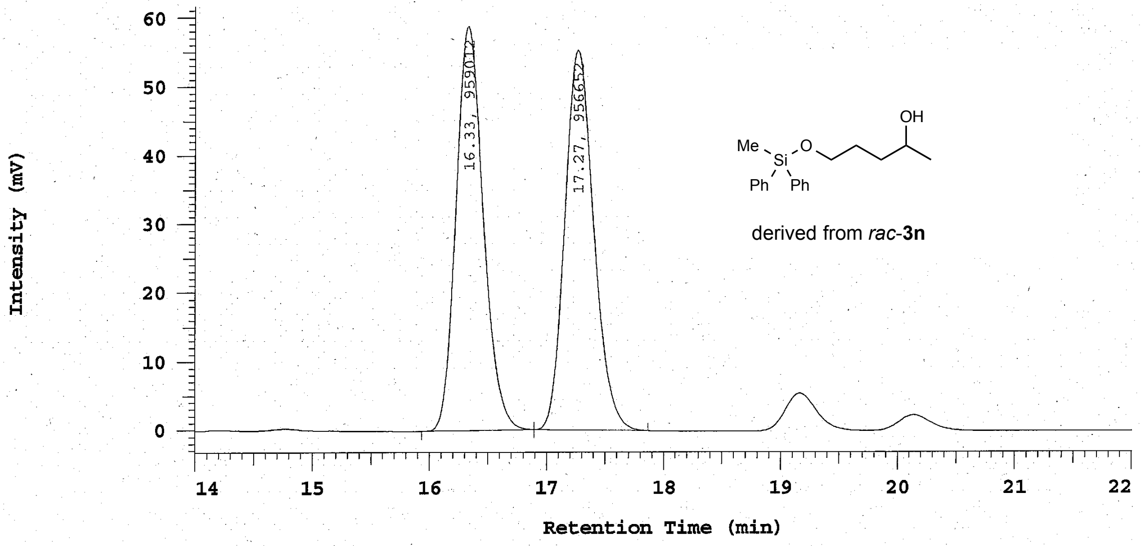

Processing Method: 03/97 iPrOH/Hexane
Column Type: ~~OD-H-2~~ OD-3
Method Developer: Administrator
Pump A: L-2130
Pump A Solvent A: Hexane
Pump A Solvent B: 10/90 iPrOH/Hexane
Pump A Solvent C: iPrOH
Pump A Solvent D: iPrOH
Method Description:

Chrom Type: HPLC Channel : 1
Peak Quantitation: AREA
Calculation Method: AREA%

No.	RT	Area	Area %
1	16.33	959012	50.062
2	17.27	956652	49.938
		1915664	100.000

Peak rejection level: 0

Supplementary Figure 93. HPLC trace of the alcohol derived from *rac*-**3n**.

D-2000: Isocratic HPLC Series: 1530 Report Name: modified System: Sys 1

D-2000 Elite HPLC System Manager Report

Analyzed Date and Time: 2017/08/04 01:00
Reported Date and Time: 2018/01/23 22:27
Processed Date and Time: 2018/01/23 22:27
Data Path: C:\WIN32APP\D2000HSM\Isocratic\DATA\1530\
Processing Method: 03/97 iPrOH/Hexane
System (acquisition): Sys 1
Series: 1530
Application(data): Isocratic HPLC
Vial Number: 172
Sample Name: IWA-1618-S-QuinoxtODAd-40-5
Vial Type: UNK
Volume: 10.0 ul
Injection from this vial: 1 of 1
Sample Description:

Chrom Type: HPLC Channel : 1

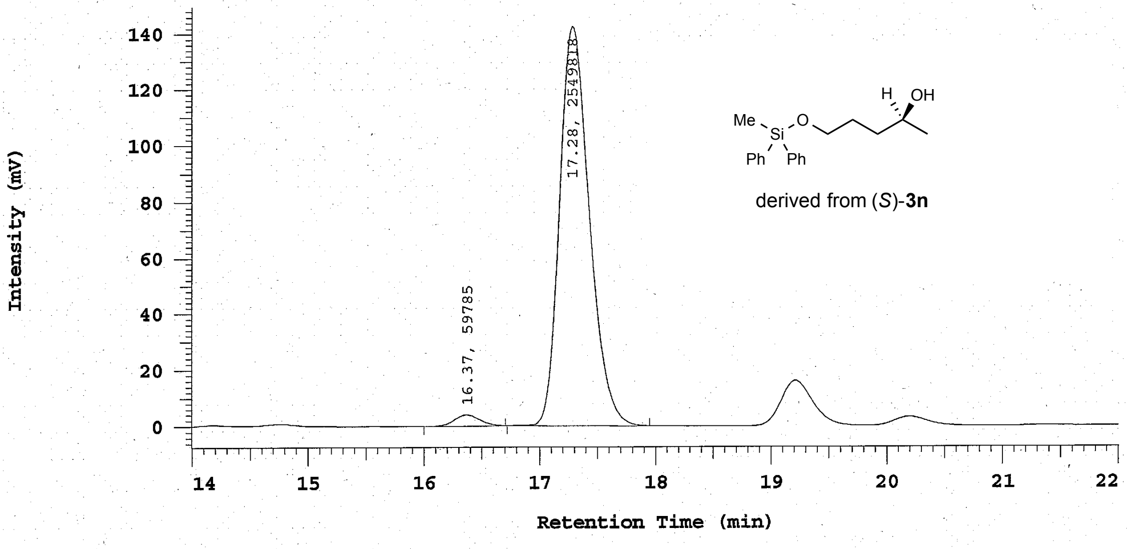

Processing Method: 03/97 iPrOH/Hexane
Column Type: ~~OD-H~~ 2 OD-3
Method Developer: Administrator
Pump A: L-2130
Pump A Solvent A: Hexane
Pump A Solvent B: 10/90 iPrOH/Hexane
Pump A Solvent C: iPrOH
Pump A Solvent D: iPrOH
Method Description:

Chrom Type: HPLC Channel : 1
Peak Quantitation: AREA
Calculation Method: AREA%

No.	RT	Area	Area %
1	16.37	59785	2.291
2	17.28	2549818	97.709
		2609603	100.000

Peak rejection level: 0

Supplementary Figure 94. HPLC trace of the alcohol derived from (*S*)-**3n**.

D-2000: Isocratic HPLC Series: 1567 Report Name: modified System: Sys 1

D-2000 Elite HPLC System Manager Report

Analyzed Date and Time: 2017/08/31 10:13
Reported Date and Time: 2018/01/23 22:29
Processed Date and Time: 2018/01/23 22:28
Data Path: C:\WIN32APP\D2000HSM\Isocratic\DATA\1567\
Processing Method: 0.5/99.5 iPrOH/Hexane

System (acquisition): Sys 1
Application(data): Isocratic HPLC
Sample Name: IWA-1635-rac
Injection from this vial: 1 of 1
Sample Description:

Series: 1567
Vial Number: 171
Vial Type: UNK
Volume: 10.0 ul

Chrom Type: HPLC Channel : 1

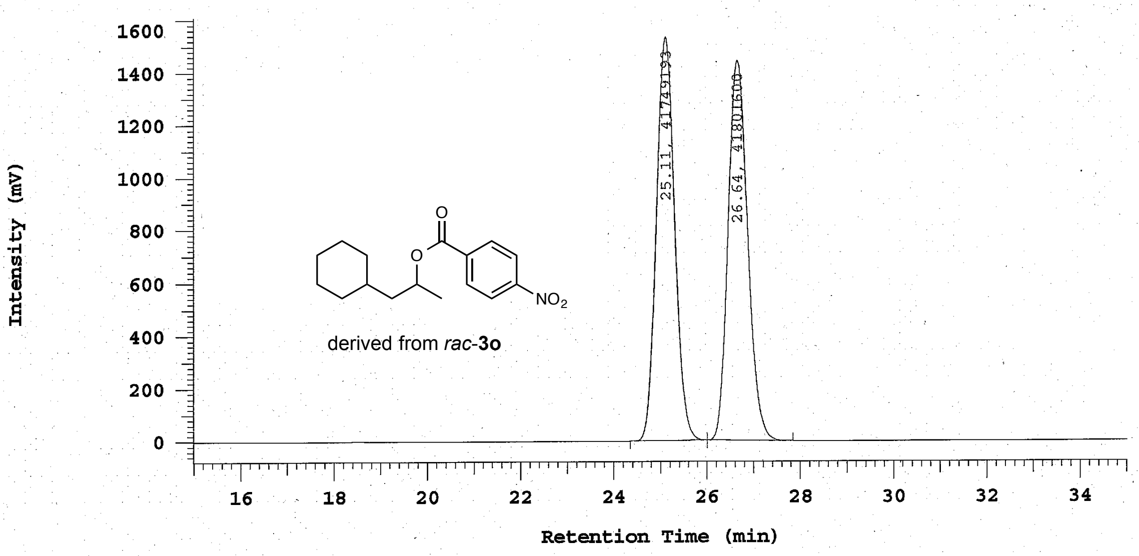

Processing Method: 0.5/99.5 iPrOH/Hexane
Column Type: ~~OD-H-2~~ OZ-3
Method Developer: Administrator
Pump A: L-2130
Pump A Solvent A: Hexane
Pump A Solvent B: 10/90 iPrOH/Hexane
Pump A Solvent C: iPrOH
Pump A Solvent D: EtOH
Method Description:

Chrom Type: HPLC Channel : 1
Peak Quantitation: AREA
Calculation Method: AREA%

No.	RT	Area	Area %
1	25.11	41749193	49.969
2	26.64	41801600	50.031
		83550793	100.000

Peak rejection level: 0

Supplementary Figure 95. HPLC trace of the ester derived from *rac*-**3o**.

D-2000: Isocratic HPLC Series: 1568 Report Name: modified System: Sys 1

D-2000 Elite HPLC System Manager Report

Analyzed Date and Time: 2017/08/31 11:14 Reported Date and Time: 2018/01/23 22:29

Processed Date and Time: 2018/01/23 22:29

Data Path: C:\WIN32APP\D2000HSM\Isocratic\DATA\1568\

Processing Method: 0.5/99.5 iPrOH/Hexane

System (acquisition): Sys 1 Series: 1568

Application(data): Isocratic HPLC Vial Number: 172

Sample Name: IWA-1643-S-Quinox-tODAd-40-5 Vial Type: UNK

Volume: 10.0 ul

Injection from this vial: 1 of 1

Sample Description:

Chrom Type: HPLC Channel : 1

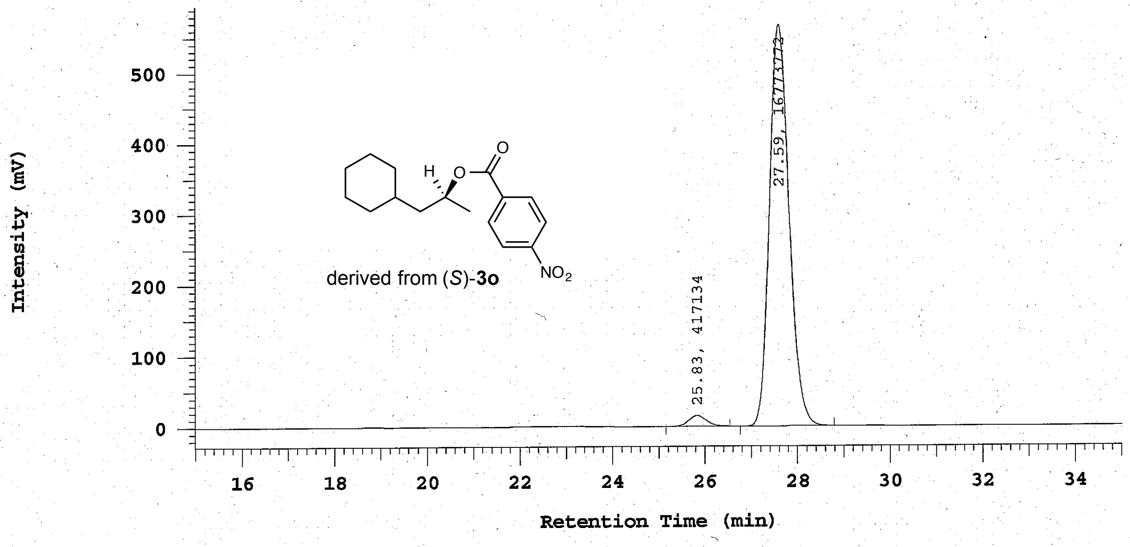

Processing Method: 0.5/99.5 iPrOH/Hexane

Column Type: ~~OD-H-2~~ OZ-3 Method Developer: Administrator

Pump A: L-2130

Pump A Solvent A: Hexane Pump A Solvent B: 10/90 iPrOH/Hexane

Pump A Solvent C: iPrOH Pump A Solvent D: EtOH

Method Description:

Chrom Type: HPLC Channel : 1

Peak Quantitation: AREA

Calculation Method: AREA%

No.	RT	Area	Area %
1	25.83	417134	2.426
2	27.59	16773772	97.574
		17190906	100.000

Peak rejection level: 0

Supplementary Figure 96. HPLC trace of the ester derived from (*S*)-**3o**.

D-2000: Isocratic HPLC Series: 1770 Report Name: modified System: Sys 1

D-2000 Elite HPLC System Manager Report

Analyzed Date and Time: 2017/12/08 10:15
Reported Date and Time: 2018/01/23 22:31
Processed Date and Time: 2018/01/23 22:31
Data Path: C:\WIN32APP\D2000HSM\Isocratic\DATA\1770\
Processing Method: 0.1/99.9 iPrOH/Hexane
System (acquisition): Sys 1
Series: 1770
Application(data): Isocratic HPLC
Vial Number: 161
Sample Name: IWA-1736-rac
Vial Type: UNK
Injection from this vial: 1 of 1
Volume: 10.0 ul
Sample Description:

Chrom Type: HPLC Channel : 1

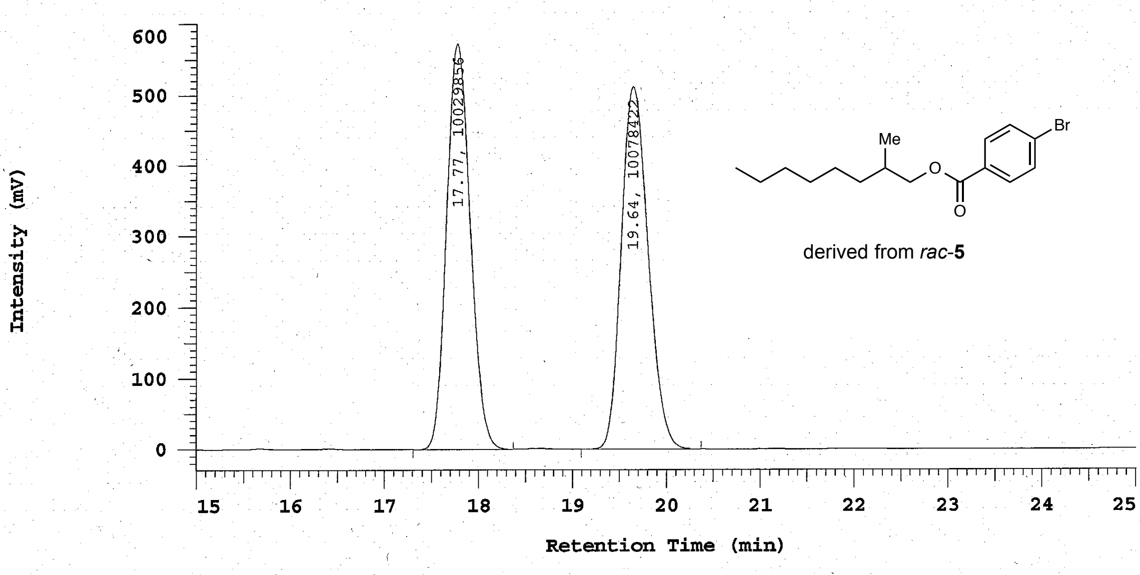

Processing Method: 0.1/99.9 iPrOH/Hexane
Column Type: ~~OD-H-2~~ OD-3
Method Developer: Administrator
Pump A: L-2130
Pump A Solvent A: Hexane
Pump A Solvent B: 10/90 iPrOH/Hexane
Pump A Solvent C: iPrOH
Pump A Solvent D: EtOH
Method Description:

Chrom Type: HPLC Channel : 1
Peak Quantitation: AREA
Calculation Method: AREA%

No.	RT	Area	Area %
1	17.77	10029856	49.879
2	19.64	10078422	50.121
		20108278	100.000

Peak rejection level: 0

Supplementary Figure 97. HPLC trace of the ester derived from *rac*-**5**.

D-2000: Isocratic HPLC Series: 1772 Report Name: modified System: Sys 1

D-2000 Elite HPLC System Manager Report

Analyzed Date and Time: 2017/12/08 12:15 Reported Date and Time: 2018/01/23 22:32

Processed Date and Time: 2018/01/23 22:32

Data Path: C:\WIN32APP\D2000HSM\Isocratic\DATA\1772\

Processing Method: 0.1/99.9 iPrOH/Hexane

System (acquisition): Sys 1 Series: 1772

Application(data): Isocratic HPLC Vial Number: 161

Sample Name: IWA=1738-octene-mixture Vial Type: UNK

Injection from this vial: 1 of 1 Volume: 10.0 ul

Sample Description:

Chrom Type: HPLC Channel : 1

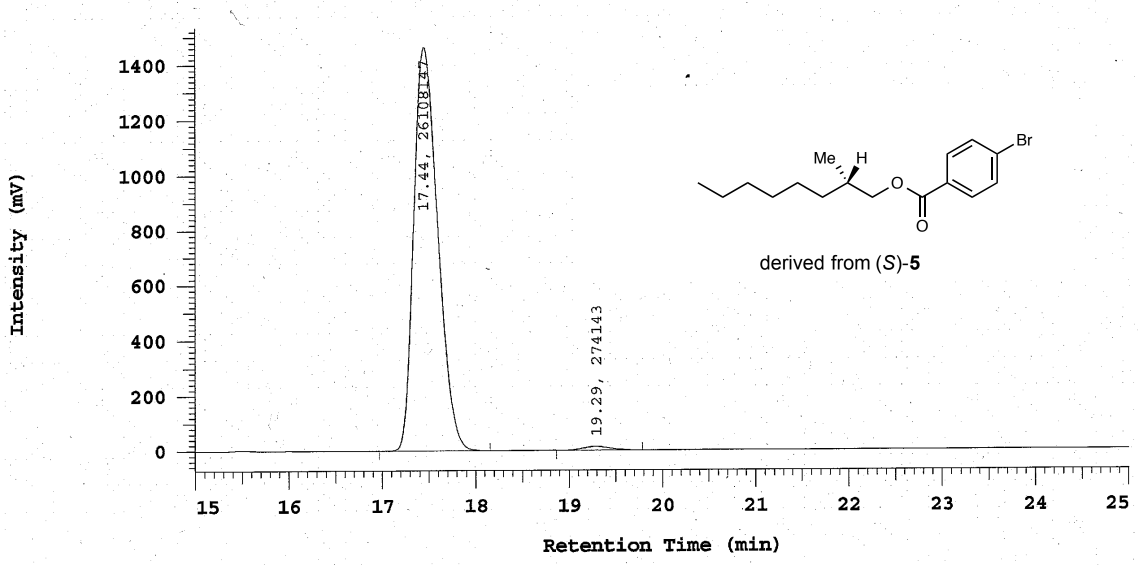

Processing Method: 0.1/99.9 iPrOH/Hexane

Column Type: ~~OD-H-2~~ OD-3 Method Developer: Administrator

Pump A: L-2130

Pump A Solvent A: Hexane Pump A Solvent B: 10/90 iPrOH/Hexane

Pump A Solvent C: iPrOH Pump A Solvent D: EtOH

Method Description:

Chrom Type: HPLC Channel : 1

Peak Quantitation: AREA

Calculation Method: AREA%

No.	RT	Area	Area %
1	17.44	26108147	98.961
2	19.29	274143	1.039
		26382290	100.000

Peak rejection level: 0

Supplementary Figure 98. HPLC trace of the ester derived from (*S*)-**5**.

Supplementary References

1. Hansch, C. & Leo, A. *Exploring QSAR: Fundamentals and Applications in Chemistry and Biology* (American Chemical Society, 1995).
2. Charton, M. Steric Effects. I. Esterification and Acid-Catalyzed Hydrolysis of Esters. *J. Am. Chem. Soc.* **97,** 1552–1556 (1975).
3. Harper, K. C., Bess, E. N. & Sigman, M. S. Multidimensional steric parameters in the analysis of asymmetric catalytic reactions. *Nat. Chem.* **4,** 366–374 (2012).
4. Zhang, Z., Tamura, K., Mayama, D., Sugiya, M. & Imamoto, T. Three-hindered quadrant phosphine ligands with an aromatic ring backbone for the rhodium-catalyzed asymmetric hydrogenation of functionalized alkenes. *J. Org. Chem.* **77,** 4184–4188 (2012).
5. Imamoto, T., Kumada, A. & Yoshida, K. Air-stable P-Chiral Bidentate Phosphine Ligand with (1-Adamantyl)methylphosphino Group. *Chem. Lett.* **36,** 500–501 (2007).
6. Imamoto, T. *et al.* Improved synthetic routes to methylene-bridged P-chiral diphosphine ligands via secondary phosphine-boranes. *Tetrahedron Asymmetry* **21,** 1522–1528 (2010).
7. Imamoto, T., Sugita, K. & Yoshida, K. An air-stable P-chiral phosphine ligand for highly enantioselective transition-metal-catalyzed reactions. *J. Am. Chem. Soc.* **127,** 11934–11935 (2005).
8. Iwamoto, H., Kubota, K. & Ito, H. Highly Selective Markovnikov Hydroboration of Alkyl-Substituted Terminal Alkenes with a Phosphine-Copper(I) Catalyst. *Chem. Commun.* 5916–5919 (2016).
9. Chen, C., Dugan, T. R., Brennessel, W. W., Weix, D. J. & Holland, P. L. Z - selective alkene isomerization by high-spin cobalt(II) complexes. *J. Am. Chem. Soc.* **136,** 945–955 (2014).
10. Yun, H. *et al.* Design and synthesis of a macrosphelide A-biotin chimera. *Org. Biomol. Chem.* **12,** 7127–35 (2014).
11. Smith, J. R. *et al.* Enantioselective Rhodium(III)-Catalyzed Markovnikov Hydroboration of Unactivated Terminal Alkenes. *J. Am. Chem. Soc.* **139,** 9148–9151 (2017).
12. Cai, Y. *et al.*, Copper-catalyzed enantioselective Markovnikov protoboration of α-olefins enabled by a buttressed NHC ligand. *Angew. Chem. Int. Ed.* **57**, 1376–1380 (2018).
13. Moran, W. J. & Morken, J. P. Rh-catalyzed enantioselective hydrogenation of vinyl boronates for the construction of secondary boronic esters. *Org. Lett.* **8,** 2413–2415 (2006).

14. Lee, Y. & Hoveyda, A. H. Efficient boron-copper additions to aryl-substituted alkenes promoted by NHC-based catalysts. Enantioselective Cu-catalyzed hydroboration reactions. *J. Am. Chem. Soc.* **131,** 3160–3161 (2009).
15. Zhang, L., Peng, D., Leng, X. & Huang, Z. Iron-catalyzed, atom-economical, chemo- and regioselective alkene hydroboration with pinacolborane. *Angew. Chem. Int. Ed.* **52,** 3676–3680 (2013).
16. Kubota, K., Yamamoto, E. & Ito, H. Regio- and enantioselective monoborylation of alkenylsilanes catalyzed by an electron-donating chiral phosphine-Copper(I) complex. *Adv. Synth. Catal.* **355,** 3527–3531 (2013).
17. Yang, C. T. *et al.* Alkylboronic esters from copper-catalyzed borylation of primary and secondary alkyl halides and pseudohalides. *Angew. Chem. Int. Ed.* **51,** 528–532 (2012).
18. Kisan, S., Krishnakumar, V. & Gunanathan, C. Ruthenium-Catalyzed Anti-Markovnikov Selective Hydroboration of Olefins. *ACS Catal.* **7,** 5950–5954 (2017).
19. Cacho, R. A. *et al.* Understanding Programming of Fungal Iterative Polyketide Synthases: The Biochemical Basis for Regioselectivity by the Methyltransferase Domain in the Lovastatin Megasynthase. *J. Am. Chem. Soc.* **137,** 15688–15691 (2015).
20. Gaussian 09, Revision C.01, Frisch, M. J.; Trucks, G. W.; Schlegel, H. B.; Scuseria, G. E.; Robb, M. A.; Cheeseman, J. R.; Scalmani, G.; Barone, V.; Mennucci, B.; Petersson, G. A.; Nakatsuji, H.; Caricato, M.; Li, X.; Hratchian, H. P.; Izmaylov, A. F.; Bloino, J.; Zheng, G.; Sonnenberg, J. L.; Hada, M.; Ehara, M.; Toyota, K.; Fukuda, R.; Hasegawa, J.; Ishida, M.; Nakajima, T.; Honda, Y.; Kitao, O.; Nakai, H.; Vreven, T.; Montgomery, J., J. A.; Peralta, J. E.; Ogliaro, F.; Bearpark, M.; Heyd, J. J.; Brothers, E. Kudin, K. N.; Staroverov, V. N.; Kobayashi, R.; Normand, J.; Raghavachari, K.; Rendell, A.; Burant, J. C.; Iyengar, S. S.; Tomasi, J.; Cossi, M.; Rega, N.; Millam, N. J.; Klene, M.; Knox, J. E.; Cross, J. B.; Bakken, V.; Adamo, C.; Jaramillo, J.; Gomperts, R.; Stratmann, R. E.; Yazyev, O.; Austin, A. J.; Cammi, R.; Pomelli, C.; Ochterski, J. W.; Martin, R. L.; Morokuma, K.; Zakrzewski, V. G.; Voth, G. A.; Salvador, P.; Dannenberg, J. J.; Dapprich, S.; Daniels, A. D.; Farkas, Ö.; Foresman, J. B.; Ortiz, J. V.; Cioslowski, J.; Fox, D. J.; Gaussian, Inc., Wallingford CT, 2009.
21. Chai, J.-D. & Head-Gordon, M. Long-range corrected hybrid density functionals with damped atom–atom dispersion corrections. *Phys. Chem. Chem. Phys.* **10,** 6615 (2008).

22. Kerchner, H. A. & Montgomery, J. Synthesis of Secondary and Tertiary Alkylboranes via Formal Hydroboration of Terminal and 1,1-Disubstituted Alkenes. *Org. Lett.* **18,** 5760–5763 (2016).
23. Kubota, K., Yamamoto, E. & Ito, H. Copper(I)-catalyzed borylative exo - cyclization of alkenyl halides containing unactivated double bond. *J. Am. Chem. Soc.* **135,** 2635–2640 (2013).
24. Sakae, R., Hirano, K. & Miura, M. Ligand-controlled regiodivergent Cu-catalyzed aminoboration of unactivated terminal alkenes. *J. Am. Chem. Soc.* **137**, 6460–6463 (2015).
25. Su, W. *et al.* Ligand-controlled regiodivergent copper-catalyzed alkylboration of alkenes. *Angew. Chem. Int. Ed.* **54**, 12957–12961 (2015).
26. Xu, Z. Y., Jiang, Y. Y., Su, W., Yu, H. Z. & Fu, Y. Mechanism of Ligand-Controlled Regioselectivity-Switchable Copper-Catalyzed Alkylboration of Alkenes. *Chem. Eur. J.* **22,** 14611–14617 (2016).
27. Johnson, E. R. *et al.* NCI : revealing non-covalent interactions. *J. Am. Chem. Soc.* **132,** 6498–6506 (2010).
28. Contreras-García, J. *et al.* NCIPLOT: A program for plotting noncovalent interaction regions. *J. Chem. Theory Comput.* **7,** 625–632 (2011).
