# Supplementary material for: Computational design of high-performance ligand for enantioselective Markovnikov hydroboration of aliphatic terminal alkenes
Source: Nat Commun. 2018 Jun 12;9:2290. doi: 10.1038/s41467-018-04693-9 (PMC5997753; doi:10.1038/s41467-018-04693-9)
Supplement: Supplementary file 3 — Description of Additional Supplementary Files [file 41467_2018_4693_MOESM3_ESM.pdf]

Description of Additional Supplementary Files

File Name: Supplementary Data 1
Description: Coordination profiles of DFT calculation.
