# Supplementary material for: Computational design of high-performance ligand for enantioselective Markovnikov hydroboration of aliphatic terminal alkenes
Source: Nat Commun. 2018 Jun 12;9:2290. doi: 10.1038/s41467-018-04693-9 (PMC5997753; doi:10.1038/s41467-018-04693-9)
Supplement: Supplementary file 4 — Supplementary Data 1 [file 41467_2018_4693_MOESM4_ESM.pdf]

Coordination Profiles of DFT calculation

1-Butene

C	-1.714730	-0.252986	-0.293764
H	-1.909268	-1.167408	0.274553
H	-1.502040	-0.541628	-1.326299
H	-2.629229	0.345335	-0.287740
C	-0.539901	0.526686	0.303899
H	-0.365102	1.440978	-0.271948
H	-0.799334	0.839531	1.322427
C	0.719656	-0.287628	0.347869
H	0.672100	-1.202149	0.938694
C	1.847436	0.013247	-0.282397
H	1.935406	0.913139	-0.884936
H	2.722703	-0.623711	-0.218392

Zero-point correction= 0.108794 (Hartree/Particle)
Thermal correction to Energy= 0.114087
Thermal correction to Enthalpy= 0.115031
Thermal correction to Gibbs Free Energy= 0.081179
Sum of electronic and zero-point Energies= -157.099789
Sum of electronic and thermal Energies= -157.094496
Sum of electronic and thermal Enthalpies= -157.093552
Sum of electronic and thermal Free Energies= -157.127404

MeOH

C	0.046376	0.657418	-0.000000
H	-0.437398	1.077953	0.892086
H	1.089299	0.977848	-0.000000
H	-0.437398	1.077953	-0.892086
O	0.046376	-0.753771	0.000000
H	-0.863770	-1.048093	0.000000

Zero-point correction= 0.051805 (Hartree/Particle)
Thermal correction to Energy= 0.055093
Thermal correction to Enthalpy= 0.056038
Thermal correction to Gibbs Free Energy= 0.029098
Sum of electronic and zero-point Energies= -115.665452
Sum of electronic and thermal Energies= -115.662163
Sum of electronic and thermal Enthalpies= -115.661219
Sum of electronic and thermal Free Energies= -115.688159

(*S*)-3

C	2.903832	-1.851202	-0.234479
H	3.101608	-1.891108	-1.310470
H	1.884920	-2.211113	-0.064046
H	3.587252	-2.548659	0.256624
C	3.075245	-0.432028	0.302168

H	2.853147	-0.420703	1.375216
H	4.123562	-0.126543	0.197605
C	2.182595	0.596207	-0.405310
H	2.413352	0.578974	-1.478398
C	2.461170	2.021348	0.108381
H	3.514994	2.284152	-0.028326
H	1.858673	2.764693	-0.420702
H	2.236075	2.103874	1.176910
B	0.652868	0.289998	-0.234336
O	-0.308382	0.658461	-1.139522
O	0.122409	-0.330262	0.868651
C	-1.552981	0.057923	-0.722626
C	-1.311054	-0.176870	0.807826
C	-1.967063	-1.424871	1.374858
H	-1.750450	-1.501448	2.442340
H	-3.052621	-1.378230	1.248294
H	-1.595229	-2.326627	0.888751
C	-1.660638	1.040248	1.662810
H	-2.741017	1.189937	1.727393
H	-1.270103	0.883733	2.670231
H	-1.205117	1.948194	1.260307
C	-2.696234	1.003560	-1.051480
H	-2.786562	1.107952	-2.134604
H	-3.640988	0.609929	-0.665246
H	-2.529809	1.993826	-0.628078
C	-1.699150	-1.242713	-1.511384
H	-2.655277	-1.731292	-1.308759
H	-1.644451	-1.014074	-2.577473
H	-0.892848	-1.940662	-1.273510

Zero-point correction= 0.305752 (Hartree/Particle)
Thermal correction to Energy= 0.320088
Thermal correction to Enthalpy= 0.321032
Thermal correction to Gibbs Free Energy= 0.266398
Sum of electronic and zero-point Energies= -568.813942
Sum of electronic and thermal Energies= -568.799607
Sum of electronic and thermal Enthalpies= -568.798663
Sum of electronic and thermal Free Energies= -568.853297

4

C	5.089772	0.532460	0.576960
H	4.943119	0.356940	1.647212
H	4.933229	1.599809	0.392658
H	6.131752	0.301980	0.340651
C	4.124800	-0.310937	-0.251092
H	4.306782	-0.141633	-1.318890
H	4.315324	-1.375617	-0.072156

C	2.659506	-0.008307	0.056494
H	2.478953	-0.173278	1.126272
C	1.675900	-0.856948	-0.768240
H	1.854561	-1.918840	-0.570701
H	1.870136	-0.686250	-1.833688
H	2.465982	1.055474	-0.126867
B	0.193047	-0.481900	-0.443177
O	-0.420971	0.643321	-0.932983
O	-0.617121	-1.192311	0.403276
C	-1.643528	0.831990	-0.191631
C	-1.938201	-0.614621	0.337807
C	-1.334167	1.828292	0.925171
H	-0.941044	2.743536	0.478236
H	-2.229238	2.078019	1.500063
H	-0.578344	1.434716	1.609063
C	-2.702233	1.401472	-1.121934
H	-3.666698	1.471135	-0.610552
H	-2.409863	2.405437	-1.436679
H	-2.821140	0.788251	-2.014936
C	-2.570830	-0.674223	1.718921
H	-3.536539	-0.160455	1.721800
H	-2.737049	-1.716288	1.999512
H	-1.928111	-0.218519	2.471978
C	-2.743423	-1.461187	-0.646769
H	-2.731995	-2.499101	-0.308319
H	-3.781674	-1.126397	-0.708918
H	-2.304216	-1.425434	-1.646313

Zero-point correction= 0.306738 (Hartree/Particle)
Thermal correction to Energy= 0.321708
Thermal correction to Enthalpy= 0.322652
Thermal correction to Gibbs Free Energy= 0.265396
Sum of electronic and zero-point Energies= -568.812999
Sum of electronic and thermal Energies= -568.798028
Sum of electronic and thermal Enthalpies= -568.797084
Sum of electronic and thermal Free Energies= -568.854340

[(*S*)-Quinox-*t*Bu$_3$]CuB(pin)

Cu	1.046131	0.118989	0.883422
C	5.258601	0.336145	0.092106
C	4.741091	-0.701181	-0.952189
B	2.985926	0.025491	0.382602
O	3.319191	-0.518861	-0.872847
O	4.164559	0.408916	1.020364
P	-0.480617	-1.620046	1.084676
P	-0.657061	1.523225	0.223344
C	-1.174494	-2.455220	2.565681

H	-2.049265	-3.054037	2.308024
H	-1.459734	-1.695704	3.295968
H	-0.405579	-3.086548	3.014359
C	0.024017	-3.024295	-0.063624
C	-0.324786	2.324729	-1.451005
C	-1.301348	2.726823	1.526380
C	-2.132977	0.427120	-0.062943
C	-2.058036	-0.953894	0.342038
C	-2.306079	3.766126	1.025595
H	-1.826999	4.502235	0.376441
H	-2.721304	4.307430	1.883219
H	-3.129399	3.301411	0.479521
C	-0.079788	3.424656	2.146363
H	0.639512	2.700760	2.538273
H	-0.412563	4.063994	2.971365
H	0.444763	4.056907	1.428624
C	-1.957973	1.859889	2.615172
H	-1.287602	1.062546	2.952125
H	-2.892951	1.409933	2.273395
H	-2.188046	2.487999	3.481725
C	0.269362	-2.413290	-1.449412
H	0.610729	-3.197286	-2.133686
H	1.046932	-1.645106	-1.413115
H	-0.644666	-1.983639	-1.871204
C	1.355078	-3.565083	0.481287
H	2.112465	-2.779458	0.520993
H	1.715725	-4.357785	-0.182893
H	1.243346	-3.999475	1.479657
C	-1.002120	-4.156507	-0.150941
H	-1.147966	-4.645846	0.815927
H	-0.636036	-4.914874	-0.851613
H	-1.972626	-3.802732	-0.501119
N	-3.100483	-1.743585	0.259123
N	-3.242556	0.932047	-0.546462
C	-4.318913	0.114476	-0.665683
C	-4.249610	-1.235528	-0.252161
C	-5.392446	-2.062377	-0.367813
H	-5.311893	-3.092270	-0.041267
C	-5.529318	0.622777	-1.194412
C	-6.549831	-1.549218	-0.884439
H	-7.427439	-2.178289	-0.977781
C	-6.618516	-0.197115	-1.300973
H	-5.556362	1.661036	-1.502927
H	-7.546967	0.186655	-1.707595
C	6.518932	-0.077309	0.837326
H	6.794811	0.702823	1.550311

H	7.352436	-0.216513	0.141291
H	6.366722	-1.003081	1.392244
C	5.441367	1.734595	-0.502550
H	6.299698	1.780185	-1.178809
H	5.601165	2.443789	0.312389
H	4.546271	2.042757	-1.047975
C	5.196574	-0.456777	-2.383230
H	4.785198	-1.230731	-3.035752
H	6.288115	-0.493638	-2.457399
H	4.852670	0.510602	-2.749987
C	5.033400	-2.149703	-0.554642
H	6.093426	-2.395350	-0.663468
H	4.453650	-2.812177	-1.201745
H	4.732147	-2.337057	0.478580
C	0.221753	1.180483	-2.323573
H	1.121317	0.731914	-1.893337
H	0.478312	1.579413	-3.310683
H	-0.526014	0.395296	-2.471182
C	-1.509048	2.974292	-2.174168
H	-2.307624	2.259096	-2.368206
H	-1.152604	3.361722	-3.135453
H	-1.934887	3.809752	-1.619631
C	0.790297	3.359958	-1.243544
H	0.421151	4.254426	-0.735107
H	1.172212	3.672954	-2.220894
H	1.623618	2.943030	-0.670782

Zero-point correction= 0.698017 (Hartree/Particle)
Thermal correction to Energy= 0.738922
Thermal correction to Enthalpy= 0.739866
Thermal correction to Gibbs Free Energy= 0.627069
Sum of electronic and zero-point Energies= -2220.842001
Sum of electronic and thermal Energies= -2220.801095
Sum of electronic and thermal Enthalpies= -2220.800151
Sum of electronic and thermal Free Energies= -2220.912948

[(*S*)-Quinox-*t*Bu$_3$]CuOMe

Cu	-2.181053	0.321448	0.292456
P	-0.560034	1.787790	0.762926
P	-0.705838	-1.431068	0.108609
C	-0.111810	2.495755	2.394096
H	0.888995	2.929842	2.371812
H	-0.149675	1.702361	3.142584
H	-0.843346	3.257884	2.668551
C	-0.531118	3.240493	-0.429829
C	-0.881703	-2.351004	-1.521740
C	-0.444360	-2.560197	1.596720

C	0.926990	-0.552059	0.004780
C	0.998553	0.838914	0.373214
C	0.846379	-3.385577	1.616844
H	0.914468	-4.074635	0.775873
H	0.866872	-3.978728	2.538315
H	1.736132	-2.754837	1.604735
C	-1.661345	-3.491265	1.689165
H	-2.602650	-2.941179	1.603280
H	-1.651695	-4.001224	2.658199
H	-1.638721	-4.258896	0.912581
C	-0.447176	-1.616398	2.812704
H	-1.382397	-1.054484	2.883670
H	0.383159	-0.903419	2.775389
H	-0.332380	-2.204966	3.728939
C	-0.456615	2.656909	-1.846911
H	-0.552109	3.463801	-2.580378
H	-1.268203	1.944879	-2.026075
H	0.496516	2.152147	-2.029134
C	-1.880944	3.952478	-0.248036
H	-2.720716	3.279296	-0.436957
H	-1.944218	4.788033	-0.953076
H	-1.991647	4.365625	0.759439
C	0.612945	4.230099	-0.195931
H	0.535114	4.709113	0.783808
H	0.561925	5.020593	-0.952424
H	1.587742	3.746307	-0.263469
N	2.145779	1.469675	0.430943
N	2.000531	-1.209416	-0.364041
C	3.186671	-0.551932	-0.357753
C	3.265667	0.792928	0.073209
C	4.522914	1.441414	0.114094
H	4.557482	2.469877	0.453077
C	4.363156	-1.227794	-0.760586
C	5.645874	0.765633	-0.275688
H	6.610954	1.257964	-0.248758
C	5.564949	-0.577456	-0.719123
H	4.273540	-2.256344	-1.088916
H	6.469547	-1.090088	-1.024882
C	-0.588115	-1.313344	-2.618438
H	-1.215573	-0.425405	-2.499841
H	-0.823293	-1.755407	-3.591815
H	0.461982	-1.012342	-2.632690
C	0.021923	-3.571895	-1.701643
H	1.073841	-3.319988	-1.555666
H	-0.105369	-3.964335	-2.716821
H	-0.248728	-4.373528	-1.010262

C	-2.365961	-2.746940	-1.634337
H	-2.643376	-3.509962	-0.904292
H	-2.539727	-3.169667	-2.630220
H	-3.026120	-1.883172	-1.496430
O	-3.852052	0.129493	-0.491614
C	-4.712316	1.173384	-0.778960
H	-5.669749	0.795572	-1.177494
H	-4.966914	1.795044	0.101004
H	-4.318139	1.870819	-1.546393

Zero-point correction= 0.556521 (Hartree/Particle)
Thermal correction to Energy= 0.590557
Thermal correction to Enthalpy= 0.591501
Thermal correction to Gibbs Free Energy= 0.492179
Sum of electronic and zero-point Energies= -1924.863801
Sum of electronic and thermal Energies= -1924.829765
Sum of electronic and thermal Enthalpies= -1924.828821
Sum of electronic and thermal Free Energies= -1924.928143

[(*S*)-Quinox-*t*Bu$_3$]: II-A$_{branch}$

C	-4.150109	-1.376728	-0.599446
C	-4.240554	-0.018990	-0.982729
C	-2.177441	0.386547	-0.080445
C	-2.054822	-1.009074	0.238839
P	-0.852005	1.565939	0.474461
P	-0.464008	-1.651184	0.951600
Cu	0.965634	0.204259	1.007718
B	2.420519	0.191583	-0.422407
O	2.347121	-0.366671	-1.706496
O	3.703568	0.731222	-0.265945
C	1.347902	0.396121	3.205113
H	1.058362	1.413566	3.436040
H	0.803380	-0.395718	3.711313
C	2.511034	0.129568	2.544867
H	3.143658	0.952615	2.225903
C	3.170733	-1.223972	2.557311
H	3.639198	-1.408972	1.588756
H	2.412075	-2.000449	2.701912
C	-1.110370	-2.476595	2.463597
H	-1.984798	-3.093786	2.251295
H	-1.377695	-1.704365	3.188019
H	-0.322498	-3.094829	2.900583
C	-0.022728	-3.073849	-0.207253
C	1.437340	-3.430964	0.107918
H	1.754536	-4.258542	-0.535988
H	1.565047	-3.753560	1.146759
H	2.094193	-2.578192	-0.074883

C	-0.112557	-2.556035	-1.649246
H	0.474392	-1.645104	-1.786252
H	-1.148882	-2.373096	-1.944775
H	0.295239	-3.314687	-2.326497
C	-0.905437	-4.313585	-0.035031
H	-0.610866	-5.065015	-0.776042
H	-1.960507	-4.078867	-0.182306
H	-0.783961	-4.765988	0.953228
C	4.227755	-1.317721	3.661471
H	4.996843	-0.551040	3.529358
H	4.720593	-2.293602	3.647566
H	3.777959	-1.173844	4.647630
C	3.506316	-0.000116	-2.471661
C	4.545430	0.309924	-1.350415
C	3.134742	1.229628	-3.301507
H	2.267601	0.986380	-3.919775
H	3.953691	1.537704	-3.956927
H	2.863169	2.068820	-2.657146
C	3.876242	-1.154172	-3.391191
H	4.816680	-0.950075	-3.912597
H	3.093469	-1.291069	-4.141055
H	3.978334	-2.087271	-2.836677
C	5.531548	1.420771	-1.677902
H	6.122060	1.167711	-2.564238
H	6.218239	1.561113	-0.839930
H	5.018387	2.366013	-1.856171
C	5.299572	-0.935104	-0.878761
H	5.838035	-0.691708	0.040201
H	6.020731	-1.281647	-1.623811
H	4.605781	-1.750463	-0.658332
C	-1.794764	2.495384	1.841423
C	-0.544657	2.657172	-1.042765
C	-0.384891	1.697867	-2.234620
H	-1.343021	1.263385	-2.527598
H	0.323307	0.894207	-2.022591
H	0.001795	2.262752	-3.089785
C	-0.854049	3.587515	2.370088
H	-1.261320	4.007976	3.295777
H	-0.755940	4.406080	1.652509
H	0.146459	3.202520	2.581260
C	-1.628302	3.687381	-1.371684
H	-2.612758	3.225352	-1.452439
H	-1.386197	4.153864	-2.333355
H	-1.665082	4.485963	-0.627561
C	0.790129	3.383340	-0.808686
H	0.729824	4.100470	0.014063

H	1.054010	3.941588	-1.713921
H	1.595932	2.680485	-0.586228
C	-3.147994	3.124032	1.484917
H	-3.061197	3.938612	0.769161
H	-3.587802	3.535033	2.401091
H	-3.845199	2.392232	1.076547
C	-2.049313	1.448950	2.940504
H	-1.135739	0.935594	3.238792
H	-2.772534	0.696727	2.609821
H	-2.471824	1.944550	3.820798
C	-5.225187	-2.254564	-0.876667
C	-6.335440	-1.783849	-1.521017
C	-5.399460	0.443419	-1.651088
C	-6.422392	-0.425426	-1.912677
N	-3.034713	-1.849009	0.010565
N	-3.241093	0.850621	-0.691036
H	-5.129885	-3.289983	-0.572349
H	-7.159060	-2.453372	-1.740290
H	-7.312777	-0.076136	-2.422410
H	-5.443747	1.488368	-1.933893

Zero-point correction= 0.809613 (Hartree/Particle)
Thermal correction to Energy= 0.856318
Thermal correction to Enthalpy= 0.857262
Thermal correction to Gibbs Free Energy= 0.733379
Sum of electronic and zero-point Energies= -2377.961616
Sum of electronic and thermal Energies= -2377.914911
Sum of electronic and thermal Enthalpies= -2377.913967
Sum of electronic and thermal Free Energies= -2378.037850

[(*S*)-Quinox-*t*Bu$_3$]: II-B$_{branch}$

C	-4.089826	-0.919802	-0.751935
C	-3.881226	0.071520	-1.736384
C	-1.875507	0.620770	-0.775791
C	-2.069792	-0.416290	0.203390
P	-0.346893	1.686579	-0.727488
P	-0.726837	-0.807127	1.429327
Cu	1.058482	0.545554	0.770130
B	2.398894	-0.456127	-0.406589
O	2.230374	-0.856220	-1.741075
O	3.681542	-0.844701	-0.002747
C	3.327330	-1.693526	-2.146430
C	4.444568	-1.273431	-1.141482
C	1.554609	1.873821	2.467507
H	1.084022	2.838784	2.306694
H	1.237227	1.323717	3.344278
C	2.689301	1.521393	1.791203

C	-1.600526	-0.570136	3.099955
C	-0.402919	-2.647790	1.084591
C	0.182484	1.555664	-2.477805
C	-1.051673	3.439486	-0.617870
C	0.975466	-2.979506	1.678957
H	1.220451	-4.022539	1.448442
H	0.991418	-2.871359	2.766495
H	1.756600	-2.338296	1.265721
C	-0.337523	-2.813136	-0.443341
H	0.302101	-2.064679	-0.915873
H	-1.331636	-2.767954	-0.893376
H	0.083834	-3.798335	-0.669446
C	-1.439503	-3.630136	1.637674
H	-1.214792	-4.629007	1.246824
H	-2.453005	-3.364783	1.333732
H	-1.397160	-3.694972	2.727107
C	-1.817020	3.891824	-1.865233
H	-2.636520	3.213353	-2.103082
H	-1.161338	3.951999	-2.737497
H	-2.228808	4.892853	-1.692698
C	0.154841	4.365468	-0.403305
H	-0.185810	5.405427	-0.353693
H	0.877921	4.295125	-1.221741
H	0.676207	4.136997	0.528713
C	-1.961956	3.513613	0.611133
H	-1.443698	3.174719	1.510847
H	-2.862779	2.906656	0.482962
H	-2.279484	4.549033	0.774212
C	3.641623	-1.422780	-3.610183
H	2.804179	-1.749348	-4.231285
H	4.533912	-1.973379	-3.924139
H	3.803609	-0.360273	-3.792621
C	2.885071	-3.146808	-1.970564
H	3.650102	-3.845601	-2.319159
H	1.973701	-3.311547	-2.549616
H	2.661527	-3.363260	-0.923598
C	5.255810	-0.068226	-1.621002
H	5.926294	-0.330822	-2.443774
H	5.857414	0.302566	-0.787884
H	4.596783	0.739027	-1.949660
C	5.383595	-2.393675	-0.720434
H	6.129231	-2.004200	-0.023488
H	5.908351	-2.806849	-1.587491
H	4.843700	-3.198402	-0.221128
C	3.436340	2.455512	0.874906
H	2.741299	3.174422	0.431244

H	3.875253	1.882807	0.056576
C	4.544731	3.198071	1.627802
H	5.102850	3.854446	0.954509
H	5.253423	2.491642	2.070200
H	4.131215	3.807441	2.436012
H	3.253678	0.654954	2.125049
H	0.930367	2.327354	-2.676285
H	-0.657426	1.665692	-3.165056
H	0.666670	0.586101	-2.604090
C	-2.934643	-1.291340	3.335235
H	-3.654197	-1.089535	2.542452
H	-3.355845	-0.929576	4.280441
H	-2.821061	-2.369711	3.420700
C	-0.601988	-0.985870	4.190101
H	-0.974516	-0.661791	5.167765
H	0.383938	-0.539434	4.037669
H	-0.479515	-2.070961	4.228459
C	-1.872147	0.938759	3.202058
H	-0.970425	1.528668	3.040472
H	-2.263895	1.169055	4.198344
H	-2.622062	1.253958	2.471274
C	-4.858397	0.275764	-2.739710
C	-5.997566	-0.480986	-2.739487
C	-5.276582	-1.690534	-0.772703
C	-6.209354	-1.470088	-1.747862
N	-2.756037	0.828713	-1.724327
N	-3.154699	-1.153140	0.201471
H	-4.672476	1.038033	-3.486878
H	-6.750217	-0.327226	-3.504138
H	-7.121021	-2.055942	-1.769243
H	-5.409967	-2.444515	-0.006039

Zero-point correction= 0.810035 (Hartree/Particle)
Thermal correction to Energy= 0.856565
Thermal correction to Enthalpy= 0.857509
Thermal correction to Gibbs Free Energy= 0.734503
Sum of electronic and zero-point Energies= -2377.958264
Sum of electronic and thermal Energies= -2377.911735
Sum of electronic and thermal Enthalpies= -2377.910791
Sum of electronic and thermal Free Energies= -2378.033796

[(*S*)-Quinox-*t*Bu$_3$]: II-C$_{branch}$

C	4.195239	-1.345123	0.057466
C	4.164142	-0.327758	1.038730
C	2.120633	0.363744	0.278732
C	2.120590	-0.716828	-0.669676
P	0.729635	1.592594	0.252104

P	0.620245	-1.004795	-1.728906
Cu	-0.979344	0.456929	-0.921453
B	-2.363586	-0.498962	0.240098
O	-2.209648	-1.046430	1.519638
O	-3.687709	-0.727338	-0.161777
C	-3.375483	-1.804725	1.881697
C	-4.465292	-1.180300	0.956354
C	-1.486232	1.683454	-2.728582
H	-0.982613	2.642034	-2.671837
H	-1.202629	1.032256	-3.547422
C	-2.602611	1.444609	-1.982979
C	1.384316	-0.922303	-3.400515
H	2.308481	-1.499943	-3.455792
H	1.593178	0.122818	-3.635153
H	0.671760	-1.300364	-4.137448
C	0.253893	-2.838672	-1.485250
C	0.344937	1.919193	2.075927
C	1.631636	3.066175	-0.550145
C	-1.144454	-3.064585	-2.080617
H	-1.429037	-4.114000	-1.946343
H	-1.166894	-2.854000	-3.155332
H	-1.894460	-2.434737	-1.596201
C	0.222024	-3.120519	0.022784
H	-0.410309	-2.408497	0.558326
H	1.225833	-3.093479	0.454383
H	-0.185640	-4.123113	0.192354
C	1.257513	-3.772191	-2.169788
H	0.993215	-4.810670	-1.941011
H	2.274920	-3.589540	-1.821872
H	1.236673	-3.662660	-3.257540
C	0.303484	0.553329	2.780838
H	1.306753	0.145190	2.915911
H	-0.319939	-0.166783	2.248206
H	-0.138828	0.691041	3.773901
C	1.315120	2.835721	2.825479
H	2.343291	2.480847	2.743592
H	1.037977	2.844183	3.885702
H	1.260430	3.867404	2.470260
C	-1.067829	2.527467	2.106564
H	-1.361219	2.697161	3.148346
H	-1.791469	1.850799	1.646827
H	-1.117006	3.488450	1.586687
C	2.936645	3.549984	0.095707
H	3.652975	2.739842	0.235948
H	2.777585	4.028545	1.059420
H	3.394043	4.292595	-0.568371

C	0.631503	4.229182	-0.604468
H	1.040398	5.038571	-1.218782
H	0.438209	4.637363	0.390746
H	-0.323920	3.924835	-1.037733
C	1.970098	2.612650	-1.981245
H	1.101621	2.205406	-2.499149
H	2.756648	1.851642	-1.980839
H	2.343966	3.468941	-2.552295
H	-3.218810	0.578716	-2.204607
C	-3.252100	2.487023	-1.109986
H	-3.652678	2.007633	-0.214673
H	-2.503625	3.213986	-0.778036
C	-3.085040	-3.271135	1.559389
H	-3.907444	-3.922853	1.866430
H	-2.182370	-3.576788	2.093168
H	-2.906280	-3.410148	0.490763
C	-3.623315	-1.642251	3.373941
H	-2.804717	-2.102935	3.931814
H	-4.556606	-2.131819	3.669013
H	-3.673524	-0.590428	3.655981
C	-5.131958	0.053602	1.568317
H	-5.798014	-0.210041	2.394279
H	-5.719810	0.553365	0.794703
H	-4.382847	0.761004	1.933151
C	-5.526364	-2.153391	0.465490
H	-6.238668	-1.626014	-0.173102
H	-6.076154	-2.586022	1.307192
H	-5.084302	-2.961734	-0.117270
C	-4.383843	3.204613	-1.851645
H	-4.870225	3.939844	-1.204821
H	-5.144431	2.490282	-2.180052
H	-4.006361	3.722437	-2.737612
C	5.247611	-0.189383	1.938491
C	6.317856	-1.035108	1.845013
C	5.319069	-2.201679	-0.024541
C	6.355274	-2.046390	0.854248
H	5.195684	0.595136	2.683854
H	7.149092	-0.935297	2.533376
H	7.215075	-2.704052	0.799339
H	5.320401	-2.971071	-0.787257
N	3.118693	0.533442	1.111798
N	3.149853	-1.518974	-0.789901

Zero-point correction= 0.809763 (Hartree/Particle)
Thermal correction to Energy= 0.856411
Thermal correction to Enthalpy= 0.857355
Thermal correction to Gibbs Free Energy= 0.733864

Sum of electronic and zero-point Energies= -2377.961072
Sum of electronic and thermal Energies= -2377.914424
Sum of electronic and thermal Enthalpies= -2377.913480
Sum of electronic and thermal Free Energies= -2378.036971

[(*S*)-Quinox-*t*Bu$_3$]: II-D$_{branch}$

C	3.913858	-0.462003	-1.376362
C	3.972747	-1.456312	-0.374740
C	1.991947	-0.709194	0.500268
C	1.930564	0.302626	-0.522394
P	0.601569	-0.894225	1.729489
P	0.472927	1.453816	-0.618554
Cu	-1.075517	0.509338	0.909543
B	-2.359355	-0.758980	-0.060183
O	-2.021446	-2.029124	-0.555179
O	-3.701382	-0.525981	-0.381569
C	-1.497642	2.059883	2.420336
H	-1.123113	1.646000	3.348463
H	-1.058728	2.997050	2.097174
C	-2.662662	1.619590	1.855009
H	-3.218587	0.824528	2.344075
C	-3.432178	2.415283	0.832331
H	-3.900152	1.728798	0.125639
H	-2.741157	3.045494	0.262725
C	1.302415	3.144781	-0.338451
C	-0.097343	1.278701	-2.422817
C	0.271590	-2.685569	1.519807
C	1.437235	-0.814090	3.422382
C	-1.543380	1.800087	-2.468564
H	-1.946840	1.652691	-3.476611
H	-1.602010	2.868341	-2.244171
H	-2.183353	1.270137	-1.760676
C	-0.096503	-0.221321	-2.763194
H	-0.587205	-0.827837	-1.999634
H	0.919069	-0.595054	-2.910214
H	-0.638144	-0.364425	-3.705185
C	0.737957	2.012299	-3.476056
H	0.376732	1.723630	-4.469645
H	1.794515	1.751015	-3.405055
H	0.629894	3.096122	-3.397829
C	2.467542	-1.918185	3.678914
H	3.268956	-1.901558	2.940507
H	2.008175	-2.909263	3.656453
H	2.903734	-1.778608	4.674737
C	0.300665	-0.939495	4.449939
H	0.709573	-0.827955	5.459874

H	-0.186667	-1.916919	4.398589
H	-0.465418	-0.173608	4.307387
C	2.100228	0.557717	3.567693
H	1.383029	1.366281	3.411603
H	2.920521	0.682590	2.855091
H	2.516894	0.663325	4.575040
C	-4.499138	3.288786	1.499225
H	-5.201397	2.675755	2.072029
H	-5.072753	3.843110	0.751352
H	-4.046823	4.008697	2.187332
C	2.505246	3.521688	-1.214490
H	3.275717	2.751261	-1.203882
H	2.941159	4.445135	-0.815926
H	2.233992	3.710519	-2.250561
C	0.212694	4.215995	-0.486005
H	0.600441	5.179727	-0.138674
H	-0.678198	3.979418	0.101033
H	-0.090354	4.340630	-1.528240
C	1.793717	3.116422	1.116424
H	1.001757	2.843831	1.812084
H	2.171168	4.106846	1.391744
H	2.614168	2.404652	1.241730
H	-0.271562	-2.808659	0.582218
H	1.196580	-3.263552	1.517730
H	-0.382602	-3.023355	2.326753
C	-3.200697	-2.718716	-0.998454
C	-4.179335	-1.537555	-1.283463
C	-3.658338	-3.613413	0.154497
H	-2.839013	-4.282788	0.426704
H	-4.524385	-4.221118	-0.121915
H	-3.913108	-3.015918	1.032681
C	-2.847390	-3.569230	-2.209623
H	-3.741413	-4.040052	-2.630023
H	-2.153944	-4.359184	-1.911809
H	-2.366475	-2.973429	-2.985999
C	-4.044843	-0.980363	-2.701487
H	-4.587485	-0.034130	-2.760275
H	-4.457174	-1.666443	-3.446295
H	-2.999211	-0.783645	-2.945504
C	-5.641304	-1.825800	-0.977490
H	-6.007876	-2.659999	-1.583781
H	-6.243857	-0.944673	-1.210221
H	-5.787478	-2.065666	0.075792
C	4.946326	-0.375527	-2.340424
C	5.992268	-1.254672	-2.291914
C	5.065058	-2.356176	-0.347143

C	6.051875	-2.252182	-1.288103
H	4.874661	0.394603	-3.099121
H	6.785185	-1.195094	-3.028378
H	6.889001	-2.940495	-1.272738
H	5.084237	-3.112881	0.427934
N	2.872221	0.403914	-1.429988
N	2.986378	-1.562076	0.549716

Zero-point correction= 0.810900 (Hartree/Particle)
Thermal correction to Energy= 0.857208
Thermal correction to Enthalpy= 0.858152
Thermal correction to Gibbs Free Energy= 0.736044
Sum of electronic and zero-point Energies= -2377.957747
Sum of electronic and thermal Energies= -2377.911439
Sum of electronic and thermal Enthalpies= -2377.910495
Sum of electronic and thermal Free Energies= -2378.032603

[(*S*)-Quinox-*t*Bu$_3$]: II-A$_{linear}$

C	-3.943525	-1.580979	0.241602
C	-3.949760	-0.931941	-1.013980
C	-1.994476	0.134580	-0.489258
C	-1.952341	-0.580712	0.757698
P	-0.694115	1.409571	-0.851470
P	-0.488964	-0.385493	1.887954
Cu	1.057782	0.782060	0.598780
B	2.430674	-0.410338	-0.326546
O	2.953654	-1.629062	0.118262
O	3.101390	-0.060284	-1.504338
C	1.648901	2.573562	1.796603
H	1.139738	3.403899	1.314974
C	2.712802	2.001414	1.151973
H	3.053612	2.358944	0.187526
C	-1.355184	0.222929	3.396457
H	-2.240208	-0.377006	3.612606
H	-1.648827	1.262184	3.246073
H	-0.668848	0.183540	4.244978
C	-0.028128	-2.155881	2.352833
C	1.355782	-2.046544	3.012537
H	1.671216	-3.039021	3.353336
H	1.334347	-1.391152	3.890842
H	2.099549	-1.675704	2.305745
C	0.077089	-2.987652	1.068887
H	0.723844	-2.503841	0.335837
H	-0.906526	-3.168350	0.628029
H	0.520703	-3.960138	1.308836
C	-1.010188	-2.821457	3.322924
H	-0.684348	-3.852199	3.501347

H	-2.021857	-2.844991	2.915858
H	-1.033567	-2.315978	4.291916
C	3.789530	-2.202011	-0.901174
C	4.214142	-0.942267	-1.717535
C	2.920254	-3.170891	-1.705254
H	2.494357	-3.908806	-1.021590
H	3.499264	-3.697770	-2.468442
H	2.094578	-2.646294	-2.192202
C	4.931909	-2.957601	-0.239912
H	5.637950	-3.327224	-0.990108
H	4.534517	-3.815547	0.307167
H	5.470295	-2.325346	0.466077
C	4.385431	-1.170742	-3.211490
H	5.164280	-1.914800	-3.405595
H	4.678910	-0.235422	-3.693666
H	3.455372	-1.508633	-3.669292
C	5.452039	-0.249815	-1.145805
H	5.562758	0.723939	-1.628061
H	6.362362	-0.829284	-1.321912
H	5.342181	-0.085167	-0.071417
C	-1.700250	2.995884	-0.532682
C	-0.320284	1.197880	-2.695082
C	-0.082033	-0.303499	-2.927639
H	-1.018474	-0.864388	-2.914069
H	0.598365	-0.717085	-2.181145
H	0.381442	-0.437481	-3.910780
C	-0.823269	4.189380	-0.937196
H	-1.273036	5.116004	-0.564709
H	-0.738803	4.277413	-2.022584
H	0.185915	4.113562	-0.523683
C	-1.390150	1.689037	-3.674754
H	-2.363195	1.242447	-3.468609
H	-1.088335	1.405214	-4.689446
H	-1.483274	2.777360	-3.662407
C	0.996317	1.949170	-2.959363
H	0.899627	3.022694	-2.780580
H	1.268493	1.815129	-4.012499
H	1.810478	1.553828	-2.351043
C	-3.069257	3.114600	-1.213971
H	-2.999699	3.157298	-2.298453
H	-3.544590	4.042379	-0.874833
H	-3.730285	2.287616	-0.952285
C	-1.936219	3.037517	0.986192
H	-1.005110	2.945608	1.546455
H	-2.610935	2.237148	1.305122
H	-2.409928	3.988362	1.252148

C	1.411211	2.419827	3.276926
H	1.775038	1.438527	3.600846
H	0.339120	2.443624	3.492440
C	2.106471	3.522193	4.082382
H	1.750472	4.511072	3.778170
H	3.187243	3.493403	3.920541
H	1.916238	3.410011	5.153662
H	3.405422	1.365686	1.695112
C	-5.003620	-2.455224	0.581168
C	-6.015188	-2.672763	-0.313043
C	-5.005850	-1.176657	-1.923650
C	-6.014923	-2.031211	-1.575659
N	-2.968522	-0.056401	-1.345875
N	-2.923172	-1.385712	1.113987
H	-4.976782	-2.938516	1.550450
H	-6.826369	-3.345256	-0.059058
H	-6.825754	-2.222905	-2.268872
H	-4.983680	-0.669013	-2.880511

Zero-point correction= 0.810635 (Hartree/Particle)
Thermal correction to Energy= 0.857010
Thermal correction to Enthalpy= 0.857954
Thermal correction to Gibbs Free Energy= 0.735383
Sum of electronic and zero-point Energies= -2377.957129
Sum of electronic and thermal Energies= -2377.910754
Sum of electronic and thermal Enthalpies= -2377.909810
Sum of electronic and thermal Free Energies= -2378.032381

[(*S*)-Quinox-*t*Bu$_3$]: II-B$_{linear}$

C	-4.018040	-1.279379	-0.126998
C	-3.804904	-0.984520	-1.492111
C	-1.818542	0.028895	-0.975980
C	-2.006045	-0.323285	0.406565
P	-0.305610	0.966685	-1.531457
P	-0.644363	0.004159	1.625556
Cu	1.146103	0.828954	0.248199
B	2.488980	-0.631805	-0.275317
O	2.248810	-1.742256	-1.101051
O	3.831721	-0.672359	0.113952
C	3.380213	-2.628135	-1.074568
C	4.533011	-1.666618	-0.648604
C	1.763892	2.851706	1.147588
H	0.960163	3.093254	1.836907
C	2.609698	1.826337	1.449319
C	-1.509092	1.075610	2.933874
C	-0.276959	-1.738103	2.286931
C	0.187989	-0.076297	-2.954983

C	-1.003797	2.521350	-2.347606
C	1.123888	-1.681470	2.918037
H	1.402122	-2.683518	3.263229
H	1.160037	-1.014038	3.783383
H	1.869837	-1.342622	2.195421
C	-0.228222	-2.676351	1.069201
H	0.390270	-2.277359	0.262335
H	-1.228983	-2.886074	0.685075
H	0.213039	-3.629176	1.381133
C	-1.273125	-2.301152	3.303125
H	-1.035352	-3.356247	3.480194
H	-2.299839	-2.238143	2.939312
H	-1.202070	-1.788930	4.264883
C	-2.066149	2.287404	-3.425796
H	-2.978630	1.855491	-3.015606
H	-1.707411	1.622447	-4.214561
H	-2.316875	3.248920	-3.888798
C	0.204749	3.223050	-2.989029
H	-0.081343	4.233312	-3.300495
H	0.548814	2.689843	-3.878891
H	1.044207	3.307022	-2.296610
C	-1.586710	3.400399	-1.237659
H	-0.851498	3.595123	-0.453885
H	-2.458504	2.929743	-0.774080
H	-1.909414	4.360667	-1.653883
C	3.546509	-3.262433	-2.447554
H	2.696465	-3.916695	-2.655382
H	4.459150	-3.865205	-2.489367
H	3.590288	-2.505898	-3.231194
C	3.089919	-3.710811	-0.033690
H	3.888736	-4.455932	0.007265
H	2.158710	-4.215431	-0.300573
H	2.962357	-3.273898	0.959460
C	5.181633	-0.949943	-1.834648
H	5.791715	-1.628644	-2.437056
H	5.823810	-0.153139	-1.453237
H	4.422684	-0.495331	-2.475513
C	5.606536	-2.298088	0.224777
H	6.361840	-1.549440	0.474126
H	6.101361	-3.121637	-0.299829
H	5.186978	-2.677841	1.156481
H	2.495520	1.246305	2.358604
H	0.972146	0.437064	-3.514837
H	-0.660739	-0.297122	-3.603360
H	0.619149	-0.994732	-2.553957
C	-2.776000	0.531483	3.608184

H	-3.496343	0.157981	2.880607
H	-3.244891	1.348272	4.169286
H	-2.561729	-0.266587	4.316289
C	-0.459883	1.394036	4.008886
H	-0.861256	2.140783	4.702748
H	0.462693	1.794227	3.580642
H	-0.207038	0.506479	4.594534
C	-1.897883	2.366451	2.196123
H	-1.062502	2.792925	1.641147
H	-2.245537	3.109429	2.921551
H	-2.713641	2.185089	1.490792
C	2.110266	3.914825	0.138526
H	1.205629	4.300526	-0.339170
H	2.728162	3.475933	-0.652187
C	2.860677	5.082190	0.789163
H	3.796232	4.735380	1.235377
H	2.261403	5.537571	1.583245
H	3.095281	5.858623	0.055512
H	3.548891	1.691860	0.925715
C	-5.201125	-1.948169	0.268454
C	-6.123181	-2.307182	-0.675246
C	-4.769751	-1.375086	-2.450968
C	-5.904273	-2.021733	-2.045184
N	-2.688223	-0.325977	-1.889419
N	-3.086737	-0.952760	0.803165
H	-4.577978	-1.143969	-3.491914
H	-6.647459	-2.321709	-2.774779
H	-7.031185	-2.819389	-0.378650
H	-5.341093	-2.160674	1.321671

Zero-point correction= 0.809562 (Hartree/Particle)
Thermal correction to Energy= 0.856530
Thermal correction to Enthalpy= 0.857474
Thermal correction to Gibbs Free Energy= 0.732033
Sum of electronic and zero-point Energies= -2377.952934
Sum of electronic and thermal Energies= -2377.905965
Sum of electronic and thermal Enthalpies= -2377.905021
Sum of electronic and thermal Free Energies= -2378.030462

[(*S*)-Quinox-*t*Bu$_3$]: II-C$_{linear}$

C	-4.132081	-1.318847	0.093151
C	-4.002094	-0.616045	-1.127084
C	-1.981045	0.211995	-0.448312
C	-2.066113	-0.587983	0.741627
P	-0.582509	1.424299	-0.631973
P	-0.608055	-0.671729	1.883774
Cu	1.059880	0.599638	0.784616

B	2.430574	-0.502220	-0.247241
O	2.355634	-1.848644	-0.621306
O	3.659269	0.001091	-0.691661
C	3.633105	-2.296163	-1.102608
C	4.300113	-0.957598	-1.549668
C	1.675878	2.039185	2.448128
H	0.925069	1.865084	3.215741
C	2.601293	1.066385	2.194075
C	-1.403399	-0.169237	3.466342
H	-2.351292	-0.684583	3.628809
H	-1.573900	0.908588	3.441532
H	-0.723096	-0.386672	4.293269
C	-0.322274	-2.525533	2.086863
C	-0.117412	1.328372	-2.466765
C	-1.524064	3.034084	-0.247753
C	1.084463	-2.651674	2.692665
H	1.312217	-3.709300	2.865146
H	1.162508	-2.137438	3.657210
H	1.834710	-2.242967	2.012227
C	-0.327085	-3.175333	0.696717
H	0.360701	-2.670768	0.014567
H	-1.330363	-3.191616	0.263861
H	0.008100	-4.214409	0.791844
C	-1.345753	-3.215435	2.993669
H	-1.128400	-4.288917	3.026055
H	-2.362423	-3.081030	2.620663
H	-1.298714	-2.841704	4.020280
C	0.022816	-0.163453	-2.816070
H	-0.952075	-0.650317	-2.879279
H	0.637879	-0.704199	-2.093823
H	0.501000	-0.247935	-3.798312
C	-1.104115	1.964800	-3.451094
H	-2.103203	1.542451	-3.340575
H	-0.754751	1.764877	-4.470328
H	-1.157736	3.049408	-3.337376
C	1.256516	2.005267	-2.608046
H	1.609243	1.880304	-3.637798
H	1.996574	1.563145	-1.937904
H	1.205311	3.077805	-2.405749
C	-2.880096	3.245973	-0.933407
H	-3.604959	2.481011	-0.653537
H	-2.811196	3.260856	-2.018895
H	-3.279769	4.214292	-0.610102
C	-0.593890	4.205791	-0.593817
H	-0.981367	5.125849	-0.143008
H	-0.538925	4.366294	-1.672679

H	0.420113	4.048741	-0.222222
C	-1.775542	3.000714	1.268977
H	-0.857731	2.826767	1.831401
H	-2.489043	2.213477	1.532991
H	-2.208981	3.954660	1.587718
H	2.614326	0.138153	2.753603
C	4.345596	-2.959165	0.077427
H	5.307629	-3.388294	-0.215184
H	3.710210	-3.759799	0.463094
H	4.511223	-2.240629	0.883765
C	3.415199	-3.310974	-2.214700
H	2.943421	-4.207022	-1.804753
H	4.367650	-3.602634	-2.667989
H	2.763790	-2.912525	-2.993101
C	3.964272	-0.575095	-2.992023
H	4.478038	-1.218757	-3.711197
H	4.277211	0.457461	-3.162470
H	2.889118	-0.633868	-3.172439
C	5.804758	-0.889861	-1.334625
H	6.181267	0.076634	-1.677460
H	6.313838	-1.675371	-1.902041
H	6.060595	-0.997759	-0.280381
C	1.895468	3.470372	2.027585
H	0.940592	3.979203	1.873987
H	2.423768	3.487098	1.068086
C	2.702456	4.247677	3.073435
H	3.687103	3.794295	3.213851
H	2.192797	4.242869	4.041638
H	2.842934	5.289603	2.771563
H	3.494153	1.287439	1.619114
C	-5.302003	-2.074821	0.343606
C	-6.285734	-2.134810	-0.604050
C	-5.030127	-0.702385	-2.096133
C	-6.145994	-1.448529	-1.835253
H	-7.182976	-2.713774	-0.418418
H	-6.936651	-1.515741	-2.573488
H	-4.902917	-0.161573	-3.026267
H	-5.379872	-2.596626	1.289860
N	-2.920771	0.168879	-1.361295
N	-3.135167	-1.296408	1.012268

Zero-point correction= 0.810231 (Hartree/Particle)
Thermal correction to Energy= 0.856808
Thermal correction to Enthalpy= 0.857752
Thermal correction to Gibbs Free Energy= 0.734642
Sum of electronic and zero-point Energies= -2377.955087
Sum of electronic and thermal Energies= -2377.908511

Sum of electronic and thermal Enthalpies= -2377.907566
Sum of electronic and thermal Free Energies= -2378.030676

[(*S*)-Quinox-*t*Bu$_3$]: II-D$_{linear}$

C	3.904216	-1.276012	-0.744449
C	3.920607	-1.501245	0.650184
C	1.920117	-0.406991	0.861359
C	1.905770	-0.172658	-0.557831
P	0.495876	0.163798	1.910745
P	0.479373	0.714183	-1.364166
Cu	-1.118427	0.852162	0.305431
B	-2.447233	-0.697354	0.191036
O	-2.284874	-2.006865	0.664897
O	-3.719439	-0.600836	-0.383595
C	-1.732283	2.961525	0.795641
H	-0.934839	3.319079	1.442991
C	-2.608457	2.022722	1.268235
H	-2.531183	1.632105	2.276554
C	1.335073	2.244024	-2.104113
C	-0.030176	-0.489224	-2.745120
C	0.094578	-1.412933	2.757756
C	1.299948	1.194545	3.273913
C	-1.439505	-0.072931	-3.198333
H	-1.802726	-0.797137	-3.936103
H	-1.441881	0.909999	-3.674715
H	-2.143978	-0.051699	-2.364896
C	-0.098679	-1.896174	-2.126687
H	-0.657479	-1.916150	-1.188988
H	0.899229	-2.302871	-1.948502
H	-0.603322	-2.563065	-2.834563
C	0.896255	-0.547106	-3.964387
H	0.540362	-1.339260	-4.632704
H	1.923016	-0.776531	-3.677770
H	0.883948	0.383286	-4.535388
C	2.215105	0.408089	4.216687
H	3.028252	-0.077032	3.675960
H	1.663635	-0.361825	4.761989
H	2.644587	1.092675	4.957453
C	0.130976	1.788032	4.077342
H	0.522619	2.417367	4.883816
H	-0.483257	1.009149	4.539014
H	-0.518806	2.406494	3.452964
C	2.081487	2.333072	2.612903
H	1.450626	2.902730	1.926448
H	2.945009	1.956994	2.056332
H	2.453949	3.021688	3.378821

C	2.661821	2.038505	-2.847823
H	3.433297	1.626749	-2.197593
H	3.007668	3.017334	-3.200931
H	2.572691	1.386836	-3.713777
C	0.330314	2.926170	-3.044678
H	0.675380	3.941714	-3.267418
H	-0.665159	2.999678	-2.603919
H	0.242978	2.394482	-3.994266
C	1.626549	3.160431	-0.906325
H	0.734875	3.353498	-0.309703
H	2.021852	4.115969	-1.267358
H	2.381297	2.718251	-0.250500
H	-0.464811	-2.031589	2.054585
H	0.993647	-1.923278	3.106055
H	-0.565028	-1.201236	3.602871
C	-3.543602	-2.699627	0.618504
C	-4.311335	-1.906807	-0.484540
C	-4.178731	-2.564195	2.003610
H	-3.483771	-2.960037	2.747665
H	-5.119350	-3.116807	2.076674
H	-4.367484	-1.514672	2.240700
C	-3.292195	-4.165693	0.300728
H	-4.237472	-4.700146	0.164210
H	-2.752982	-4.633494	1.127793
H	-2.692026	-4.279057	-0.602373
C	-4.034576	-2.426254	-1.896209
H	-4.420627	-1.703039	-2.618126
H	-4.519076	-3.390157	-2.074284
H	-2.962278	-2.534436	-2.070494
C	-5.811985	-1.789768	-0.262780
H	-6.280135	-2.778819	-0.236658
H	-6.259397	-1.222630	-1.082191
H	-6.036329	-1.271302	0.669538
C	-2.073350	3.822222	-0.395208
H	-2.624070	3.221160	-1.127075
H	-1.164453	4.172946	-0.889608
C	-2.912288	5.040103	0.007422
H	-2.377950	5.657252	0.735963
H	-3.853931	4.725299	0.464539
H	-3.143226	5.665233	-0.860022
H	-3.545391	1.833098	0.755844
C	4.972415	-1.749434	-1.543576
C	6.012265	-2.415627	-0.956699
C	5.006363	-2.197073	1.233456
C	6.029348	-2.640710	0.441866
H	4.933085	-1.564904	-2.610512

H	6.834402	-2.778613	-1.562569
H	6.864868	-3.171544	0.883284
H	4.992847	-2.359558	2.304570
N	2.873965	-0.613628	-1.324887
N	2.901514	-1.064670	1.430531

Zero-point correction= 0.810367 (Hartree/Particle)
Thermal correction to Energy= 0.856831
Thermal correction to Enthalpy= 0.857775
Thermal correction to Gibbs Free Energy= 0.735246
Sum of electronic and zero-point Energies= -2377.953032
Sum of electronic and thermal Energies= -2377.906569
Sum of electronic and thermal Enthalpies= -2377.905625
Sum of electronic and thermal Free Energies= -2378.028154

[(*S*)-Quinox-*t*Bu$_3$]: TS-A$_{branch}$

C	4.393663	1.110139	-0.422081
C	4.456076	-0.298875	-0.498419
C	2.223495	-0.442584	-0.002031
C	2.161211	0.995740	0.072781
P	0.712987	-1.472760	0.362371
P	0.571249	1.841320	0.534820
Cu	-0.767990	0.090393	1.045343
B	-2.615074	0.008839	0.081575
O	-3.269634	0.956398	-0.690250
O	-3.021347	-1.266586	-0.299289
C	-1.473117	0.128120	2.923822
H	-1.538478	-0.805611	3.474549
H	-1.176400	0.966106	3.550905
C	-2.571840	0.422079	1.973814
H	-3.376145	-0.321582	2.021669
C	-3.120440	1.847714	2.086178
H	-3.694944	2.126527	1.202260
H	-2.271456	2.538742	2.137172
C	1.170346	2.914490	1.897780
H	2.090172	3.437025	1.631616
H	1.340537	2.279076	2.768261
H	0.391547	3.635886	2.154361
C	0.226065	3.040725	-0.873332
C	-1.145177	3.663498	-0.567154
H	-1.399611	4.376253	-1.359573
H	-1.140354	4.216611	0.377317
H	-1.929219	2.904767	-0.523697
C	0.119858	2.220031	-2.162047
H	-0.623367	1.426942	-2.057005
H	1.079473	1.771101	-2.436156
H	-0.191705	2.870331	-2.986371

C	1.275151	4.144954	-1.032169
H	1.010413	4.768803	-1.893378
H	2.273798	3.736428	-1.191053
H	1.309121	4.796704	-0.155063
C	-3.989455	2.007432	3.333120
H	-4.850846	1.332149	3.293900
H	-4.371784	3.028741	3.418094
H	-3.427479	1.773444	4.240347
C	-4.080435	0.312553	-1.686936
C	-4.175715	-1.161479	-1.149086
C	-3.356252	0.443364	-3.026282
H	-3.171535	1.502458	-3.219445
H	-3.957472	0.040811	-3.845490
H	-2.393880	-0.068183	-3.020301
C	-5.413310	1.046312	-1.767227
H	-6.095531	0.537215	-2.454337
H	-5.247352	2.059097	-2.140683
H	-5.890235	1.121154	-0.790033
C	-4.084784	-2.238069	-2.219995
H	-4.904294	-2.146429	-2.938794
H	-4.149047	-3.222912	-1.751943
H	-3.137071	-2.181473	-2.756611
C	-5.395141	-1.402388	-0.259490
H	-5.296033	-2.383452	0.209527
H	-6.328155	-1.380460	-0.828017
H	-5.454422	-0.654757	0.535464
C	1.297715	-2.596888	1.773347
C	0.472944	-2.448349	-1.246566
C	-0.064162	-1.422194	-2.251509
H	0.674742	-0.647456	-2.473605
H	-0.962537	-0.943526	-1.864551
H	-0.309564	-1.928142	-3.192076
C	0.048234	-3.261571	2.372307
H	0.336738	-3.806987	3.277485
H	-0.410097	-3.975981	1.687303
H	-0.699634	-2.516500	2.644376
C	1.708191	-3.114989	-1.863778
H	2.468967	-2.386808	-2.143612
H	1.390601	-3.639090	-2.772778
H	2.172657	-3.845131	-1.204425
C	-0.612997	-3.505035	-0.994753
H	-0.230460	-4.339554	-0.402855
H	-0.939221	-3.912498	-1.958404
H	-1.487386	-3.076566	-0.499443
C	2.319265	-3.673808	1.397739
H	1.876391	-4.445368	0.763932

H	2.662917	-4.166339	2.314420
H	3.186137	-3.251862	0.887738
C	1.900699	-1.663721	2.836744
H	1.197564	-0.868564	3.099544
H	2.839863	-1.217254	2.499798
H	2.114855	-2.247040	3.738419
C	5.560909	1.875811	-0.652644
C	6.737098	1.244446	-0.949990
C	5.685197	-0.929395	-0.804357
C	6.799697	-0.168257	-1.026833
N	3.226407	1.733153	-0.122139
N	3.351152	-1.053224	-0.273210
H	5.485309	2.954431	-0.583797
H	7.634698	1.825045	-1.128706
H	7.743787	-0.645695	-1.262247
H	5.705992	-2.011460	-0.856686

Zero-point correction= 0.810083 (Hartree/Particle)
Thermal correction to Energy= 0.855518
Thermal correction to Enthalpy= 0.856463
Thermal correction to Gibbs Free Energy= 0.736131
Sum of electronic and zero-point Energies= -2377.942494
Sum of electronic and thermal Energies= -2377.897058
Sum of electronic and thermal Enthalpies= -2377.896114
Sum of electronic and thermal Free Energies= -2378.016446

[(*S*)-Quinox-*t*Bu$_3$]: TS-B$_{branch}$

C	-4.417103	-0.444141	-0.462558
C	-4.262102	0.838691	-1.034918
C	-2.046570	0.834797	-0.467843
C	-2.184659	-0.506996	0.037608
P	-0.419030	1.716158	-0.299897
P	-0.669853	-1.426058	0.601278
Cu	0.818242	0.274956	0.884270
B	2.609358	-0.001368	-0.094959
O	3.118883	0.944454	-0.969699
O	3.122016	-1.254608	-0.393185
C	3.912344	0.300472	-1.980237
C	4.167192	-1.130154	-1.370669
C	1.448708	0.723057	2.725375
H	1.369510	1.758950	3.049861
H	1.226541	0.020942	3.523585
C	2.616072	0.412209	1.872709
C	-1.195134	-2.295081	2.192694
C	-0.451787	-2.646363	-0.837264
C	-0.068321	2.132817	-2.050887
C	-0.854672	3.358758	0.503606

C	0.696297	-3.599102	-0.475046
H	0.977753	-4.171679	-1.366326
H	0.393832	-4.318292	0.289888
H	1.580853	-3.055357	-0.134837
C	-0.016001	-1.782934	-2.030989
H	0.872918	-1.202899	-1.783584
H	-0.806915	-1.095594	-2.348263
H	0.216442	-2.433509	-2.881313
C	-1.685352	-3.453393	-1.259228
H	-1.413820	-4.066798	-2.126259
H	-2.517132	-2.811593	-1.551598
H	-2.039028	-4.125187	-0.479509
C	-1.679038	4.298993	-0.380034
H	-2.614563	3.836247	-0.694931
H	-1.125280	4.599230	-1.273979
H	-1.910035	5.210377	0.182744
C	0.493958	4.015194	0.837494
H	0.318009	4.978998	1.327002
H	1.089647	4.203978	-0.061094
H	1.081654	3.388862	1.511281
C	-1.603747	3.047331	1.805470
H	-1.046316	2.328778	2.413035
H	-2.599590	2.640281	1.608645
H	-1.728307	3.969154	2.383334
C	5.173427	1.125696	-2.202336
H	4.901051	2.103416	-2.605513
H	5.839582	0.634649	-2.917842
H	5.715594	1.284892	-1.270399
C	3.086239	0.288910	-3.266494
H	3.643487	-0.145866	-4.100076
H	2.828046	1.318795	-3.522271
H	2.156940	-0.268109	-3.142458
C	5.491285	-1.248421	-0.615773
H	6.349520	-1.189407	-1.289818
H	5.517011	-2.213355	-0.105256
H	5.585943	-0.467899	0.142139
C	4.037210	-2.276523	-2.363928
H	4.196679	-3.224844	-1.845942
H	4.784706	-2.190930	-3.157924
H	3.045113	-2.303027	-2.815506
C	3.690330	1.506981	1.815665
H	3.222732	2.445999	1.503771
H	4.449873	1.289047	1.061315
C	4.371892	1.680261	3.172104
H	5.148372	2.449195	3.122572
H	4.844478	0.745384	3.490544

H	3.651531	1.965467	3.941501
H	3.056089	-0.561996	2.095684
H	0.787378	2.809603	-2.085212
H	-0.931345	2.586441	-2.540804
H	0.213210	1.215519	-2.569655
C	-2.172607	-3.464445	2.046353
H	-3.059664	-3.178819	1.479962
H	-2.487390	-3.786412	3.045549
H	-1.702171	-4.324664	1.564681
C	0.095893	-2.787396	2.868523
H	-0.149387	-3.185075	3.859280
H	0.815422	-1.976836	2.993324
H	0.579408	-3.586165	2.303367
C	-1.825733	-1.207495	3.079743
H	-1.178790	-0.328976	3.147115
H	-1.966269	-1.611796	4.087761
H	-2.804276	-0.901034	2.703150
C	-5.379743	1.486757	-1.611535
C	-6.601646	0.872435	-1.595747
C	-5.692665	-1.056034	-0.452498
C	-6.760122	-0.406145	-1.008530
N	-3.055980	1.461373	-1.018944
N	-3.348685	-1.108533	0.047566
H	-5.232766	2.466595	-2.049896
H	-7.461895	1.365008	-2.033977
H	-7.738858	-0.871661	-1.005665
H	-5.785698	-2.037486	-0.003166

Zero-point correction= 0.809295 (Hartree/Particle)
Thermal correction to Energy= 0.854923
Thermal correction to Enthalpy= 0.855867
Thermal correction to Gibbs Free Energy= 0.735233
Sum of electronic and zero-point Energies= -2377.943144
Sum of electronic and thermal Energies= -2377.897516
Sum of electronic and thermal Enthalpies= -2377.896572
Sum of electronic and thermal Free Energies= -2378.017206

[(*S*)-Quinox-*t*Bu$_3$]: TS-C$_{branch}$

C	4.194230	-1.348972	0.222366
C	4.234825	-0.140330	0.952126
C	2.146299	0.407116	0.188133
C	2.084368	-0.849632	-0.512387
P	0.741770	1.613462	0.040383
P	0.545163	-1.317646	-1.451602
Cu	-0.850537	0.395235	-1.041495
B	-2.563926	-0.168746	0.054585
O	-3.012863	0.341413	1.264428

O	-3.000152	-1.486232	-0.078952
C	-3.662398	-0.697107	2.018759
C	-4.015834	-1.738132	0.904624
C	-1.859982	1.128961	-2.606540
H	-1.705125	2.162188	-2.909865
H	-1.989439	0.455331	-3.449793
C	-2.797207	0.951345	-1.465784
C	1.233232	-1.413974	-3.151701
H	2.157677	-1.993235	-3.180981
H	1.418308	-0.398691	-3.502685
H	0.488862	-1.865544	-3.810234
C	0.212753	-3.115478	-1.002804
C	0.443115	2.183090	1.817080
C	1.536637	2.967213	-1.030218
C	-1.093640	-3.484768	-1.725265
H	-1.360162	-4.516307	-1.468177
H	-0.976182	-3.442732	-2.812565
H	-1.916069	-2.833525	-1.428030
C	-0.002799	-3.168797	0.513525
H	-0.772321	-2.455813	0.815934
H	0.919992	-2.950575	1.058865
H	-0.334957	-4.171833	0.802689
C	1.308426	-4.101300	-1.421465
H	1.006589	-5.111130	-1.120925
H	2.266510	-3.868920	-0.958867
H	1.444890	-4.112841	-2.506177
C	0.353745	0.897542	2.655490
H	1.335088	0.443804	2.808463
H	-0.308973	0.166050	2.187132
H	-0.062309	1.141851	3.638588
C	1.494614	3.117252	2.422099
H	2.503142	2.717409	2.312869
H	1.282615	3.239433	3.490488
H	1.450878	4.110918	1.970612
C	-0.931314	2.871346	1.846902
H	-1.138794	3.192121	2.874256
H	-1.725282	2.190614	1.540235
H	-0.965750	3.760830	1.213918
C	2.868964	3.549879	-0.545460
H	3.623483	2.777694	-0.391582
H	2.770807	4.111774	0.381364
H	3.242426	4.239485	-1.311026
C	0.500306	4.088454	-1.180984
H	0.848666	4.806177	-1.931281
H	0.356085	4.634090	-0.245363
H	-0.463485	3.693549	-1.512380

C	1.765094	2.320578	-2.406618
H	0.840748	1.889883	-2.799702
H	2.528385	1.536415	-2.359069
H	2.125120	3.084562	-3.103857
H	-3.601202	0.244447	-1.702604
C	-3.366682	2.265442	-0.919648
H	-3.820061	2.122685	0.062494
H	-2.542070	2.971999	-0.778901
C	-2.661507	-1.225401	3.044244
H	-3.117050	-1.977041	3.693763
H	-2.318221	-0.393791	3.662935
H	-1.788356	-1.666473	2.560098
C	-4.862014	-0.095425	2.735981
H	-4.517467	0.621666	3.484241
H	-5.436480	-0.873680	3.247185
H	-5.520233	0.429711	2.043844
C	-5.362050	-1.460302	0.235278
H	-6.199167	-1.678883	0.902779
H	-5.449346	-2.091572	-0.651490
H	-5.434761	-0.417170	-0.083529
C	-3.933193	-3.194319	1.333940
H	-4.179622	-3.838577	0.486906
H	-4.643985	-3.401865	2.139230
H	-2.930104	-3.452682	1.673946
C	-4.400551	2.859965	-1.875509
H	-4.807342	3.795354	-1.480588
H	-5.236399	2.167030	-2.019532
H	-3.964206	3.059851	-2.856697
C	5.377722	0.173418	1.725088
C	6.434289	-0.694744	1.754231
C	5.301942	-2.228252	0.264108
C	6.396681	-1.903083	1.016998
H	5.382780	1.107393	2.274301
H	7.313591	-0.459153	2.342534
H	7.246620	-2.574680	1.053998
H	5.247271	-3.148100	-0.305843
N	3.197651	0.733163	0.899979
N	3.099677	-1.676847	-0.509486

Zero-point correction= 0.810352 (Hartree/Particle)
Thermal correction to Energy= 0.855514
Thermal correction to Enthalpy= 0.856458
Thermal correction to Gibbs Free Energy= 0.737865
Sum of electronic and zero-point Energies= -2377.942395
Sum of electronic and thermal Energies= -2377.897234
Sum of electronic and thermal Enthalpies= -2377.896290
Sum of electronic and thermal Free Energies= -2378.014882

[(*S*)-Quinox-*t*Bu$_3$]: TS-D$_{branch}$

C	4.252971	-0.065881	-0.965898
C	4.056374	-1.436647	-0.686955
C	1.946850	-0.988877	0.076668
C	2.138633	0.407148	-0.220886
P	0.319656	-1.581184	0.747846
P	0.737287	1.606756	0.040044
Cu	-0.896039	0.279326	0.924693
B	-2.560083	-0.232381	-0.190739
O	-3.095793	0.366545	-1.322227
O	-2.956904	-1.569506	-0.136988
C	-1.841040	0.819712	2.614576
H	-1.970476	0.069310	3.389327
H	-1.619510	1.807011	3.014228
C	-2.794520	0.791879	1.488891
H	-3.593608	0.056682	1.631349
C	-3.351468	2.148309	1.058014
H	-3.817470	2.080956	0.073261
H	-2.520564	2.853993	0.957332
C	1.493382	2.890480	1.211701
C	0.453975	2.279930	-1.705233
C	-0.069672	-2.872004	-0.493648
C	0.736999	-2.537782	2.310059
C	-0.918565	2.971834	-1.706990
H	-1.118193	3.356355	-2.713726
H	-0.957168	3.819529	-1.019241
H	-1.715559	2.273941	-1.446527
C	0.363313	1.045302	-2.617599
H	-0.326056	0.303057	-2.206474
H	1.339273	0.580020	-2.776169
H	-0.024166	1.352326	-3.594748
C	1.520461	3.236692	-2.243998
H	1.339705	3.404099	-3.311930
H	2.527619	2.834729	-2.122673
H	1.466127	4.209805	-1.751613
C	1.568559	-3.802270	2.079235
H	2.514733	-3.582391	1.583470
H	1.026842	-4.533975	1.474105
H	1.780731	-4.271635	3.046350
C	-0.620267	-2.914476	2.924123
H	-0.456878	-3.439588	3.871302
H	-1.193565	-3.577551	2.269476
H	-1.222944	-2.025212	3.119576
C	1.470407	-1.575835	3.250612
H	0.890647	-0.661265	3.399200

H	2.455872	-1.305601	2.858931
H	1.617388	-2.054999	4.224177
C	-4.364272	2.683644	2.069870
H	-5.202884	1.988334	2.182366
H	-4.770180	3.646869	1.747692
H	-3.908744	2.813700	3.054417
C	2.799329	3.567536	0.779430
H	3.569659	2.842333	0.519207
H	3.164813	4.173705	1.616160
H	2.662670	4.234607	-0.070527
C	0.421225	3.959542	1.462755
H	0.755176	4.623255	2.267473
H	-0.527757	3.511613	1.766959
H	0.250942	4.577898	0.577971
C	1.748535	2.129043	2.523386
H	0.838752	1.643951	2.883029
H	2.097277	2.836707	3.283029
H	2.523604	1.365903	2.401169
H	-0.246683	-2.371278	-1.447087
H	0.746960	-3.586111	-0.603583
H	-0.994875	-3.370707	-0.203327
C	-3.728801	-0.637946	-2.134691
C	-4.012454	-1.767400	-1.091041
C	-2.727270	-1.053212	-3.210919
H	-2.412352	-0.163724	-3.760472
H	-3.164775	-1.763447	-3.917030
H	-1.839490	-1.505855	-2.764763
C	-4.966350	-0.036185	-2.782128
H	-5.526997	-0.801406	-3.327421
H	-4.667908	0.738716	-3.491525
H	-5.622833	0.417723	-2.039946
C	-5.335244	-1.570748	-0.349849
H	-5.374485	-2.269176	0.488858
H	-6.194731	-1.757873	-0.998361
H	-5.415976	-0.556598	0.050070
C	-3.923941	-3.182352	-1.640329
H	-4.664704	-3.337935	-2.430418
H	-4.124330	-3.899114	-0.840689
H	-2.932937	-3.391203	-2.044002
C	5.485805	0.367934	-1.508551
C	6.473805	-0.544385	-1.758125
C	5.091928	-2.361773	-0.958136
C	6.274903	-1.918868	-1.482571
H	5.611959	1.424582	-1.712232
H	7.419873	-0.217713	-2.174009
H	7.071333	-2.623372	-1.692603

H	4.913229	-3.407882	-0.740215
N	3.266951	0.837000	-0.731958
N	2.880913	-1.873943	-0.169019

Zero-point correction= 0.809373 (Hartree/Particle)
Thermal correction to Energy= 0.854925
Thermal correction to Enthalpy= 0.855869
Thermal correction to Gibbs Free Energy= 0.735553
Sum of electronic and zero-point Energies= -2377.945638
Sum of electronic and thermal Energies= -2377.900085
Sum of electronic and thermal Enthalpies= -2377.899141
Sum of electronic and thermal Free Energies= -2378.019458

[(*S*)-Quinox-*t*Bu$_3$]: TS-A$_{linear}$

C	4.139142	1.256740	-0.609672
C	4.189493	-0.092389	-1.024981
C	2.092267	-0.449443	-0.176004
C	2.019293	0.940717	0.192655
P	0.698729	-1.594851	0.279525
P	0.458093	1.626833	0.933567
Cu	-0.906868	-0.160853	0.983899
B	-2.656372	-0.043529	-0.202730
O	-3.226606	1.145710	-0.647389
O	-3.040224	-1.081990	-1.044009
C	-1.847359	-0.431802	2.743634
H	-1.735857	-1.416163	3.198199
C	-2.830281	-0.391253	1.635445
H	-3.338374	-1.337354	1.450753
C	1.104096	2.257343	2.532194
H	2.026557	2.823665	2.394876
H	1.283777	1.405679	3.189181
H	0.346127	2.888315	3.000332
C	0.114666	3.184407	-0.061604
C	-1.287828	3.642697	0.369094
H	-1.538195	4.570328	-0.158203
H	-1.330513	3.858088	1.442139
H	-2.046397	2.894804	0.131931
C	0.093479	2.778678	-1.538949
H	-0.523993	1.890478	-1.690167
H	1.100002	2.573959	-1.913363
H	-0.329116	3.590771	-2.140049
C	1.116814	4.320348	0.165328
H	0.860266	5.156361	-0.495201
H	2.138756	4.007139	-0.048512
H	1.075041	4.693158	1.192332
C	-3.876060	0.919359	-1.910448
C	-4.110637	-0.627912	-1.887737

C	-2.921507	1.361180	-3.018186
H	-2.648423	2.405279	-2.853701
H	-3.388480	1.274845	-4.002470
H	-2.005786	0.765999	-3.014910
C	-5.144516	1.758152	-1.960277
H	-5.712883	1.544564	-2.870540
H	-4.881826	2.818346	-1.963159
H	-5.780736	1.569235	-1.095835
C	-3.995007	-1.318408	-3.237167
H	-4.742632	-0.932747	-3.936669
H	-4.164714	-2.390153	-3.112378
H	-3.003848	-1.182097	-3.670438
C	-5.416990	-1.024652	-1.200363
H	-5.419981	-2.106228	-1.049576
H	-6.289239	-0.755163	-1.801140
H	-5.507171	-0.546792	-0.221660
C	1.503692	-2.574382	1.695926
C	0.425763	-2.641967	-1.270824
C	0.180145	-1.623243	-2.397501
H	1.091165	-1.079230	-2.658774
H	-0.596545	-0.909016	-2.113437
H	-0.161343	-2.156652	-3.290932
C	0.488157	-3.627818	2.159286
H	0.818358	-4.054121	3.112467
H	0.405650	-4.448895	1.443575
H	-0.502036	-3.189037	2.308115
C	1.557972	-3.592646	-1.671776
H	2.523699	-3.089083	-1.706857
H	1.338079	-3.999680	-2.665408
H	1.622213	-4.440559	-0.986528
C	-0.863231	-3.453453	-1.062626
H	-0.750843	-4.211161	-0.284871
H	-1.095595	-3.977889	-1.996858
H	-1.711249	-2.812748	-0.821359
C	2.847996	-3.245836	1.395028
H	2.769658	-4.027447	0.641217
H	3.216696	-3.710828	2.316378
H	3.598061	-2.529372	1.058197
C	1.713171	-1.560667	2.833944
H	0.774703	-1.068344	3.100024
H	2.447353	-0.795362	2.561252
H	2.101519	-2.087687	3.711806
C	-1.892481	0.691819	3.758499
H	-1.969468	1.653630	3.232975
H	-0.946640	0.729960	4.313807
C	-3.046644	0.582574	4.762547

H	-2.981604	-0.357724	5.319035
H	-4.011936	0.592432	4.246288
H	-3.042019	1.404993	5.486165
H	-3.546395	0.430869	1.713040
C	5.247319	2.103632	-0.846895
C	6.353215	1.609947	-1.482624
C	5.343699	-0.579521	-1.682383
C	6.401117	0.259247	-1.904554
N	3.153286	-0.932398	-0.774780
N	3.032662	1.748489	0.003358
H	5.183568	3.133470	-0.516266
H	7.204180	2.254781	-1.669091
H	7.288910	-0.109492	-2.405104
H	5.357474	-1.618552	-1.989346

Zero-point correction= 0.809347 (Hartree/Particle)
Thermal correction to Energy= 0.854964
Thermal correction to Enthalpy= 0.855908
Thermal correction to Gibbs Free Energy= 0.734228
Sum of electronic and zero-point Energies= -2377.943323
Sum of electronic and thermal Energies= -2377.897707
Sum of electronic and thermal Enthalpies= -2377.896763
Sum of electronic and thermal Free Energies= -2378.018443

[(*S*)-Quinox-*t*Bu$_3$]: TS-B$_{linear}$

C	-4.199918	-0.601591	-0.856651
C	-3.999503	0.636726	-1.506034
C	-1.898606	0.778612	-0.610384
C	-2.094621	-0.489991	0.041712
P	-0.273816	1.670907	-0.466090
P	-0.703652	-1.250484	1.015141
Cu	0.974750	0.312487	0.791511
B	2.598449	-0.226289	-0.390254
O	3.158169	0.707125	-1.260326
O	2.997844	-1.506096	-0.757205
C	3.815045	0.006496	-2.332352
C	4.079990	-1.397219	-1.696744
C	1.987021	0.777531	2.490709
H	1.682054	0.165987	3.339785
C	2.843971	0.075029	1.526006
C	-1.453282	-1.430875	2.746689
C	-0.470017	-2.925167	0.161900
C	0.099024	1.900644	-2.244379
C	-0.709655	3.397606	0.143456
C	0.854938	-3.518034	0.668788
H	1.035570	-4.465306	0.147555
H	0.828466	-3.734893	1.738056

H	1.696507	-2.858183	0.457614
C	-0.312599	-2.610945	-1.336040
H	0.445304	-1.840209	-1.498568
H	-1.255827	-2.291934	-1.787068
H	0.017254	-3.517150	-1.854670
C	-1.593161	-3.949249	0.352297
H	-1.421652	-4.790351	-0.329046
H	-2.574554	-3.524150	0.138296
H	-1.595410	-4.353277	1.367088
C	-1.668440	4.186280	-0.752949
H	-2.626101	3.681269	-0.876437
H	-1.244652	4.357427	-1.745760
H	-1.846433	5.167943	-0.299631
C	0.626759	4.147863	0.234834
H	0.458202	5.137361	0.672768
H	1.078165	4.294778	-0.750637
H	1.339120	3.614211	0.863874
C	-1.305198	3.238033	1.545758
H	-0.642540	2.651833	2.187404
H	-2.279712	2.741242	1.509475
H	-1.450020	4.223219	2.001077
C	5.066417	0.775972	-2.726410
H	4.784451	1.734991	-3.166524
H	5.646633	0.217565	-3.467314
H	5.699071	0.973688	-1.861184
C	2.847773	-0.056361	-3.513745
H	3.309752	-0.527802	-4.384764
H	2.553309	0.958870	-3.786429
H	1.946061	-0.615212	-3.251698
C	5.378570	-1.451479	-0.891723
H	6.257959	-1.423140	-1.539955
H	5.398559	-2.381934	-0.320345
H	5.439026	-0.619861	-0.185615
C	4.013337	-2.568696	-2.663043
H	4.206483	-3.498545	-2.123531
H	4.768560	-2.466580	-3.448122
H	3.030354	-2.647039	-3.127684
H	3.088173	-0.959266	1.759919
H	1.039479	2.444057	-2.342537
H	-0.708621	2.415414	-2.765655
H	0.240758	0.912211	-2.682314
C	-2.819278	-2.117003	2.861986
H	-3.584005	-1.604676	2.278660
H	-3.123491	-2.097439	3.914934
H	-2.797702	-3.157930	2.544672
C	-0.428103	-2.181595	3.609104

H	-0.692646	-2.068154	4.665701
H	0.582790	-1.788501	3.471857
H	-0.420894	-3.250960	3.385499
C	-1.593863	0.005424	3.277201
H	-0.638418	0.532487	3.260246
H	-1.965943	-0.029503	4.306759
H	-2.312335	0.580724	2.685421
C	2.372915	2.186441	2.887127
H	1.492969	2.753739	3.216317
H	2.760355	2.712516	2.006435
C	3.434022	2.242440	3.992396
H	4.346637	1.726182	3.677924
H	3.071673	1.748022	4.899231
H	3.698158	3.273229	4.252183
H	3.732735	0.632996	1.217329
C	-5.430002	-1.282989	-1.015661
C	-6.411838	-0.735165	-1.794570
C	-5.028796	1.181367	-2.309543
C	-6.210039	0.505955	-2.446031
N	-2.826431	1.304225	-1.371184
N	-3.220871	-1.148251	-0.092612
H	-4.847484	2.131385	-2.797801
H	-7.003029	0.918555	-3.058941
H	-7.356678	-1.251440	-1.918852
H	-5.558596	-2.231902	-0.508721

Zero-point correction= 0.809964 (Hartree/Particle)
Thermal correction to Energy= 0.855386
Thermal correction to Enthalpy= 0.856330
Thermal correction to Gibbs Free Energy= 0.735883
Sum of electronic and zero-point Energies= -2377.940732
Sum of electronic and thermal Energies= -2377.895310
Sum of electronic and thermal Enthalpies= -2377.894366
Sum of electronic and thermal Free Energies= -2378.014813

[(*S*)-Quinox-*t*Bu$_3$]: TS-C$_{linear}$

C	4.190105	-1.367446	0.002062
C	4.205417	-0.310261	0.939560
C	2.101109	0.313530	0.290186
C	2.058377	-0.805067	-0.612355
P	0.688246	1.521066	0.344503
P	0.505035	-1.153206	-1.571956
Cu	-0.917596	0.504253	-0.864004
B	-2.623862	-0.259389	0.135631
O	-2.940553	-1.614324	0.171539
O	-3.256945	0.389348	1.191971
C	-4.044052	-1.807533	1.071552

C	-3.893168	-0.585995	2.037078
C	-1.924788	1.341040	-2.409252
H	-1.712908	0.872632	-3.370559
C	-2.835804	0.549456	-1.553252
C	1.162285	-1.054884	-3.284976
H	2.094037	-1.612151	-3.396389
H	1.327310	-0.003155	-3.522853
H	0.413465	-1.442036	-3.978966
C	0.181884	-2.987845	-1.306504
C	0.422170	1.822173	2.189743
C	1.479044	3.020423	-0.513666
C	-1.163412	-3.272445	-1.995058
H	-1.413528	-4.330673	-1.859398
H	-1.113365	-3.086821	-3.072763
H	-1.968390	-2.677720	-1.562030
C	0.041539	-3.212215	0.204825
H	-0.715110	-2.548368	0.628858
H	0.992286	-3.056217	0.722972
H	-0.273745	-4.244660	0.390849
C	1.250796	-3.919562	-1.886040
H	0.969418	-4.957400	-1.674401
H	2.232900	-3.728830	-1.454450
H	1.328029	-3.818118	-2.971987
C	0.355023	0.424244	2.825087
H	1.347093	-0.020109	2.925363
H	-0.271772	-0.246876	2.232378
H	-0.086278	0.502598	3.823782
C	1.483169	2.656377	2.913022
H	2.485575	2.258380	2.754914
H	1.267897	2.637111	3.987559
H	1.456974	3.702227	2.598872
C	-0.951032	2.497093	2.340246
H	-1.152850	2.644095	3.407799
H	-1.751951	1.885824	1.921979
H	-0.980256	3.479524	1.864063
C	2.863771	3.454896	-0.017612
H	3.609234	2.668268	-0.137306
H	2.866362	3.762003	1.026330
H	3.185681	4.313750	-0.617562
C	0.498110	4.193128	-0.382042
H	0.809583	5.000933	-1.052805
H	0.485892	4.595973	0.633727
H	-0.517768	3.904143	-0.656456
C	1.608265	2.630370	-1.995376
H	0.654049	2.296299	-2.408689
H	2.341911	1.827947	-2.129595

H	1.964137	3.497022	-2.562535
H	-3.209971	-0.367009	-2.007934
C	-5.319519	-1.751005	0.230908
H	-6.211292	-1.937339	0.834397
H	-5.261656	-2.515410	-0.546885
H	-5.426387	-0.778896	-0.256656
C	-3.908414	-3.172261	1.727684
H	-4.017259	-3.952435	0.970933
H	-4.685884	-3.317625	2.483602
H	-2.933145	-3.293834	2.199222
C	-2.961559	-0.871735	3.212717
H	-3.434946	-1.532014	3.943912
H	-2.711796	0.071212	3.703549
H	-2.032323	-1.334489	2.877453
C	-5.204405	-0.007125	2.546548
H	-4.998079	0.834097	3.211977
H	-5.765005	-0.759025	3.110073
H	-5.826391	0.353956	1.727342
C	-2.206738	2.824584	-2.518952
H	-1.321765	3.355647	-2.891367
H	-2.402286	3.231574	-1.518089
C	-3.396047	3.163347	-3.426516
H	-4.313563	2.698516	-3.051503
H	-3.226118	2.783891	-4.439106
H	-3.567013	4.243200	-3.494038
H	-3.651903	1.137806	-1.126056
C	5.323088	-2.205810	-0.124492
C	6.415969	-1.991734	0.669428
C	5.346287	-0.111668	1.752805
C	6.426444	-0.939258	1.616825
H	7.285558	-2.632275	0.578510
H	7.304342	-0.790872	2.234878
H	5.331209	0.704202	2.465536
H	5.288465	-3.005169	-0.854996
N	3.148721	0.533204	1.046865
N	3.094164	-1.593014	-0.765276

Zero-point correction= 0.809662 (Hartree/Particle)
Thermal correction to Energy= 0.855221
Thermal correction to Enthalpy= 0.856165
Thermal correction to Gibbs Free Energy= 0.735087
Sum of electronic and zero-point Energies= -2377.940948
Sum of electronic and thermal Energies= -2377.895389
Sum of electronic and thermal Enthalpies= -2377.894445
Sum of electronic and thermal Free Energies= -2378.015523

[(*S*)-Quinox-*t*Bu$_3$]: TS-D$_{linear}$

C	4.172194	-0.331052	-1.016182
C	4.009438	-1.586131	-0.388001
C	1.901974	-0.991000	0.277382
C	2.067038	0.287918	-0.360770
P	0.282898	-1.420426	1.079231
P	0.671624	1.521497	-0.371276
Cu	-0.978470	0.454200	0.745978
B	-2.604562	-0.339067	-0.296064
O	-2.879287	-1.704862	-0.283518
O	-3.278635	0.262000	-1.353561
C	-1.970983	1.238691	2.335605
H	-1.787858	0.718208	3.275001
C	-2.890721	0.544661	1.423664
H	-3.327253	-0.376574	1.807940
C	1.447223	3.017565	0.497401
C	0.413290	1.830628	-2.219932
C	-0.072412	-2.988090	0.196311
C	0.715055	-1.962839	2.827256
C	-0.959191	2.505404	-2.376351
H	-1.152018	2.664942	-3.443669
H	-0.997022	3.481176	-1.887468
H	-1.761031	1.883536	-1.974805
C	0.344039	0.438312	-2.866964
H	-0.305582	-0.227246	-2.292400
H	1.332164	-0.019438	-2.953173
H	-0.076232	0.529152	-3.873648
C	1.483864	2.664907	-2.927859
H	1.286916	2.646914	-4.005879
H	2.485551	2.269860	-2.753931
H	1.452947	3.710117	-2.612251
C	1.599573	-3.209179	2.913694
H	2.550852	-3.066223	2.400429
H	1.107596	-4.084025	2.480721
H	1.800608	-3.433286	3.967450
C	-0.636584	-2.241773	3.503841
H	-0.471239	-2.497471	4.555882
H	-1.157430	-3.083056	3.037086
H	-1.291505	-1.368586	3.463013
C	1.394487	-0.776200	3.518302
H	0.778427	0.123747	3.446408
H	2.372569	-0.563893	3.075758
H	1.550932	-1.005079	4.577710
C	2.828208	3.481606	0.017418
H	3.582391	2.702634	0.122928
H	3.131619	4.333615	0.636756

H	2.830571	3.810476	-1.020038
C	0.454184	4.181650	0.375365
H	0.752274	4.981564	1.061422
H	-0.561658	3.880445	0.635465
H	0.447573	4.600307	-0.634091
C	1.573294	2.604747	1.972380
H	0.621129	2.256100	2.375340
H	1.919168	3.463769	2.557136
H	2.307486	1.802516	2.096133
H	-0.329920	-2.740337	-0.834262
H	0.785638	-3.661292	0.216497
H	-0.949190	-3.458992	0.641553
C	-3.987214	-1.954988	-1.164505
C	-3.885569	-0.757876	-2.166117
C	-5.256455	-1.912351	-0.313361
H	-5.168780	-2.652396	0.485070
H	-6.147794	-2.142416	-0.902468
H	-5.387535	-0.930208	0.147395
C	-3.822091	-3.331356	-1.789199
H	-4.601665	-3.514647	-2.534716
H	-3.904864	-4.097281	-1.014765
H	-2.848444	-3.438763	-2.267692
C	-2.946672	-1.041321	-3.337257
H	-2.730556	-0.101898	-3.850533
H	-3.395889	-1.735879	-4.051799
H	-2.001172	-1.461959	-2.990892
C	-5.218694	-0.240861	-2.684773
H	-5.755683	-1.028755	-3.221767
H	-5.046258	0.586551	-3.376648
H	-5.848292	0.122356	-1.872358
C	-2.185615	2.726760	2.519629
H	-2.369517	3.188311	1.541107
H	-1.281689	3.205641	2.914914
C	-3.360125	3.061185	3.447162
H	-3.201138	2.627332	4.439496
H	-4.292988	2.645952	3.052777
H	-3.493428	4.141573	3.567647
H	-3.642726	1.187740	0.963484
C	5.384672	-0.033265	-1.682512
C	6.387601	-0.962470	-1.710359
C	5.060606	-2.532298	-0.434086
C	6.224016	-2.221153	-1.082247
H	5.483645	0.936407	-2.155635
H	7.319445	-0.739986	-2.217198
H	7.032020	-2.942536	-1.119739
H	4.909083	-3.488648	0.051905

N	3.175186	0.588180	-0.993915
N	2.850408	-1.895136	0.245638

Zero-point correction= 0.810810 (Hartree/Particle)
Thermal correction to Energy= 0.855967
Thermal correction to Enthalpy= 0.856912
Thermal correction to Gibbs Free Energy= 0.737505
Sum of electronic and zero-point Energies= -2377.940245
Sum of electronic and thermal Energies= -2377.895088
Sum of electronic and thermal Enthalpies= -2377.894144
Sum of electronic and thermal Free Energies= -2378.013551

[(*S*)-Quinox-*t*Bu$_3$]: III-A$_{branch}$

C	-4.478049	-1.402486	-0.281532
C	-4.703081	-0.027883	-0.522646
C	-2.489335	0.417568	-0.140265
C	-2.257009	-0.987173	0.080232
P	-1.077286	1.610764	0.094530
P	-0.542372	-1.588993	0.499722
Cu	0.704509	0.318448	0.842225
B	4.086606	-0.144154	0.782881
O	3.840262	-1.046613	-0.236987
O	4.932307	0.859159	0.365088
C	2.217515	0.830284	2.011864
H	2.635848	1.803094	1.716602
H	1.774757	1.015724	3.004561
C	3.395602	-0.176511	2.175922
H	4.098392	0.190741	2.941143
C	2.940770	-1.582961	2.588884
H	3.816278	-2.235812	2.696480
H	2.337083	-2.008915	1.779997
C	-0.912965	-2.565013	2.007908
H	-1.760927	-3.234840	1.859353
H	-1.132526	-1.870067	2.820458
H	-0.022167	-3.131092	2.287462
C	-0.172805	-2.876891	-0.820683
C	1.294621	-3.281760	-0.614285
H	1.584752	-3.992307	-1.396433
H	1.441050	-3.781936	0.347996
H	1.977237	-2.429391	-0.645695
C	-0.331659	-2.196438	-2.184699
H	0.251876	-1.274921	-2.242608
H	-1.377370	-1.953903	-2.395680
H	0.020397	-2.869213	-2.973411
C	-1.063198	-4.120706	-0.751432
H	-0.789884	-4.799709	-1.566828
H	-2.121048	-3.872036	-0.844476

H	-0.919778	-4.663001	0.186506
C	2.133020	-1.613648	3.883826
H	2.689799	-1.151624	4.705935
H	1.888566	-2.639420	4.179352
H	1.195001	-1.063330	3.769511
C	4.286115	-0.444507	-1.462865
C	5.389519	0.545016	-0.959196
C	3.075968	0.272711	-2.061919
H	2.285230	-0.464276	-2.219155
H	3.307182	0.742513	-3.021118
H	2.698332	1.032482	-1.371407
C	4.774198	-1.531380	-2.406358
H	5.199116	-1.091767	-3.313573
H	3.935801	-2.169782	-2.695524
H	5.529426	-2.158454	-1.932733
C	5.502283	1.832821	-1.760374
H	5.748241	1.619471	-2.805121
H	6.295580	2.455845	-1.341952
H	4.572673	2.401176	-1.725603
C	6.761621	-0.113616	-0.820211
H	7.416452	0.558545	-0.262220
H	7.214366	-0.312825	-1.794996
H	6.690100	-1.053193	-0.267604
C	-1.683229	2.670492	1.538339
C	-0.992156	2.546182	-1.540874
C	-0.513669	1.496600	-2.557320
H	-1.251389	0.700387	-2.693659
H	0.432028	1.044193	-2.251437
H	-0.361020	1.976856	-3.529511
C	-0.485827	3.487906	2.049853
H	-0.786752	4.026052	2.955369
H	-0.152854	4.230350	1.322661
H	0.363850	2.846642	2.293899
C	-2.284958	3.180267	-2.063286
H	-3.082436	2.445532	-2.169843
H	-2.085019	3.620834	-3.046836
H	-2.647878	3.975421	-1.413364
C	0.093622	3.621919	-1.392166
H	-0.252601	4.458969	-0.781830
H	0.344807	4.019520	-2.381117
H	1.006077	3.220335	-0.941808
C	-2.861665	3.600582	1.241267
H	-2.555393	4.437306	0.609683
H	-3.229199	4.023919	2.182887
H	-3.685326	3.076636	0.753417
C	-2.078194	1.667653	2.637805

H	-1.270551	0.955695	2.839822
H	-2.984080	1.112075	2.382086
H	-2.273962	2.214236	3.565739
C	-5.559068	-2.312686	-0.360006
C	-6.808286	-1.853973	-0.673640
C	-6.006174	0.422220	-0.844230
C	-7.033366	-0.477021	-0.918130
N	-3.237285	-1.855742	0.025514
N	-3.687392	0.866523	-0.431755
H	-5.360634	-3.360191	-0.167636
H	-7.639190	-2.547081	-0.735728
H	-8.033516	-0.138370	-1.162512
H	-6.152509	1.480900	-1.022000

Zero-point correction= 0.810438 (Hartree/Particle)
Thermal correction to Energy= 0.856685
Thermal correction to Enthalpy= 0.857630
Thermal correction to Gibbs Free Energy= 0.733649
Sum of electronic and zero-point Energies= -2377.989298
Sum of electronic and thermal Energies= -2377.943051
Sum of electronic and thermal Enthalpies= -2377.942106
Sum of electronic and thermal Free Energies= -2378.066087

[(*S*)-Quinox-*t*Bu$_3$]: III-B$_{branch}$

C	-4.708627	-0.260253	0.138592
C	-4.360456	-1.629188	0.073425
C	-2.141518	-1.074505	0.027305
C	-2.494116	0.316723	0.152649
P	-0.348121	-1.567420	-0.110455
P	-1.135991	1.575575	0.318849
Cu	0.723121	0.472707	-0.461189
B	3.860380	0.418756	-0.158982
O	4.145803	-0.905195	-0.424383
O	3.949123	0.691512	1.191177
C	4.629288	-1.517470	0.778157
C	4.092841	-0.551759	1.895558
C	2.098665	0.947355	-1.806954
H	2.302026	0.076326	-2.449161
H	1.674680	1.710175	-2.478108
C	3.459095	1.448785	-1.255372
C	-1.638673	2.985300	-0.826139
C	-1.250217	2.007489	2.153932
C	-0.261774	-2.813591	1.237807
C	-0.247620	-2.574676	-1.694261
C	-0.220414	3.111816	2.430440
H	-0.107894	3.240793	3.512039
H	-0.542047	4.071034	2.018448

H	0.760592	2.866693	2.013172
C	-0.818266	0.728000	2.891029
H	0.160448	0.378879	2.554640
H	-1.542283	-0.081125	2.752146
H	-0.757089	0.932261	3.965080
C	-2.626883	2.429888	2.675794
H	-2.557779	2.584913	3.758532
H	-3.385602	1.667713	2.492314
H	-2.973398	3.360417	2.229059
C	-1.090308	-3.852231	-1.702663
H	-2.150782	-3.643705	-1.559879
H	-0.773197	-4.549877	-0.922629
H	-0.959800	-4.357423	-2.666062
C	1.239522	-2.933655	-1.852756
H	1.391756	-3.409072	-2.827471
H	1.562774	-3.649157	-1.090173
H	1.885522	-2.054291	-1.792278
C	-0.674225	-1.646523	-2.840168
H	-0.063339	-0.739229	-2.863691
H	-1.727372	-1.360129	-2.756464
H	-0.544984	-2.164342	-3.796057
C	6.154780	-1.542221	0.684950
H	6.439866	-2.080509	-0.221191
H	6.606505	-2.041818	1.545714
H	6.558889	-0.529767	0.614601
C	4.088131	-2.937427	0.855083
H	4.369149	-3.413306	1.799205
H	4.499347	-3.529405	0.034590
H	3.001859	-2.945308	0.763169
C	5.038668	-0.349712	3.070060
H	5.226777	-1.298441	3.581560
H	4.590032	0.342542	3.785961
H	5.990896	0.070116	2.746574
C	2.704413	-0.934501	2.405482
H	2.343447	-0.138113	3.060134
H	2.718447	-1.869245	2.971923
H	2.004106	-1.034640	1.571974
C	4.552454	1.630885	-2.326888
H	4.715715	0.667858	-2.825652
H	5.504589	1.893557	-1.845376
C	4.219539	2.694700	-3.369802
H	5.024837	2.805018	-4.102242
H	4.062299	3.668618	-2.893424
H	3.305856	2.439648	-3.912698
H	3.301472	2.416951	-0.759222
H	0.670271	-3.374960	1.146615

H	-1.111156	-3.497647	1.201253
H	-0.250704	-2.294474	2.198148
C	-2.841883	3.826670	-0.393356
H	-3.706572	3.204493	-0.159774
H	-3.111379	4.507676	-1.208419
H	-2.602535	4.444734	0.475412
C	-0.402574	3.886384	-0.991375
H	-0.621018	4.650276	-1.745327
H	0.468773	3.314819	-1.319441
H	-0.142233	4.403485	-0.065899
C	-1.925876	2.318268	-2.183210
H	-1.099950	1.670348	-2.493109
H	-2.039265	3.097157	-2.944051
H	-2.847958	1.733165	-2.162780
C	-5.380105	-2.610253	0.050992
C	-6.691664	-2.224643	0.082477
C	-6.073564	0.113207	0.168944
C	-7.040659	-0.852927	0.140129
N	-3.059577	-2.009454	0.019362
N	-3.748607	0.696548	0.196446
H	-5.086235	-3.651917	0.002285
H	-7.476443	-2.971714	0.061977
H	-8.087455	-0.573084	0.164066
H	-6.313151	1.168620	0.219294

Zero-point correction= 0.809845 (Hartree/Particle)
Thermal correction to Energy= 0.856385
Thermal correction to Enthalpy= 0.857330
Thermal correction to Gibbs Free Energy= 0.732167
Sum of electronic and zero-point Energies= -2377.987577
Sum of electronic and thermal Energies= -2377.941036
Sum of electronic and thermal Enthalpies= -2377.940092
Sum of electronic and thermal Free Energies= -2378.065255

[(*S*)-Quinox-*t*Bu$_3$]: III-C$_{branch}$

C	4.545992	-1.255288	0.179734
C	4.471933	-0.118888	1.018655
C	2.370300	0.337863	0.238485
C	2.426100	-0.849351	-0.573141
P	0.860771	1.424157	0.137832
P	0.938157	-1.320712	-1.585626
Cu	-0.656335	0.368123	-1.233993
B	-3.950564	0.154296	-0.305637
O	-4.212738	0.570459	0.983860
O	-4.022076	-1.220454	-0.409625
C	-4.275108	-0.589838	1.831193
C	-4.575946	-1.739476	0.806584

C	-2.363410	0.482492	-2.215687
H	-2.174328	1.029116	-3.152613
H	-2.619549	-0.535357	-2.539502
C	-3.601113	1.086391	-1.507102
C	1.745567	-1.806417	-3.159429
H	2.628447	-2.422777	-2.984667
H	2.032700	-0.900179	-3.696109
H	1.024654	-2.348332	-3.774651
C	0.381159	-2.945638	-0.815093
C	0.504047	1.889006	1.931910
C	1.521887	2.884875	-0.872154
C	-0.964287	-3.286437	-1.476283
H	-1.344499	-4.224640	-1.056773
H	-0.860262	-3.429060	-2.556249
H	-1.712759	-2.507254	-1.307168
C	0.153509	-2.688677	0.678305
H	-0.535729	-1.853385	0.826505
H	1.085948	-2.466978	1.205380
H	-0.291577	-3.575210	1.142069
C	1.374984	-4.092755	-1.007642
H	0.992829	-4.988685	-0.505921
H	2.355884	-3.855944	-0.591141
H	1.504339	-4.338973	-2.064602
C	0.550422	0.581588	2.739728
H	1.573018	0.223950	2.872171
H	-0.036580	-0.209785	2.269029
H	0.123290	0.761158	3.731646
C	1.459064	2.903994	2.566562
H	2.496374	2.574940	2.495899
H	1.202527	3.015917	3.626167
H	1.364658	3.889938	2.106717
C	-0.933594	2.432706	1.968454
H	-1.211430	2.630704	3.009741
H	-1.652093	1.724066	1.551973
H	-1.035753	3.369137	1.417090
C	2.883948	3.443995	-0.449610
H	3.672084	2.692457	-0.512274
H	2.881154	3.842639	0.563539
H	3.148435	4.261457	-1.129889
C	0.462419	3.995126	-0.840904
H	0.722922	4.759424	-1.580437
H	0.415314	4.485092	0.134229
H	-0.530783	3.612062	-1.087670
C	1.638658	2.356139	-2.313499
H	0.677770	1.994484	-2.693402
H	2.371675	1.546332	-2.389450

H	1.977721	3.166724	-2.967132
H	-4.468951	1.063124	-2.193818
C	-3.413284	2.547968	-1.078848
H	-4.303413	2.893857	-0.540247
H	-2.584239	2.598794	-0.364213
C	-2.906040	-0.729198	2.491804
H	-2.862537	-1.583646	3.171453
H	-2.688652	0.178763	3.057703
H	-2.132262	-0.842167	1.729899
C	-5.346182	-0.377345	2.889370
H	-5.052279	0.443455	3.547155
H	-5.469141	-1.278141	3.498058
H	-6.305843	-0.124084	2.439339
C	-6.070835	-1.950845	0.566654
H	-6.559564	-2.402029	1.433962
H	-6.196623	-2.615684	-0.290148
H	-6.567729	-1.005660	0.336517
C	-3.905436	-3.068833	1.120391
H	-4.162217	-3.796296	0.347231
H	-4.245500	-3.458538	2.084646
H	-2.820021	-2.969874	1.142313
C	-3.128751	3.498285	-2.239223
H	-3.044753	4.534633	-1.896050
H	-3.928778	3.455406	-2.985981
H	-2.194719	3.236272	-2.744347
C	5.568240	0.207360	1.852278
C	6.689592	-0.575141	1.834349
C	5.719901	-2.045627	0.175701
C	6.766532	-1.708784	0.988574
H	5.486298	1.083098	2.484876
H	7.532283	-0.328485	2.469726
H	7.667281	-2.311604	0.990396
H	5.751551	-2.908225	-0.479113
N	3.370583	0.673898	1.017240
N	3.498406	-1.602507	-0.609461

Zero-point correction= 0.810680 (Hartree/Particle)
Thermal correction to Energy= 0.856711
Thermal correction to Enthalpy= 0.857655
Thermal correction to Gibbs Free Energy= 0.735245
Sum of electronic and zero-point Energies= -2377.989636
Sum of electronic and thermal Energies= -2377.943605
Sum of electronic and thermal Enthalpies= -2377.942660
Sum of electronic and thermal Free Energies= -2378.065071

[(*S*)-Quinox-*t*Bu$_3$]: III-D$_{branch}$

C	4.514537	0.154002	-0.882400
C	4.417855	-1.246894	-0.719284
C	2.246912	-1.026672	-0.032843
C	2.337412	0.398348	-0.216568
P	0.634257	-1.781814	0.509099
P	0.804918	1.437774	-0.000607
Cu	-0.793240	0.030624	0.891087
B	-4.041162	0.109644	0.298310
O	-3.499106	0.402429	-0.942230
O	-4.911958	-0.953677	0.220253
C	-2.373430	0.044126	2.096846
H	-2.694088	-0.988600	2.300566
H	-2.068060	0.430646	3.081787
C	-3.628034	0.831532	1.612879
H	-4.441285	0.739044	2.350044
C	-3.361907	2.327471	1.396358
H	-4.259791	2.807625	0.986307
H	-2.580923	2.436310	0.635067
C	1.335927	2.835011	1.154353
C	0.516759	2.025110	-1.775070
C	0.506929	-3.141764	-0.719574
C	1.052832	-2.646202	2.126535
C	-0.929698	2.537832	-1.862651
H	-1.143995	2.814480	-2.901403
H	-1.091172	3.425109	-1.248042
H	-1.654075	1.779919	-1.552886
C	0.635575	0.776281	-2.666507
H	0.050258	-0.058466	-2.270801
H	1.671601	0.448812	-2.780068
H	0.247693	1.009926	-3.663077
C	1.493565	3.090926	-2.277246
H	1.311152	3.268045	-3.343280
H	2.531394	2.776033	-2.152689
H	1.352665	4.042513	-1.760482
C	2.088671	-3.764711	1.994486
H	3.030841	-3.401646	1.581905
H	1.727593	-4.573470	1.353681
H	2.282772	-4.192531	2.984254
C	-0.273613	-3.218181	2.650408
H	-0.104208	-3.692675	3.622791
H	-0.678012	-3.983056	1.980093
H	-1.025848	-2.435463	2.771691
C	1.553634	-1.568736	3.096802
H	0.819849	-0.764981	3.209197
H	2.501680	-1.134229	2.764598

H	1.719009	-2.013356	4.083510
C	-2.949373	3.065980	2.667168
H	-3.711093	2.952464	3.445450
H	-2.813199	4.136742	2.484346
H	-2.009811	2.673776	3.065993
C	2.707098	3.476157	0.915421
H	3.518524	2.759821	1.038685
H	2.842174	4.275358	1.653299
H	2.800866	3.916696	-0.076552
C	0.246535	3.914194	1.098377
H	0.399248	4.620383	1.921132
H	-0.751239	3.486867	1.208288
H	0.285121	4.482213	0.166035
C	1.329283	2.196622	2.554411
H	0.348162	1.781817	2.801555
H	1.581026	2.958210	3.299866
H	2.072102	1.396839	2.634513
H	0.225393	-2.715556	-1.684360
H	1.453131	-3.675055	-0.822010
H	-0.281751	-3.831225	-0.412190
C	-3.799649	-0.695888	-1.817364
C	-5.095292	-1.284963	-1.166679
C	-2.616984	-1.661654	-1.747883
H	-1.712426	-1.118398	-2.027801
H	-2.740824	-2.505927	-2.430853
H	-2.477443	-2.037410	-0.731123
C	-3.964684	-0.166910	-3.232622
H	-4.274446	-0.966296	-3.912281
H	-3.010860	0.232867	-3.585919
H	-4.702761	0.634078	-3.272727
C	-6.368103	-0.580292	-1.635277
H	-7.196284	-0.891022	-0.995323
H	-6.611390	-0.833782	-2.670346
H	-6.265436	0.504269	-1.554323
C	-5.250054	-2.791806	-1.300142
H	-5.296539	-3.083192	-2.353861
H	-6.176755	-3.107697	-0.816497
H	-4.423951	-3.319301	-0.823299
C	5.731115	0.723790	-1.328373
C	6.800054	-0.085901	-1.595813
C	5.537397	-2.063788	-1.006747
C	6.702360	-1.489813	-1.434049
H	5.778849	1.799599	-1.447404
H	7.733720	0.345144	-1.937952
H	7.563390	-2.110099	-1.654450
H	5.434644	-3.134201	-0.875051

N	3.448199	0.955345	-0.636514
N	3.260905	-1.814337	-0.296909

Zero-point correction= 0.811520 (Hartree/Particle)
Thermal correction to Energy= 0.857277
Thermal correction to Enthalpy= 0.858221
Thermal correction to Gibbs Free Energy= 0.736196
Sum of electronic and zero-point Energies= -2377.989589
Sum of electronic and thermal Energies= -2377.943832
Sum of electronic and thermal Enthalpies= -2377.942888
Sum of electronic and thermal Free Energies= -2378.064914

[(*S*)-Quinox-*t*Bu$_3$]: III-D$_{branch,rot}$

C	-5.080410	-0.805442	0.513741
C	-5.324117	0.491437	0.004206
C	-3.081344	0.800209	-0.310215
C	-2.839106	-0.547067	0.134962
P	-1.638477	1.889933	-0.765789
P	-1.101810	-1.201008	0.053077
Cu	0.254515	0.636715	-0.396504
B	4.466409	-0.203416	-0.172540
O	5.440695	0.484007	-0.867158
O	4.907418	-0.582433	1.078692
C	2.204142	0.803291	-0.149293
H	2.622545	1.715357	-0.600905
H	2.388841	0.906685	0.932763
C	3.013086	-0.419764	-0.684374
H	2.608552	-1.334562	-0.229038
C	2.905541	-0.546537	-2.204164
H	3.297237	0.366997	-2.665510
H	1.842070	-0.589770	-2.479123
C	-0.846554	-2.110187	1.682113
C	-1.209320	-2.361922	-1.435935
C	-2.260967	2.610074	-2.333666
C	-1.730927	3.291229	0.486376
C	0.107270	-3.146904	-1.511828
H	0.169254	-3.655551	-2.479497
H	0.165430	-3.911182	-0.733639
H	0.977070	-2.491330	-1.421720
C	-1.301813	-1.427604	-2.656293
H	-0.453548	-0.739057	-2.705923
H	-2.225985	-0.840140	-2.646245
H	-1.305222	-2.027901	-3.572099
C	-2.399235	-3.325975	-1.468578
H	-2.351090	-3.906726	-2.396844
H	-3.354127	-2.798711	-1.454208
H	-2.393534	-4.028848	-0.636520

C	-3.023114	4.108539	0.433323
H	-3.905257	3.493040	0.613034
H	-3.147643	4.602971	-0.533707
H	-2.984047	4.889883	1.200204
C	-0.526953	4.199212	0.189145
H	-0.483280	4.996593	0.938556
H	-0.611272	4.676209	-0.791818
H	0.412877	3.641856	0.219885
C	-1.553274	2.660822	1.873929
H	-0.615732	2.098643	1.933160
H	-2.378714	1.988788	2.127733
H	-1.519823	3.448383	2.633602
C	3.637544	-1.759157	-2.774882
H	4.713919	-1.682398	-2.593177
H	3.488502	-1.852465	-3.854727
H	3.285837	-2.686019	-2.307810
C	-1.589776	-3.439055	1.835620
H	-2.660941	-3.318966	1.667614
H	-1.436681	-3.819181	2.852026
H	-1.205263	-4.194607	1.146890
C	0.669267	-2.330263	1.828659
H	0.870111	-2.777277	2.808124
H	1.220326	-1.389484	1.757663
H	1.066431	-3.006719	1.069789
C	-1.292807	-1.136158	2.784911
H	-0.815419	-0.158568	2.671839
H	-0.992810	-1.540530	3.756987
H	-2.376096	-1.000039	2.798265
H	-2.193260	1.850261	-3.114488
H	-3.296595	2.938449	-2.235476
H	-1.624469	3.448458	-2.621812
C	6.473010	0.833940	0.070228
C	6.307068	-0.270219	1.168903
C	6.148255	2.236824	0.584217
H	6.096323	2.918185	-0.267455
H	6.911077	2.600948	1.277287
H	5.177458	2.251559	1.085535
C	7.814929	0.831868	-0.644113
H	8.628928	1.013704	0.064168
H	7.829832	1.624884	-1.394759
H	7.994121	-0.116902	-1.150010
C	7.075148	-1.552208	0.846061
H	6.747190	-2.337043	1.530787
H	8.153535	-1.416513	0.962099
H	6.870004	-1.884263	-0.174324
C	6.621986	0.186130	2.585016

H	7.658952	0.527084	2.661049
H	6.484344	-0.647057	3.277826
H	5.961270	0.995835	2.894747
C	-6.166201	-1.594265	0.963862
C	-7.438756	-1.098873	0.892761
C	-6.651360	0.977404	-0.066992
C	-7.683518	0.194558	0.370074
H	-5.953518	-2.582822	1.352747
H	-8.273556	-1.699465	1.234884
H	-8.701452	0.563307	0.319865
H	-6.809890	1.971370	-0.467764
N	-3.821727	-1.311478	0.548957
N	-4.297208	1.281037	-0.398592

Zero-point correction= 0.810432 (Hartree/Particle)
Thermal correction to Energy= 0.856870
Thermal correction to Enthalpy= 0.857814
Thermal correction to Gibbs Free Energy= 0.731999
Sum of electronic and zero-point Energies= -2377.984984
Sum of electronic and thermal Energies= -2377.938547
Sum of electronic and thermal Enthalpies= -2377.937603
Sum of electronic and thermal Free Energies= -2378.063417

[(*S*)-Quinox-*t*Bu$_3$]: III-A$_{linear}$

C	-4.476325	-1.200987	-0.037339
C	-4.403669	-0.214561	-1.049308
C	-2.298339	0.355175	-0.364513
C	-2.345974	-0.696180	0.617801
P	-0.794041	1.442106	-0.403125
P	-0.826672	-1.061843	1.636985
Cu	0.739251	0.522999	1.076144
B	3.887435	-0.280073	0.379439
O	4.066665	-1.634164	0.177082
O	3.978491	0.416501	-0.810872
C	2.428077	1.358068	1.703447
H	2.688837	2.142093	0.973424
C	3.630907	0.386704	1.758849
H	4.563100	0.898197	2.060444
C	-1.545805	-1.054533	3.324017
H	-2.477616	-1.620395	3.369072
H	-1.730278	-0.018634	3.614250
H	-0.816847	-1.474306	4.020051
C	-0.482771	-2.870910	1.251990
C	0.952192	-3.131523	1.736536
H	1.211832	-4.180227	1.553745
H	1.055209	-2.949243	2.810823
H	1.683168	-2.501200	1.224186

C	-0.558008	-3.048003	-0.269899
H	0.014007	-2.279647	-0.794568
H	-1.589607	-3.013181	-0.628270
H	-0.137024	-4.019847	-0.546942
C	-1.452806	-3.839317	1.934126
H	-1.226577	-4.861698	1.611574
H	-2.490109	-3.617492	1.676103
H	-1.351465	-3.810540	3.022062
C	4.099668	-1.878963	-1.239381
C	4.492015	-0.474738	-1.812721
C	2.691394	-2.304544	-1.643458
H	2.407503	-3.188589	-1.069363
H	2.619834	-2.539872	-2.708096
H	1.981518	-1.507519	-1.410156
C	5.091949	-2.993675	-1.528455
H	5.205568	-3.140950	-2.606590
H	4.730011	-3.926860	-1.091243
H	6.069483	-2.776434	-1.098334
C	3.862080	-0.136184	-3.155380
H	4.187024	-0.846135	-3.921784
H	4.172273	0.865553	-3.461230
H	2.773518	-0.148815	-3.103105
C	6.003900	-0.256471	-1.875955
H	6.195588	0.797751	-2.085376
H	6.466513	-0.858967	-2.661799
H	6.476130	-0.502180	-0.922037
C	-1.459475	3.024465	0.387265
C	-0.355584	1.644650	-2.220145
C	-0.342696	0.223200	-2.806281
H	-1.348051	-0.193522	-2.895356
H	0.262318	-0.453993	-2.197108
H	0.102296	0.252669	-3.806375
C	-0.340832	4.074049	0.322931
H	-0.608716	4.925094	0.958072
H	-0.204120	4.454025	-0.692437
H	0.611516	3.669358	0.678096
C	-1.282365	2.531871	-3.054154
H	-2.324498	2.221505	-2.962915
H	-0.990165	2.462329	-4.108240
H	-1.196933	3.581645	-2.764617
C	1.082151	2.193483	-2.256965
H	1.143698	3.211774	-1.869125
H	1.428525	2.214077	-3.296421
H	1.773674	1.578920	-1.674431
C	-2.755366	3.591899	-0.199538
H	-2.637749	3.918559	-1.231775

H	-3.055681	4.463658	0.392919
H	-3.570135	2.866965	-0.167905
C	-1.709010	2.660212	1.862524
H	-0.802356	2.284310	2.345828
H	-2.500073	1.910842	1.968019
H	-2.032022	3.555726	2.403393
C	2.211272	2.065099	3.053247
H	1.971832	1.319122	3.824299
H	1.328568	2.716775	2.987963
C	3.383776	2.924647	3.543241
H	3.651656	3.671628	2.788000
H	4.274557	2.321665	3.740001
H	3.136354	3.454437	4.469578
H	3.458482	-0.394344	2.510201
C	-5.654684	-1.972957	0.100908
C	-6.704783	-1.765943	-0.750190
C	-5.502780	-0.023205	-1.920128
C	-6.627380	-0.785771	-1.769696
N	-3.303657	0.569261	-1.179517
N	-3.421666	-1.425608	0.787226
H	-5.686772	-2.719682	0.885149
H	-7.608699	-2.355276	-0.648977
H	-7.472283	-0.642124	-2.433135
H	-5.419944	0.737816	-2.686723

Zero-point correction= 0.810217 (Hartree/Particle)
Thermal correction to Energy= 0.856578
Thermal correction to Enthalpy= 0.857522
Thermal correction to Gibbs Free Energy= 0.733008
Sum of electronic and zero-point Energies= -2377.985577
Sum of electronic and thermal Energies= -2377.939217
Sum of electronic and thermal Enthalpies= -2377.938273
Sum of electronic and thermal Free Energies= -2378.062786

[(*S*)-Quinox-*t*Bu$_3$]: III-B$_{linear}$

C	4.533440	0.019467	-0.608256
C	4.258440	-1.355149	-0.789338
C	2.072281	-1.002628	-0.216084
C	2.341894	0.404514	-0.065811
P	0.340077	-1.651110	0.028601
P	0.918757	1.547353	0.295932
Cu	-0.860779	0.154462	0.795141
B	-3.741126	0.818810	-0.047881
O	-4.741078	-0.117744	-0.185618
O	-3.016620	0.950134	-1.223602
C	-4.491632	-0.854038	-1.394625
C	-3.667071	0.173099	-2.241918

C	-2.371042	0.600239	2.022642
H	-1.942289	1.195620	2.847021
C	-3.363452	1.545899	1.268925
C	1.489860	2.551658	1.785730
C	0.800166	2.539274	-1.305461
C	0.176092	-2.606531	-1.532466
C	0.498036	-2.958116	1.371161
C	-0.539475	3.292915	-1.288770
H	-0.657199	3.820227	-2.242499
H	-0.583390	4.040244	-0.494849
H	-1.382984	2.608911	-1.172681
C	0.736267	1.492432	-2.432088
H	-0.046230	0.752205	-2.240795
H	1.690011	0.976696	-2.572215
H	0.491551	1.997190	-3.372444
C	1.951740	3.512050	-1.568063
H	1.870675	3.896043	-2.591375
H	2.926605	3.034149	-1.453001
H	1.903746	4.370929	-0.895540
C	1.497155	-4.078691	1.075734
H	2.510348	-3.699964	0.941361
H	1.224736	-4.639177	0.177562
H	1.497399	-4.783764	1.914226
C	-0.909514	-3.552996	1.537391
H	-0.904239	-4.264068	2.370097
H	-1.226419	-4.099439	0.643663
H	-1.651520	-2.781299	1.752681
C	0.894108	-2.219875	2.656491
H	0.171737	-1.435256	2.900758
H	1.885375	-1.764118	2.569632
H	0.923313	-2.927180	3.491583
C	-5.817987	-1.272075	-2.007329
H	-6.319256	-1.978951	-1.342999
H	-5.659785	-1.761014	-2.973301
H	-6.478048	-0.416430	-2.149847
C	-3.676439	-2.087811	-1.001167
H	-3.473363	-2.730280	-1.862455
H	-4.241857	-2.661520	-0.263926
H	-2.729604	-1.793225	-0.540084
C	-4.546819	1.129336	-3.046012
H	-5.029828	0.623296	-3.885765
H	-3.922281	1.935207	-3.437592
H	-5.318529	1.573948	-2.413827
C	-2.607728	-0.445614	-3.138382
H	-2.085280	0.340525	-3.688719
H	-3.060052	-1.129567	-3.862374

H	-1.875686	-0.993973	-2.546714
H	-2.845993	2.482065	1.029696
H	-0.774818	-3.142066	-1.534771
H	1.004786	-3.304776	-1.656094
H	0.178631	-1.905972	-2.369818
C	2.837901	3.272668	1.689445
H	3.648109	2.588169	1.440218
H	3.055348	3.732466	2.660256
H	2.829221	4.067993	0.944638
C	0.376589	3.562243	2.097722
H	0.557646	4.002596	3.083898
H	-0.607676	3.085857	2.113495
H	0.362418	4.379457	1.372810
C	1.552886	1.522002	2.928538
H	0.590951	1.021169	3.067594
H	1.807571	2.036014	3.861185
H	2.320694	0.762982	2.749190
C	-3.080691	-0.591473	2.685182
H	-2.329787	-1.170675	3.238876
H	-3.479453	-1.271803	1.921753
C	-4.216596	-0.241651	3.654031
H	-5.072241	0.193006	3.130570
H	-3.877676	0.485234	4.400869
H	-4.576456	-1.129085	4.185587
H	-4.243931	1.809830	1.870945
C	5.847410	0.503187	-0.814841
C	6.836043	-0.363726	-1.189948
C	5.297650	-2.231143	-1.183820
C	6.559021	-1.739998	-1.377507
N	3.007833	-1.840734	-0.589107
N	3.547425	0.883935	-0.259626
H	5.059601	-3.279220	-1.320105
H	7.359122	-2.406553	-1.677831
H	7.844100	0.001111	-1.349282
H	6.031484	1.560778	-0.668713

Zero-point correction= 0.811237 (Hartree/Particle)
Thermal correction to Energy= 0.857206
Thermal correction to Enthalpy= 0.858150
Thermal correction to Gibbs Free Energy= 0.735505
Sum of electronic and zero-point Energies= -2377.988585
Sum of electronic and thermal Energies= -2377.942616
Sum of electronic and thermal Enthalpies= -2377.941672
Sum of electronic and thermal Free Energies= -2378.064317

[(*S*)-Quinox-*t*Bu$_3$]: III-C$_{linear}$

C	4.503280	-1.214506	0.154059
C	4.427980	-0.110012	1.034861
C	2.348755	0.401947	0.232716
C	2.392638	-0.768220	-0.602566
P	0.871446	1.518737	0.104986
P	0.888592	-1.233172	-1.605358
Cu	-0.635460	0.485073	-1.319006
B	-3.754285	-0.563227	-0.557682
O	-2.836271	-0.945965	0.411836
O	-5.029734	-0.536501	-0.042778
C	-3.577486	-1.446542	1.540968
C	-4.946815	-0.708403	1.381599
C	-2.397144	0.995792	-2.087241
H	-2.239930	1.219497	-3.155053
C	-3.390006	-0.200585	-2.027166
C	1.683245	-1.631900	-3.210419
H	2.594118	-2.216341	-3.072708
H	1.922067	-0.695775	-3.718630
H	0.974791	-2.181768	-3.832877
C	0.405627	-2.904385	-0.881824
C	0.392810	1.875568	1.892032
C	1.614639	3.017774	-0.776005
C	-0.918031	-3.294181	-1.556139
H	-1.251944	-4.261020	-1.163244
H	-0.811458	-3.399380	-2.640044
H	-1.694926	-2.556044	-1.350925
C	0.152536	-2.693302	0.615569
H	-0.616856	-1.932148	0.768196
H	1.061957	-2.402674	1.149867
H	-0.206277	-3.628762	1.059110
C	1.448445	-4.002189	-1.102086
H	1.096649	-4.927940	-0.633410
H	2.414827	-3.740775	-0.669122
H	1.597225	-4.208464	-2.165276
C	0.412781	0.525702	2.624960
H	1.430018	0.166645	2.790792
H	-0.144854	-0.233509	2.072602
H	-0.068285	0.642903	3.601792
C	1.275559	2.874915	2.641998
H	2.321734	2.564187	2.631605
H	0.942107	2.933806	3.684405
H	1.199659	3.879336	2.219883
C	-1.063845	2.367719	1.844824
H	-1.424937	2.528264	2.867170
H	-1.705310	1.628229	1.358211

H	-1.171870	3.307843	1.299995
C	2.942383	3.556517	-0.233925
H	3.732083	2.805183	-0.260334
H	2.860748	3.921402	0.789094
H	3.257666	4.396610	-0.863311
C	0.559295	4.131690	-0.771657
H	0.884327	4.936928	-1.438811
H	0.425718	4.562931	0.223259
H	-0.407907	3.767827	-1.125809
C	1.829032	2.547603	-2.226617
H	0.895687	2.197340	-2.678638
H	2.569240	1.742899	-2.285700
H	2.205412	3.383808	-2.825233
H	-2.923723	-1.072768	-2.502511
C	-3.692473	-2.960966	1.364445
H	-4.192206	-3.428655	2.216312
H	-2.689349	-3.383104	1.275944
H	-4.244466	-3.208813	0.454716
C	-2.831313	-1.126392	2.825217
H	-1.897073	-1.691399	2.864873
H	-3.433582	-1.408203	3.694066
H	-2.591703	-0.065621	2.895286
C	-4.945976	0.688496	2.003509
H	-4.962186	0.642960	3.095504
H	-5.835293	1.222181	1.662959
H	-4.070278	1.259948	1.687148
C	-6.156128	-1.495855	1.859343
H	-7.061688	-0.906230	1.702512
H	-6.072041	-1.717703	2.927531
H	-6.263626	-2.432753	1.312818
C	-3.023590	2.254846	-1.463042
H	-2.322755	3.091155	-1.573155
H	-3.140910	2.119494	-0.377223
C	-4.379245	2.690285	-2.034733
H	-5.159651	1.955191	-1.819817
H	-4.318960	2.804838	-3.122690
H	-4.703897	3.647329	-1.611824
H	-4.319130	-0.011785	-2.584994
C	5.670716	-2.014612	0.135016
C	6.706863	-1.722008	0.977841
C	5.512488	0.168963	1.900640
C	6.625728	-0.624455	1.869869
H	7.601743	-2.333466	0.969215
H	7.458993	-0.414120	2.530249
H	5.428282	1.018457	2.567795
H	5.704870	-2.850140	-0.553890

N	3.339523	0.700076	1.039323
N	3.460180	-1.526988	-0.655214

Zero-point correction= 0.810059 (Hartree/Particle)
Thermal correction to Energy= 0.856406
Thermal correction to Enthalpy= 0.857351
Thermal correction to Gibbs Free Energy= 0.733295
Sum of electronic and zero-point Energies= -2377.990353
Sum of electronic and thermal Energies= -2377.944006
Sum of electronic and thermal Enthalpies= -2377.943061
Sum of electronic and thermal Free Energies= -2378.067117

[(*S*)-Quinox-*t*Bu$_3$]: III-D$_{linear}$

C	4.520863	-0.101564	-0.783777
C	4.293754	-1.483260	-0.588031
C	2.116101	-1.058217	-0.033523
C	2.339516	0.347417	-0.256769
P	0.420287	-1.654712	0.459134
P	0.888672	1.507144	-0.142148
Cu	-0.837725	0.276002	0.773002
B	-3.937323	0.470427	-0.042361
O	-4.507252	-0.773477	0.115697
O	-3.715323	0.753420	-1.377512
C	-2.409210	0.665060	1.934162
H	-2.831932	-0.293324	2.274138
C	-3.543132	1.383089	1.150523
H	-4.426944	1.573176	1.780326
C	1.480268	2.919050	0.956637
C	0.656511	2.007670	-1.948045
C	0.215368	-3.032068	-0.739030
C	0.691404	-2.496072	2.119158
C	-0.773827	2.555427	-2.083023
H	-0.962412	2.804520	-3.133580
H	-0.920916	3.465658	-1.498265
H	-1.528801	1.832372	-1.764455
C	0.767426	0.715380	-2.775573
H	0.170921	-0.094610	-2.347514
H	1.801887	0.375229	-2.863350
H	0.390715	0.904746	-3.786057
C	1.665319	3.026901	-2.480728
H	1.524194	3.137693	-3.561844
H	2.695010	2.711536	-2.298456
H	1.513964	4.010612	-2.031557
C	1.600485	-3.725637	2.062395
H	2.583432	-3.488284	1.653325
H	1.162514	-4.521512	1.454069
H	1.731518	-4.122881	3.074970

C	-0.705963	-2.898609	2.615887
H	-0.617837	-3.360385	3.604955
H	-1.177100	-3.631367	1.953284
H	-1.368049	-2.032848	2.691500
C	1.278423	-1.442555	3.067083
H	0.643979	-0.552455	3.112719
H	2.284831	-1.138946	2.763311
H	1.346820	-1.857968	4.077598
C	2.803665	3.607037	0.605170
H	3.629072	2.897447	0.562300
H	3.022535	4.348606	1.381816
H	2.761494	4.131887	-0.348154
C	0.344346	3.952308	0.986289
H	0.532001	4.673248	1.788711
H	-0.624808	3.480654	1.171169
H	0.286429	4.511302	0.048604
C	1.614775	2.280408	2.350118
H	0.676523	1.827815	2.677859
H	1.888481	3.053069	3.075872
H	2.398010	1.516426	2.368774
H	0.034704	-2.609454	-1.729459
H	1.101913	-3.666987	-0.773048
H	-0.659564	-3.622126	-0.458340
C	-4.847937	-1.291556	-1.177020
C	-3.928502	-0.451380	-2.131942
C	-6.337918	-1.024393	-1.389717
H	-6.893840	-1.483051	-0.569636
H	-6.695502	-1.445506	-2.332839
H	-6.547708	0.047582	-1.381032
C	-4.578266	-2.788994	-1.189502
H	-4.741706	-3.207076	-2.187358
H	-5.257298	-3.286619	-0.494025
H	-3.556325	-3.006663	-0.877349
C	-2.555383	-1.085604	-2.347605
H	-1.933976	-0.386038	-2.909280
H	-2.620077	-2.019530	-2.911555
H	-2.069031	-1.281883	-1.387738
C	-4.551767	-0.095516	-3.472604
H	-4.783205	-1.001241	-4.041131
H	-3.849287	0.504814	-4.055112
H	-5.466989	0.482385	-3.346482
C	-2.016753	1.436693	3.204737
H	-1.613281	2.423215	2.935994
H	-1.194020	0.908617	3.708247
C	-3.138548	1.633667	4.231483
H	-3.580808	0.669686	4.504841

H	-3.940673	2.264229	3.838267
H	-2.766853	2.107379	5.146377
H	-3.196012	2.357011	0.782664
C	5.806263	0.347795	-1.170090
C	6.814463	-0.558448	-1.349212
C	5.352553	-2.401438	-0.784090
C	6.585997	-1.942608	-1.155368
H	5.954141	1.411166	-1.315345
H	7.801014	-0.219707	-1.643723
H	7.400851	-2.641503	-1.304483
H	5.151503	-3.454568	-0.628704
N	3.515484	0.796153	-0.624854
N	3.070461	-1.937246	-0.215900

Zero-point correction= 0.810662 (Hartree/Particle)
Thermal correction to Energy= 0.856832
Thermal correction to Enthalpy= 0.857776
Thermal correction to Gibbs Free Energy= 0.734485
Sum of electronic and zero-point Energies= -2377.986855
Sum of electronic and thermal Energies= -2377.940685
Sum of electronic and thermal Enthalpies= -2377.939740
Sum of electronic and thermal Free Energies= -2378.063032

[(*S*)-Quinox-*t*Bu$_3$]: TS$_{pro}$-D$_{branch}$

C	5.012828	-0.237240	-1.080833
C	5.103705	1.084612	-0.588893
C	2.932066	0.975366	0.125176
C	2.845622	-0.382558	-0.352011
P	1.479798	1.764187	0.981917
P	1.308257	-1.385427	-0.026796
Cu	-0.029113	0.099602	1.032210
B	-4.308047	-0.260996	-0.310001
O	-5.503280	-0.315168	0.369664
O	-4.408523	0.486196	-1.464019
C	-2.227693	0.265191	0.961438
H	-2.916521	0.784832	1.645150
H	-1.955522	1.047563	0.233323
C	-2.971976	-0.873867	0.210138
H	-2.365144	-1.145934	-0.662697
C	-3.143522	-2.125214	1.075980
H	-3.703441	-1.863423	1.980451
H	-2.157218	-2.453929	1.422523
C	0.823703	-2.078227	-1.710579
C	1.939616	-2.694432	1.178098
C	2.306672	2.441012	2.470966
C	1.057335	3.260103	-0.072147
C	0.716732	-3.383263	1.808260

H	1.072349	-4.104483	2.552907
H	0.124620	-3.936376	1.077601
H	0.074726	-2.656951	2.314787
C	2.653803	-1.916775	2.299064
H	1.985743	-1.167893	2.736200
H	3.572378	-1.434405	1.954190
H	2.926382	-2.619200	3.092998
C	2.886108	-3.731422	0.570409
H	3.285447	-4.361738	1.372664
H	3.723823	-3.266039	0.047410
H	2.360653	-4.388290	-0.126789
C	2.166603	4.312255	-0.139782
H	3.097206	3.896311	-0.527936
H	2.370927	4.745996	0.842475
H	1.848126	5.127299	-0.798934
C	-0.206040	3.871247	0.553908
H	-0.534568	4.722347	-0.051679
H	-0.020131	4.241650	1.566447
H	-1.023332	3.147336	0.600834
C	0.727468	2.745546	-1.478668
H	-0.057087	1.984546	-1.453052
H	1.608273	2.318923	-1.967873
H	0.371306	3.574235	-2.098884
C	-3.850728	-3.268130	0.350631
H	-4.879170	-2.993938	0.096499
H	-3.890323	-4.170861	0.966736
H	-3.333314	-3.523729	-0.581182
C	1.938158	-2.664482	-2.584275
H	2.671206	-1.911473	-2.871101
H	1.482503	-3.063819	-3.497684
H	2.472244	-3.477233	-2.093305
C	-0.252862	-3.147990	-1.473422
H	-0.754233	-3.366833	-2.421736
H	-1.012053	-2.819151	-0.760236
H	0.184526	-4.079445	-1.107038
C	0.195172	-0.885186	-2.450695
H	-0.654539	-0.468937	-1.904133
H	-0.160060	-1.214811	-3.432560
H	0.925373	-0.086464	-2.613132
H	2.543923	1.609246	3.136719
H	3.222175	2.974971	2.212255
H	1.615006	3.104222	2.993653
C	-6.388016	0.652108	-0.223662
C	-5.798931	0.795053	-1.668910
C	-6.261302	1.932961	0.600815
H	-6.488688	1.702104	1.643445

H	-6.952840	2.705534	0.255232
H	-5.242798	2.326703	0.557422
C	-7.812362	0.124947	-0.160098
H	-8.497672	0.802851	-0.677608
H	-8.126838	0.049357	0.882917
H	-7.891343	-0.864670	-0.610087
C	-6.348719	-0.247392	-2.642734
H	-5.757774	-0.217408	-3.560484
H	-7.394114	-0.052267	-2.894594
H	-6.271072	-1.252921	-2.222459
C	-5.915179	2.186058	-2.271891
H	-6.965020	2.478363	-2.368329
H	-5.466001	2.190226	-3.267426
H	-5.397351	2.927666	-1.663695
C	6.131600	-0.827474	-1.715163
C	7.290668	-0.113723	-1.843065
C	6.316214	1.800771	-0.728282
C	7.384117	1.208790	-1.344183
H	6.033115	-1.841345	-2.084191
H	8.151776	-0.559915	-2.326737
H	8.315827	1.751364	-1.454263
H	6.362863	2.809769	-0.336416
N	3.871531	-0.954573	-0.934190
N	4.039614	1.667449	0.018110
O	-0.734625	-0.636153	2.968345
H	-1.471055	-0.246839	2.061510
C	-0.694674	0.305315	3.994615
H	0.334505	0.470837	4.351610
H	-1.083467	1.286884	3.664738
H	-1.294325	-0.008161	4.862056

Zero-point correction= 0.859368 (Hartree/Particle)
Thermal correction to Energy= 0.909474
Thermal correction to Enthalpy= 0.910418
Thermal correction to Gibbs Free Energy= 0.776761
Sum of electronic and zero-point Energies= -2493.652872
Sum of electronic and thermal Energies= -2493.602766
Sum of electronic and thermal Enthalpies= -2493.601822
Sum of electronic and thermal Free Energies= -2493.735479

[(*S*)-Quinox-Ad$_3$]CuB(pin)

P	0.294747	-0.885988	0.192360
P	-1.572091	1.029859	-1.619301
Cu	0.576921	1.199351	-0.833779
C	4.655661	2.366985	-1.065460
C	4.312713	2.702256	0.419199
B	2.391724	2.030249	-0.682310

O	2.880261	2.791040	0.387717
O	3.477021	1.669189	-1.495840
C	0.515615	-1.119790	2.036954
C	-2.665335	2.288720	-0.776815
C	1.326677	-2.067593	-0.844292
C	1.038297	-3.573883	-0.703359
H	-0.011437	-3.781486	-0.928233
H	1.211487	-3.907255	0.320710
C	2.818112	-1.799251	-0.539172
H	3.032323	-0.730354	-0.635694
H	3.046291	-2.100918	0.488407
C	1.052055	-1.656870	-2.314261
H	-0.003713	-1.831648	-2.558427
H	1.254703	-0.586800	-2.446476
C	-2.124893	0.996818	-3.365891
H	-3.166902	0.685125	-3.448201
H	-1.491382	0.297701	-3.915019
H	-1.988839	1.988202	-3.800807
C	-2.423775	2.164280	0.741465
H	-1.350184	2.251163	0.950418
H	-2.749495	1.178351	1.093921
C	-4.170988	2.166256	-1.064027
H	-4.539000	1.181715	-0.764232
H	-4.357433	2.263883	-2.138986
C	-2.176262	3.681879	-1.236457
H	-1.100739	3.777904	-1.054537
H	-2.334742	3.800661	-2.314603
C	1.751964	-0.282621	2.447719
H	2.654257	-0.691215	1.986766
H	1.654882	0.743536	2.078674
C	0.694538	-2.561095	2.544362
H	-0.160352	-3.171976	2.240843
H	1.592534	-3.000376	2.099953
C	-0.723489	-0.493996	2.719146
H	-1.625296	-1.052531	2.451165
H	-0.854044	0.534607	2.368842
C	4.794710	3.613105	-1.941094
H	4.817746	3.304122	-2.988345
H	5.713532	4.164123	-1.720452
H	3.940871	4.279943	-1.804070
C	5.862499	1.459384	-1.256345
H	6.765780	1.918427	-0.842070
H	6.025837	1.286341	-2.322689
H	5.710278	0.491478	-0.776855
C	4.675475	1.566064	1.379300
H	4.205890	1.760058	2.346691

H	5.756023	1.482637	1.524871
H	4.296346	0.611333	1.008152
C	4.885518	4.014722	0.932443
H	5.978853	4.010255	0.876030
H	4.598181	4.156838	1.976955
H	4.507635	4.861880	0.359993
C	3.417612	-4.096138	-1.333478
H	4.063536	-4.684199	-1.995396
H	3.639248	-4.406769	-0.305620
C	1.942742	-4.372706	-1.658529
H	1.731700	-5.439242	-1.530030
C	1.653563	-3.959369	-3.106868
H	2.275696	-4.540912	-3.796650
H	0.607064	-4.171308	-3.355442
C	3.412352	-2.170135	-2.946605
H	3.616763	-1.100877	-3.063335
H	4.064983	-2.713109	-3.639695
C	3.705717	-2.598850	-1.503002
H	4.754383	-2.392932	-1.266151
C	1.941036	-2.461515	-3.271642
H	1.723143	-2.151985	-4.298701
C	-2.944160	4.780293	-0.485977
H	-2.585733	5.756406	-0.826793
C	-2.695507	4.636748	1.021893
H	-1.627108	4.745769	1.237000
H	-3.220129	5.430157	1.566127
C	-4.445329	4.646579	-0.774845
H	-4.999856	5.437369	-0.256948
H	-4.633853	4.769621	-1.847520
C	-4.689785	3.121744	1.200390
H	-5.050730	2.146199	1.546215
H	-5.251647	3.887227	1.747298
C	-3.189518	3.261710	1.491775
H	-3.008378	3.149901	2.565438
C	-4.933956	3.268414	-0.308628
H	-6.003234	3.164811	-0.518608
C	0.852124	-2.562749	4.074335
H	0.975087	-3.595993	4.414732
C	2.090776	-1.741268	4.460374
H	2.230579	-1.759466	5.547267
H	2.987906	-2.183601	4.012007
C	-0.392808	-1.948600	4.727464
H	-1.281945	-2.534206	4.466574
H	-0.295637	-1.973675	5.818755
C	0.678075	0.321338	4.630915
H	0.795660	0.336851	5.720455

H	0.556412	1.358961	4.301254
C	-0.557814	-0.501473	4.244859
H	-1.451985	-0.060761	4.697391
C	1.918381	-0.295695	3.973349
H	2.805626	0.290414	4.233146
C	-1.431519	-1.446887	-0.179651
C	-2.240031	-0.609839	-1.028255
N	-1.890809	-2.581123	0.293205
N	-3.438972	-0.976685	-1.408144
C	-3.148378	-2.951735	-0.059248
C	-3.921741	-2.153516	-0.934226
C	-3.684320	-4.163861	0.436841
C	-4.940792	-4.553159	0.062541
C	-5.712821	-3.756287	-0.817620
C	-5.217455	-2.580478	-1.309892
H	-3.070555	-4.757893	1.103286
H	-5.355593	-5.480751	0.439644
H	-6.705755	-4.086704	-1.099930
H	-5.787736	-1.950287	-1.981772

Zero-point correction= 1.036613 (Hartree/Particle)
Thermal correction to Energy= 1.083314
Thermal correction to Enthalpy= 1.084258
Thermal correction to Gibbs Free Energy= 0.959340
Sum of electronic and zero-point Energies= -2917.315195
Sum of electronic and thermal Energies= -2917.268495
Sum of electronic and thermal Enthalpies= -2917.267551
Sum of electronic and thermal Free Energies= -2917.392469

[(*S*)-Quinox-Ad$_3$]CuOMe]

P	-1.218292	-0.364157	-0.253620
P	1.630241	0.722479	-1.510432
Cu	0.386868	-1.152434	-1.667234
C	-1.510169	-1.515593	1.193615
C	3.317528	0.286225	-0.831386
C	-2.792203	0.346900	-0.985932
C	-3.624598	1.292413	-0.098245
H	-3.021016	2.147549	0.214682
H	-3.943736	0.785056	0.813747
C	-3.675010	-0.837432	-1.436233
H	-3.089061	-1.526678	-2.054837
H	-4.020045	-1.397660	-0.562476
C	-2.348914	1.131776	-2.246112
H	-1.715516	1.978774	-1.952168
H	-1.745404	0.485195	-2.893274
C	1.922795	2.100715	-2.685187
H	2.337285	2.970915	-2.173945

H	0.972856	2.369673	-3.150725
H	2.604596	1.766350	-3.468852
C	3.103358	-0.685513	0.348499
H	2.494585	-1.535415	0.016730
H	2.557481	-0.183791	1.156821
C	4.183694	1.471219	-0.372920
H	3.672427	2.035837	0.409903
H	4.350526	2.161142	-1.207529
C	4.060711	-0.474855	-1.953964
H	3.452509	-1.316800	-2.299425
H	4.228210	0.185498	-2.813239
C	-1.743958	-2.927687	0.595881
H	-2.673743	-2.945612	0.019822
H	-0.932381	-3.184665	-0.095599
C	-2.657495	-1.163277	2.156073
H	-2.503674	-0.163318	2.572377
H	-3.610902	-1.155331	1.618626
C	-0.182201	-1.558839	1.988334
H	0.030698	-0.578963	2.426426
H	0.639217	-1.811367	1.310340
C	-5.719425	0.598364	-1.304498
H	-6.611534	0.951088	-1.834044
H	-6.064353	0.047166	-0.422083
C	-4.855809	1.791697	-0.874190
H	-5.437948	2.449790	-0.221304
C	-4.400593	2.569462	-2.115902
H	-5.270375	2.949483	-2.663442
H	-3.801679	3.437326	-1.816540
C	-4.432789	0.448201	-3.453437
H	-3.853634	-0.209704	-4.110598
H	-5.299945	0.797969	-4.024660
C	-4.896106	-0.325274	-2.212370
H	-5.504029	-1.182307	-2.518111
C	-3.574120	1.643766	-3.017824
H	-3.232038	2.193144	-3.900548
C	5.414975	-0.982929	-1.434405
H	5.924142	-1.515216	-2.243722
C	5.184982	-1.936561	-0.254142
H	4.590247	-2.796845	-0.578234
H	6.144182	-2.320895	0.110786
C	6.268704	0.206856	-0.975475
H	7.242961	-0.146217	-0.618794
H	6.457764	0.883637	-1.816771
C	5.302447	0.006186	1.330470
H	4.792292	0.537880	2.142152
H	6.262179	-0.344248	1.726836

C	4.456046	-1.188630	0.870464
H	4.280871	-1.866874	1.711246
C	5.536516	0.955282	0.146608
H	6.138018	1.809424	0.473914
C	-2.733181	-2.211013	3.279975
H	-3.553943	-1.945103	3.954175
C	-2.988586	-3.598178	2.673250
H	-3.074244	-4.345461	3.470647
H	-3.939151	-3.598946	2.126884
C	-1.412126	-2.231239	4.060759
H	-1.226464	-1.248851	4.510479
H	-1.471813	-2.957767	4.879058
C	-0.516302	-3.987733	2.504772
H	-0.555892	-4.744100	3.297077
H	0.303850	-4.258031	1.831484
C	-0.264897	-2.601738	3.110724
H	0.683291	-2.602646	3.658168
C	-1.837006	-3.963258	1.726001
H	-2.019196	-4.946398	1.281855
C	-0.407058	1.135547	0.476178
C	0.800105	1.648225	-0.118580
N	-0.957461	1.727986	1.508984
N	1.344759	2.764228	0.301443
C	-0.374016	2.856020	1.983560
C	0.773296	3.395704	1.357516
C	-0.934950	3.512387	3.104763
C	-0.365559	4.665428	3.568667
C	0.778770	5.211349	2.936800
C	1.339801	4.594699	1.852711
H	-1.813172	3.075603	3.564776
H	-0.790245	5.172312	4.427316
H	1.211879	6.126658	3.323178
H	2.217554	4.989272	1.355041
O	0.612439	-2.989996	-1.875971
C	1.770087	-3.603124	-2.310756
H	2.592580	-3.559724	-1.565874
H	1.601110	-4.675414	-2.510889
H	2.182344	-3.175439	-3.245902

Zero-point correction= 0.893971 (Hartree/Particle)
Thermal correction to Energy= 0.933945
Thermal correction to Enthalpy= 0.934890
Thermal correction to Gibbs Free Energy= 0.822782
Sum of electronic and zero-point Energies= -2621.335448
Sum of electronic and thermal Energies= -2621.295473
Sum of electronic and thermal Enthalpies= -2621.294529
Sum of electronic and thermal Free Energies= -2621.406636

[(*S*)-Quinox-Ad$_3$]: II-A$_{branch}$

C	1.703309	3.789382	-1.216640
C	2.462521	2.796327	-1.876622
C	1.519748	1.182843	-0.558182
C	0.673822	2.179196	0.038517
P	1.497415	-0.544720	0.122035
P	-0.665878	1.656311	1.215229
Cu	-0.550268	-0.701952	1.229408
B	-2.026262	-1.668631	0.204530
O	-3.298832	-1.170986	-0.109823
O	-2.010146	-3.031518	-0.136594
C	-4.041311	-2.155879	-0.847020
C	-3.352728	-3.475444	-0.392427
C	-0.142563	-1.145444	3.358662
H	0.760700	-1.742592	3.347407
H	-0.098237	-0.203586	3.897663
C	-1.347948	-1.677212	2.986797
C	1.609402	-1.652713	-1.394996
C	3.118647	-0.497375	1.101973
C	0.516018	-1.157073	-2.373441
H	0.758053	-0.149992	-2.724449
H	-0.443938	-1.104344	-1.849951
C	2.956366	-1.702519	-2.138433
H	3.257079	-0.696309	-2.439132
H	3.729501	-2.101259	-1.474765
C	1.246130	-3.088278	-0.942755
H	0.291523	-3.086225	-0.413128
H	2.007549	-3.468999	-0.254256
C	4.393534	-0.008940	0.381047
H	4.245557	1.000121	-0.009856
H	4.628239	-0.643284	-0.472725
C	3.378875	-1.913399	1.657951
H	3.608727	-2.595219	0.833190
H	2.480074	-2.300175	2.148918
C	2.861508	0.471654	2.282408
H	1.947158	0.187577	2.806272
H	2.710230	1.486927	1.895679
C	-5.515392	-2.051001	-0.486829
H	-6.079555	-2.877611	-0.929838
H	-5.930284	-1.115840	-0.869662
H	-5.659653	-2.068387	0.593925
C	-3.834662	-1.854531	-2.332709
H	-4.130652	-0.820469	-2.525471
H	-4.434300	-2.511297	-2.968653
H	-2.782314	-1.961589	-2.608873
C	-3.343920	-4.586810	-1.429938

H	-4.366556	-4.904794	-1.656005
H	-2.797508	-5.448541	-1.039777
H	-2.866745	-4.275283	-2.358402
C	-3.905975	-4.011567	0.930559
H	-3.240390	-4.798575	1.292289
H	-4.908307	-4.431756	0.813365
H	-3.942224	-3.224742	1.687857
C	5.308266	0.942631	2.520942
H	5.167987	1.963539	2.147876
H	6.161856	0.961356	3.208111
C	5.588271	-0.009793	1.352775
H	6.477947	0.324822	0.809790
C	5.818981	-1.425933	1.897147
H	6.048966	-2.113935	1.075545
H	6.680027	-1.432155	2.575148
C	4.256558	-0.943624	3.801424
H	5.082604	-0.952927	4.521257
H	3.358603	-1.276534	4.334459
C	4.046727	0.474977	3.255846
H	3.823174	1.158604	4.081327
C	4.560616	-1.897097	2.638213
H	4.715361	-2.909864	3.023404
C	2.850481	-2.627598	-3.363541
H	3.816489	-2.636260	-3.879197
C	2.498439	-4.047874	-2.900581
H	2.443205	-4.723305	-3.762262
H	3.283666	-4.431608	-2.238347
C	1.761590	-2.116861	-4.316151
H	1.702408	-2.764171	-5.198805
H	2.010820	-1.108852	-4.667321
C	1.154744	-4.019605	-2.161154
H	0.899703	-5.025367	-1.812621
C	0.057368	-3.508502	-3.101677
H	-0.892605	-3.485214	-2.561994
H	-0.055209	-4.184038	-3.957932
C	0.415320	-2.097100	-3.580539
H	-0.366834	-1.725121	-4.251009
C	3.358194	3.170086	-2.906741
C	3.492183	4.488899	-3.242231
C	1.866950	5.148469	-1.576007
C	2.744361	5.485942	-2.569052
N	2.370825	1.495059	-1.505264
N	0.803501	3.448605	-0.260640
H	3.924470	2.388568	-3.399297
H	4.179867	4.781320	-4.027298
H	2.872153	6.525136	-2.849197

H	1.277127	5.889906	-1.050285
C	-2.664842	-1.065930	3.394759
H	-2.521911	0.004014	3.578209
H	-3.386276	-1.143927	2.577739
C	-3.227228	-1.741926	4.647997
H	-3.395967	-2.808943	4.472927
H	-4.181918	-1.294446	4.936994
H	-2.534379	-1.647525	5.488468
H	-1.385620	-2.718989	2.678654
C	-2.198046	2.442109	0.475572
C	-2.304225	2.002277	-1.000804
C	-2.257828	3.978723	0.556943
C	-3.399719	1.857238	1.251689
H	-1.465085	2.412310	-1.574136
H	-2.257835	0.911357	-1.064260
C	-3.623139	2.510950	-1.600512
H	-2.204065	4.300794	1.602927
H	-1.402187	4.416540	0.038544
C	-3.574279	4.484312	-0.056226
H	-3.375915	0.767691	1.194252
H	-3.339577	2.145589	2.309667
C	-4.715820	2.372755	0.650963
H	-3.683275	2.191098	-2.646207
C	-3.660873	4.043271	-1.524574
C	-4.802392	1.922864	-0.814014
H	-3.594521	5.577707	0.001632
C	-4.761064	3.903940	0.725166
H	-5.552408	1.947096	1.214497
H	-2.824621	4.467943	-2.092119
H	-4.585443	4.421757	-1.975825
H	-4.764713	0.831956	-0.860498
H	-5.751095	2.251570	-1.254697
H	-5.704411	4.275257	0.307999
H	-4.717205	4.232237	1.770492
C	-0.263572	2.724529	2.658129
H	-0.052246	3.752089	2.357989
H	0.607994	2.306558	3.165272
H	-1.103678	2.712827	3.356605

Zero-point correction= 1.147399 (Hartree/Particle)
Thermal correction to Energy= 1.200105
Thermal correction to Enthalpy= 1.201050
Thermal correction to Gibbs Free Energy= 1.064649
Sum of electronic and zero-point Energies= -3074.437041
Sum of electronic and thermal Energies= -3074.384335
Sum of electronic and thermal Enthalpies= -3074.383391
Sum of electronic and thermal Free Energies= -3074.519791

[(*S*)-Quinox-Ad$_3$]: II-B$_{branch}$

C	1.540508	-2.646011	2.696961
C	2.094875	-1.492340	3.294133
C	1.281125	-0.201380	1.586635
C	0.675612	-1.369763	1.003416
P	1.137023	1.458160	0.751715
P	-0.418991	-1.207655	-0.490337
Cu	-0.593387	1.086363	-0.829529
B	-2.253683	1.960544	-0.018246
O	-2.239526	2.975803	0.946450
O	-3.580216	1.797883	-0.448921
C	-3.585907	3.336443	1.294899
C	-4.375486	2.897881	0.026085
C	0.124404	1.570011	-2.838289
H	1.175571	1.832798	-2.775790
H	-0.122378	0.700076	-3.434143
C	-0.846991	2.458809	-2.451172
C	0.439192	-2.325248	-1.749765
C	-2.047345	-1.947814	0.119013
C	0.645940	2.491552	2.185151
C	-3.151552	-1.511586	-0.873994
H	-2.977277	-1.968585	-1.853214
H	-3.132525	-0.428301	-1.006438
C	-2.339819	-1.299725	1.495154
H	-2.277590	-0.210580	1.406419
H	-1.589083	-1.615598	2.225757
C	-2.112433	-3.478530	0.272639
H	-1.319548	-3.828665	0.938554
H	-1.964490	-3.952284	-0.701839
C	-3.637999	4.825496	1.599421
H	-3.057631	5.037817	2.500323
H	-4.668657	5.150563	1.772673
H	-3.218982	5.410961	0.780443
C	-3.960223	2.530223	2.540415
H	-4.951453	2.796876	2.916893
H	-3.226362	2.735675	3.323168
H	-3.940328	1.457375	2.332269
C	-4.375594	3.959349	-1.076582
H	-5.009549	4.812460	-0.820298
H	-4.756678	3.507810	-1.995314
H	-3.364346	4.322837	-1.271956
C	-5.801201	2.434287	0.283144
H	-6.246907	2.086200	-0.651542
H	-6.408017	3.261692	0.663995
H	-5.844086	1.617537	1.002483
C	-0.551254	3.882588	-2.045578

H	0.503705	3.969970	-1.772511
H	-1.121896	4.147478	-1.152163
C	-0.879795	4.856513	-3.179988
H	-0.664713	5.887583	-2.886751
H	-1.940028	4.798601	-3.445629
H	-0.298453	4.626648	-4.077130
H	-1.874350	2.265180	-2.750026
H	0.813275	3.541921	1.936025
H	1.206633	2.224697	3.081714
H	-0.425587	2.364621	2.335672
C	0.802930	-3.769916	-1.343592
H	1.440778	-3.761453	-0.457574
H	-0.087851	-4.338886	-1.080637
C	-0.449831	-2.374828	-3.011141
H	-0.749826	-1.363329	-3.304120
H	-1.367843	-2.929676	-2.793373
C	1.760513	-1.601082	-2.092067
H	1.557755	-0.559945	-2.351668
H	2.404593	-1.592166	-1.206107
C	-3.759877	-3.241457	2.164618
H	-3.002152	-3.557497	2.891017
H	-4.734367	-3.560563	2.552173
C	-3.493946	-3.900789	0.805292
H	-3.507161	-4.990178	0.916154
C	-4.576987	-3.467915	-0.192205
H	-5.564689	-3.781405	0.165592
H	-4.412896	-3.956403	-1.160040
C	-4.792252	-1.269503	0.995496
H	-5.793108	-1.524042	1.363718
H	-4.744467	-0.186224	0.863759
C	-4.531756	-1.943574	-0.356145
H	-5.289946	-1.622708	-1.077242
C	-3.725635	-1.716914	2.000772
H	-3.904261	-1.235846	2.968507
C	0.626483	-4.506278	-3.746022
H	-0.296633	-5.056879	-3.531227
H	1.132144	-5.029057	-4.565974
C	1.526952	-4.479243	-2.503164
H	1.755644	-5.504348	-2.194192
C	2.826916	-3.738683	-2.837807
H	3.493883	-3.735810	-1.968148
H	3.355107	-4.249158	-3.651419
C	0.291383	-3.067777	-4.163215
H	-0.357779	-3.079989	-5.044528
C	1.584932	-2.306795	-4.483698
H	2.100284	-2.781016	-5.326563

H	1.352335	-1.278379	-4.783173
C	2.490274	-2.299297	-3.245041
H	3.411946	-1.749971	-3.464026
N	0.816761	-2.551952	1.554071
N	1.945542	-0.275980	2.713860
C	1.722991	-3.908502	3.310175
C	2.430769	-3.998071	4.476854
C	2.816982	-1.609871	4.505532
C	2.979933	-2.840136	5.079881
H	1.286949	-4.777311	2.831586
H	2.573632	-4.961807	4.951738
H	3.532209	-2.936576	6.007506
H	3.227306	-0.708693	4.945389
C	2.943362	1.934475	0.487821
C	3.648518	0.818211	-0.304282
C	3.735004	2.220378	1.778207
C	2.949379	3.211037	-0.382510
H	3.654386	-0.111744	0.275770
H	3.097461	0.621826	-1.228813
C	5.091138	1.222867	-0.638020
H	3.259818	3.034573	2.334591
H	3.725642	1.341059	2.425899
C	5.179035	2.621152	1.437792
H	2.400436	3.026335	-1.310731
H	2.436729	4.029184	0.137546
C	4.391626	3.626666	-0.715410
H	5.569222	0.409286	-1.193173
C	5.860905	1.485117	0.663378
C	5.077496	2.495956	-1.494943
H	5.722515	2.809476	2.369332
C	5.166985	3.894221	0.581490
H	4.367444	4.534558	-1.326606
H	5.882865	0.575711	1.274877
H	6.900583	1.750077	0.439575
H	4.546131	2.310114	-2.435493
H	6.102122	2.785920	-1.753823
H	6.192152	4.204195	0.348591
H	4.698943	4.715369	1.136365

Zero-point correction= 1.147635 (Hartree/Particle)
Thermal correction to Energy= 1.200296
Thermal correction to Enthalpy= 1.201240
Thermal correction to Gibbs Free Energy= 1.064473
Sum of electronic and zero-point Energies= -3074.430690
Sum of electronic and thermal Energies= -3074.378029
Sum of electronic and thermal Enthalpies= -3074.377085
Sum of electronic and thermal Free Energies= -3074.513852

[(*S*)-Quinox-Ad$_3$]: II-C$_{branch}$

C	-0.667466	3.979125	1.501736
C	-1.610259	3.140314	2.138756
C	-1.124549	1.456098	0.670241
C	-0.112804	2.284797	0.069806
P	-1.490767	-0.202207	-0.073572
P	0.974909	1.576812	-1.263353
Cu	0.535691	-0.696691	-1.214324
B	1.870361	-1.922228	-0.252732
O	2.248551	-1.962063	1.099763
O	2.554719	-2.961495	-0.907722
C	3.334974	-2.889212	1.277420
C	3.148645	-3.839036	0.058142
C	-0.055547	-1.011330	-3.443723
H	-1.135237	-0.963072	-3.531106
H	0.511287	-0.216256	-3.913997
C	0.543500	-2.127475	-2.963704
C	0.535260	2.673746	-2.678135
H	0.459242	3.718785	-2.373626
H	-0.412928	2.346797	-3.103595
H	1.303134	2.577410	-3.449522
C	-1.807728	-1.343299	1.392158
C	-3.106087	0.222745	-0.975875
C	-0.689693	-1.084743	2.430880
H	-0.807015	-0.082879	2.851770
H	0.292780	-1.143739	1.955939
C	-3.169617	-1.235443	2.102677
H	-3.309386	-0.219672	2.481564
H	-3.980884	-1.449632	1.401051
C	-1.642533	-2.784574	0.850947
H	-0.671513	-2.872337	0.354993
H	-2.408096	-3.002065	0.098292
C	-4.221393	0.927503	-0.172596
H	-3.847491	1.866239	0.242814
H	-4.545817	0.320422	0.670554
C	-3.666467	-1.082975	-1.579679
H	-3.975632	-1.763964	-0.781031
H	-2.886185	-1.595500	-2.152749
C	-2.715239	1.174398	-2.130268
H	-1.901128	0.739071	-2.711314
H	-2.345790	2.119214	-1.713970
H	1.620562	-2.237206	-3.038903
C	-0.204535	-3.380711	-2.598077
H	0.217718	-3.794619	-1.681190
H	-1.254217	-3.143427	-2.396566
C	4.631870	-2.086745	1.197504

H	5.508337	-2.702502	1.416606
H	4.587820	-1.271768	1.923794
H	4.749233	-1.654097	0.202483
C	3.232028	-3.552147	2.641975
H	3.326360	-2.794725	3.423797
H	4.038024	-4.281092	2.772735
H	2.281298	-4.064067	2.780982
C	2.146176	-4.965080	0.322806
H	2.544457	-5.712690	1.014147
H	1.915285	-5.457783	-0.624724
H	1.213886	-4.569772	0.733785
C	4.435926	-4.418144	-0.509444
H	4.202773	-5.065283	-1.358239
H	4.956451	-5.017806	0.243897
H	5.107590	-3.633578	-0.858179
C	-0.104625	-4.425064	-3.713502
H	-0.623809	-5.345982	-3.434546
H	0.941549	-4.675425	-3.912065
H	-0.543073	-4.051923	-4.643234
C	-5.021723	2.140092	-2.226522
H	-4.660485	3.094409	-1.826652
H	-5.884312	2.360508	-2.865642
C	-5.433213	1.212380	-1.078131
H	-6.210468	1.694188	-0.476384
C	-5.970110	-0.105441	-1.652770
H	-6.289297	-0.768533	-0.840308
H	-6.850167	0.086229	-2.277129
C	-4.439376	0.141789	-3.626012
H	-5.285163	0.336485	-4.294858
H	-3.658786	-0.341835	-4.224701
C	-3.915409	1.461038	-3.042415
H	-3.592269	2.121748	-3.853516
C	-4.871748	-0.784614	-2.482083
H	-5.248396	-1.726352	-2.893675
C	-3.251353	-2.252876	3.253965
H	-4.226644	-2.150160	3.741361
C	-3.103548	-3.672488	2.689584
H	-3.186542	-4.410132	3.496076
H	-3.910732	-3.879834	1.976852
C	-2.134451	-1.985159	4.270396
H	-2.203107	-2.699873	5.098976
H	-2.246321	-0.981275	4.696004
C	-1.741956	-3.801334	1.995115
H	-1.628640	-4.808835	1.580807
C	-0.619692	-3.526598	3.001290
H	0.346775	-3.603338	2.499579

H	-0.639676	-4.268684	3.808033
C	-0.776545	-2.112109	3.569350
H	0.030785	-1.911485	4.280810
C	-2.345355	3.626300	3.245976
C	-2.143175	4.905615	3.684201
C	-0.482359	5.302040	1.969593
C	-1.207174	5.750400	3.039147
N	-1.838470	1.883232	1.683445
N	0.073652	3.521223	0.461441
H	-3.058461	2.959861	3.716390
H	-2.702506	5.283572	4.532148
H	-1.065337	6.760960	3.404472
H	0.243713	5.926471	1.462712
C	2.701733	2.154711	-0.809920
C	3.009043	1.781444	0.655316
C	2.942810	3.663237	-1.018473
C	3.670077	1.363342	-1.718881
H	2.337796	2.324682	1.328169
H	2.838185	0.710159	0.811522
C	4.464140	2.149385	0.991936
H	2.753333	3.933796	-2.062129
H	2.250373	4.237206	-0.399648
C	4.395755	4.018782	-0.673456
H	3.508289	0.289187	-1.575734
H	3.467694	1.583626	-2.775066
C	5.124865	1.733828	-1.389345
H	4.661929	1.883827	2.035406
C	4.676067	3.656758	0.791358
C	5.423294	1.381897	0.073237
H	4.541876	5.093854	-0.821359
C	5.340856	3.238575	-1.597382
H	5.791662	1.167898	-2.047705
H	4.008111	4.223932	1.449786
H	5.704030	3.929246	1.057174
H	5.310562	0.308629	0.230262
H	6.461500	1.638081	0.314863
H	6.383613	3.499628	-1.383259
H	5.148342	3.508014	-2.642600

Zero-point correction= 1.149003 (Hartree/Particle)
Thermal correction to Energy= 1.201679
Thermal correction to Enthalpy= 1.202624
Thermal correction to Gibbs Free Energy= 1.065212
Sum of electronic and zero-point Energies= -3074.431055
Sum of electronic and thermal Energies= -3074.378379
Sum of electronic and thermal Enthalpies= -3074.377435
Sum of electronic and thermal Free Energies= -3074.514847

[(*S*)-Quinox-Ad$_3$]: II-D$_{branch}$

C	-1.749891	2.439077	2.857213
C	-2.443985	1.276868	3.262730
C	-1.470896	0.091731	1.565564
C	-0.770526	1.276262	1.146683
P	-1.171674	-1.520002	0.685430
P	0.374478	1.209496	-0.315838
Cu	0.522456	-1.113808	-0.831860
B	2.123175	-2.131023	-0.050569
O	2.624611	-2.077717	1.261695
O	2.855992	-3.102830	-0.743959
C	-0.238799	-1.328721	-2.911766
H	-1.116315	-1.958037	-2.823813
H	-0.392884	-0.347495	-3.345324
C	1.022720	-1.842080	-2.792797
H	1.149678	-2.900996	-2.589257
C	2.253251	-1.138845	-3.306837
H	3.101243	-1.408136	-2.674385
H	2.122964	-0.055233	-3.226685
C	-0.474243	2.436318	-1.477372
C	2.001802	1.876148	0.368565
C	-0.654997	-2.534081	2.124088
C	-2.869607	-2.174414	0.247957
C	3.113151	1.397740	-0.597163
H	2.986403	1.867959	-1.579318
H	3.026973	0.317240	-0.744522
C	2.215174	1.194070	1.741532
H	2.110091	0.110273	1.646404
H	1.462303	1.549736	2.450679
C	2.139185	3.398301	0.548369
H	1.347244	3.772278	1.203326
H	2.039926	3.898362	-0.419683
C	-3.820081	-2.466656	1.423287
H	-3.995502	-1.554739	1.997946
H	-3.363091	-3.192094	2.104712
C	-2.620368	-3.494497	-0.519660
H	-2.151278	-4.230144	0.143689
H	-1.923800	-3.323834	-1.346583
C	-3.540950	-1.167863	-0.705873
H	-2.869967	-0.948384	-1.541389
H	-3.722524	-0.223280	-0.180036
C	2.548465	-1.519136	-4.760506
H	2.687544	-2.599557	-4.860699
H	3.460095	-1.029657	-5.114381
H	1.725478	-1.225772	-5.418340
C	-0.759940	3.872057	-0.985120

H	-1.362415	3.845429	-0.075117
H	0.164417	4.389719	-0.733969
C	0.375949	2.518474	-2.763433
H	0.612783	1.514220	-3.125650
H	1.329488	3.011978	-2.545246
C	-1.838943	1.788310	-1.811213
H	-1.693133	0.754946	-2.127345
H	-2.459120	1.758433	-0.907785
H	0.360103	-2.231109	2.386731
H	-1.328627	-2.406644	2.972742
H	-0.618309	-3.584392	1.826896
C	3.516712	-3.183508	1.485775
C	3.986090	-3.515664	0.036723
C	2.692210	-4.308086	2.116496
H	2.224722	-3.932125	3.029424
H	3.312691	-5.170188	2.375668
H	1.900067	-4.635803	1.439443
C	4.620755	-2.760709	2.442550
H	5.344447	-3.570781	2.576972
H	4.188546	-2.524601	3.417801
H	5.151391	-1.878684	2.085860
C	5.179587	-2.672070	-0.416913
H	5.322486	-2.818220	-1.490074
H	6.101398	-2.957754	0.096843
H	4.995306	-1.609116	-0.244425
C	4.265504	-4.988072	-0.224940
H	5.063651	-5.354228	0.428531
H	4.584104	-5.122296	-1.261187
H	3.374514	-5.595392	-0.065158
C	-4.593051	-3.036049	-1.995914
H	-3.930128	-2.839254	-2.846438
H	-5.527746	-3.439388	-2.401554
C	-3.945952	-4.057608	-1.050610
H	-3.746104	-4.987156	-1.592815
C	-4.887800	-4.337961	0.128162
H	-5.832118	-4.757593	-0.237024
H	-4.440620	-5.082117	0.797117
C	-5.149314	-3.032810	0.892807
H	-5.813985	-3.229887	1.740039
C	-5.804811	-2.013647	-0.049516
H	-6.763183	-2.400859	-0.414089
H	-6.014045	-1.083012	0.490022
C	-4.866655	-1.733162	-1.231529
H	-5.325663	-1.000991	-1.903574
C	3.718081	3.049794	2.482528
H	4.697555	3.310769	2.900017

H	2.959053	3.396685	3.193192
C	3.525276	3.740028	1.126283
H	3.593917	4.825641	1.252294
C	4.613858	3.264103	0.154131
H	4.503981	3.771794	-0.811439
H	5.605100	3.521436	0.544942
C	3.605277	1.531762	2.295287
H	3.729819	1.026480	3.258369
C	4.497146	1.746009	-0.033263
H	5.261183	1.398688	-0.736695
C	-2.833074	4.003570	-2.406958
H	-3.367017	4.579831	-3.171342
H	-3.469235	3.978661	-1.514829
C	-1.492820	4.671312	-2.078905
H	-1.668605	5.685598	-1.706114
C	-0.630625	4.731749	-3.347196
H	-1.139698	5.317960	-4.120821
H	0.319837	5.233955	-3.133311
C	-2.570482	2.576814	-2.903332
H	-3.520033	2.077757	-3.122725
C	-1.702257	2.617216	-4.167849
H	-2.223464	3.158119	-4.965815
H	-1.522736	1.599494	-4.533390
C	-0.367739	3.306625	-3.851181
H	0.252782	3.339989	-4.752289
C	4.678249	1.042570	1.316812
H	5.679700	1.237512	1.717968
H	4.572643	-0.037045	1.185783
N	-0.908890	2.408917	1.793721
N	-2.272121	0.104244	2.603037
C	-1.931855	3.647885	3.570589
C	-2.781436	3.680916	4.641552
C	-3.315302	1.335920	4.376707
C	-3.478586	2.516533	5.047198
H	-1.386052	4.523268	3.239341
H	-2.927123	4.604984	5.188910
H	-4.146194	2.567243	5.899576
H	-3.832587	0.429782	4.668655

Zero-point correction= 1.147566 (Hartree/Particle)
Thermal correction to Energy= 1.200276
Thermal correction to Enthalpy= 1.201220
Thermal correction to Gibbs Free Energy= 1.064117
Sum of electronic and zero-point Energies= -3074.431526
Sum of electronic and thermal Energies= -3074.378816
Sum of electronic and thermal Enthalpies= -3074.377872
Sum of electronic and thermal Free Energies= -3074.514975

[(*S*)-Quinox-Ad$_3$]: II-A$_{linear}$

C	1.112187	3.702497	-1.883826
C	2.064172	2.766605	-2.348025
C	1.330777	1.239476	-0.810270
C	0.328957	2.173919	-0.374291
P	1.490720	-0.382183	0.072323
P	-0.884824	1.688893	0.952311
Cu	-0.628145	-0.598364	1.155278
B	-2.031113	-1.798945	0.265193
O	-3.262669	-2.094827	0.869552
O	-1.885963	-2.653439	-0.838090
C	-0.190954	-0.903756	3.412313
H	0.889888	-0.849356	3.492577
C	-0.746926	-1.999214	2.828932
H	-0.126557	-2.805102	2.450540
C	-0.489777	2.988769	2.198194
H	-0.381945	3.964755	1.722808
H	0.438161	2.730082	2.708659
H	-1.289367	3.031105	2.940413
C	-3.971544	-3.088990	0.112241
C	-2.833043	-3.728687	-0.742550
C	-5.020326	-2.375415	-0.737783
H	-5.677207	-1.802749	-0.079659
H	-5.632601	-3.082488	-1.303738
H	-4.547775	-1.683232	-1.438339
C	-4.658331	-4.044508	1.078177
H	-5.122530	-4.877076	0.540285
H	-5.441317	-3.512868	1.624022
H	-3.954374	-4.447673	1.806602
C	-3.256362	-4.167488	-2.137446
H	-4.067729	-4.899823	-2.080380
H	-2.416620	-4.637529	-2.653528
H	-3.590082	-3.320232	-2.736727
C	-2.120435	-4.880820	-0.029470
H	-1.218896	-5.138782	-0.590710
H	-2.751979	-5.770577	0.039859
H	-1.816438	-4.586693	0.978214
C	3.046493	-0.026958	1.093392
C	1.814659	-1.638462	-1.291803
C	0.764649	-1.377105	-2.399553
H	0.946138	-0.401867	-2.859092
H	-0.237476	-1.372600	-1.963236
C	3.405558	-1.328996	1.841619
H	3.740461	-2.083546	1.122909
H	2.519795	-1.737414	2.340704
C	3.218076	-1.647518	-1.923166

H	3.448733	-0.660337	-2.331076
H	3.967509	-1.882770	-1.161704
C	1.531625	-3.036830	-0.688503
H	2.246510	-3.258572	0.111049
H	0.528651	-3.047334	-0.254481
C	4.307478	0.497090	0.372727
H	4.653341	-0.209074	-0.380061
H	4.081291	1.429552	-0.150776
C	2.638555	1.056742	2.121311
H	1.715715	0.769907	2.628471
H	2.434276	1.996246	1.593764
C	-0.972792	0.070717	4.253741
H	-1.997116	0.138045	3.872349
H	-0.535881	1.069681	4.168557
C	-0.991461	-0.340552	5.729561
H	0.024624	-0.414143	6.129183
H	-1.467655	-1.316952	5.850615
H	-1.540209	0.385994	6.335517
H	-1.807282	-2.205801	2.920612
C	2.256916	-2.436466	-4.109974
H	2.330100	-3.188539	-4.904250
H	2.447630	-1.458798	-4.567715
C	3.300446	-2.719818	-3.022176
H	4.304196	-2.696944	-3.459614
C	3.038632	-4.099069	-2.401600
H	3.118069	-4.878879	-3.167889
H	3.795645	-4.316489	-1.638541
C	0.590092	-3.836829	-2.863574
H	-0.406234	-3.839926	-2.419840
H	0.627669	-4.615128	-3.635189
C	0.857127	-2.460701	-3.483875
H	0.103281	-2.251614	-4.249664
C	1.636547	-4.115132	-1.778630
H	1.445482	-5.092812	-1.322860
C	5.439141	0.735567	1.391024
H	6.323533	1.088570	0.851088
C	5.770748	-0.574522	2.119192
H	6.106350	-1.330458	1.400024
H	6.591668	-0.413958	2.827228
C	4.524009	-1.075453	2.860763
H	4.751925	-2.012668	3.378065
C	4.064490	-0.021568	3.877187
H	4.844277	0.141733	4.629489
H	3.173442	-0.376596	4.408038
C	3.754727	1.291706	3.146350
H	3.419979	2.044414	3.867685

C	5.008914	1.788853	2.418396
H	4.799671	2.740908	1.917654
H	5.816867	1.970804	3.136405
C	2.932989	3.120767	-3.407762
C	2.844863	4.364703	-3.969055
C	1.045337	4.985731	-2.476587
C	1.895437	5.304749	-3.498942
N	2.167439	1.541845	-1.774123
N	0.255081	3.378336	-0.884206
H	3.652686	2.384634	-3.745443
H	3.507990	4.641053	-4.780671
H	1.845090	6.284165	-3.960326
H	0.307663	5.684880	-2.101095
C	-2.549674	2.244341	0.293090
C	-2.775911	1.614732	-1.096288
C	-2.742010	3.770862	0.194926
C	-3.601352	1.670107	1.271276
H	-2.042551	2.007750	-1.809390
H	-2.627353	0.531561	-1.036054
C	-4.193385	1.937445	-1.591416
H	-2.608548	4.233935	1.178314
H	-1.990042	4.197841	-0.471288
C	-4.156984	4.090320	-0.314163
H	-3.480526	0.584337	1.348704
H	-3.452664	2.093116	2.273567
C	-5.016230	2.008133	0.776470
H	-4.337760	1.478442	-2.574881
C	-4.362421	3.458873	-1.697557
C	-5.224427	1.374728	-0.604336
H	-4.269348	5.177185	-0.386608
C	-5.189644	3.528813	0.672230
H	-5.745184	1.598647	1.483107
H	-3.635360	3.868819	-2.408207
H	-5.361386	3.701547	-2.077990
H	-5.110596	0.292422	-0.527526
H	-6.239948	1.576928	-0.964343
H	-6.204867	3.767501	0.334669
H	-5.057807	3.993996	1.656254

Zero-point correction= 1.148932 (Hartree/Particle)
Thermal correction to Energy= 1.201584
Thermal correction to Enthalpy= 1.202529
Thermal correction to Gibbs Free Energy= 1.064907
Sum of electronic and zero-point Energies= -3074.429117
Sum of electronic and thermal Energies= -3074.376465
Sum of electronic and thermal Enthalpies= -3074.375520
Sum of electronic and thermal Free Energies= -3074.513142

[(*S*)-Quinox-Ad$_3$]: II-B$_{linear}$

C	0.606746	-2.410516	3.177686
C	1.485282	-1.393664	3.612987
C	1.070134	-0.201068	1.702161
C	0.179711	-1.239381	1.257049
P	1.305032	1.343053	0.683312
P	-0.722399	-1.071353	-0.356351
Cu	-0.359233	1.169025	-0.969626
B	-1.749416	2.510801	-0.303306
O	-3.145304	2.424706	-0.397925
O	-1.426249	3.776119	0.198603
C	-3.721279	3.725618	-0.189066
C	-2.619047	4.431823	0.656411
C	0.262093	1.099465	-3.110532
H	-0.019556	0.094642	-3.409415
C	-0.742253	2.003837	-2.872847
C	0.045682	-2.487824	-1.351146
C	-2.513897	-1.443521	0.095564
C	0.875673	2.576497	1.972478
C	-3.386950	-0.920773	-1.072220
H	-3.165980	-1.472049	-1.991954
H	-3.162123	0.133919	-1.251084
C	-2.859732	-0.610127	1.353616
H	-2.611704	0.439120	1.172680
H	-2.271135	-0.959429	2.207016
C	-2.868014	-2.917423	0.365999
H	-2.244016	-3.307159	1.173841
H	-2.673591	-3.519451	-0.526311
C	-5.066462	3.580718	0.505672
H	-5.772403	3.073555	-0.156386
H	-5.479743	4.563322	0.753839
H	-4.983016	2.995449	1.422078
C	-3.911882	4.358359	-1.568320
H	-4.404771	5.332210	-1.504178
H	-4.531626	3.695162	-2.175845
H	-2.951493	4.483684	-2.073897
C	-2.746218	4.146802	2.154750
H	-3.598740	4.668067	2.598408
H	-1.835488	4.484520	2.654566
H	-2.858224	3.074979	2.338094
C	-2.489077	5.928816	0.421751
H	-1.696986	6.332341	1.056764
H	-3.422620	6.442931	0.671049
H	-2.235051	6.146256	-0.615731
H	-1.783687	1.740708	-3.015393
H	1.079212	3.579271	1.594501

H	1.418236	2.388645	2.899449
H	-0.198262	2.512946	2.142186
C	0.180409	-3.876606	-0.689117
H	0.757320	-3.800060	0.234646
H	-0.794648	-4.278406	-0.414555
C	-0.751916	-2.647187	-2.663933
H	-0.898120	-1.675855	-3.145857
H	-1.748036	-3.043778	-2.446387
C	1.476323	-2.004586	-1.680351
H	1.441926	-1.004907	-2.115935
H	2.049779	-1.927399	-0.750452
C	1.679839	1.488679	-3.437799
H	2.395119	0.886603	-2.869244
H	1.843693	2.530445	-3.145555
C	1.976374	1.318300	-4.930290
H	1.318307	1.949499	-5.533336
H	1.817951	0.280317	-5.239802
H	3.012921	1.581336	-5.160027
H	-0.524347	3.066782	-2.826730
C	-4.677150	-2.217549	1.968429
H	-4.088790	-2.579187	2.819828
H	-5.735233	-2.329156	2.232342
C	-4.358459	-3.048899	0.719131
H	-4.579892	-4.103968	0.912171
C	-5.203002	-2.550930	-0.461513
H	-4.991854	-3.153761	-1.353098
H	-6.270009	-2.667159	-0.238762
C	-5.196501	-0.240002	0.513427
H	-4.964309	0.808208	0.315618
H	-6.263448	-0.314787	0.755710
C	-4.876951	-1.076253	-0.731266
H	-5.468755	-0.714421	-1.578291
C	-4.352064	-0.744401	1.689875
H	-4.564771	-0.143949	2.580871
C	0.077983	-4.985483	-2.950648
H	-0.923001	-5.378715	-2.738912
H	0.567807	-5.695980	-3.626237
C	0.881458	-4.856519	-1.649909
H	0.947052	-5.833571	-1.160505
C	2.291405	-4.349055	-1.976586
H	2.887729	-4.278712	-1.059775
H	2.802102	-5.052333	-2.644303
C	-0.031003	-3.608899	-3.618991
H	-0.610909	-3.690733	-4.543676
C	1.372529	-3.074115	-3.935394
H	1.877400	-3.739998	-4.644158

H	1.299739	-2.088863	-4.410921
C	2.182784	-2.970142	-2.637130
H	3.182743	-2.580523	-2.855237
C	0.395324	-3.550552	3.989206
C	1.047130	-3.659308	5.186115
C	2.146856	-1.528761	4.857103
C	1.929675	-2.641091	5.622578
N	-0.040092	-2.303819	1.991472
N	1.688681	-0.287460	2.855208
H	-0.286678	-4.312585	3.631200
H	0.890435	-4.530252	5.811937
H	2.433551	-2.749518	6.576063
H	2.814397	-0.734847	5.170327
C	3.172360	1.522639	0.537883
C	3.734983	0.271147	-0.160907
C	3.941419	1.745472	1.854264
C	3.408161	2.751316	-0.370041
H	3.585811	-0.607760	0.477416
H	3.193285	0.088777	-1.092700
C	5.231258	0.444793	-0.452320
H	3.572365	2.646761	2.353825
H	3.771828	0.908957	2.534677
C	5.443430	1.911418	1.565990
H	2.858710	2.634173	-1.306221
H	3.022851	3.654257	0.117570
C	4.906617	2.921602	-0.660288
H	5.604524	-0.455385	-0.951272
C	5.981144	0.653458	0.869715
C	5.435967	1.663447	-1.362917
H	5.969650	2.059752	2.514493
C	5.662595	3.129415	0.658489
H	5.046540	3.791978	-1.309149
H	5.847918	-0.220579	1.517281
H	7.055787	0.758455	0.681783
H	4.911119	1.514618	-2.313808
H	6.500073	1.783997	-1.595506
H	6.732106	3.268172	0.462891
H	5.303770	4.037628	1.155799

Zero-point correction= 1.148298 (Hartree/Particle)
Thermal correction to Energy= 1.200896
Thermal correction to Enthalpy= 1.201841
Thermal correction to Gibbs Free Energy= 1.064596
Sum of electronic and zero-point Energies= -3074.425650
Sum of electronic and thermal Energies= -3074.373052
Sum of electronic and thermal Enthalpies= -3074.372108
Sum of electronic and thermal Free Energies= -3074.509353

[(*S*)-Quinox-Ad$_3$]: II-C$_{linear}$

C	0.555906	3.798329	-1.744267
C	1.434625	2.901007	-2.392756
C	1.034207	1.324300	-0.784740
C	0.069248	2.201665	-0.180783
P	1.474636	-0.273477	0.053397
P	-0.975732	1.575107	1.219132
Cu	-0.471550	-0.743198	1.268085
B	-1.850039	-1.913668	0.309855
O	-3.244209	-1.819813	0.466320
O	-1.588893	-3.045741	-0.472766
C	-3.859953	-3.036976	0.012333
C	-2.817265	-3.553376	-1.020369
C	-0.057107	-1.089331	3.499199
H	-0.077537	-0.074969	3.890577
C	-1.220951	-1.657230	3.069868
C	-0.581632	2.812203	2.527214
H	-0.536289	3.825261	2.123711
H	0.373934	2.558529	2.987689
H	-1.352918	2.765547	3.299502
C	1.821530	-1.476706	-1.357996
C	3.100439	0.272671	0.878306
C	0.646245	-1.382320	-2.361729
H	0.645535	-0.398732	-2.838328
H	-0.300005	-1.509163	-1.832992
C	3.136995	-1.279553	-2.138066
H	3.152904	-0.282101	-2.582897
H	3.997581	-1.361617	-1.469681
C	1.804000	-2.894900	-0.734216
H	0.855717	-3.047939	-0.212254
H	2.610283	-3.001291	-0.001492
C	4.069890	1.124965	0.029340
H	3.574624	2.040619	-0.301654
H	4.379628	0.594316	-0.870537
C	3.846014	-0.982799	1.379449
H	4.181270	-1.584246	0.531267
C	2.695438	1.132719	2.097625
H	1.966374	0.597387	2.710420
H	2.205636	2.047446	1.743346
H	-2.170716	-1.139807	3.126047
C	-3.995498	-3.953035	1.230101
H	-4.518583	-4.881441	0.985754
H	-4.562641	-3.427504	2.001684
H	-3.014197	-4.201997	1.640542
C	-5.234161	-2.742152	-0.567388
H	-5.902868	-2.385860	0.219971

H	-5.669644	-3.649685	-0.996992
H	-5.188385	-1.978300	-1.343854
C	-2.994771	-2.924016	-2.404482
H	-3.879135	-3.314473	-2.914933
H	-2.117355	-3.150727	-3.013631
H	-3.083155	-1.837265	-2.328372
C	-2.727988	-5.066992	-1.139409
H	-1.989638	-5.338640	-1.897250
H	-3.692209	-5.488768	-1.439894
H	-2.425923	-5.520566	-0.195270
C	1.159012	-1.918833	3.819313
H	2.066119	-1.325509	3.693693
H	1.226353	-2.751420	3.110237
C	1.122452	-2.459260	5.252683
H	0.253892	-3.106374	5.399951
H	1.054570	-1.641999	5.976956
H	2.023668	-3.036415	5.479574
H	-1.277566	-2.729465	2.910274
C	2.099885	-2.247058	-4.211621
H	2.203241	-2.997196	-5.004444
H	2.100017	-1.261807	-4.692056
C	3.274103	-2.352282	-3.230852
H	4.216630	-2.187626	-3.763796
C	3.284471	-3.742734	-2.581982
H	3.412758	-4.515804	-3.348566
H	4.132017	-3.827329	-1.891078
C	0.788993	-3.854376	-2.805553
H	0.867819	-4.626229	-3.580670
H	-0.142965	-4.011552	-2.259424
C	0.787742	-2.461069	-3.446992
H	-0.058831	-2.373043	-4.136725
C	1.965750	-3.959741	-1.829187
H	1.965166	-4.948043	-1.358371
C	6.027804	0.234647	1.337408
H	6.927062	0.501259	1.904358
H	6.353373	-0.362055	0.477395
C	5.311305	1.503386	0.856638
H	5.985006	2.088783	0.222745
C	4.887028	2.342118	2.068181
H	5.766080	2.635429	2.653424
H	4.397499	3.263779	1.733711
C	4.637602	0.251123	3.423268
H	5.512282	0.522939	4.025058
H	3.974145	-0.333373	4.069542
C	3.925050	1.518691	2.933223
H	3.596024	2.112883	3.792067

C	5.072714	-0.586286	2.213768
H	5.574607	-1.496405	2.556976
H	3.173037	-1.612226	1.967096
C	0.364118	5.095531	-2.277359
C	1.019071	5.461686	-3.420624
C	2.097415	3.302017	-3.577026
C	1.889488	4.557409	-4.077222
N	-0.122722	3.416819	-0.633507
N	1.677993	1.673490	-1.871746
H	-0.311923	5.765523	-1.759609
H	0.871741	6.451724	-3.836423
H	2.394766	4.870895	-4.983389
H	2.763869	2.593353	-4.054103
C	-2.719497	2.040068	0.710011
C	-2.993486	1.485687	-0.704000
C	-3.037205	3.547751	0.731289
C	-3.652433	1.318085	1.710420
H	-2.349329	1.988884	-1.433460
H	-2.763459	0.417320	-0.731132
C	-4.464432	1.718241	-1.078794
H	-2.869848	3.956255	1.733893
H	-2.370473	4.078331	0.048493
C	-4.506151	3.781181	0.340210
H	-3.451550	0.243625	1.686740
H	-3.465774	1.681605	2.729679
C	-5.120622	1.569141	1.335333
H	-4.640965	1.313248	-2.080891
C	-4.761932	3.223099	-1.067032
C	-5.371465	1.002951	-0.068449
H	-4.707424	4.857563	0.351741
C	-5.420174	3.073149	1.349312
H	-5.762812	1.054870	2.057667
H	-4.125182	3.738656	-1.795360
H	-5.803061	3.404675	-1.358279
H	-5.151719	-0.066434	-0.069614
H	-6.423813	1.135068	-0.346503
H	-6.472109	3.249151	1.095980
H	-5.256589	3.482588	2.353252

Zero-point correction= 1.148257 (Hartree/Particle)
Thermal correction to Energy= 1.201013
Thermal correction to Enthalpy= 1.201957
Thermal correction to Gibbs Free Energy= 1.064376
Sum of electronic and zero-point Energies= -3074.429576
Sum of electronic and thermal Energies= -3074.376820
Sum of electronic and thermal Enthalpies= -3074.375876
Sum of electronic and thermal Free Energies= -3074.513458

[(*S*)-Quinox-Ad$_3$]: II-D$_{linear}$

C	-1.104238	2.268400	3.153804
C	-1.781246	1.115515	3.610408
C	-1.183658	-0.000720	1.703904
C	-0.480475	1.165388	1.245863
P	-1.189578	-1.543344	0.671134
P	0.496644	1.125423	-0.340391
Cu	0.485359	-1.134882	-0.943876
B	2.079596	-2.295090	-0.367287
O	2.658008	-2.372602	0.912746
O	2.713367	-3.247237	-1.175146
C	-0.519444	-1.452962	-3.080653
H	-1.573681	-1.304202	-2.854361
C	0.117717	-2.552573	-2.612388
H	-0.395145	-3.306072	-2.026876
C	-0.325523	2.508166	-1.342364
C	2.215839	1.654879	0.254047
C	-0.657734	-2.757419	1.939507
C	3.220092	1.207531	-0.834992
H	3.035559	1.747846	-1.769524
H	3.074107	0.143374	-1.043567
C	2.517478	0.862010	1.550160
H	2.358818	-0.206492	1.390068
H	1.844392	1.190039	2.347005
C	2.431265	3.152357	0.547803
H	1.726683	3.483468	1.314994
H	2.251713	3.751355	-0.348881
C	-0.671809	3.825226	-0.613691
H	-1.340657	3.625183	0.224465
H	0.220475	4.290433	-0.195619
C	0.582936	2.834190	-2.549239
H	0.869368	1.916152	-3.069304
H	1.509254	3.300257	-2.203386
C	-1.656083	1.914287	-1.858775
H	-1.475426	0.953687	-2.345131
H	-2.315606	1.717585	-1.006312
H	0.414774	-2.616532	2.085396
H	-1.192955	-2.629477	2.881461
H	-0.812457	-3.767714	1.553306
C	3.491808	-3.538865	1.004304
C	3.843944	-3.807629	-0.492553
C	2.643011	-4.649083	1.628183
H	2.272131	-4.305843	2.596911
H	3.219170	-5.565106	1.783671
H	1.779743	-4.878899	0.999276
C	4.681528	-3.237698	1.903501

H	5.368357	-4.089364	1.934939
H	4.331365	-3.044009	2.920156
H	5.231792	-2.360755	1.563408
C	5.083598	-3.046118	-0.966004
H	5.161716	-3.144548	-2.050935
H	5.999302	-3.438459	-0.515371
H	5.002386	-1.982598	-0.731996
C	3.971379	-5.278144	-0.863738
H	4.774372	-5.755452	-0.293227
H	4.207730	-5.368521	-1.926418
H	3.041390	-5.816283	-0.679601
C	0.076134	-0.582322	-4.153481
H	1.167390	-0.661390	-4.112011
H	-0.170836	0.464691	-3.976456
C	-0.426556	-0.973770	-5.546445
H	-1.515576	-0.883736	-5.608159
H	-0.164268	-2.009682	-5.775977
H	0.006771	-0.329077	-6.316435
H	1.112021	-2.816600	-2.952688
C	4.160784	2.599663	2.290122
H	3.482724	2.920616	3.089386
H	5.184100	2.789979	2.634279
C	3.878668	3.397968	1.011225
H	4.004798	4.467804	1.208231
C	4.853235	2.959172	-0.090348
H	4.674583	3.538694	-1.003991
H	5.885868	3.155846	0.220319
C	3.963059	1.106304	2.003116
H	4.145005	0.524433	2.912314
C	4.929970	0.656351	0.902995
H	5.968400	0.792096	1.227351
H	4.776649	-0.407923	0.706760
C	4.660241	1.463490	-0.372129
H	5.346607	1.143282	-1.162922
C	-0.435697	5.105574	-2.768605
H	0.493772	5.565365	-2.413446
H	-0.912982	5.822052	-3.446792
C	-1.359387	4.804040	-1.581656
H	-1.575338	5.731367	-1.041167
C	-2.668398	4.190196	-2.093436
H	-3.345776	3.996011	-1.253978
H	-3.178053	4.888692	-2.766900
C	-1.431168	3.175236	-4.018969
H	-1.927959	3.861338	-4.714264
H	-1.223324	2.254043	-4.573620
C	-0.124270	3.799663	-3.511958

H	0.540009	4.003931	-4.357588
C	-2.351909	2.881398	-2.826822
H	-3.278986	2.419618	-3.181962
C	-1.101360	3.440169	3.948448
C	-1.755983	3.444917	5.148769
C	-2.445795	1.143498	4.859779
C	-2.432335	2.287815	5.608135
N	-0.446690	2.259388	1.969246
N	-1.794769	-0.015783	2.864054
H	-1.759214	4.339468	5.760871
H	-2.940756	2.315974	6.564936
H	-2.952617	0.244305	5.189138
H	-0.574389	4.309723	3.574094
C	-3.014369	-1.907921	0.426379
C	-3.664837	-0.704340	-0.282189
C	-3.806788	-2.231249	1.705925
C	-3.091783	-3.126602	-0.522211
H	-3.620400	0.179685	0.365168
H	-3.106421	-0.465371	-1.193436
C	-5.127873	-1.014656	-0.628929
H	-3.367250	-3.100606	2.206188
H	-3.745781	-1.395621	2.406371
C	-5.272612	-2.535972	1.357255
H	-2.539477	-2.918581	-1.444293
H	-2.623780	-4.000648	-0.053956
C	-4.556024	-3.444092	-0.863002
H	-5.568655	-0.145213	-1.127149
C	-5.901211	-1.320802	0.660844
C	-5.184817	-2.231258	-1.563491
H	-5.818657	-2.753090	2.280967
C	-5.332891	-3.752341	0.423850
H	-4.585450	-4.310555	-1.531170
H	-5.875028	-0.452392	1.329092
H	-6.953951	-1.520482	0.430695
H	-4.649054	-2.015864	-2.495197
H	-6.224827	-2.449454	-1.831091
H	-6.374659	-3.994771	0.184777
H	-4.902524	-4.628910	0.921438

Zero-point correction= 1.148230 (Hartree/Particle)
Thermal correction to Energy= 1.201070
Thermal correction to Enthalpy= 1.202014
Thermal correction to Gibbs Free Energy= 1.063841
Sum of electronic and zero-point Energies= -3074.424626
Sum of electronic and thermal Energies= -3074.371786
Sum of electronic and thermal Enthalpies= -3074.370842
Sum of electronic and thermal Free Energies= -3074.509015

[(*S*)-Quinox-Ad$_3$]: TS-A$_{branch}$

C	-2.076361	3.951729	0.125566
C	-2.914652	3.074165	0.847849
C	-1.653769	1.286405	0.169316
C	-0.768546	2.188310	-0.518605
P	-1.395827	-0.554687	0.052416
P	0.716529	1.532961	-1.418074
Cu	0.421950	-0.724533	-1.282631
B	2.074926	-1.853335	-0.621315
O	3.338782	-1.373159	-0.294392
O	1.828007	-3.030921	0.083800
C	3.892800	-2.184872	0.757691
C	3.079525	-3.510074	0.602760
C	0.319796	-1.613373	-3.084728
H	-0.363709	-2.451565	-3.197259
H	0.365338	-0.985141	-3.972205
C	1.635899	-1.912177	-2.465605
C	-1.383238	-1.076287	1.867814
C	-2.956376	-1.072853	-0.887939
C	-0.314830	-0.183640	2.543759
H	-0.610628	0.869082	2.493833
H	0.631551	-0.277755	2.001575
C	-2.712290	-0.946058	2.636135
H	-3.116239	0.063192	2.536758
H	-3.443048	-1.637743	2.213001
C	-0.904361	-2.542207	1.970068
H	0.034612	-2.676268	1.427246
H	-1.639064	-3.210148	1.508895
C	-4.335952	-0.790207	-0.259971
H	-4.447262	0.275963	-0.049562
H	-4.447455	-1.316222	0.687354
C	-2.833253	-2.589645	-1.149205
H	-2.911533	-3.133585	-0.201453
H	-1.851714	-2.811775	-1.577341
C	-2.894436	-0.318328	-2.241103
H	-1.914442	-0.470410	-2.700896
H	-3.018190	0.757767	-2.065318
C	5.390649	-2.323503	0.534697
H	5.824328	-3.017650	1.260881
H	5.874202	-1.352274	0.662315
H	5.612373	-2.683025	-0.470218
C	3.622014	-1.476573	2.083081
H	4.088628	-0.490290	2.070788
H	4.027301	-2.036738	2.929311
H	2.550494	-1.339599	2.232623
C	2.824362	-4.269083	1.894177

H	3.767940	-4.547949	2.372420
H	2.267527	-5.183487	1.677932
H	2.237933	-3.675200	2.594229
C	3.674441	-4.448277	-0.448738
H	2.949757	-5.236403	-0.663534
H	4.599954	-4.911542	-0.098032
H	3.883654	-3.917023	-1.381060
C	-5.369531	-0.525419	-2.545182
H	-5.497713	0.551740	-2.387859
H	-6.175570	-0.854926	-3.211213
C	-5.454485	-1.266885	-1.206616
H	-6.419378	-1.062371	-0.731064
C	-5.312230	-2.775768	-1.451923
H	-5.402390	-3.320240	-0.504681
H	-6.118027	-3.127700	-2.106235
C	-3.838787	-2.309734	-3.428263
H	-4.608931	-2.664089	-4.123124
H	-2.865155	-2.505121	-3.890074
C	-4.003674	-0.804109	-3.181332
H	-3.918132	-0.266334	-4.131043
C	-3.947910	-3.060010	-2.093992
H	-3.834946	-4.135250	-2.265036
C	-2.514744	-1.319321	4.113390
H	-3.471047	-1.201580	4.633379
C	-2.057153	-2.781950	4.199250
H	-1.933643	-3.080834	5.246624
H	-2.819167	-3.437659	3.762595
C	-1.464922	-0.402114	4.752107
H	-1.344307	-0.644701	5.814024
H	-1.794158	0.641615	4.691706
C	-0.728326	-2.938642	3.447828
H	-0.397278	-3.981460	3.491545
C	0.319208	-2.037934	4.112891
H	1.291010	-2.161884	3.630027
H	0.446610	-2.321894	5.163989
C	-0.130654	-0.575880	4.015839
H	0.628527	0.075935	4.460522
C	-4.014867	3.593932	1.569970
C	-4.257686	4.940058	1.557716
C	-2.352311	5.339398	0.121995
C	-3.421928	5.818832	0.826700
N	-2.688642	1.736335	0.836304
N	-1.003155	3.476756	-0.554881
H	-4.642532	2.900339	2.116842
H	-5.099679	5.343935	2.107883
H	-3.636058	6.881343	0.830410

H	-1.695785	5.988531	-0.444955
C	2.823482	-1.277470	-3.196657
H	2.575812	-0.233610	-3.417821
H	3.713933	-1.258747	-2.565302
C	3.135503	-2.018652	-4.495940
H	3.393770	-3.063623	-4.294095
H	3.982503	-1.560204	-5.014383
H	2.275720	-2.015073	-5.169930
H	1.789078	-2.989095	-2.333465
C	2.172885	2.392801	-0.616815
C	2.310745	1.843952	0.817230
C	2.111100	3.929249	-0.566005
C	3.421299	1.966269	-1.423679
H	1.434487	2.129483	1.412790
H	2.347201	0.752364	0.784582
C	3.584570	2.396322	1.471936
H	2.015718	4.339542	-1.577354
H	1.230538	4.256051	-0.009204
C	3.384674	4.481874	0.095705
H	3.484358	0.875461	-1.457058
H	3.347941	2.332592	-2.454267
C	4.690888	2.533368	-0.772661
H	3.665427	1.993834	2.487608
C	3.506456	3.928385	1.523292
C	4.811235	1.973401	0.649765
H	3.318208	5.574162	0.131760
C	4.613068	4.064301	-0.722986
H	5.559047	2.224083	-1.363415
H	2.641785	4.239805	2.120703
H	4.400543	4.336856	2.008116
H	4.875819	0.882295	0.600256
H	5.727335	2.341198	1.126293
H	5.524534	4.473929	-0.272573
H	4.542069	4.471571	-1.738260
C	0.500072	2.385324	-3.028619
H	0.328716	3.455461	-2.904549
H	-0.357638	1.929382	-3.526382
H	1.380715	2.213516	-3.649864

Zero-point correction= 1.147934 (Hartree/Particle)
Thermal correction to Energy= 1.199321
Thermal correction to Enthalpy= 1.200265
Thermal correction to Gibbs Free Energy= 1.067695
Sum of electronic and zero-point Energies= -3074.415215
Sum of electronic and thermal Energies= -3074.363828
Sum of electronic and thermal Enthalpies= -3074.362884
Sum of electronic and thermal Free Energies= -3074.495453

[(*S*)-Quinox-Ad$_3$]: TS-B$_{branch}$

C	-0.676751	-3.189681	-2.623641
C	-1.715690	-2.369723	-3.117795
C	-1.205831	-0.768892	-1.566092
C	-0.167032	-1.616790	-1.038974
P	-1.437810	0.947840	-0.894999
P	0.837062	-1.047961	0.420949
Cu	0.076545	1.058709	0.773075
B	1.292487	2.703381	0.250592
O	0.811613	3.632512	-0.662374
O	2.686486	2.722557	0.247682
C	1.913096	4.229741	-1.364675
C	3.120312	3.924609	-0.416719
C	-0.366762	1.631044	2.623606
H	-1.382958	1.946107	2.854784
H	0.085402	1.068848	3.437106
C	0.490191	2.682068	2.003587
C	0.289050	-2.286305	1.744932
C	2.622711	-1.314812	-0.100412
C	-1.206461	1.881777	-2.454908
C	3.518447	-0.555526	0.909371
H	3.420953	-0.990355	1.907403
H	3.204196	0.489654	0.972270
C	2.789543	-0.633735	-1.479824
H	2.451370	0.406379	-1.409126
H	2.170674	-1.139524	-2.227802
C	3.106070	-2.772659	-0.192524
H	2.464094	-3.340125	-0.870613
H	3.045774	-3.241064	0.793528
C	1.621109	5.709621	-1.564538
H	0.763677	5.827054	-2.230907
H	2.478731	6.214807	-2.018909
H	1.385381	6.200135	-0.620016
C	2.029998	3.536194	-2.721586
H	2.826017	3.972253	-3.329996
H	1.086693	3.652313	-3.258378
H	2.225514	2.467807	-2.599439
C	3.328357	4.982897	0.668465
H	3.706504	5.919126	0.250569
H	4.058252	4.604893	1.387509
H	2.403021	5.190455	1.208684
C	4.437479	3.682252	-1.135403
H	5.211808	3.425520	-0.409168
H	4.751170	4.589439	-1.660496
H	4.366717	2.872514	-1.860571
C	-0.181382	4.066543	1.986478

H	-1.169918	3.964752	1.528592
H	0.357079	4.779612	1.360910
C	-0.307953	4.638695	3.396972
H	-0.784982	5.622956	3.375195
H	0.678740	4.754877	3.857572
H	-0.898730	3.983057	4.039822
H	1.480301	2.730240	2.463762
H	-1.373080	2.942824	-2.265162
H	-1.862634	1.516531	-3.245633
H	-0.165537	1.757918	-2.753677
C	0.333381	-3.788992	1.395717
H	-0.280116	-3.991093	0.515030
H	1.345371	-4.109724	1.151831
C	1.139201	-2.035590	3.008428
H	1.132014	-0.968310	3.252521
H	2.177947	-2.325676	2.821974
C	-1.180579	-1.927164	2.062474
H	-1.249145	-0.861059	2.293192
H	-1.799937	-2.112073	1.176743
C	4.705983	-2.148481	-2.029022
H	4.091310	-2.677752	-2.766322
H	5.745134	-2.201813	-2.373584
C	4.570351	-2.819709	-0.656275
H	4.882596	-3.866794	-0.727548
C	5.444381	-2.086754	0.370140
H	6.498753	-2.134152	0.074100
H	5.360633	-2.575616	1.347912
C	5.133115	0.049028	-0.899463
H	6.181153	0.039869	-1.221644
H	4.817824	1.089932	-0.817265
C	4.988922	-0.625511	0.467977
H	5.599904	-0.094285	1.204687
C	4.258903	-0.683992	-1.923921
H	4.348573	-0.198200	-2.901563
C	0.679199	-4.350631	3.826569
H	1.720156	-4.634627	3.632027
H	0.328656	-4.961402	4.666402
C	-0.182059	-4.620960	2.584888
H	-0.118139	-5.681276	2.319217
C	-1.638228	-4.249969	2.887184
H	-2.268611	-4.462035	2.015895
H	-2.018263	-4.854805	3.718681
C	0.602714	-2.860795	4.186747
H	1.220689	-2.660546	5.067809
C	-0.852718	-2.471666	4.476543
H	-1.228722	-3.036641	5.337141

H	-0.913088	-1.407732	4.730283
C	-1.708700	-2.759101	3.236331
H	-2.747225	-2.471071	3.429931
N	0.086587	-2.785981	-1.575935
N	-1.948100	-1.147494	-2.576742
C	-0.436922	-4.449116	-3.222724
C	-1.218017	-4.862901	-4.266891
C	-2.509214	-2.819099	-4.199190
C	-2.262688	-4.042772	-4.757928
H	0.366370	-5.058882	-2.826551
H	-1.041056	-5.828185	-4.726924
H	-2.868113	-4.392800	-5.586013
H	-3.297660	-2.168286	-4.558031
C	-3.264130	1.113213	-0.521635
C	-3.665445	0.009599	0.475333
C	-4.203972	1.068523	-1.739558
C	-3.423028	2.479303	0.184286
H	-3.553070	-0.975446	0.007473
H	-2.996463	0.044273	1.339650
C	-5.119831	0.202583	0.924965
H	-3.943638	1.863768	-2.445843
H	-4.088614	0.117667	-2.265185
C	-5.659436	1.260397	-1.281298
H	-2.757460	2.514930	1.051801
H	-3.125281	3.291631	-0.489841
C	-4.878614	2.678697	0.630401
H	-5.383968	-0.594880	1.626789
C	-6.043610	0.144874	-0.299066
C	-5.262468	1.565757	1.615432
H	-6.314870	1.219943	-2.157495
C	-5.804751	2.624431	-0.592003
H	-4.969765	3.652674	1.121432
H	-5.955593	-0.831779	-0.788563
H	-7.088489	0.260940	0.010790
H	-4.614748	1.607408	2.498314
H	-6.292911	1.707845	1.960737
H	-6.844642	2.783071	-0.284308
H	-5.548408	3.426994	-1.293175

Zero-point correction= 1.148225 (Hartree/Particle)
Thermal correction to Energy= 1.199354
Thermal correction to Enthalpy= 1.200298
Thermal correction to Gibbs Free Energy= 1.067615
Sum of electronic and zero-point Energies= -3074.411575
Sum of electronic and thermal Energies= -3074.360447
Sum of electronic and thermal Enthalpies= -3074.359503
Sum of electronic and thermal Free Energies= -3074.492186

[(*S*)-Quinox-Ad$_3$]: TS-C$_{branch}$

C	-1.532604	4.090836	0.377840
C	-2.241512	3.293406	1.303946
C	-1.363570	1.400120	0.364812
C	-0.541849	2.219265	-0.484926
P	-1.409509	-0.429392	0.068253
P	0.803701	1.442975	-1.507945
Cu	0.367189	-0.750220	-1.362051
B	1.999412	-1.962712	-0.744958
O	2.043822	-2.685078	0.441771
O	3.303016	-1.654553	-1.145097
C	3.409687	-2.759363	0.896536
C	4.193722	-2.527632	-0.431374
C	0.041247	-1.782214	-3.047023
H	-0.939532	-2.217070	-3.229389
H	0.508268	-1.410041	-3.955648
C	0.914363	-2.587818	-2.146140
C	0.512777	2.233147	-3.136236
H	0.321686	3.302622	-3.037934
H	-0.342721	1.736823	-3.595981
H	1.385475	2.064672	-3.770730
C	-1.371198	-1.198818	1.779441
C	-3.102018	-0.550006	-0.777250
C	-0.114564	-0.608938	2.466434
H	-0.235049	0.470028	2.608116
H	0.755940	-0.768359	1.822274
C	-2.598461	-0.982881	2.682069
H	-2.809301	0.083762	2.783900
H	-3.474432	-1.453988	2.226705
C	-1.140472	-2.719569	1.610435
H	-0.256342	-2.892968	0.994051
H	-1.994817	-3.182961	1.108791
C	-4.316175	0.073088	-0.057925
H	-4.142279	1.133832	0.133665
H	-4.481883	-0.398020	0.911338
C	-3.394385	-2.041354	-1.045378
H	-3.545751	-2.564876	-0.095967
H	-2.538451	-2.502428	-1.547117
C	-2.937515	0.173501	-2.138131
H	-2.064577	-0.226021	-2.661409
H	-2.756869	1.242694	-1.969022
H	1.874941	-2.809391	-2.622485
C	0.267931	-3.875077	-1.620938
H	0.836467	-4.288773	-0.784915
H	-0.723509	-3.636658	-1.224339
C	3.628742	-1.639238	1.911515

H	4.634545	-1.677339	2.337480
H	2.907159	-1.737080	2.724337
H	3.482107	-0.663980	1.445532
C	3.637259	-4.114841	1.547072
H	3.067636	-4.182199	2.476490
H	4.695899	-4.252425	1.787159
H	3.317800	-4.926482	0.893181
C	4.335749	-3.807537	-1.258497
H	5.056562	-4.497430	-0.812857
H	4.683461	-3.541798	-2.259107
H	3.377689	-4.324490	-1.358936
C	5.547079	-1.855118	-0.279288
H	6.011930	-1.739901	-1.260850
H	6.209804	-2.461929	0.344718
H	5.454541	-0.866282	0.167468
C	0.142797	-4.928154	-2.720648
H	-0.314193	-5.843802	-2.334495
H	1.127655	-5.189043	-3.122409
H	-0.465602	-4.562813	-3.550981
C	-5.398420	0.623654	-2.262248
H	-5.230047	1.694164	-2.097930
H	-6.306805	0.526362	-2.867993
C	-5.582499	-0.091291	-0.918393
H	-6.427852	0.349459	-0.380008
C	-5.850991	-1.581430	-1.167675
H	-6.008438	-2.099183	-0.214206
H	-6.766046	-1.705606	-1.757954
C	-4.451976	-1.480874	-3.249228
H	-5.336149	-1.612142	-3.883387
H	-3.599699	-1.918417	-3.780502
C	-4.199262	0.010812	-2.994989
H	-4.043779	0.524183	-3.949249
C	-4.655640	-2.196024	-1.907573
H	-4.834981	-3.261928	-2.080524
C	-2.363255	-1.630056	4.056970
H	-3.240750	-1.447256	4.685818
C	-2.162520	-3.140295	3.874442
H	-2.020444	-3.624429	4.847645
H	-3.055694	-3.584048	3.419271
C	-1.119884	-1.020730	4.719439
H	-0.967257	-1.465360	5.709705
H	-1.262115	0.056327	4.864249
C	-0.938624	-3.382395	2.982354
H	-0.793889	-4.456986	2.832274
C	0.305910	-2.784687	3.646889
H	1.174711	-2.974244	3.011750

H	0.486766	-3.262431	4.616983
C	0.105886	-1.274767	3.831464
H	0.996513	-0.837336	4.295918
C	-3.084784	3.910282	2.257611
C	-3.218979	5.271588	2.262434
C	-1.695885	5.495992	0.395917
C	-2.522897	6.069482	1.322513
N	-2.162614	1.939235	1.252433
N	-0.671769	3.521741	-0.504151
H	-3.617018	3.275523	2.956016
H	-3.868151	5.749629	2.986876
H	-2.649560	7.145730	1.343766
H	-1.145454	6.084554	-0.328426
C	2.326131	2.306836	-0.830608
C	2.358237	2.086322	0.695545
C	2.404224	3.817219	-1.127686
C	3.559716	1.619086	-1.455317
H	1.494431	2.568253	1.167362
H	2.283477	1.014975	0.911614
C	3.646683	2.674843	1.287400
H	2.405797	3.983171	-2.210194
H	1.529648	4.330283	-0.724471
C	3.690784	4.404010	-0.525730
H	3.524186	0.541860	-1.278398
H	3.557022	1.767732	-2.542424
C	4.848808	2.212814	-0.864864
H	3.650729	2.507509	2.369335
C	3.694484	4.180474	0.993480
C	4.866875	1.995176	0.654359
H	3.722233	5.477769	-0.737454
C	4.910620	3.717886	-1.155242
H	5.709239	1.710113	-1.318138
H	2.830233	4.679307	1.446963
H	4.594219	4.621565	1.437294
H	4.854759	0.926588	0.884556
H	5.788507	2.405733	1.082647
H	5.835492	4.144133	-0.749581
H	4.920826	3.892598	-2.237203

Zero-point correction= 1.148768 (Hartree/Particle)
Thermal correction to Energy= 1.199996
Thermal correction to Enthalpy= 1.200940
Thermal correction to Gibbs Free Energy= 1.069410
Sum of electronic and zero-point Energies= -3074.415127
Sum of electronic and thermal Energies= -3074.363899
Sum of electronic and thermal Enthalpies= -3074.362955
Sum of electronic and thermal Free Energies= -3074.494485

[(*S*)-Quinox-Ad$_3$]: TS-D$_{branch}$

C	-1.209116	-2.889331	-2.759948
C	-2.110560	-1.899678	-3.211818
C	-1.385743	-0.479388	-1.571787
C	-0.470831	-1.489295	-1.105738
P	-1.399506	1.195115	-0.768726
P	0.669206	-1.127068	0.319352
Cu	0.146995	1.036825	0.832827
B	1.412933	2.586521	0.307211
O	2.749453	2.546018	-0.070801
O	0.818172	3.740982	-0.207008
C	-0.177685	1.579569	2.742030
H	-1.044511	2.195800	2.965062
H	0.019918	0.829867	3.505968
C	1.018687	2.268850	2.216224
H	0.902594	3.357164	2.205078
C	2.344986	1.856680	2.854130
H	3.190093	2.138064	2.221640
H	2.369036	0.765080	2.937352
C	0.162024	-2.429724	1.588778
C	2.376148	-1.446389	-0.408509
C	-1.169996	2.248991	-2.249322
C	-3.168268	1.491107	-0.246800
C	3.418240	-0.834372	0.558511
H	3.397729	-1.346929	1.523833
H	3.176488	0.215582	0.736187
C	2.443032	-0.638418	-1.727449
H	2.178828	0.403524	-1.524550
H	1.725043	-1.035135	-2.452616
C	2.752748	-2.910713	-0.690060
H	2.010780	-3.371384	-1.346992
H	2.760781	-3.474618	0.247155
C	-4.210960	1.529305	-1.377704
H	-4.207388	0.584939	-1.927068
H	-3.962894	2.320111	-2.093714
C	-3.173883	2.853195	0.484500
H	-2.901512	3.654709	-0.212137
H	-2.421998	2.844494	1.279223
C	-3.548943	0.391629	0.763378
H	-2.801355	0.362573	1.561579
H	-3.542434	-0.586550	0.267309
C	2.510871	2.481023	4.239195
H	2.508862	3.574269	4.174351
H	3.455181	2.175602	4.698821
H	1.694113	2.186886	4.902950
C	0.041620	-3.895646	1.124579

H	-0.681232	-3.973562	0.310365
H	0.990690	-4.266011	0.735800
C	1.164046	-2.352938	2.760862
H	1.269982	-1.313682	3.091945
H	2.149316	-2.697341	2.431756
C	-1.226717	-1.982597	2.102410
H	-1.179116	-0.937223	2.417267
H	-1.955359	-2.041185	1.285099
H	-0.188066	2.022825	-2.668224
H	-1.934083	2.059949	-3.003627
H	-1.168483	3.294189	-1.938453
C	3.007653	3.618604	-0.996912
C	1.860767	4.622354	-0.652883
C	2.893099	3.054249	-2.412768
H	3.597573	2.229329	-2.531219
H	3.125189	3.813114	-3.163971
H	1.888287	2.671619	-2.599946
C	4.415146	4.142528	-0.758407
H	4.604295	5.032815	-1.365618
H	5.144626	3.378878	-1.038109
H	4.572847	4.392631	0.290760
C	2.212880	5.539464	0.519247
H	1.308174	6.057951	0.843856
H	2.960356	6.285326	0.237991
H	2.597166	4.965174	1.366321
C	1.350672	5.440759	-1.827711
H	2.154536	6.047392	-2.255316
H	0.559564	6.113992	-1.489808
H	0.941338	4.800091	-2.609362
C	-4.928533	2.018785	2.068069
H	-4.197490	1.998351	2.883665
H	-5.910203	2.217647	2.512995
C	-4.567575	3.127198	1.068961
H	-4.553542	4.094418	1.581033
C	-5.603009	3.155102	-0.063819
H	-6.598751	3.366361	0.342586
H	-5.364099	3.957534	-0.771232
C	-5.605505	1.801279	-0.787167
H	-6.337437	1.820182	-1.601104
C	-5.971694	0.691656	0.208809
H	-6.975116	0.866575	0.613652
H	-5.992462	-0.278364	-0.301149
C	-4.941022	0.664854	1.345055
H	-5.192765	-0.129108	2.055337
C	4.192608	-2.184274	-2.622638
H	5.185200	-2.259164	-3.081505

H	3.472225	-2.599514	-3.337012
C	4.156204	-2.985626	-1.314183
H	4.394306	-4.034674	-1.518204
C	5.176959	-2.404791	-0.326454
H	5.167388	-2.982464	0.605326
H	6.188540	-2.477404	-0.742433
C	3.852782	-0.716625	-2.330110
H	3.867681	-0.142323	-3.263012
C	4.828834	-0.938166	-0.041229
H	5.543893	-0.519976	0.674418
C	-1.786347	-4.325156	2.796138
H	-2.124641	-4.968110	3.617032
H	-2.524977	-4.410593	1.990884
C	-0.410201	-4.784076	2.298150
H	-0.472847	-5.818921	1.945865
C	0.603563	-4.690963	3.446305
H	0.297024	-5.342702	4.272409
H	1.587234	-5.037837	3.108435
C	-1.688833	-2.868917	3.265436
H	-2.669551	-2.519551	3.604414
C	-0.677926	-2.761506	4.414612
H	-1.005964	-3.370726	5.264708
H	-0.614566	-1.724236	4.761658
C	0.696346	-3.236289	3.926183
H	1.424981	-3.160856	4.739582
C	4.875018	-0.134372	-1.345030
H	5.880088	-0.172187	-1.781422
H	4.640615	0.912124	-1.129907
N	-0.391769	-2.653585	-1.703380
N	-2.168889	-0.688595	-2.601604
C	-1.152873	-4.139725	-3.419700
C	-1.975549	-4.381100	-4.485037
C	-2.949782	-2.172721	-4.317989
C	-2.880971	-3.390610	-4.936886
H	-0.450226	-4.878744	-3.053462
H	-1.938167	-5.337885	-4.992860
H	-3.523183	-3.605415	-5.783197
H	-3.632037	-1.397392	-4.645538

Zero-point correction= 1.147290 (Hartree/Particle)
Thermal correction to Energy= 1.198785
Thermal correction to Enthalpy= 1.199730
Thermal correction to Gibbs Free Energy= 1.066050
Sum of electronic and zero-point Energies= -3074.419094
Sum of electronic and thermal Energies= -3074.367599
Sum of electronic and thermal Enthalpies= -3074.366655
Sum of electronic and thermal Free Energies= -3074.500335

[(*S*)-Quinox-Ad$_3$]: TS-A$_{linear}$

C	1.318846	4.011290	-1.099657
C	2.257570	3.143714	-1.701669
C	1.385892	1.373474	-0.543011
C	0.399782	2.247120	0.030221
P	1.447155	-0.413880	-0.042828
P	-0.881520	1.556222	1.184116
Cu	-0.385390	-0.679529	1.234696
B	-1.931348	-2.028731	0.618996
O	-3.246694	-1.646264	0.359630
O	-1.657912	-3.222968	-0.054219
C	-0.281090	-1.390410	3.119102
H	0.531965	-2.095507	3.300526
C	-1.435654	-1.989154	2.403054
H	-1.412408	-3.079412	2.353825
C	-0.543364	2.606566	2.652505
H	-0.422617	3.653537	2.369889
H	0.369092	2.241028	3.126261
H	-1.357180	2.507168	3.372345
C	-3.802594	-2.542850	-0.622481
C	-2.918778	-3.810639	-0.423360
C	-3.615829	-1.912133	-2.002735
H	-4.096755	-0.932810	-2.022462
H	-4.066270	-2.528528	-2.784671
H	-2.555904	-1.775741	-2.229965
C	-5.280768	-2.746621	-0.330283
H	-5.695226	-3.523303	-0.980007
H	-5.829846	-1.821366	-0.517508
H	-5.443806	-3.032527	0.708942
C	-2.742536	-4.682911	-1.653514
H	-3.703066	-5.109621	-1.957130
H	-2.061185	-5.504878	-1.422867
H	-2.330525	-4.128596	-2.494299
C	-3.382364	-4.672616	0.753317
H	-2.606037	-5.407378	0.977542
H	-4.306896	-5.205943	0.519495
H	-3.550096	-4.069820	1.648961
C	3.032139	-0.432860	0.991388
C	1.622404	-1.320522	-1.680976
C	0.511814	-0.754635	-2.600618
H	0.696117	0.302524	-2.809788
H	-0.455131	-0.825028	-2.088630
C	3.288141	-1.898473	1.401348
H	3.545694	-2.489586	0.516250
H	2.376188	-2.325905	1.831576
C	2.979367	-1.215257	-2.402313

H	3.243679	-0.167512	-2.561055
H	3.757945	-1.667832	-1.781673
C	1.317809	-2.815579	-1.427534
H	2.086072	-3.253022	-0.782777
H	0.361163	-2.927300	-0.913994
C	4.316460	0.148028	0.364591
H	4.587471	-0.385131	-0.546292
H	4.161128	1.192386	0.084376
C	2.718612	0.393834	2.263440
H	1.796724	0.023484	2.718820
H	2.553016	1.443796	1.987230
C	-0.588640	-0.527267	4.323525
H	-1.401513	0.166583	4.071955
H	0.278125	0.103495	4.564470
C	-0.981505	-1.316731	5.578253
H	-0.180072	-2.004561	5.866062
H	-1.878056	-1.916591	5.391139
H	-1.186113	-0.659521	6.430685
H	-2.400243	-1.637157	2.775029
C	1.827592	-1.382059	-4.631941
H	1.806674	-1.900645	-5.597304
H	2.035849	-0.325128	-4.834484
C	2.926560	-1.972980	-3.739441
H	3.896866	-1.872242	-4.236689
C	2.633446	-3.455648	-3.473113
H	2.617445	-4.012790	-4.417095
H	3.428103	-3.888805	-2.854604
C	0.179623	-3.001250	-3.652516
H	-0.786063	-3.093153	-3.149552
H	0.117452	-3.558606	-4.594316
C	0.472002	-1.523204	-3.927355
H	-0.318465	-1.100198	-4.556094
C	1.281490	-3.582486	-2.758942
H	1.067736	-4.635541	-2.550215
C	5.480861	0.052653	1.369043
H	6.381686	0.454539	0.893953
C	5.715006	-1.413257	1.759878
H	5.976541	-2.002136	0.872730
H	6.557904	-1.485676	2.456616
C	4.443345	-1.977456	2.408484
H	4.602076	-3.025087	2.683063
C	4.092164	-1.160552	3.658770
H	4.897291	-1.238335	4.398327
H	3.182352	-1.558148	4.121947
C	3.877101	0.306839	3.264175
H	3.617598	0.890756	4.153291

C	5.153458	0.866901	2.625662
H	5.009655	1.921685	2.364684
H	5.986939	0.818878	3.336068
C	3.198205	3.661092	-2.623523
C	3.193761	4.996572	-2.918178
C	1.338116	5.390329	-1.416532
C	2.258148	5.867848	-2.308971
N	2.280630	1.824310	-1.388669
N	0.397991	3.532233	-0.225905
H	3.906702	2.973572	-3.070043
H	3.912957	5.398104	-3.622677
H	2.276861	6.923248	-2.555070
H	0.611025	6.035057	-0.937293
C	-2.514485	2.180719	0.532174
C	-2.707972	1.589294	-0.878445
C	-2.693996	3.708388	0.475815
C	-3.591958	1.585896	1.470747
H	-1.953058	1.997840	-1.561612
H	-2.569473	0.505487	-0.838898
C	-4.111904	1.929802	-1.398210
H	-2.569250	4.140160	1.475334
H	-1.928061	4.153325	-0.161689
C	-4.096936	4.047756	-0.056305
H	-3.482366	0.498748	1.519327
H	-3.467816	1.985228	2.484776
C	-4.992949	1.939480	0.949844
H	-4.235320	1.502454	-2.399358
C	-4.273982	3.454827	-1.461798
C	-5.166862	1.339980	-0.451004
H	-4.204872	5.136356	-0.102636
C	-5.157946	3.463029	0.885371
H	-5.739964	1.513305	1.627053
H	-3.531007	3.881902	-2.145229
H	-5.263725	3.712720	-1.855939
H	-5.049852	0.255201	-0.389993
H	-6.173331	1.549666	-0.831464
H	-6.162467	3.716870	0.527403
H	-5.050648	3.897606	1.885984

Zero-point correction= 1.147318 (Hartree/Particle)
Thermal correction to Energy= 1.198931
Thermal correction to Enthalpy= 1.199875
Thermal correction to Gibbs Free Energy= 1.065395
Sum of electronic and zero-point Energies= -3074.415974
Sum of electronic and thermal Energies= -3074.364362
Sum of electronic and thermal Enthalpies= -3074.363417
Sum of electronic and thermal Free Energies= -3074.497897

[(*S*)-Quinox-Ad$_3$]: TS-B$_{linear}$

C	-0.470692	-3.251935	-2.618441
C	-1.412829	-2.408678	-3.248808
C	-1.052707	-0.802020	-1.661499
C	-0.073101	-1.648387	-1.032916
P	-1.391282	0.898830	-0.993285
P	0.891243	-1.008009	0.420402
Cu	0.019974	1.102201	0.726843
B	1.184773	2.776754	0.280645
O	2.566468	2.890978	0.144776
O	0.563311	3.859651	-0.340399
C	2.867056	4.236210	-0.272931
C	1.558748	4.638854	-1.025142
C	-0.422686	1.577810	2.654434
H	-0.074321	0.819175	3.354658
C	0.652405	2.426280	2.120833
C	0.492109	-2.266886	1.764365
C	2.668577	-1.103888	-0.188060
C	-1.147565	1.871999	-2.525173
C	3.545861	-0.284503	0.789289
H	3.567808	-0.763878	1.771961
H	3.126843	0.716455	0.915538
C	2.676911	-0.375493	-1.555903
H	2.234766	0.619852	-1.437901
H	2.071511	-0.929708	-2.280738
C	3.292680	-2.500886	-0.355657
H	2.671441	-3.119387	-1.008669
H	3.344406	-2.997433	0.617783
C	4.121889	4.237779	-1.130886
H	4.983272	3.942153	-0.527490
H	4.310958	5.239896	-1.527084
H	4.039825	3.542643	-1.966362
C	3.106054	5.055497	0.995600
H	3.391743	6.084568	0.764105
H	3.913121	4.590253	1.565419
H	2.215126	5.077714	1.627632
C	1.569114	4.207368	-2.491310
H	2.266522	4.807736	-3.080820
H	0.569008	4.336622	-2.908950
H	1.845438	3.154418	-2.587091
C	1.188931	6.110229	-0.918039
H	0.264856	6.297100	-1.469565
H	1.975672	6.739942	-1.344381
H	1.027534	6.403357	0.119461
H	1.655301	2.225966	2.493002
H	-1.352721	2.921598	-2.313333

H	-1.772892	1.507304	-3.340567
H	-0.097102	1.786370	-2.805375
C	0.682585	-3.765639	1.452656
H	0.093033	-4.048548	0.578519
H	1.724215	-3.983164	1.212163
C	1.340816	-1.910032	3.003779
H	1.252430	-0.842140	3.226290
H	2.396179	-2.115220	2.797265
C	-1.002867	-2.037163	2.094785
H	-1.176242	-0.976831	2.293845
H	-1.613188	-2.307527	1.224410
C	-1.701233	2.243012	3.114914
H	-2.523952	1.516887	3.128107
H	-1.993645	3.016287	2.394812
C	-1.590128	2.882439	4.504057
H	-0.807293	3.647374	4.514408
H	-1.323897	2.129966	5.253192
H	-2.528907	3.353055	4.815877
H	0.430333	3.496961	2.139345
C	4.697277	-1.659756	-2.270389
H	4.093866	-2.231440	-2.985122
H	5.712003	-1.595151	-2.679690
C	4.721606	-2.377075	-0.913972
H	5.139505	-3.381655	-1.037019
C	5.582621	-1.576345	0.072762
H	5.626842	-2.093931	1.038197
H	6.610407	-1.498981	-0.300645
C	4.958018	0.549233	-1.100127
H	4.526669	1.543097	-0.965849
H	5.977164	0.669933	-1.486064
C	4.980807	-0.176489	0.249685
H	5.579811	0.397791	0.963698
C	4.108316	-0.254754	-2.091168
H	4.080288	0.260354	-3.057339
C	1.106114	-4.234760	3.890293
H	2.165999	-4.430564	3.690101
H	0.819851	-4.850958	4.750387
C	0.258908	-4.612632	2.667280
H	0.414658	-5.669257	2.426261
C	-1.221730	-4.368196	2.983244
H	-1.841785	-4.658077	2.127065
H	-1.533292	-4.986068	3.833518
C	0.900053	-2.748355	4.212185
H	1.509460	-2.468786	5.077535
C	-0.580868	-2.484188	4.515811
H	-0.891112	-3.055675	5.398092

H	-0.733946	-1.423290	4.743397
C	-1.428265	-2.882411	3.300196
H	-2.485643	-2.689866	3.508841
C	-0.211320	-4.534809	-3.156788
C	-0.874343	-4.944474	-4.280750
C	-2.081178	-2.852614	-4.414142
C	-1.814931	-4.096925	-4.915032
N	0.198793	-2.838325	-1.512852
N	-1.680039	-1.176194	-2.748963
H	0.514891	-5.162405	-2.653978
H	-0.680939	-5.926281	-4.697134
H	-2.324920	-4.443689	-5.806299
H	-2.795406	-2.183247	-4.878642
C	-3.236505	0.952917	-0.696766
C	-3.555444	-0.034532	0.442598
C	-4.135408	0.638835	-1.906204
C	-3.546783	2.387682	-0.214138
H	-3.341325	-1.059248	0.114834
H	-2.905787	0.174360	1.298004
C	-5.031772	0.079477	0.844905
H	-3.930869	1.340971	-2.721528
H	-3.926013	-0.362289	-2.287453
C	-5.614454	0.751507	-1.497832
H	-2.900343	2.640987	0.629370
H	-3.334217	3.107161	-1.013098
C	-5.022981	2.499748	0.193926
H	-5.236622	-0.631221	1.651891
C	-5.915031	-0.243561	-0.367960
C	-5.318883	1.507980	1.327729
H	-6.238273	0.519182	-2.367186
C	-5.910595	2.177852	-1.015723
H	-5.218948	3.520422	0.537081
H	-5.724187	-1.267483	-0.709282
H	-6.973795	-0.185926	-0.090351
H	-4.699090	1.741735	2.199682
H	-6.366254	1.596931	1.638153
H	-6.968220	2.272964	-0.744341
H	-5.718584	2.894538	-1.822732

Zero-point correction= 1.147654 (Hartree/Particle)
Thermal correction to Energy= 1.199059
Thermal correction to Enthalpy= 1.200003
Thermal correction to Gibbs Free Energy= 1.065456
Sum of electronic and zero-point Energies= -3074.414641
Sum of electronic and thermal Energies= -3074.363236
Sum of electronic and thermal Enthalpies= -3074.362292
Sum of electronic and thermal Free Energies= -3074.496839

[(*S*)-Quinox-Ad$_3$]: TS-C$_{linear}$

C	-0.320085	3.944716	1.519061
C	-1.264105	3.157922	2.216357
C	-0.959362	1.450520	0.723733
C	0.082608	2.210261	0.082553
P	-1.481350	-0.163767	-0.023314
P	1.124208	1.423349	-1.245975
Cu	0.319597	-0.698313	-1.316602
B	1.711548	-2.156159	-0.545883
O	3.100652	-2.002261	-0.476991
O	1.311397	-3.112876	0.386215
C	3.633406	-3.152407	0.206082
C	2.456754	-3.525215	1.157112
C	0.093602	-1.599905	-3.104789
H	0.538441	-1.066087	-3.944720
C	1.093768	-2.328528	-2.281532
C	0.822280	2.563077	-2.657872
H	0.799903	3.604615	-2.332574
H	-0.120636	2.299016	-3.134396
H	1.618432	2.428048	-3.392664
C	-1.980272	-1.256385	1.420164
C	-3.017280	0.416190	-0.985250
C	-0.808923	-1.228551	2.428715
H	-0.710157	-0.227768	2.858230
H	0.120396	-1.465383	1.905845
C	-3.278887	-0.896545	2.166688
H	-3.205268	0.120435	2.558370
H	-4.133533	-0.931560	1.486544
C	-2.086914	-2.698797	0.861653
H	-1.150016	-2.965454	0.364732
H	-2.885676	-2.768630	0.119887
C	-3.945606	1.406972	-0.249364
H	-3.393098	2.309729	0.023106
H	-4.326323	0.980240	0.678958
C	-3.836358	-0.819232	-1.413927
H	-4.247870	-1.323467	-0.536328
C	-2.501906	1.126308	-2.257042
H	-1.800807	0.475262	-2.788284
H	-1.952693	2.028984	-1.963726
H	2.106535	-2.288333	-2.680129
C	3.903433	-4.220662	-0.854807
H	4.366969	-5.110032	-0.421232
H	4.581848	-3.806985	-1.604124
H	2.982398	-4.521219	-1.359801
C	4.926983	-2.792530	0.916518
H	5.708274	-2.577143	0.184470

H	5.264402	-3.631817	1.532014
H	4.805637	-1.918244	1.556123
C	2.487692	-2.720063	2.455786
H	3.324334	-3.026648	3.088227
H	1.565964	-2.889908	3.011637
H	2.574722	-1.649683	2.254272
C	2.338281	-5.010783	1.461417
H	1.516777	-5.187109	2.158809
H	3.257898	-5.382408	1.923508
H	2.143870	-5.584632	0.555248
C	-1.151882	-2.369586	-3.490865
H	-1.927772	-1.675841	-3.836940
H	-1.564653	-2.864654	-2.601543
C	-0.923429	-3.424382	-4.580835
H	-0.187384	-4.166036	-4.254153
H	-0.535993	-2.957207	-5.491774
H	-1.846626	-3.955290	-4.837866
H	0.800514	-3.361124	-2.068843
C	-2.352595	-1.859973	4.296056
H	-2.533814	-2.558491	5.121202
H	-2.259015	-0.858311	4.730940
C	-3.525316	-1.897455	3.307712
H	-4.450521	-1.620242	3.823622
C	-3.663341	-3.309768	2.723568
H	-3.875005	-4.030985	3.521364
H	-4.506811	-3.344196	2.023848
C	-1.192742	-3.652882	2.986140
H	-1.349826	-4.375464	3.795690
H	-0.277389	-3.933119	2.459235
C	-1.060003	-2.236476	3.560595
H	-0.218227	-2.196866	4.260590
C	-2.364933	-3.689898	2.000744
H	-2.457765	-4.694270	1.575466
C	-5.919824	0.554050	-1.558855
H	-6.775289	0.838087	-2.182291
H	-6.319935	0.059121	-0.666207
C	-5.124333	1.802941	-1.156490
H	-5.772515	2.489090	-0.601861
C	-4.592979	2.498207	-2.416093
H	-5.425816	2.804576	-3.059542
H	-4.045339	3.406882	-2.140643
C	-4.458244	0.284926	-3.581018
H	-5.284091	0.564940	-4.244994
H	-3.812827	-0.401582	-4.138874
C	-3.668556	1.533151	-3.168132
H	-3.262899	2.024087	-4.058684

C	-5.001005	-0.406455	-2.324823
H	-5.557972	-1.304839	-2.609319
H	-3.189813	-1.532366	-1.930175
C	-0.045400	5.261250	1.957383
C	-0.680289	5.752909	3.064634
C	-1.902292	3.685988	3.363297
C	-1.610941	4.957428	3.775757
N	0.344882	3.439350	0.449311
N	-1.593771	1.919066	1.770552
H	0.676208	5.845830	1.399192
H	-0.471356	6.759569	3.407717
H	-2.098165	5.367719	4.652712
H	-2.618282	3.060877	3.883530
C	2.886265	1.825066	-0.757758
C	3.151429	1.251871	0.649123
C	3.255278	3.322085	-0.776532
C	3.786978	1.080834	-1.772361
H	2.524038	1.765841	1.386667
H	2.892503	0.189632	0.666049
C	4.631704	1.436890	1.015309
H	3.091184	3.738195	-1.776462
H	2.613180	3.874190	-0.089109
C	4.732882	3.505030	-0.394422
H	3.549597	0.013217	-1.765962
H	3.608886	1.461377	-2.785410
C	5.265720	1.282247	-1.406187
H	4.804602	1.020083	2.013481
C	4.977141	2.931757	1.009097
C	5.510538	0.703845	-0.007692
H	4.968101	4.574455	-0.400222
C	5.615491	2.774969	-1.414610
H	5.885097	0.750749	-2.135772
H	4.360467	3.464178	1.742429
H	6.024658	3.078367	1.296707
H	5.265345	-0.358642	-0.013227
H	6.567881	0.805015	0.263972
H	6.674742	2.913933	-1.168888
H	5.457700	3.196449	-2.414254

Zero-point correction= 1.149076 (Hartree/Particle)
Thermal correction to Energy= 1.200211
Thermal correction to Enthalpy= 1.201155
Thermal correction to Gibbs Free Energy= 1.069297
Sum of electronic and zero-point Energies= -3074.413032
Sum of electronic and thermal Energies= -3074.361897
Sum of electronic and thermal Enthalpies= -3074.360953
Sum of electronic and thermal Free Energies= -3074.492811

[(*S*)-Quinox-Ad$_3$]: TS-D$_{linear}$

C	0.590011	-2.722906	3.051017
C	1.629089	-1.863104	3.471597
C	1.203470	-0.471473	1.706110
C	0.178746	-1.373496	1.247239
P	1.425089	1.169028	0.865671
P	-0.758169	-1.016406	-0.321302
Cu	-0.084514	1.091152	-0.828591
B	-1.341561	2.694100	-0.344906
O	-0.864719	3.743836	0.442025
O	-2.735234	2.729628	-0.367198
C	0.474007	1.686510	-2.685902
H	1.485185	2.088339	-2.748658
C	-0.509916	2.612244	-2.105210
H	-0.141348	3.619410	-1.910400
C	-0.161645	-2.436700	-1.428048
C	-2.565248	-1.231685	0.151928
C	1.192846	2.246980	2.329783
C	-3.405271	-0.600543	-0.986820
H	-3.257063	-1.146725	-1.921973
H	-3.082483	0.430679	-1.152839
C	-2.802951	-0.395316	1.432108
H	-2.458349	0.629741	1.262866
H	-2.229625	-0.813778	2.265275
C	-3.054244	-2.670414	0.399188
H	-2.457671	-3.136550	1.186936
H	-2.931966	-3.267743	-0.508461
C	-0.106838	-3.847277	-0.801913
H	0.535044	-3.843779	0.080811
H	-1.092857	-4.171704	-0.468526
C	-1.037880	-2.485480	-2.697418
H	-1.097174	-1.492143	-3.149915
H	-2.056219	-2.790490	-2.440543
C	1.278072	-2.056755	-1.838751
H	1.288376	-1.045636	-2.252963
H	1.915450	-2.041545	-0.947773
H	0.169940	2.104745	2.679081
H	1.889778	1.992116	3.128894
H	1.297273	3.289861	2.030579
C	-1.975648	4.390030	1.081343
C	-3.157689	4.013254	0.134749
C	-1.689067	5.880397	1.181544
H	-0.854431	6.048687	1.865935
H	-2.560875	6.415724	1.569714
H	-1.422117	6.300249	0.211845
C	-2.128491	3.793092	2.481016

H	-2.935072	4.276612	3.037663
H	-1.197243	3.939716	3.031210
H	-2.332438	2.720449	2.428776
C	-4.505863	3.882270	0.822743
H	-5.269528	3.604257	0.092917
H	-4.795474	4.836962	1.272056
H	-4.489025	3.121886	1.603393
C	-3.275203	4.946105	-1.071053
H	-3.646317	5.933028	-0.783604
H	-3.974429	4.508086	-1.786583
H	-2.311201	5.068845	-1.570623
C	0.062200	0.934093	-3.933010
H	-0.948035	0.528405	-3.794970
H	0.717167	0.068836	-4.087125
C	0.080528	1.791617	-5.204083
H	1.079620	2.205240	-5.374316
H	-0.612728	2.633582	-5.111933
H	-0.203234	1.215125	-6.091400
H	-1.475261	2.628462	-2.612988
C	-4.755524	-1.835425	2.052127
H	-4.188960	-2.271330	2.883099
H	-5.813856	-1.848019	2.337300
C	-4.544170	-2.665901	0.779366
H	-4.863843	-3.698156	0.955990
C	-5.358337	-2.061746	-0.372658
H	-5.222933	-2.660566	-1.281138
H	-6.427295	-2.078646	-0.130513
C	-4.294404	-0.394327	1.797873
H	-4.432582	0.203136	2.705653
C	-5.106870	0.214761	0.648596
H	-6.170682	0.246762	0.912592
H	-4.774685	1.237036	0.460547
C	-4.896222	-0.620147	-0.618409
H	-5.465572	-0.183405	-1.445036
C	-0.443634	-4.886931	-3.068442
H	-1.461603	-5.190601	-2.797023
H	-0.066607	-5.624971	-3.785659
C	0.448028	-4.858742	-1.820669
H	0.459107	-5.848846	-1.353464
C	1.874813	-4.460266	-2.218363
H	2.524729	-4.462063	-1.335677
H	2.286891	-5.187494	-2.927476
C	0.960132	-3.084391	-4.101187
H	1.362586	-3.792847	-4.834374
H	0.950570	-2.097568	-4.574675
C	-0.465204	-3.492040	-3.706934

H	-1.105350	-3.500739	-4.594921
C	1.845130	-3.062562	-2.848456
H	2.860595	-2.754253	-3.118058
C	0.294565	-3.884545	3.802907
C	1.024468	-4.169502	4.923936
C	2.369976	-2.179330	4.634978
C	2.070670	-3.311356	5.342054
N	-0.120685	-2.453304	1.927702
N	1.901669	-0.724177	2.786266
H	0.805594	-5.060475	5.501062
H	2.633820	-3.557502	6.234896
H	3.159065	-1.501264	4.937404
H	-0.507649	-4.526142	3.458225
C	3.240890	1.334402	0.456424
C	3.600097	0.236272	-0.561479
C	4.222814	1.267946	1.639352
C	3.384853	2.712535	-0.231990
H	3.506175	-0.747411	-0.086412
H	2.891688	0.269499	-1.394580
C	5.034941	0.425374	-1.067217
H	3.992242	2.052375	2.367960
H	4.123748	0.310977	2.157288
C	5.662549	1.456563	1.128638
H	2.677592	2.785931	-1.063833
H	3.138390	3.512716	0.475443
C	4.821852	2.900067	-0.739205
H	5.267093	-0.366653	-1.786336
C	6.004526	0.350552	0.120136
C	5.154949	1.794265	-1.750267
H	6.349872	1.402800	1.979198
C	5.794155	2.825451	0.446120
H	4.903445	3.878297	-1.223359
H	5.931067	-0.631443	0.601241
H	7.037516	0.466454	-0.227422
H	4.467731	1.847267	-2.602163
H	6.169311	1.934477	-2.140928
H	6.822903	2.975869	0.099296
H	5.573520	3.624779	1.162909

Zero-point correction= 1.147333 (Hartree/Particle)
Thermal correction to Energy= 1.198969
Thermal correction to Enthalpy= 1.199913
Thermal correction to Gibbs Free Energy= 1.065714
Sum of electronic and zero-point Energies= -3074.414844
Sum of electronic and thermal Energies= -3074.363208
Sum of electronic and thermal Enthalpies= -3074.362264
Sum of electronic and thermal Free Energies= -3074.496463

[(*S*)-Quinox-Ad$_3$]: III-A$_{branch}$

C	-3.012497	3.408908	0.025853
C	-3.550860	2.454314	0.919838
C	-1.980769	0.929893	0.256657
C	-1.365455	1.939178	-0.565511
P	-1.318153	-0.801263	0.147281
P	0.269997	1.580289	-1.385205
Cu	0.568861	-0.692657	-1.180892
B	3.680740	-1.956843	-1.382347
O	4.168764	-0.833619	-0.738889
O	3.907408	-3.094625	-0.638888
C	4.483195	-1.203188	0.612640
C	4.717401	-2.748680	0.492918
C	1.406547	-2.151028	-2.225056
H	1.362446	-3.104679	-1.681660
H	0.758983	-2.303887	-3.104187
C	2.869331	-1.930779	-2.710066
C	-1.248366	-1.427931	1.909020
C	-2.655150	-1.608169	-0.904155
C	-0.464933	-0.354320	2.702475
H	-1.022373	0.586876	2.717820
H	0.493100	-0.160625	2.207556
C	-2.587730	-1.706365	2.612448
H	-3.214037	-0.811204	2.590965
H	-3.122736	-2.500079	2.081851
C	-0.403732	-2.726077	1.912490
H	0.547854	-2.555669	1.399551
H	-0.916164	-3.516128	1.357826
C	-4.119769	-1.425191	-0.463006
H	-4.366241	-0.362961	-0.391635
H	-4.281083	-1.852172	0.528253
C	-2.339634	-3.116359	-1.005545
H	-2.479043	-3.589505	-0.027811
H	-1.293724	-3.260305	-1.298605
C	-2.490822	-0.979989	-2.313132
H	-1.453828	-1.093412	-2.650886
H	-2.711242	0.094044	-2.271564
C	5.689537	-0.401873	1.074970
H	6.001956	-0.713182	2.076223
H	5.434406	0.659612	1.110882
H	6.529444	-0.521485	0.390870
C	3.259963	-0.858159	1.460558
H	3.044414	0.204484	1.346660
H	3.426580	-1.065450	2.520435
H	2.383297	-1.414226	1.118930
C	4.251908	-3.555719	1.696723

H	4.790630	-3.253031	2.599861
H	4.445665	-4.615944	1.521170
H	3.181549	-3.432782	1.866858
C	6.160842	-3.109740	0.143528
H	6.204611	-4.170666	-0.110713
H	6.839407	-2.923564	0.980030
H	6.506480	-2.540549	-0.722444
C	-4.886418	-1.471338	-2.855208
H	-5.134877	-0.404613	-2.810240
H	-5.572516	-1.934421	-3.573360
C	-5.060404	-2.108762	-1.471253
H	-6.092581	-1.975100	-1.131819
C	-4.732700	-3.606274	-1.553142
H	-4.874686	-4.076501	-0.573035
H	-5.416234	-4.102609	-2.251262
C	-3.096422	-3.147362	-3.399496
H	-3.745944	-3.640960	-4.131121
H	-2.062917	-3.278819	-3.737495
C	-3.435137	-1.653079	-3.317201
H	-3.295451	-1.187919	-4.298054
C	-3.280909	-3.786261	-2.017531
H	-3.039106	-4.852190	-2.070606
C	-2.333736	-2.164435	4.058496
H	-3.297204	-2.348719	4.544371
C	-1.506404	-3.457355	4.045120
H	-1.340896	-3.810170	5.069434
H	-2.052868	-4.246891	3.516508
C	-1.570677	-1.075302	4.824546
H	-1.415293	-1.385023	5.864163
H	-2.156933	-0.149550	4.845652
C	-0.159964	-3.196528	3.355080
H	0.427428	-4.119674	3.328609
C	0.606640	-2.116130	4.127521
H	1.577632	-1.936532	3.654502
H	0.800258	-2.451198	5.152748
C	-0.219257	-0.823731	4.141943
H	0.326724	-0.041371	4.678982
C	-4.674061	2.794187	1.710956
C	-5.238029	4.034163	1.590096
C	-3.620577	4.681384	-0.088258
C	-4.711028	4.982810	0.679990
N	-3.029458	1.203112	0.995362
N	-1.901820	3.126663	-0.701392
H	-5.064329	2.046770	2.391379
H	-6.101006	4.297873	2.190537
H	-5.179698	5.956515	0.597353

H	-3.193303	5.390889	-0.786786
C	3.058285	-0.621598	-3.490407
H	2.900416	0.220119	-2.807224
H	4.097382	-0.546745	-3.835228
C	2.121027	-0.470236	-4.685535
H	2.215839	-1.316795	-5.374132
H	2.335769	0.444708	-5.247788
H	1.079044	-0.425852	-4.355993
H	3.182953	-2.764873	-3.358808
C	1.394724	2.814715	-0.534347
C	1.235817	2.636978	0.990031
C	1.124858	4.285880	-0.901055
C	2.847945	2.461386	-0.922611
H	0.212970	2.880598	1.297612
H	1.410837	1.590776	1.259605
C	2.216613	3.550975	1.736678
H	1.257433	4.432420	-1.978154
H	0.091756	4.552094	-0.663746
C	2.102772	5.200220	-0.145103
H	3.066293	1.412052	-0.701456
H	2.986296	2.585279	-2.003457
C	3.827312	3.382018	-0.176352
H	2.085182	3.406899	2.813873
C	1.921078	5.010748	1.367717
C	3.656024	3.201093	1.338386
H	1.893139	6.241088	-0.411599
C	3.543128	4.846039	-0.540357
H	4.848793	3.117089	-0.465981
H	0.896433	5.270950	1.657454
H	2.593742	5.680248	1.915396
H	3.882929	2.170134	1.625930
H	4.360201	3.846120	1.876060
H	4.248744	5.506163	-0.023146
H	3.685019	5.000172	-1.615953
C	-0.014855	2.271133	-3.058143
H	-0.504004	3.245050	-3.015496
H	-0.636198	1.569170	-3.617398
H	0.945814	2.348860	-3.571406

Zero-point correction= 1.148932 (Hartree/Particle)
Thermal correction to Energy= 1.200848
Thermal correction to Enthalpy= 1.201793
Thermal correction to Gibbs Free Energy= 1.066113
Sum of electronic and zero-point Energies= -3074.464472
Sum of electronic and thermal Energies= -3074.412556
Sum of electronic and thermal Enthalpies= -3074.411612
Sum of electronic and thermal Free Energies= -3074.547291

[(*S*)-Quinox-Ad$_3$]: III-B$_{branch}$

C	-4.047414	-0.823583	-1.623521
C	-3.930063	0.441364	-2.245635
C	-1.853708	0.709619	-1.331475
C	-1.947057	-0.603151	-0.745574
P	-0.342487	1.752484	-1.014282
P	-0.478060	-1.257603	0.183073
Cu	0.856878	0.594018	0.544640
B	3.933254	0.304920	0.686817
O	4.768692	1.393873	0.573278
O	3.681688	-0.265328	-0.553006
C	4.811013	1.769386	-0.811609
C	4.495102	0.413950	-1.528443
C	2.061221	0.871695	2.088658
H	2.462968	1.888966	2.201010
H	1.475310	0.683983	3.001656
C	3.259873	-0.121651	2.022850
C	-1.177514	-2.044268	1.730517
C	0.221561	-2.491460	-1.046375
C	0.067252	2.260081	-2.729502
C	1.383209	-3.243665	-0.360954
H	0.995728	-3.875007	0.444929
H	2.082058	-2.525267	0.080917
C	0.815188	-1.629915	-2.189686
H	1.519817	-0.904305	-1.772749
H	0.012913	-1.075674	-2.694139
C	-0.757389	-3.509106	-1.662247
H	-1.579777	-2.989972	-2.161778
H	-1.205145	-4.134321	-0.888186
C	6.177337	2.356397	-1.126583
H	6.302359	3.298902	-0.589547
H	6.275220	2.556971	-2.197858
H	6.977779	1.682784	-0.820297
C	3.719111	2.821189	-1.020021
H	3.721261	3.210778	-2.041856
H	3.896876	3.648464	-0.329845
H	2.733570	2.406005	-0.792578
C	5.738546	-0.444323	-1.755737
H	6.387809	-0.016462	-2.523761
H	5.430333	-1.440363	-2.081352
H	6.311337	-0.550995	-0.831988
C	3.711150	0.557446	-2.823248
H	3.554568	-0.423964	-3.275623
H	4.261488	1.177202	-3.537176
H	2.736889	1.011870	-2.642429
C	4.189269	-0.107595	3.247711

H	4.586975	0.905005	3.381324
H	5.056029	-0.756763	3.063354
C	3.498675	-0.562953	4.531576
H	4.197341	-0.598133	5.372674
H	3.071040	-1.564467	4.406298
H	2.683499	0.112838	4.802617
H	2.859527	-1.140299	1.911265
H	0.838112	3.032820	-2.699835
H	-0.814632	2.629136	-3.255010
H	0.469003	1.394829	-3.260612
C	-1.909614	-3.387283	1.572022
H	-2.729900	-3.283877	0.856830
H	-1.218242	-4.139772	1.179976
C	0.002298	-2.227881	2.719410
H	0.536731	-1.281901	2.839044
H	0.722944	-2.949946	2.325219
C	-2.143992	-1.001167	2.342987
H	-1.623790	-0.042477	2.458716
H	-2.994300	-0.839004	1.674869
C	0.556603	-3.534172	-3.802696
H	-0.254561	-3.013066	-4.324587
H	1.065721	-4.164005	-4.541095
C	-0.017039	-4.400793	-2.674835
H	-0.727023	-5.123661	-3.089542
C	1.126225	-5.145268	-1.971168
H	1.641371	-5.800609	-2.682682
H	0.725452	-5.783955	-1.175461
C	2.687479	-3.250066	-2.500792
H	3.244246	-3.865760	-3.216432
H	3.381223	-2.521639	-2.069916
C	2.112740	-4.128492	-1.382049
H	2.927396	-4.653021	-0.873222
C	1.539661	-2.518500	-3.209220
H	1.939483	-1.884566	-4.007781
C	-1.250901	-4.055587	3.899224
H	-0.563073	-4.811382	3.502381
H	-1.610019	-4.422506	4.867573
C	-2.432519	-3.862816	2.937764
H	-2.956535	-4.814562	2.802745
C	-3.398152	-2.819281	3.515552
H	-4.249476	-2.682088	2.838817
H	-3.798327	-3.167739	4.474485
C	-0.516528	-2.719803	4.078264
H	0.336516	-2.851484	4.750881
C	-1.478206	-1.679160	4.664576
H	-1.839950	-2.008287	5.645435

H	-0.954821	-0.727993	4.811439
C	-2.659021	-1.486920	3.704594
H	-3.347532	-0.737498	4.108132
N	-3.026264	-1.334850	-0.889901
N	-2.812360	1.193143	-2.082060
C	-5.236881	-1.573424	-1.785363
C	-6.256955	-1.071108	-2.545071
C	-4.996864	0.935276	-3.033155
C	-6.135220	0.191406	-3.175332
H	-5.302482	-2.538176	-1.296769
H	-7.170727	-1.640157	-2.671084
H	-6.956959	0.566799	-3.774088
H	-4.878782	1.905181	-3.501272
C	-1.011361	3.289925	-0.191933
C	-1.742496	2.850069	1.094886
C	-1.952881	4.156539	-1.044889
C	0.222817	4.130993	0.212102
H	-2.617183	2.238801	0.841872
H	-1.072669	2.231291	1.705006
C	-2.186146	4.083645	1.893287
H	-1.439862	4.486124	-1.955229
H	-2.822471	3.574060	-1.359504
C	-2.396295	5.388539	-0.237728
H	0.904705	3.519633	0.812336
H	0.771335	4.449095	-0.683412
C	-0.222609	5.366108	1.007675
H	-2.706117	3.753165	2.797821
C	-3.130014	4.934632	1.032338
C	-0.951930	4.910903	2.279574
H	-3.069067	5.992738	-0.854873
C	-1.165766	6.220303	0.150055
H	0.662633	5.948960	1.280263
H	-4.020403	4.354173	0.764136
H	-3.471747	5.807450	1.600135
H	-0.279206	4.310131	2.901042
H	-1.254481	5.782000	2.871707
H	-1.474856	7.113433	0.704905
H	-0.646443	6.564337	-0.751909

Zero-point correction= 1.148004 (Hartree/Particle)
Thermal correction to Energy= 1.200222
Thermal correction to Enthalpy= 1.201166
Thermal correction to Gibbs Free Energy= 1.064009
Sum of electronic and zero-point Energies= -3074.467960
Sum of electronic and thermal Energies= -3074.415743
Sum of electronic and thermal Enthalpies= -3074.414798
Sum of electronic and thermal Free Energies= -3074.551955

[(*S*)-Quinox-Ad$_3$]: III-C$_{branch}$

C	-3.314070	3.283366	0.310556
C	-3.634750	2.242112	1.212572
C	-2.019807	0.918000	0.281556
C	-1.642457	2.004679	-0.579514
P	-1.163910	-0.722967	0.089906
P	-0.159202	1.798060	-1.679052
Cu	0.581389	-0.414180	-1.391450
B	3.642204	-2.128562	-1.018869
O	3.411786	-2.318121	0.330599
O	4.817175	-1.439264	-1.233987
C	4.325767	-1.471983	1.047729
C	5.496933	-1.305142	0.021977
C	1.932564	-1.252812	-2.567732
H	1.395683	-1.434468	-3.513803
H	2.687978	-0.500711	-2.838508
C	2.677954	-2.556569	-2.159826
C	-0.784666	2.532335	-3.239577
H	-1.334183	3.457320	-3.059313
H	-1.439473	1.805833	-3.724423
H	0.060066	2.721590	-3.904945
C	-0.918708	-1.327166	1.851725
C	-2.500170	-1.688690	-0.833044
C	-0.334213	-0.147192	2.665272
H	-1.064444	0.662805	2.729418
H	0.552720	0.250390	2.159482
C	-2.168752	-1.857184	2.580657
H	-2.934418	-1.078465	2.616726
H	-2.585949	-2.710695	2.039771
C	0.153520	-2.442666	1.799531
H	1.050476	-2.090741	1.280485
H	-0.216002	-3.298607	1.226604
C	-3.945155	-1.547408	-0.315021
H	-4.251934	-0.498799	-0.327811
H	-4.024690	-1.884869	0.719127
C	-2.104186	-3.180366	-0.848410
H	-2.136712	-3.592021	0.164728
H	-1.079068	-3.287772	-1.211181
C	-2.460646	-1.166048	-2.291636
H	-1.442539	-1.250914	-2.691004
H	-2.734965	-0.103487	-2.314840
H	3.285952	-2.913596	-3.008005
C	1.735740	-3.694794	-1.750834
H	2.316895	-4.557691	-1.401734
H	1.137364	-3.359632	-0.895787
C	3.592061	-0.157537	1.304646

H	4.189931	0.538947	1.898013
H	2.666429	-0.367226	1.841535
H	3.324719	0.316038	0.357123
C	4.707156	-2.144230	2.355504
H	3.824071	-2.248562	2.988454
H	5.447412	-1.546474	2.895670
H	5.117191	-3.139246	2.184034
C	6.526418	-2.431787	0.106170
H	7.131105	-2.358178	1.013773
H	7.188245	-2.367994	-0.759863
H	6.038469	-3.408999	0.087774
C	6.195568	0.045886	0.063520
H	6.996815	0.064262	-0.678233
H	6.635649	0.226756	1.048828
H	5.504546	0.856242	-0.170301
C	0.812347	-4.142399	-2.880179
H	0.143271	-4.950288	-2.564867
H	1.390334	-4.499501	-3.738894
H	0.194122	-3.308982	-3.224242
C	-4.856700	-1.825701	-2.635108
H	-5.168426	-0.774865	-2.652853
H	-5.554772	-2.382222	-3.270520
C	-4.903892	-2.362928	-1.199317
H	-5.917913	-2.258616	-0.800052
C	-4.493823	-3.841895	-1.188278
H	-4.543040	-4.238211	-0.167331
H	-5.190075	-4.429782	-1.796972
C	-3.018679	-3.441375	-3.174963
H	-3.695971	-4.019571	-3.813658
H	-2.010850	-3.555228	-3.584354
C	-3.429066	-1.963179	-3.177786
H	-3.378090	-1.567254	-4.196945
C	-3.068176	-3.978330	-1.738883
H	-2.764223	-5.029685	-1.728971
C	-1.798254	-2.310658	4.001787
H	-2.700170	-2.684973	4.496791
C	-0.750013	-3.429845	3.924355
H	-0.500512	-3.782669	4.931585
H	-1.157317	-4.285789	3.374148
C	-1.234250	-1.120348	4.788413
H	-0.996667	-1.422713	5.814633
H	-1.984435	-0.323571	4.849127
C	0.508383	-2.902046	3.221688
H	1.259872	-3.694374	3.153143
C	1.071706	-1.723691	4.025255
H	1.988728	-1.351328	3.561766

H	1.333525	-2.049281	5.038727
C	0.028218	-0.603518	4.086556
H	0.434066	0.251811	4.636281
C	-4.665534	2.433226	2.163476
C	-5.346999	3.618158	2.195132
C	-4.041639	4.496607	0.356929
C	-5.035215	4.656042	1.282845
N	-2.983680	1.053799	1.160643
N	-2.300756	3.138694	-0.580172
H	-4.889464	1.620933	2.844605
H	-6.136572	3.769756	2.921975
H	-5.590988	5.585543	1.326006
H	-3.778885	5.275759	-0.348541
C	1.034527	3.066620	-0.993964
C	1.240321	2.777906	0.507783
C	0.591437	4.529064	-1.174204
C	2.384081	2.853631	-1.717863
H	0.297824	2.911652	1.051424
H	1.547849	1.735662	0.645855
C	2.301922	3.725847	1.085421
H	0.458012	4.750182	-2.238445
H	-0.373976	4.693807	-0.688946
C	1.652502	5.474090	-0.589608
H	2.709068	1.813357	-1.606290
H	2.267324	3.034740	-2.792967
C	3.441853	3.810656	-1.145510
H	2.432099	3.507291	2.150166
C	1.834929	5.177303	0.905895
C	3.634069	3.524963	0.349797
H	1.317957	6.508075	-0.720988
C	2.981856	5.263482	-1.327457
H	4.386565	3.650758	-1.674095
H	0.889003	5.336783	1.436106
H	2.570395	5.864611	1.338885
H	3.994059	2.502823	0.494254
H	4.394215	4.196772	0.764433
H	3.742749	5.950178	-0.939632
H	2.856641	5.487707	-2.392955

Zero-point correction= 1.148899 (Hartree/Particle)
Thermal correction to Energy= 1.200830
Thermal correction to Enthalpy= 1.201774
Thermal correction to Gibbs Free Energy= 1.066753
Sum of electronic and zero-point Energies= -3074.465107
Sum of electronic and thermal Energies= -3074.413176
Sum of electronic and thermal Enthalpies= -3074.412232
Sum of electronic and thermal Free Energies= -3074.547252

[(*S*)-Quinox-Ad$_3$]: III-D$_{branch}$

C	-4.027413	-0.013825	-1.904046
C	-3.631526	1.219998	-2.469250
C	-1.621162	1.081343	-1.389262
C	-2.017588	-0.181306	-0.823948
P	0.082842	1.749097	-1.044663
P	-0.789122	-1.118294	0.210050
Cu	0.962955	0.329172	0.590589
B	4.150324	-0.155195	0.901846
O	4.024152	-1.101076	-0.100262
O	4.963423	0.884946	0.508456
C	2.199649	0.743146	2.090083
H	2.594966	1.763912	1.991772
H	1.615160	0.761405	3.024076
C	3.417475	-0.213871	2.273048
H	4.089236	0.174650	3.055848
C	3.014763	-1.640512	2.670007
H	3.881902	-2.309002	2.599815
H	2.284828	-2.012994	1.942966
C	-1.742806	-1.557291	1.763210
C	-0.383598	-2.584500	-0.891990
C	0.600966	2.126988	-2.767877
C	-0.207208	3.417193	-0.256375
C	0.962773	-3.173450	-0.406992
H	0.860182	-3.567119	0.608850
H	1.729271	-2.392120	-0.366336
C	-0.169421	-2.025471	-2.319999
H	0.566307	-1.213163	-2.290339
H	-1.102579	-1.607283	-2.709328
C	-1.436503	-3.703678	-0.951216
H	-2.401195	-3.295813	-1.268943
H	-1.573336	-4.136398	0.044241
C	-1.052498	4.409455	-1.071690
H	-2.042830	3.992510	-1.267677
H	-0.584324	4.592229	-2.045128
C	1.194233	4.025210	-0.016087
H	1.695576	4.205209	-0.974583
H	1.810998	3.321495	0.551624
C	-0.877080	3.179340	1.114099
H	-0.277356	2.473154	1.700169
H	-1.868608	2.731436	0.972496
C	2.435294	-1.742674	4.079527
H	3.177589	-1.443627	4.826464
H	2.117214	-2.764461	4.311139
H	1.568595	-1.088129	4.202331
C	-3.128865	-2.216881	1.630036

H	-3.787977	-1.592941	1.025222
H	-3.050323	-3.178591	1.120060
C	-0.834539	-2.459870	2.624014
H	0.157047	-2.008933	2.711153
H	-0.711383	-3.434964	2.140037
C	-1.912122	-0.200424	2.492838
H	-0.935403	0.284735	2.597543
H	-2.547359	0.465203	1.895339
H	0.802736	1.184800	-3.281945
H	-0.174828	2.673535	-3.305912
H	1.526117	2.705745	-2.749360
C	4.582233	-0.548081	-1.304066
C	5.563692	0.541962	-0.748652
C	3.425277	0.053107	-2.098882
H	2.690416	-0.730194	-2.296447
H	3.758522	0.466559	-3.054185
H	2.929391	0.837253	-1.522174
C	5.246380	-1.660708	-2.099685
H	5.749220	-1.256204	-2.983123
H	4.491105	-2.374602	-2.434844
H	5.974905	-2.202234	-1.496614
C	6.958257	-0.007157	-0.448078
H	7.514353	0.742482	0.118307
H	7.509380	-0.232536	-1.364765
H	6.899530	-0.913803	0.158413
C	5.670569	1.797913	-1.600987
H	6.044452	1.558427	-2.601072
H	6.368583	2.495862	-1.133698
H	4.706099	2.297650	-1.693858
C	0.390803	5.100201	2.099385
H	0.998008	4.410750	2.695614
H	0.313554	6.038483	2.660353
C	1.064269	5.353351	0.743501
H	2.063764	5.769751	0.901609
C	0.216296	6.334305	-0.078005
H	0.134409	7.292910	0.446779
H	0.700397	6.533641	-1.041174
C	-1.178668	5.736479	-0.305531
H	-1.785474	6.429442	-0.897331
C	-1.856669	5.485198	1.048894
H	-1.974793	6.430771	1.590158
H	-2.860240	5.072146	0.894645
C	-1.007352	4.507498	1.872579
H	-1.488790	4.319743	2.837354
C	-0.767119	-4.234830	-3.317286
H	-0.459806	-5.027048	-4.009082

H	-1.709306	-3.821289	-3.694967
C	-0.971283	-4.812831	-1.910316
H	-1.735917	-5.595370	-1.941879
C	0.351795	-5.401391	-1.399369
H	0.209744	-5.833347	-0.402135
H	0.681508	-6.212715	-2.058376
C	0.304176	-3.136985	-3.265564
H	0.447597	-2.711935	-4.264316
C	1.417417	-4.297951	-1.349060
H	2.360240	-4.707188	-0.974221
C	-3.911434	-1.075132	3.724364
H	-4.375791	-1.212410	4.707564
H	-4.577319	-0.432306	3.137111
C	-3.741068	-2.429929	3.025377
H	-4.719001	-2.907920	2.907088
C	-2.825685	-3.330583	3.866294
H	-3.271044	-3.503381	4.852655
H	-2.716486	-4.309237	3.384457
C	-2.538191	-0.409152	3.877132
H	-2.649010	0.564515	4.364749
C	-1.616308	-1.305447	4.714058
H	-2.038616	-1.449212	5.715014
H	-0.639769	-0.826110	4.838842
C	-1.452234	-2.661959	4.015433
H	-0.787455	-3.300231	4.605578
C	1.624968	-3.726015	-2.756545
H	1.974745	-4.509255	-3.438618
H	2.396084	-2.949661	-2.732352
N	-3.188165	-0.707383	-1.094403
N	-2.407723	1.741278	-2.203908
C	-5.308349	-0.539225	-2.198150
C	-6.151698	0.154329	-3.021268
C	-4.519911	1.914961	-3.324135
C	-5.754428	1.389888	-3.588538
H	-5.585635	-1.487030	-1.752472
H	-7.135193	-0.241665	-3.246218
H	-6.440316	1.920559	-4.238629
H	-4.190090	2.856777	-3.745995

Zero-point correction= 1.150072 (Hartree/Particle)
Thermal correction to Energy= 1.201861
Thermal correction to Enthalpy= 1.202805
Thermal correction to Gibbs Free Energy= 1.067794
Sum of electronic and zero-point Energies= -3074.463443
Sum of electronic and thermal Energies= -3074.411653
Sum of electronic and thermal Enthalpies= -3074.410709
Sum of electronic and thermal Free Energies= -3074.545721

[(*S*)-Quinox-Ad$_3$]: III-A$_{linear}$

C	2.915470	3.506852	-0.687349
C	3.356692	2.449211	-1.516390
C	1.923902	1.011212	-0.466305
C	1.428022	2.101955	0.330741
P	1.258997	-0.688850	-0.135842
P	0.018237	1.793309	1.510063
Cu	-0.475315	-0.473956	1.370464
B	-3.668166	-1.553143	1.093971
O	-4.287660	-0.560288	0.356718
O	-3.673538	-2.752434	0.414690
C	-1.542589	-1.705399	2.503597
H	-1.435391	-2.730713	2.116954
C	-3.058389	-1.383655	2.512699
H	-3.615515	-2.053067	3.191372
C	0.631002	2.669505	2.999784
H	1.065689	3.636948	2.744530
H	1.384351	2.045926	3.484750
H	-0.197325	2.802057	3.698260
C	-4.582463	-1.081280	-0.952288
C	-4.553652	-2.631208	-0.710763
C	-3.476511	-0.607827	-1.891720
H	-3.461767	0.482916	-1.915132
H	-3.638393	-0.965979	-2.911039
H	-2.501064	-0.955835	-1.544776
C	-5.923810	-0.533219	-1.416789
H	-6.219260	-0.994972	-2.363521
H	-5.847722	0.545210	-1.572561
H	-6.704755	-0.714678	-0.678657
C	-3.992095	-3.449630	-1.863230
H	-4.569252	-3.287515	-2.778433
H	-4.039292	-4.511656	-1.613261
H	-2.949332	-3.197090	-2.053822
C	-5.909313	-3.192689	-0.280103
H	-5.767622	-4.219687	0.061942
H	-6.628370	-3.194255	-1.103249
H	-6.326909	-2.616728	0.548886
C	2.662980	-1.402322	0.906266
C	1.109038	-1.506049	-1.818418
C	0.435180	-0.491967	-2.774640
H	1.090772	0.368424	-2.927488
H	-0.496223	-0.119754	-2.331531
C	2.371227	-2.898729	1.152378
H	2.430976	-3.452606	0.210171
H	1.353085	-3.018106	1.540228
C	2.417435	-2.002287	-2.461544

H	3.112855	-1.166702	-2.576269
H	2.891782	-2.745809	-1.814897
C	0.145660	-2.707307	-1.644358
H	0.576834	-3.447431	-0.963815
H	-0.787319	-2.376223	-1.176106
C	4.095610	-1.234307	0.363017
H	4.208788	-1.724983	-0.603657
H	4.316981	-0.175161	0.206376
C	2.585182	-0.666364	2.268581
H	1.575022	-0.751518	2.684569
H	2.794936	0.400997	2.126280
C	-0.964286	-1.705776	3.930919
H	-1.084473	-0.707625	4.376992
H	0.119707	-1.879711	3.885994
C	-1.555651	-2.749367	4.887529
H	-1.455705	-3.754211	4.462932
H	-2.618841	-2.574807	5.073718
H	-1.047286	-2.739820	5.857827
H	-3.231184	-0.364814	2.881326
C	1.471300	-1.625457	-4.753721
H	1.284835	-2.072105	-5.736911
H	2.145315	-0.774586	-4.904549
C	2.126569	-2.653316	-3.822388
H	3.070451	-2.996263	-4.258224
C	1.186010	-3.850409	-3.622104
H	0.993048	-4.343239	-4.581660
H	1.657841	-4.591302	-2.966469
C	-0.786148	-2.349234	-3.950848
H	-1.744515	-2.016328	-3.545388
H	-0.990050	-2.818059	-4.920288
C	0.150401	-1.149511	-4.133900
H	-0.319765	-0.409795	-4.789743
C	-0.132294	-3.364387	-3.005057
H	-0.805881	-4.213746	-2.853188
C	5.108529	-1.829662	1.356471
H	6.114318	-1.712155	0.940502
C	4.807568	-3.319816	1.567985
H	4.891418	-3.857658	0.616395
H	5.542478	-3.757780	2.252786
C	3.392664	-3.480751	2.141252
H	3.172700	-4.542224	2.290362
C	3.291556	-2.739968	3.480821
H	4.000014	-3.165057	4.200737
H	2.287910	-2.859913	3.902238
C	3.597424	-1.252699	3.261269
H	3.509674	-0.715353	4.210770

C	5.014171	-1.091463	2.697642
H	5.244190	-0.028954	2.557198
H	5.749987	-1.492531	3.403759
C	4.349984	2.694075	-2.493905
C	4.879827	3.948114	-2.622627
C	3.485853	4.793510	-0.834912
C	4.446764	5.004666	-1.784944
N	2.857363	1.195903	-1.368514
N	1.940724	3.304099	0.235828
H	4.668030	1.866534	-3.116780
H	5.641286	4.140935	-3.369385
H	4.885011	5.988784	-1.903612
H	3.134307	5.584643	-0.183451
C	-1.347353	2.871065	0.838004
C	-1.620125	2.423601	-0.612543
C	-1.072206	4.384130	0.865933
C	-2.606609	2.566201	1.682426
H	-0.742193	2.616856	-1.240946
H	-1.806422	1.343721	-0.630717
C	-2.831310	3.182149	-1.173866
H	-0.892421	4.713018	1.895401
H	-0.170575	4.616881	0.293391
C	-2.282104	5.141294	0.293772
H	-2.818017	1.493029	1.660100
H	-2.437456	2.844998	2.729498
C	-3.810246	3.340719	1.126498
H	-3.008036	2.856963	-2.205049
C	-2.536248	4.689012	-1.151784
C	-4.068818	2.884949	-0.315008
H	-2.068828	6.214973	0.309835
C	-3.520902	4.846651	1.151047
H	-4.686582	3.117068	1.741998
H	-1.660445	4.911031	-1.772670
H	-3.382564	5.242050	-1.574442
H	-4.285989	1.814125	-0.310565
H	-4.942319	3.404968	-0.725306
H	-4.385100	5.400642	0.767034
H	-3.352410	5.183216	2.180609

Zero-point correction= 1.148287 (Hartree/Particle)
Thermal correction to Energy= 1.200525
Thermal correction to Enthalpy= 1.201469
Thermal correction to Gibbs Free Energy= 1.065130
Sum of electronic and zero-point Energies= -3074.462263
Sum of electronic and thermal Energies= -3074.410024
Sum of electronic and thermal Enthalpies= -3074.409080
Sum of electronic and thermal Free Energies= -3074.545419

[(*S*)-Quinox-Ad$_3$]: III-B$_{linear}$

C	-3.759325	0.848733	-2.133263
C	-2.957551	1.764530	-2.851864
C	-1.144917	1.187134	-1.582759
C	-1.953697	0.232117	-0.869600
P	0.672041	1.340870	-1.190499
P	-1.136344	-0.849369	0.401261
Cu	1.006005	-0.028554	0.638645
B	3.531351	-1.807817	0.935614
O	2.946724	-2.329429	-0.209996
O	4.810024	-1.364417	0.679713
C	3.942362	-2.360558	-1.248264
C	4.978090	-1.298448	-0.744892
C	2.148589	-0.205283	2.263298
H	1.458392	-0.161678	3.124147
C	2.792605	-1.630985	2.289515
C	-2.199183	-0.652410	1.932377
C	-1.199782	-2.541600	-0.400762
C	1.332536	1.283121	-2.904095
C	-0.304301	-3.494933	0.426389
H	-0.724601	-3.642968	1.423665
H	0.689805	-3.056694	0.547354
C	-0.531803	-2.363978	-1.788279
H	0.458678	-1.912732	-1.658826
H	-1.130666	-1.693199	-2.414776
C	-2.586702	-3.178156	-0.583843
H	-3.243234	-2.506172	-1.144244
H	-3.048451	-3.343799	0.394114
C	3.285237	-2.020458	-2.575993
H	2.585853	-2.809099	-2.863007
H	4.036261	-1.927470	-3.365927
H	2.734220	-1.084031	-2.503862
C	4.514101	-3.777267	-1.286344
H	5.248317	-3.891556	-2.087785
H	3.702616	-4.487407	-1.458668
H	4.990562	-4.030369	-0.336779
C	4.620744	0.128950	-1.165504
H	4.765256	0.283615	-2.238316
H	5.264018	0.824485	-0.622549
H	3.584715	0.359561	-0.903154
C	6.426358	-1.603113	-1.090977
H	7.069012	-0.811051	-0.701127
H	6.562750	-1.652792	-2.175531
H	6.749972	-2.546912	-0.652129
H	1.995750	-2.377774	2.379173
H	2.408631	1.463226	-2.886534

H	0.835352	2.016217	-3.540256
H	1.154529	0.285928	-3.311491
C	-3.721130	-0.857699	1.811022
H	-4.132015	-0.194595	1.048137
H	-3.944790	-1.879137	1.494261
C	-1.649732	-1.608435	3.015002
H	-0.562795	-1.497982	3.098496
H	-1.858351	-2.644551	2.732021
C	-1.932536	0.803440	2.390977
H	-0.853439	0.964931	2.484782
H	-2.307015	1.505789	1.636396
C	3.179195	0.911823	2.486145
H	2.648322	1.872538	2.470741
H	3.887558	0.949115	1.649120
C	3.981663	0.835366	3.790484
H	4.656421	-0.024813	3.798055
H	3.310099	0.741223	4.651458
H	4.594057	1.731688	3.937165
H	3.465015	-1.777963	3.146534
C	-1.794980	-4.330538	-2.677960
H	-2.415339	-3.671977	-3.296929
H	-1.719468	-5.290972	-3.200242
C	-2.449425	-4.531259	-1.303681
H	-3.445901	-4.966006	-1.432333
C	-1.580499	-5.471394	-0.456064
H	-2.049022	-5.633996	0.521548
H	-1.499610	-6.450205	-0.942581
C	0.471385	-4.655997	-1.646466
H	1.463438	-4.219051	-1.505037
H	0.595870	-5.619423	-2.154259
C	-0.187236	-4.854611	-0.277223
H	0.435133	-5.512165	0.337425
C	-0.398851	-3.719937	-2.494015
H	0.069150	-3.563255	-3.472035
C	-3.837209	-1.543234	4.228585
H	-4.041916	-2.581945	3.944102
H	-4.331793	-1.368900	5.190955
C	-4.395341	-0.585806	3.167189
H	-5.473246	-0.743807	3.058602
C	-4.127804	0.862881	3.596465
H	-4.538704	1.556903	2.854137
H	-4.627901	1.071919	4.549005
C	-2.324055	-1.325408	4.365481
H	-1.917029	-2.014300	5.112072
C	-2.046738	0.121628	4.791581
H	-2.505801	0.320003	5.766738

H	-0.968346	0.280720	4.899586
C	-2.615497	1.078874	3.735955
H	-2.413188	2.114272	4.028035
C	-5.132533	0.721709	-2.450549
C	-5.669887	1.485037	-3.449683
C	-3.535482	2.537137	-3.887315
C	-4.865079	2.397254	-4.175020
N	-3.224637	0.077054	-1.153764
N	-1.640605	1.908475	-2.558066
H	-5.724614	0.014388	-1.882203
H	-6.721641	1.393130	-3.694770
H	-5.313510	2.988272	-4.965172
H	-2.897696	3.227344	-4.426423
C	0.901315	3.118982	-0.659957
C	0.174078	3.291051	0.689637
C	0.403523	4.190215	-1.644985
C	2.417730	3.315101	-0.427916
H	-0.903771	3.141597	0.553046
H	0.523139	2.529087	1.396317
C	0.436799	4.691104	1.259095
H	0.917719	4.088743	-2.606749
H	-0.663592	4.061664	-1.839468
C	0.670110	5.590944	-1.067860
H	2.791621	2.551191	0.260288
H	2.960891	3.200722	-1.373800
C	2.681145	4.716647	0.143805
H	-0.090785	4.791973	2.212677
C	-0.071061	5.746593	0.267723
C	1.944844	4.872027	1.481743
H	0.307785	6.340177	-1.779116
C	2.176948	5.777241	-0.844304
H	3.757307	4.837263	0.300992
H	-1.149737	5.628041	0.113300
H	0.090212	6.751901	0.672942
H	2.310203	4.130004	2.199087
H	2.144685	5.862032	1.906954
H	2.378193	6.780905	-0.452988
H	2.710660	5.688090	-1.797658

Zero-point correction= 1.150387 (Hartree/Particle)
Thermal correction to Energy= 1.202092
Thermal correction to Enthalpy= 1.203036
Thermal correction to Gibbs Free Energy= 1.068468
Sum of electronic and zero-point Energies= -3074.463028
Sum of electronic and thermal Energies= -3074.411323
Sum of electronic and thermal Enthalpies= -3074.410379
Sum of electronic and thermal Free Energies= -3074.544948

[(*S*)-Quinox-Ad$_3$]: III-C$_{linear}$

C	-2.338860	3.488562	1.705248
C	-2.846972	2.318780	2.315746
C	-1.809428	1.026708	0.739386
C	-1.250523	2.215161	0.149088
P	-1.455599	-0.612940	-0.061758
P	-0.111948	2.053785	-1.317104
Cu	0.281898	-0.258102	-1.511297
B	3.193268	-2.140558	-0.709652
O	4.310761	-1.327684	-0.783014
O	3.097698	-2.731143	0.536520
C	5.173967	-1.690727	0.310686
C	4.153659	-2.217208	1.370634
C	1.623249	-1.225870	-2.600319
H	2.448303	-0.544784	-2.864343
C	2.251477	-2.456497	-1.907582
C	-0.873209	3.292096	-2.442878
H	-1.108120	4.216814	-1.913240
H	-1.788057	2.873247	-2.865214
H	-0.185618	3.497821	-3.264678
C	-1.273537	-1.827912	1.353655
C	-3.039689	-0.880798	-1.051165
C	-0.236831	-1.212837	2.324289
H	-0.625827	-0.287560	2.758148
H	0.677753	-0.967904	1.771339
C	-2.554312	-2.183013	2.130663
H	-3.015686	-1.273997	2.522280
H	-3.274128	-2.660751	1.459343
C	-0.647318	-3.123919	0.781747
H	0.280587	-2.880674	0.257054
H	-1.316118	-3.591309	0.055315
C	-4.369164	-0.603222	-0.321969
H	-4.391158	0.426956	0.044642
H	-4.477155	-1.248137	0.551199
C	-3.044489	-2.327827	-1.588955
H	-3.138567	-3.034553	-0.759704
C	-2.950978	0.083676	-2.259555
H	-2.008152	-0.073637	-2.796121
H	-2.958763	1.119841	-1.901340
H	2.884627	-3.035950	-2.607179
C	6.109027	-2.785005	-0.209692
H	6.845989	-3.077334	0.542265
H	6.636191	-2.403517	-1.086187
H	5.548440	-3.672511	-0.513067
C	5.992599	-0.489342	0.751227
H	6.635866	-0.163460	-0.068957

H	6.629140	-0.757382	1.599922
H	5.362397	0.349220	1.040071
C	3.567976	-1.099426	2.231618
H	4.318009	-0.681056	2.907425
H	2.752899	-1.498053	2.836575
H	3.169568	-0.297277	1.608190
C	4.674568	-3.338778	2.256189
H	3.903924	-3.632990	2.972096
H	5.553118	-3.006426	2.817226
H	4.944392	-4.216455	1.668854
C	0.940248	-1.621168	-3.925594
H	0.428884	-0.745381	-4.347875
H	0.149606	-2.359058	-3.722783
C	1.870900	-2.184252	-5.008155
H	2.344230	-3.118419	-4.693284
H	2.669073	-1.468447	-5.231962
H	1.331000	-2.388961	-5.939103
H	1.468982	-3.152612	-1.581088
C	-1.208452	-2.519981	4.225386
H	-0.991379	-3.202298	5.055056
H	-1.626116	-1.603402	4.658039
C	-2.226063	-3.159440	3.270863
H	-3.148730	-3.385905	3.815265
C	-1.643733	-4.449491	2.677619
H	-1.433028	-5.172434	3.474002
H	-2.374217	-4.913572	2.004320
C	0.664469	-3.496305	2.876165
H	0.900026	-4.197898	3.685344
H	1.586406	-3.294311	2.327185
C	0.081450	-2.201799	3.456508
H	0.806238	-1.737164	4.134169
C	-0.354161	-4.118048	1.915961
H	0.065741	-5.028431	1.477855
C	-5.535910	-2.276317	-1.795743
H	-6.391607	-2.447866	-2.458400
H	-5.630906	-2.976392	-0.957591
C	-5.551736	-0.830447	-1.280588
H	-6.483507	-0.644179	-0.737188
C	-5.446539	0.134060	-2.468713
H	-6.297634	-0.003937	-3.144979
H	-5.478822	1.170951	-2.114572
C	-4.106044	-1.570212	-3.734799
H	-4.929659	-1.733131	-4.438872
H	-3.172114	-1.755155	-4.276784
C	-4.132411	-0.127505	-3.214815
H	-4.035830	0.569936	-4.052965

C	-4.224230	-2.536169	-2.549337
H	-4.203465	-3.568483	-2.911825
H	-2.096156	-2.539499	-2.096101
C	-2.652766	4.755709	2.251735
C	-3.436085	4.835778	3.369802
C	-3.657558	2.428505	3.470664
C	-3.941139	3.663766	3.984060
N	-1.541856	3.406815	0.610063
N	-2.583696	1.093972	1.795468
H	-2.250102	5.635680	1.764528
H	-3.674056	5.803112	3.796738
H	-4.559265	3.753462	4.869834
H	-4.035323	1.517152	3.918498
C	1.484348	2.847880	-0.765833
C	2.070065	1.970466	0.360770
C	1.388822	4.305176	-0.285081
C	2.438774	2.780430	-1.982071
H	1.402543	1.976925	1.231960
H	2.147723	0.933277	0.008880
C	3.458892	2.489292	0.756651
H	0.981678	4.937683	-1.081581
H	0.703787	4.381445	0.562283
C	2.784402	4.814800	0.111510
H	2.508555	1.748813	-2.342246
H	2.047389	3.392174	-2.803079
C	3.828885	3.296733	-1.584450
H	3.853909	1.867440	1.566204
C	3.346970	3.940856	1.240627
C	4.387596	2.412060	-0.463750
H	2.697703	5.850264	0.456364
C	3.722119	4.749130	-1.101353
H	4.487803	3.242617	-2.456442
H	2.692648	3.994634	2.118537
H	4.331934	4.311217	1.546732
H	4.460891	1.377010	-0.811905
H	5.396932	2.741214	-0.189684
H	4.713863	5.129862	-0.831587
H	3.339594	5.387391	-1.906353

Zero-point correction= 1.148021 (Hartree/Particle)
Thermal correction to Energy= 1.200317
Thermal correction to Enthalpy= 1.201261
Thermal correction to Gibbs Free Energy= 1.063526
Sum of electronic and zero-point Energies= -3074.460046
Sum of electronic and thermal Energies= -3074.407750
Sum of electronic and thermal Enthalpies= -3074.406806
Sum of electronic and thermal Free Energies= -3074.544541

[(*S*)-Quinox-Ad$_3$]: III-D$_{linear}$

C	-3.600178	0.617257	-2.413238
C	-2.803979	1.550290	-3.115399
C	-1.041991	1.102855	-1.727588
C	-1.849824	0.145790	-1.015702
P	0.748746	1.318761	-1.266984
P	-1.076801	-0.835884	0.364605
Cu	1.023184	0.036229	0.660553
B	3.690693	-1.530596	1.066753
O	4.954938	-1.063926	0.782092
O	3.127875	-2.154765	-0.036119
C	2.284584	0.149412	2.200335
H	3.095029	0.863771	1.977229
C	2.961433	-1.241595	2.405005
H	3.678332	-1.246388	3.239367
C	-2.272962	-0.633110	1.797201
C	-1.022106	-2.566975	-0.354991
C	1.478237	1.317590	-2.954447
C	-0.114178	-3.442337	0.542846
H	-0.552698	-3.554812	1.537216
H	0.861247	-2.962230	0.664720
C	-0.321471	-2.431156	-1.729906
H	0.648067	-1.938817	-1.595171
H	-0.924052	-1.812833	-2.404225
C	-2.378657	-3.265337	-0.539891
H	-3.045611	-2.645539	-1.146375
H	-2.856445	-3.402872	0.434738
C	-3.778441	-0.861492	1.556181
H	-4.135304	-0.211360	0.756509
H	-3.967402	-1.887513	1.233629
C	-1.807149	-1.566240	2.936583
H	-0.731474	-1.447720	3.105048
H	-1.983953	-2.608772	2.653932
C	-2.071817	0.833935	2.249697
H	-1.008773	1.021420	2.424767
H	-2.399483	1.513255	1.453595
H	1.363475	0.319229	-3.381545
H	0.976188	2.036113	-3.602969
H	2.543572	1.544272	-2.889971
C	5.119060	-1.086011	-0.643095
C	4.127859	-2.220634	-1.070700
C	6.578116	-1.350496	-0.975834
H	7.186721	-0.508521	-0.639429
H	6.714644	-1.462713	-2.055640
H	6.942655	-2.250222	-0.480116
C	4.696114	0.293436	-1.151706

H	4.834273	0.390606	-2.232043
H	5.303589	1.050795	-0.652062
H	3.649840	0.486422	-0.901614
C	3.466278	-1.997413	-2.421237
H	2.811305	-2.836416	-2.666323
H	4.220631	-1.914583	-3.209154
H	2.867193	-1.087444	-2.411985
C	4.754365	-3.612594	-1.006191
H	5.490628	-3.758300	-1.800687
H	3.970542	-4.364292	-1.120446
H	5.242300	-3.775318	-0.042717
C	1.617283	0.649709	3.491274
H	0.848066	-0.067944	3.808205
H	1.079777	1.587586	3.288200
C	2.564797	0.904971	4.668764
H	3.339439	1.625449	4.385176
H	3.068539	-0.011499	4.987808
H	2.031952	1.307267	5.537368
H	2.197404	-1.998523	2.621693
C	-1.491307	-4.481096	-2.555070
H	-2.117342	-3.871425	-3.216933
H	-1.369664	-5.459639	-3.033127
C	-2.174598	-4.643819	-1.189940
H	-3.151150	-5.120338	-1.323147
C	-1.296345	-5.508579	-0.275083
H	-1.786135	-5.643136	0.696341
H	-1.164632	-6.505247	-0.711722
C	-0.122171	-3.813589	-2.364514
H	0.366677	-3.687017	-3.336660
C	0.755132	-4.677620	-1.450458
H	0.922791	-5.659802	-1.907342
H	1.729547	-4.203497	-1.307072
C	0.068080	-4.831310	-0.089509
H	0.696140	-5.436030	0.571787
C	-4.083492	-1.509973	3.970246
H	-4.251947	-2.556480	3.690630
H	-4.656065	-1.324552	4.886184
C	-4.565041	-0.579606	2.849245
H	-5.627832	-0.757094	2.655106
C	-4.356450	0.879536	3.274571
H	-4.717630	1.555084	2.490536
H	-4.934113	1.094998	4.180726
C	-2.366755	0.191386	4.644285
H	-2.903362	0.398667	5.577045
H	-1.302948	0.370423	4.832410
C	-2.589690	-1.267113	4.223900

H	-2.234025	-1.936783	5.013025
C	-2.863893	1.121805	3.530219
H	-2.698853	2.164807	3.818359
C	-4.940600	0.400539	-2.811259
C	-5.453134	1.096511	-3.870845
C	-3.355997	2.254336	-4.211890
C	-4.654657	2.029028	-4.576930
N	-3.091041	-0.084400	-1.369499
N	-1.514347	1.768290	-2.753125
H	-6.479778	0.936084	-4.178959
H	-5.082623	2.565418	-5.415863
H	-2.723146	2.960305	-4.736234
H	-5.528252	-0.319738	-2.254677
C	0.888504	3.094567	-0.698029
C	0.055304	3.231676	0.592656
C	0.439003	4.166893	-1.703625
C	2.376972	3.320556	-0.344517
H	-1.005341	3.062298	0.372620
H	0.367731	2.468482	1.313332
C	0.243575	4.625379	1.202756
H	1.027840	4.095563	-2.624397
H	-0.607406	4.013461	-1.980080
C	0.626712	5.564825	-1.087792
H	2.707354	2.554813	0.365077
H	2.997109	3.226395	-1.244194
C	2.563381	4.719265	0.261015
H	-0.356809	4.696963	2.115179
C	-0.212838	5.685567	0.192104
C	1.726220	4.831214	1.542879
H	0.300735	6.316757	-1.813531
C	2.107944	5.782193	-0.748502
H	3.621800	4.863688	0.498350
H	-1.275213	5.548298	-0.040294
H	-0.101358	6.687956	0.620871
H	2.050677	4.077312	2.268571
H	1.873119	5.814321	2.004166
H	2.254508	6.784352	-0.329926
H	2.714224	5.719710	-1.659665

Zero-point correction= 1.150145 (Hartree/Particle)
Thermal correction to Energy= 1.201990
Thermal correction to Enthalpy= 1.202934
Thermal correction to Gibbs Free Energy= 1.067617
Sum of electronic and zero-point Energies= -3074.464554
Sum of electronic and thermal Energies= -3074.412709
Sum of electronic and thermal Enthalpies= -3074.411765
Sum of electronic and thermal Free Energies= -3074.547082

[(*S*)-Quinox-Ad$_3$]: TS$_{pro}$-D$_{branch}$

C	-3.965872	1.787634	1.588518
C	-3.869599	2.851000	0.661380
C	-2.152658	1.780642	-0.401953
C	-2.301154	0.663281	0.494054
P	-0.804073	1.776880	-1.685631
P	-1.300686	-0.873459	0.204616
Cu	0.109286	-0.278768	-1.468762
B	4.000712	-1.553280	-0.416012
O	5.319022	-1.391368	-0.775890
O	3.743454	-1.000957	0.826660
C	2.184135	-0.809546	-1.878874
H	2.798995	-0.349354	-2.663595
H	2.172970	-0.050278	-1.070437
C	2.880316	-2.099450	-1.345780
H	2.142487	-2.668243	-0.767611
C	3.362629	-2.985243	-2.495751
H	4.109443	-2.435836	-3.080890
H	2.517482	-3.180250	-3.164984
C	-0.666935	-1.372790	1.893979
C	-2.602228	-2.023481	-0.514077
C	-1.697340	2.459034	-3.131429
C	0.328327	3.147087	-1.103775
C	-1.938930	-3.403520	-0.717684
H	-1.714500	-3.854476	0.255172
H	-0.999721	-3.283160	-1.268032
C	-2.932506	-1.427897	-1.908297
H	-2.006250	-1.301798	-2.474690
H	-3.410996	-0.446798	-1.789849
C	-3.923489	-2.195500	0.258948
H	-4.409272	-1.226802	0.403190
H	-3.746222	-2.611211	1.252350
C	-0.306178	4.548871	-1.089803
H	-1.191598	4.559063	-0.449416
H	-0.637669	4.819655	-2.098178
C	1.555686	3.159107	-2.043473
H	1.246923	3.401679	-3.067240
H	2.015428	2.165243	-2.072751
C	0.812956	2.788307	0.316770
H	1.276620	1.795377	0.308307
H	-0.035115	2.745080	1.010069
C	3.964069	-4.305184	-2.019888
H	4.849666	-4.130479	-1.400693
H	4.266090	-4.935759	-2.860905
H	3.241242	-4.870921	-1.422595
C	-1.686172	-2.008191	2.855437

H	-2.527642	-1.327824	3.010600
H	-2.080041	-2.930702	2.418379
C	0.500751	-2.361149	1.661975
H	1.242495	-1.901928	1.004236
H	0.144395	-3.269488	1.167494
C	-0.071272	-0.101005	2.544080
H	0.665073	0.342996	1.866109
H	-0.856067	0.640176	2.720320
H	-2.322470	1.668471	-3.550618
H	-2.319799	3.309225	-2.849658
H	-0.971875	2.756523	-3.890781
C	5.890363	-0.410963	0.107700
C	4.989547	-0.547261	1.384064
C	5.733365	0.940710	-0.589052
H	6.246694	0.900102	-1.551698
H	6.155391	1.758871	-0.000355
H	4.678668	1.150210	-0.783730
C	7.360761	-0.726738	0.323954
H	7.799505	-0.036148	1.050363
H	7.898405	-0.618527	-0.620264
H	7.501848	-1.747454	0.679204
C	5.477479	-1.629142	2.345836
H	4.708751	-1.805519	3.101192
H	6.398951	-1.330181	2.851685
H	5.656423	-2.568494	1.817899
C	4.747420	0.755866	2.131829
H	5.694238	1.199884	2.452686
H	4.143120	0.566498	3.022073
H	4.216005	1.475347	1.507971
C	3.037022	3.837298	-0.136824
H	3.515016	2.853328	-0.141695
H	3.784206	4.558746	0.212351
C	2.576563	4.199797	-1.555088
H	3.435535	4.196899	-2.233066
C	1.930028	5.591702	-1.539158
H	2.659373	6.340033	-1.209386
H	1.614828	5.871712	-2.550843
C	0.719614	5.581488	-0.595568
H	0.250092	6.570115	-0.587067
C	1.174509	5.215085	0.824410
H	1.886504	5.961044	1.194907
H	0.316599	5.218637	1.506448
C	1.829030	3.827117	0.808344
H	2.152454	3.556261	1.818577
C	-5.177731	-2.535163	-1.899295
H	-5.863952	-3.191653	-2.446490

H	-5.678183	-1.566680	-1.782336
C	-4.863139	-3.133791	-0.522225
H	-5.790382	-3.251656	0.047999
C	-4.192876	-4.502397	-0.706306
H	-3.987310	-4.955594	0.270721
H	-4.867752	-5.180229	-1.241327
C	-3.871269	-2.361394	-2.681334
H	-4.077844	-1.915915	-3.659815
C	-2.886460	-4.330515	-1.493068
H	-2.399162	-5.302108	-1.619896
C	-0.435491	-1.066999	4.823151
H	0.027159	-1.298304	5.789354
H	-1.242737	-0.350036	5.013019
C	-1.004072	-2.345324	4.191988
H	-1.746768	-2.787096	4.864214
C	0.131733	-3.348757	3.944614
H	0.607547	-3.621114	4.893577
H	-0.272377	-4.269734	3.508568
C	0.603664	-0.451236	3.877132
H	1.007797	0.466639	4.317088
C	1.739553	-1.450206	3.628841
H	2.245880	-1.687283	4.572374
H	2.476134	-1.018918	2.946696
C	1.164642	-2.723508	2.997914
H	1.973687	-3.433925	2.802257
C	-3.180320	-3.718621	-2.869128
H	-3.820588	-4.390138	-3.452626
H	-2.243604	-3.582003	-3.418431
N	-3.177019	0.689857	1.469301
N	-2.940257	2.824343	-0.327208
C	-4.917641	1.855721	2.633437
C	-5.741799	2.942864	2.728914
C	-4.738507	3.961553	0.777486
C	-5.654091	4.001455	1.792376
H	-4.969142	1.029325	3.332156
H	-6.475711	2.998042	3.524469
H	-6.322649	4.849329	1.886282
H	-4.648258	4.758793	0.049443
O	0.087491	-1.775517	-3.158039
H	1.084372	-1.342546	-2.629723
C	-0.058483	-1.225417	-4.430212
H	0.258914	-1.922167	-5.220208
H	-1.110370	-0.963109	-4.631398
H	0.534636	-0.300186	-4.548168

Zero-point correction= 1.197405 (Hartree/Particle)
Thermal correction to Energy= 1.253470

Thermal correction to Enthalpy=	1.254414
Thermal correction to Gibbs Free Energy=	1.108838
Sum of electronic and zero-point Energies=	-3190.132732
Sum of electronic and thermal Energies=	-3190.076668
Sum of electronic and thermal Enthalpies=	-3190.075723
Sum of electronic and thermal Free Energies=	-3190.221299
